# Anion–π Catalysis Enabled by the Mechanical Bond**

**DOI:** 10.1002/anie.202115961

**Published:** 2022-02-03

**Authors:** John R. J. Maynard, Bartomeu Galmés, Athanasios D. Stergiou, Mark D. Symes, Antonio Frontera, Stephen M. Goldup

**Affiliations:** ^1^ Chemistry University of Southampton Highfield Southampton S017 1BJ UK; ^2^ Department of Chemistry Universitat de les Illes Balears Crta de Valldemossa km 7.5 07122 Palma de Mallorca Baleares Spain; ^3^ WestCHEM School of Chemistry University of Glasgow, Joseph Black Building University Avenue Glasgow G12 8QQ UK

**Keywords:** Anion–π Catalysis, DFT Calculations, Mechanical Bonds, Rotaxanes, Supramolecular Chemistry

## Abstract

We report a series of rotaxane‐based anion–π catalysts in which the mechanical bond between a bipyridine macrocycle and an axle containing an NDI unit is intrinsic to the activity observed, including a [3]rotaxane that catalyses an otherwise disfavoured Michael addition in >60 fold selectivity over a competing decarboxylation pathway that dominates under Brønsted base conditions. The results are rationalized by detailed experimental investigations, electrochemical and computational analysis.

## Graphical abstract

A series of rotaxane‐based catalysts in which the mechanical bond between a bipyridine macrocycle and an NDI‐containing axle is intrinsic to their activity is reported. Computational analysis suggests that the high selectivity observed (>60 fold for Michael addition over a competing decarboxylation pathway) is due to π‐stacking of the mechanically bonded components that leads to high polarizability.

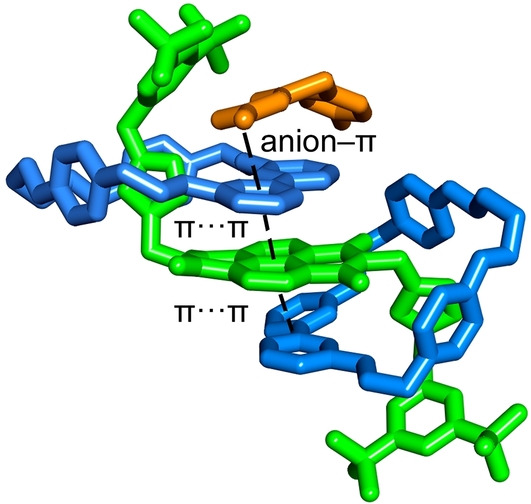

## Introduction

Anion–π interactions,^[1a]^ the counterintuitive cousins of cation–π interactions, arise when an anionic species interacts with an aromatic surface possessing a positive quadrapole moment perpendicular to the aromatic plane.^[2]^ They remained largely overlooked^[3]^ until their nature was delineated through molecular modelling in 2002.^[1]^ Since then, thanks to extensive efforts by many research groups, anion–π interactions are now recognised as important non‐covalent interactions in both the solution‐^[4]^ and solid‐state,^[5]^ and evidence is emerging that they play a role in the structure and function of enzymes.^[6]^

Matile and co‐workers coined the term anion–π catalysis^[7]^ to describe reactions that are accelerated by stabilizing anionic transition states and intermediates through interaction with a catalyst containing a π‐acidic aromatic surface. The anion–π catalysis concept^[8]^ has since been applied to a range of reactions, typically making use of electron‐deficient naphthalene diimides (NDIs) and their extended homologues,^[9]^ or electron‐deficient heteroaromatic species,^[10]^ including enantioselective examples.^[11]^ More recently, examples have been reported in which the surfaces of fullerenes or carbon nanotubes display anion–π catalysis,^[12]^ as well examples in which an electron deficient π surface is engaged in π–π stacking interactions,^[13]^ the results of which, supported by computational analysis, suggest that the polarizability of the π surface, as well as its permanent quadrapole moment, plays a key role in catalyst activity.

Mechanically interlocked molecules^[14]^ such as rotaxanes and catenanes have recently begun to attract increased interest as scaffolds for the development of new catalysts.^[15]^ The key features of the mechanical bond that suggest this is a promising avenue for research are the ability of the components to undergo large amplitude motion,^[16]^ the potential for multiple cooperating functional groups to be projected into the cavity created by the mechanical bond,^[17]^ the sterically crowded environment of the mechanical bond itself^[18]^ including the potential for the mechanical bond to direct reactions that otherwise would not occur without the steric confinement it provides,^[19]^ and the potential for enantioselective interlocked catalysts to rely on molecular chirality that arises due to the symmetry properties of the mechanical bond itself.[^20^, ^21^]

Here we report the development of rotaxane‐based, highly selective anion–π catalysts that, due to the cooperation of several convergent functional groups and the confined environment of the mechanical bond, deliver remarkably high selectivity in one of the benchmark reactions of the field, the Michael addition of a malonic acid half‐thioester (MAHT) to β‐nitrostyrene.^[9d]^ Experimental and computational data suggest the selectivity of the best catalysts is due to the polarizability of a π‐stacked structure formed by interaction of a bipyridinium moiety and the NDI unit,^[22]^ with the bipyridinium unit, in some cases, providing the π surface that interacts directly with the substrate.

## Results and Discussion

**Scheme 1:**
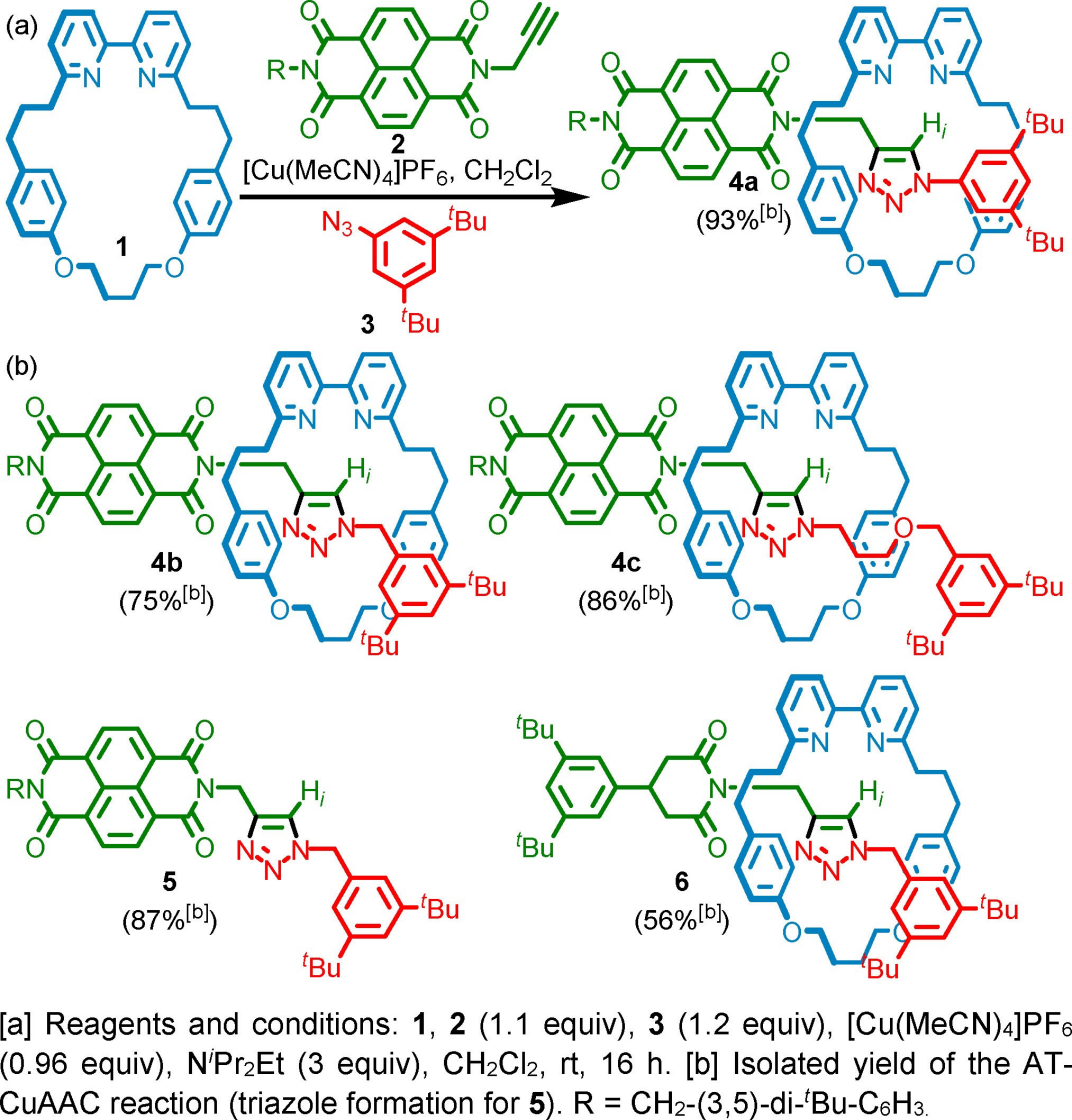
a) Synthesis of rotaxane **4** **a** using the AT‐CuAAC reaction^[a]^ and b) structures of rotaxanes **4** **b**, **4** **c** and **6**, and axle **5**.

**Figure 1:**
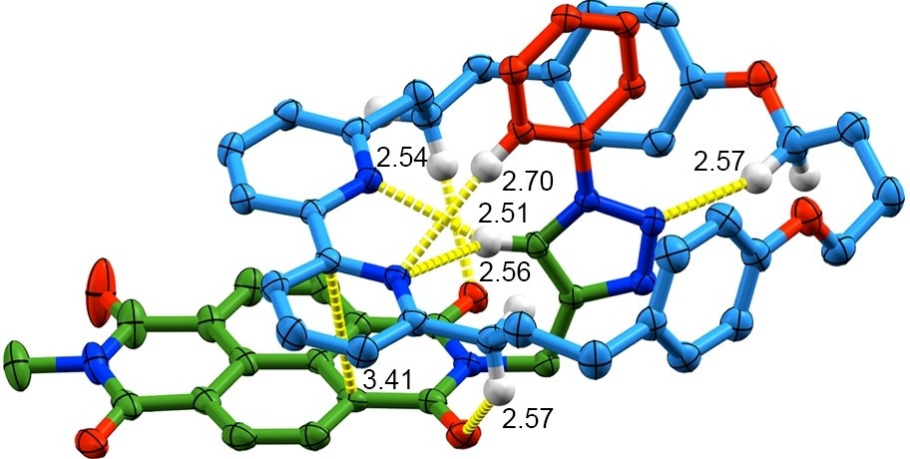
SCXRD structure of rotaxane **4** **a** in ellipsoid representation (Hs in ball‐and‐stick) with selected intercomponent interactions highlighted (counterions, majority of H omitted and stopper units truncated for clarity; measurements in Å; colours as in Scheme 1 except N [blue], O [red], H [white]).

[2]Rotaxanes **4** were prepared using an active template^[23]^ Cu‐mediated alkyne‐azide cycloaddition (AT‐CuAAC)^[24]^ reaction. (Scheme 1). Our expectation was that the bipyridine‐triazole cavity produced in the AT‐CuAAC approach would act as a Brønsted base,[^7^, ^9^, ^25^] and that the short alkyl linker between the NDI unit and triazole would facilitate π–π stacking of the bipyridine and NDI units. Rotaxane **6**, which lacks the NDI unit, was synthesized using a similar strategy for comparison (see Supporting Information). Rotaxanes **4**, axle **5** and rotaxane **6** were fully characterized by NMR and MS (see Supporting Information). Comparison of the ^1^H NMR spectra of rotaxanes **4** revealed that although the triazole proton H_*i*_ resonance appears at high ppm in all cases, presumably due to C−H⋅⋅⋅N H‐bonding between the triazole and bipyridine unit[^24b^, ^24c^, ^26^] (Figure S54), it appears at progressively lower chemical shift in the series **4** **a**–**4** **c** (10.2, 9.4 and 9.1 ppm respectively, Figure S55). This effect was tentatively assigned to increased co‐conformational freedom as the axle length is increased. Single crystal X‐ray diffraction (SCXRD) analysis of **4** **a** confirmed its interlocked nature and the proposed C−H⋅⋅⋅N interaction between the bipyridine Ns and H_*i*_ alongside other weak interactions and reveals the anticipated π–π stacking interaction between the macrocycle bipyridine and axle NDI (Figure 1).

The behaviour of rotaxanes **4** as anion–π catalysts was assessed through their performance in the Michael addition of malonic acid half‐thioester **7** to β‐nitrostyrene (**11**) (Table 1, entries 2–4), a key benchmarking reaction in anion‐π catalysis.^[9d]^ Under Brønsted base catalysis, the major product of this reaction is thioester **10**, the product of deprotonation and subsequent decarboxylation of **7**, whereas in the presence of an anion‐π catalyst planar enolate **9** is stabilized in the equilibrium, allowing the addition/decarboxylation product **12** to form preferentially. Broadly speaking, the key parameters that determine catalyst performance are thus Brønsted basicity (determines position of the equilibrium between **7** and **8** and thus significantly affects rate) and the ability of the catalyst to stabilise **9** and the subsequent transition state leading to **12** (determines selectivity). Reactions mediated by NEt_3_ (entry 1), macrocycle **1** (entry 5), an equimolar combination of axle **5** and macrocycle **1** (entry 6), axle **5** alone and rotaxane **6** were also assessed for comparison.

**Table 1:** Comparison of the outcome of the reaction of **7** with **11** in the presence of rotaxanes **4**, macrocycle **1**, axle **5** and control rotaxane **6**.

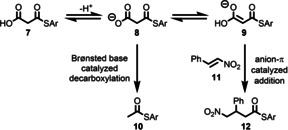				
		
Entry	Catalyst	**12**/**10** ^[a]^ (reaction time^[b]^)		
		*d*_8_‐THF, 30 °C	CDCl_3_, 30 °C	CDCl_3_, 7 °C
1	NEt_3_	0.4	3.2 (40 h)	8.7 (60 h)
2	**4** **a**	n.r.^[c]^	10.7 (16 h)	n.d.^[d]^
3	**4** **b**	4.4 (60 h)	11.9 (6 h)	46.2 (40 h)
4	**4** **c**	n.r.^[c]^	10.3 (16 h)	n.d.^[d]^
5	**1**	n.r.^[c]^	7.1 (60 h)	n.d.^[d]^
6	**1**+**5**	n.r.^[c]^	7.2 (60 h)	n.d.^[d]^
7	**5**	n.r.^[c]^	n.d.^[d]^	n.d.^[d]^
8	**6**	2.4 (60 h)	8.0 (16 h)	33.3 (40 h)

[a] Determined by in situ ^1^H NMR analysis. [b] Time required for >95 % conversion of **7**. [c] No reaction was observed after 16 h and so the reaction was stopped. [d] Not determined. Ar=4‐OMe‐C_6_H_4_.

The key conclusions of this study are i) **4b** is an effective anion‐π catalyst (entry 3), delivering **12** selectively in THF at 30 °C^[27]^ (**12**/**10**=4.4) and with enhanced activity (16 h vs 60 h for >95 % conversion) and selectivity (11.9) in CDCl_3_, which was improved further at lower temperature (46.2); ii) rotaxanes **4** **a** and **4** **c** (entries 2 and 4 respectively) display reduced activity and slightly reduced selectivity compared with **4** **b** in CDCl_3_ and no activity over 16 h in THF; iii) non‐interlocked macrocycle **1** (in CDCl_3_, entry 5) and rotaxane **6** (in THF or CDCl_3_, entry 8), both of which lack an NDI unit, are active catalysts, albeit less selective than rotaxanes **4**; iv) **4** **b** and **6** display similar catalytic activity; v) NDI axle **5** is not a competent catalyst and combining **1** and **5** results in the same outcome as **1** alone.

These results are surprising for several reasons. Firstly, it is striking that **4** **b** displays selectivities equivalent or higher than those previously reported for simple mono‐NDI‐based catalysts (Table S4). Indeed, the reported example that is most comparable in selectivity to **4** **b** (**12**/**10**=4.4, THF, 20 °C, entry 7) includes a sulfone‐substituted NDI unit, which is proposed to enhance the π‐acidity of the system and so selectivity for **12**.^[9e]^ Secondly, it is not immediately obvious why rotaxanes **4** **a** and **4** **c** are inactive in THF, whereas **4** **b** performs reasonably well. Given that **4** **a** and **4** **c** produce **12** with good selectivity in CDCl_3_, albeit with lower activity and slightly reduced selectivity compared with **4** **b**, we considered that the differences observed may relate primarily to differences in their Brønsted basicity. In keeping with this, when a mixture of rotaxanes **4** was titrated with MsOH in CDCl_3_ and the changes observed by ^1^H NMR, selective protonation of **4** **b** was observed (Figure S56).

The observed lower basicity of **4** **c** compared with **4** **b** was tentatively rationalised by considering that the protonated bipyridinium is expected to be stabilised by H‐bonding to the triazole unit. This effect is likely to be reduced by the increased co‐conformational flexibility of **4** **c**, as has been observed in crown ether‐ammonium‐based rotaxanes.^[25]^ The origin of the lower basicity of **4** **a** appears to run counter to this argument but direct conjugation of the triazole unit with the aromatic ring of the stopper unit can be expected to reduce its basicity and so reduce the favourability of this stabilising interaction. Thus, the lower activity of **4** **a** and **4** **c** compared with **4** **b** appears to be due to the initial step of the reaction, deprotonation of **7** to give carboxylate salt **8**, being less favourable in the former cases. Similarly, the lack of activity of **5** under all conditions examined was attributed to the low basicity of the isolated triazole unit.

Thirdly, we were extremely surprised to observe that rotaxane **6** (in all solvents examined) and macrocycle **1** (in CDCl_3_) deliver **12** in much higher selectivity than a simple Brønsted base such as NEt_3_, albeit less selectively than rotaxanes **4**. These results suggest that the protonated bipyridine unit may operate as a π‐acidic surface in this context, with the lower activity of **1** attributed to the lower basicity of the non‐interlocked macrocycle whereas, conversely, the similar activity of **4** **b** and **6** can be attributed to the similarity of their proton binding pocket. This proposal is consistent with previous observations of anion‐π interactions involving protonated or metal coordinated pyridine units in the solid state^[28]^ and reports of alkylated pyridinium‐based anion–π catalysts.^[10a]^

**Figure 2:**
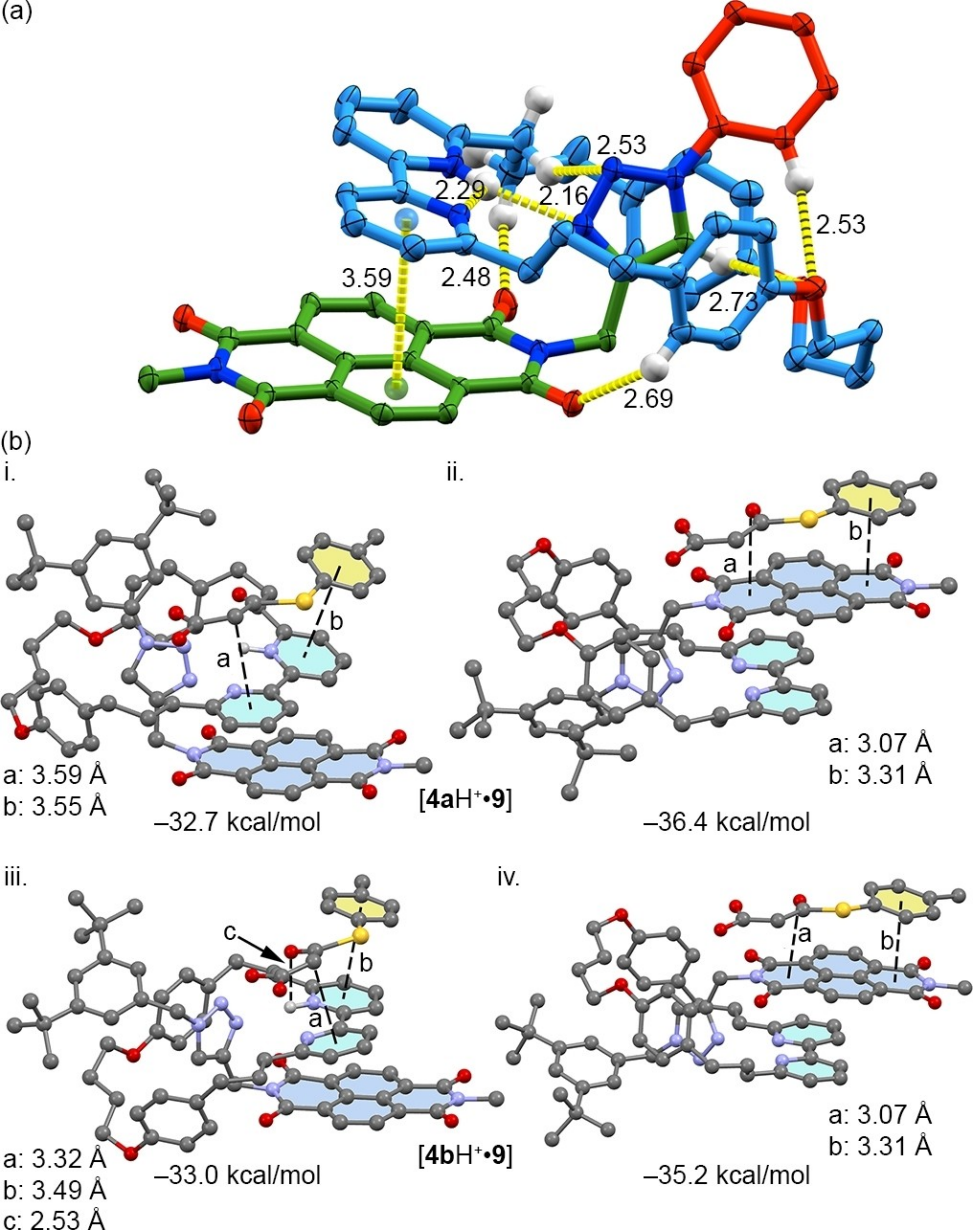
a) SCXRD structure of **4 a**H ⋅ PF_6_^−^ in ellipsoid representation (Hs in ball‐and‐stick) with selected intercomponent interactions highlighted (counterions, majority of H omitted and stopper units truncated for clarity; measurements in Å; colours as in Scheme 1 except N [blue], O [red], H [white]). b) BP86‐D3/def2‐TZVP optimized geometries (selected distances indicated) of **4 a**H^+^ and **4 b**H^+^ complexed with enolate **9** via the bipy moiety (i and iii respectively) or the NDI moiety (ii and iv respectively).

To rationalise these observations, as well as to attempt to rationalise the different selectivities of rotaxanes **4**, macrocycle **1** and rotaxane **6**, we turned to DFT molecular modelling. Optimised structures (Turbomole 7.2,^[29]^ PB86‐D3/def2‐TZVP level of theory) of macrocycle **1** and truncated models of **4** **a**–**c**, **6**, their protonated structures (**1**H^+^, **4** **a**–**c**H^+^, **6**H^+^) and their complexes with enolate **9** ([**1**H ⋅ **9**], [**4** **a**–**c**H ⋅ **9**], [**6**H ⋅ **9**]) were computed and their properties analysed (Supporting Information section S7). The minimum energy structures of rotaxanes **4** **a**–**c** (Figure S57) were found to be arranged such that a π–π stacking interaction was maintained between the bipyridine and NDI units, and a C−H⋅⋅⋅N interaction was present between the bipyridine unit and the triazole C−H, as observed in the solid‐state structure of **4** **a** (Figure 1). Protonated **4** **a**–**c**H^+^ structures (Figure S58) were found to maintain the NDI‐pyridine stacking interaction but, in this case, the protonated bipyridine unit engages in an H‐bond with the triazole unit. This prediction is corroborated by a SCXRD structure of **4 a**H^+^ (Figure 2a) obtained during catalysis screening experiments (see Supporting Information section S3.2 for details); the predicted π‐π stacking interactions and N−H⋅⋅⋅N H‐bond are both observed in the solid state. In keeping with our experimental observations, the p*K*_a_ difference between **4** **a** and **4** **b** was computed to be 1.47 units in THF and 1.35 units in CHCl_3_ (Supporting Information section 7.3). This was attributed to a stronger N⋅⋅⋅H bond formed in protonated **4** **b**, with a shorter distance and better directionality, combined with the lower basicity of the aryl triazole unit of **4** **a** (Supporting Information section S7.3).

The molecular electrostatic potential (MEP) values of **4** **a**–**c** (Figure S58) are large and positive over the NDI unit, in keeping with its established applications in anion–π catalysis.[^7^, ^9^, ^11^] The MEP value over the bipyridine is close to neutral. In contrast, the MEP values of **4** **a**–**c**H^+^ (Table 2, entries 1–3; Figure S59) associated with the NDI unit and protonated pyridine units are similar and larger than those observed in the neutral state, suggesting that either the NDI or the bipyridine motif can stabilise enolate **9**. The increase in MEP on protonation is consistent with electrochemical analysis; the reduction potential of **4** **a**–**c**, a proxy for *E*_LUMO_ of the NDI unit,[^8a^, ^30^] shifts to less negative potentials in the presence of TsOH (Δ*E*_1/2_=+190, +160 and +90 mV respectively; Supporting Information section S8).

**Table 2:** Computed properties of selected catalysts and complexes with **9**.

Entry	Cat.	MEP^[a]^ [kcal mol^−1^]	α_⊥_ [a.u.]^[b]^	*E*_int_ [kcal mol^−1^]^[c]^
1^[d]^	**4 a**H^+^	+71 (+69)	849.9^[e]^	−32.7 (−36.4)
2^[d]^	**4 b**H^+^	+70 (+62)	864.9^[e]^	−33.0 (−35.2)
2b^[d]^	**4 c**H^**+**^	+69 (+65)	851.4^[e]^	−33.7 (−30.1)
3	**1**H^+^	+87	426.7^[f]^	−34.3 (anion‐π),^[g]^ −41.4 (HB)^[g]^
4	**5**	+23	633.8^[e]^	−20.8
5	**6**H^+^	+65	735.3^[f]^	−32.4
6	**13**H^+^	+69	1532.0^[f]^	−47.3
7	**13**H_2_^2+^	+96	1532.1^[f]^	−51.3

[a] Molecular electrostatic potential over the π‐acidic surfaces. [b] Polarizability perpendicular to the mean plane of the catalytic surface. [c] Substrate‐catalyst interaction energy in CHCl_3_. [d] Values in parenthesis correspond to the NDI surface. [e] α_⊥_ of NDI. [f] α_⊥_ of bipy. [g] See Figure S68.

Considering the similar values of MEP calculated for the NDI and bipyridine π surfaces, both possible catalyst‐substrate complexes were computed for [**4 a**–**c**H ⋅ **9**] (Figure 2b and Figure S65). In the case of **4 a**H^+^ and **4 b**H^+^, binding of **9** to the NDI face of the catalyst was found to be slightly preferred (entries 1 and 2), as a result of increased π‐π interactions between the electron rich aromatic substituent of the thioester and the larger aromatic surface provided by the NDI. In the case of **4 c**H^+^, binding of **9** to the bipy unit was found to be preferred. Relatively small differences in complex energies were found for [**4** **a**–**c**H ⋅ **9**], which is consistent with the small difference in their selectivities observed in CDCl_3_ and reinforces the point that the different reaction rates observed between **4** **a** and **4** **b** and **4** **c** are most likely due to the p*K*_a_ difference between these catalysts.

Perhaps unsurprisingly, the optimised structure of [**1**H^+^ ⋅ **9**] was found to be a threaded complex, in which enolate **9** H‐bonds to the protonated bipyridinium, which was predicted to be 7.1 kcal mol^−1^ more stable than the π‐stacked structure (Figure S68). The competition between the threaded structure and the stacked structure may explain the lower selectivity for **12** observed in reactions catalysed by **1**H^+^. The same calculations for rotaxane **6**H^+^, which lacks the NDI moiety but in which the cavity of the macrocycle is blocked, show that the binding of **9** is around 3 kcal mol^−1^ less favourable than for rotaxanes **4** **a**–**c**H^+^, which is in keeping with the smaller MEP of **1**H^+^. Based on these results, regardless of which π‐surface mediates anion–π catalysis in the case of rotaxanes **4**, the pyridinium moiety appears to be responsible for the behaviour of rotaxane **6** and may well play a role for macrocycle **1**, albeit in competition with a threaded, hydrogen bonded structure.

Although MEP can be used to readily identify surfaces suitable for anion‐π catalysis, it is typically not sufficient to fully explain the trends observed as catalyst polarizability can lead to enhanced stabilisation of the substrate‐catalyst complex.[^12^, ^13^] Thus we calculated the polarizability of **4** **a**‐**c**H^+^, **6**H^+^ and **1**H^+^ perpendicular to the π‐surface. This revealed that, as expected, the most selective catalyst is also the most polarisable structure (**4b**H^+^), which suggests that the π‐stacking interaction between the bipyridinium and NDI unit, regardless of which surface is in direct contact with the substrate, is responsible for the high activity observed, as previously observed in oligo‐NDI and NDI‐fullerene conjugate‐based catalysts.[^12^, ^13^] Similarly, the polarizability of **6**H^+^ perpendicular to the bipyridine unit was calculated to be larger than **1**H^+^ thanks to the stacking interaction with the piperidine‐2,6‐dione unit (Figure S66), which, alongside the competition between threaded and stacked complexes for **1**, explains the higher selectivity demonstrated by **6**. These results clearly emphasize that both electrostatic and polarizability effects are important rationalizing anion–π catalysis.

These calculations, in conjunction with the experimental results presented, suggest that the key benefits of the mechanical bond in **4** **b** are threefold: i) it enhances the basicity of the bipyridine unit, increasing catalyst activity by favouring deprotonation of the substrate; ii) it enforces a stacked orientation for the bipyridinium and NDI units, which increases the polarizability of protonated species, favouring anion‐π catalysis and iii) the axle blocks the cavity of the bipyridine ring, preventing the formation of a threaded structure with the substrate, as observed for **1**H^+^.

**Figure 3:**
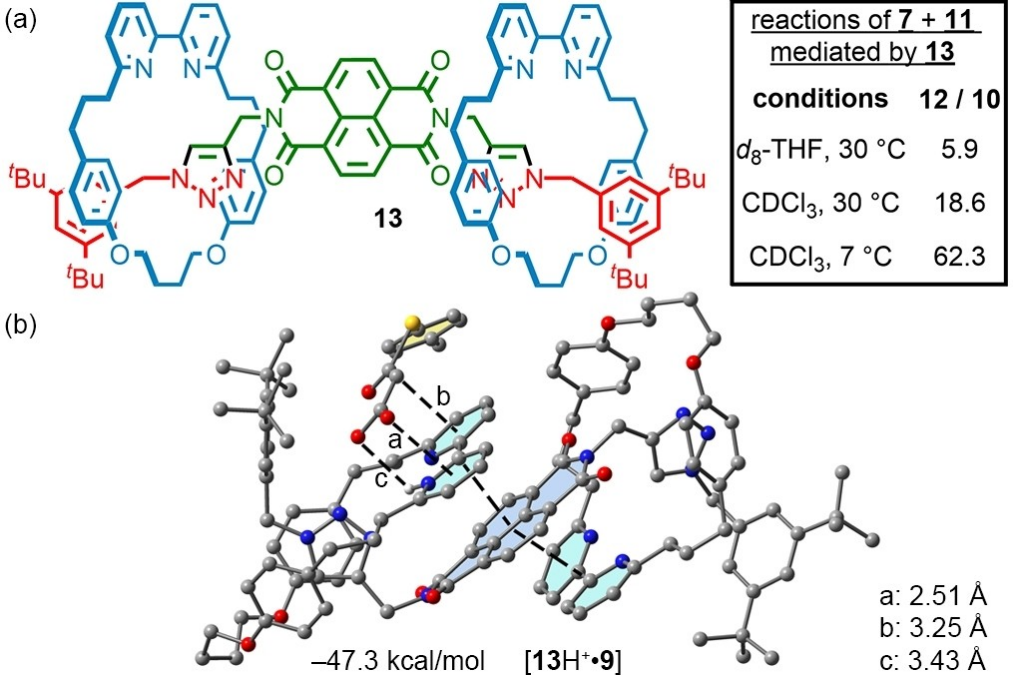
a) Structure of [3]rotaxane **13** and summary of its catalytic behaviour. b) computed structure of [**13**H^+^ ⋅ **9**] with key values labelled.

To take further advantage of all of these effects while increasing the impact of π‐stacking on catalyst activity, we synthesised [3]rotaxane **13** (Figure 3) an analogue of [2]rotaxane **4** **b**, using the AT‐CuAAC reaction^[24]^ of a simple bis‐propargyl NDI in excellent yield (Supporting Information section S2.1). Molecular modelling indicates that **13** is structurally similar to rotaxane **4** **b** in that in the neutral state it is folded such that both bipyridines π‐stack with the NDI unit (Figure S60). Either single or double protonation, to give **13**H^+^ or **13**H_2_^+^ respectively, is predicted to result in a folded structure in which one neutral and one protonated, or two protonated bipyridines stack with the NDI core respectively (Figure S62). Importantly, in both cases the NDI is blocked such that it is unavailable to interact with enolate **9**, suggesting that any anion‐π catalysis that arises in the case of **13** would take place through interaction of **9** with the bipyridium unit.

When rotaxane **13** was applied in the reaction of **7** with **11**, extremely high levels of activity and selectivity were observed, even compared with rotaxane **4** **b**. In THF, the reaction was complete after 16 h with a high selectivity for **12** (5.9) whereas In CDCl_3_ the reaction was complete in just 3 h with still higher chemoselectivity (18.6). Most strikingly, the activity of **13** remained high even at 7 °C in CDCl_3_, with only 16 h required for complete conversion of **7** and a significant further enhancement in chemoselectivity (62.3), which is one of the highest values reported to date.^[11f]^

Calculations suggest that the excellent selectivity observed in the case of catalyst **13** is due to the high polarizability of **13**H^+^, which is almost double that of **4b**H^+^, rather than the value of MEP, which is actually calculated to be lower for **13**H^+^ than **4b**H^+^ (Table 2, entry 6). Double protonation to give **13**H_2_^2+^, which has a much higher calculated MEP (entry 7), was also considered but seems unlikely under the reaction conditions; *E*_1/2_ for the reduction of **4** **b** in the presence of 1 equiv. of TsOH is more negative than that of **13** in the presence of 2 equiv. TsOH (−1.07 vs. −1.01 V). This suggests that even in the presence of TsOH, a much stronger acid that **7**, the doubly protonated species does not dominate (Supporting Information section S8). Regardless, the calculated values of *E*_int_ for [**13**H^+^ ⋅ **9**] (Figure 3b) and [**13**H_2_^2+^ ⋅ **9**] are similar, in line with their comparable calculated polarisabilities (entries 6 and 7), and both are higher than [**4 b**H^+^ ⋅ **9**]. This is consistent with the excellent selectivity observed for **13** and the suggestion that polarizability is once again the key factor in determining catalytic performance.

## Conclusion

A series of interlocked anion–π catalysts have been prepared that combine a π‐acidic NDI unit in the axle component with a basic site provided by a bipyridine‐triazole cavity. These catalysts were found to be highly selective in an established benchmarking reaction for anion–π catalysis, with a dependence of catalyst activity and selectivity on the length of azide half‐axle used in the AT‐CuAAC synthesis. The selectivity obtained with readily accessible [3]rotaxane catalyst **13** is comparable to the most effective example previously reported.^[11f]^ Control experiments demonstrated the importance of the interlocked structure and the NDI unit to catalyst selectivity. Computational studies strongly suggest that the high selectivities observed, particularly for **13**, are due to π‐stacking between the protonated bipyridine unit and the NDI surface which results in a highly polarisable, π‐acidic surface that can efficiently stabilise the planar enolate, which is corroborated by solid state structures of a [2]rotaxane catalyst in both neutral and protonated forms. In addition to the ability of the mechanical bond to place all the required units in the required arrangement with minimal synthetic effort, the solubility of the rotaxane catalysts, which can be an issue in the case of NDI‐based molecules,^[31]^ makes this scaffold particularly attractive.

A key limitation of the structures presented appears to be their basicity which, although enhanced by the mechanical bond, remains low enough that even in the case of relatively acidic substrate **7**, rotaxanes **4** **a** and **4** **c** are inactive in THF and reactions mediated by **4** **b** are slow at reduced temperatures in CDCl_3_. Indeed, attempts to apply these catalysts to other previously studied anion‐π catalysed reactions with less acidic substrates[^9b^, ^11e^, ^32^] revealed reaction rates that were impractically slow, requiring multiple weeks to observe even trace amounts of product (Supporting Information section S9). Future work will focus on overcoming this limitation by engineering more basic functional groups into the axle of suitable structures, as well as adding electron‐withdrawing groups to the NDI core to enhance π‐acidity,^[8a]^ both while maintaining the π–π stacking interactions that deliver high polarizability and thus strong anion‐π interactions for catalysis. We are also exploring chiral structures that take advantage of the mechanical bond[^20^, ^21^] to deliver enantioselective examples.

## Author Contributions

J.R.J.M. conceived the project and developed the idea in collaboration with S.M.G. J.R.J.M. carried out all experimental and synthetic work. B.G. performed the computational modelling and analysed the data obtained in collaboration with A.F., who prepared the discussion presented in the Supporting Information. A.D.S. contributed to the collection of the electrochemical data and its preliminary analysis, which was supervised by M.D.S. J.R.J.M. prepared the initial manuscript draft with input from A.F. on computational aspects. The text was finalised by S.M.G. with all authors providing input.

## Conflict of interest

The authors declare no conflict of interest.

1

## Supporting information

As a service to our authors and readers, this journal provides supporting information supplied by the authors. Such materials are peer reviewed and may be re‐organized for online delivery, but are not copy‐edited or typeset. Technical support issues arising from supporting information (other than missing files) should be addressed to the authors.

Supporting Information

Supporting Information

Supporting Information

## Data Availability

Data (characterization data for reported compounds) is available from the University of Southampton data repository (https://doi.org/10.5258/SOTON/D2121).

## References

[1a] D. Quiñonero , C. Garau , C. Rotger , A. Frontera , P. Ballester , A. Costa , P. M. Deyà , Angew. Chem. Int. Ed. 2002, 41, 3389–3392; 10.1002/1521-3773(20020916)41:18<3389::AID-ANIE3389>3.0.CO;2-S 12298041

[1b] M. Mascal , A. Armstrong , M. D. Bartberger , J. Am. Chem. Soc. 2002, 124, 6274–6276; 12033854 10.1021/ja017449s

[1c] I. Alkorta , I. Rozas , J. Elguero , J. Am. Chem. Soc. 2002, 124, 8593–8598. 12121100 10.1021/ja025693t

[2] Selected general reviews:

[2a] P. Gamez , T. J. Mooibroek , S. J. Teat , J. Reedijk , Acc. Chem. Res. 2007, 40, 435–444; 17439191 10.1021/ar7000099

[2b] B. L. Schottel , H. T. Chifotides , K. R. Dunbar , Chem. Soc. Rev. 2008, 37, 68–83; 18197334 10.1039/b614208g

[2c] A. Frontera , P. Gamez , M. Mascal , T. J. Mooibroek , J. Reedijk , Angew. Chem. Int. Ed. 2011, 50, 9564–9583; 10.1002/anie.201100208 21928463

[2d] P. Gamez , Inorg. Chem. Front. 2014, 1, 35–43;

[2e] M. Giese , M. Albrecht , K. Rissanen , Chem. Rev. 2015, 115, 8867–8895; 26278927 10.1021/acs.chemrev.5b00156

[2f] X. Kan , H. Liu , Q. Pan , Z. Li , Y. Zhao , Chin. Chem. Lett. 2018, 29, 261–266;

[2g] I. A. Rather , S. A. Wagay , R. Ali , Coord. Chem. Rev. 2020, 415, 213327.

[3] For an excellent overview of the history of anion-π interactions see: P. Ballester , Acc. Chem. Res. 2013, 46, 874–884. 22621170 10.1021/ar300080f

[4] Selected accounts and examples:

[4a] R. E. Dawson , A. Hennig , D. P. Weimann , D. Emery , V. Ravikumar , J. Montenegro , T. Takeuchi , S. Gabutti , M. Mayor , J. Mareda , C. A. Schalley , S. Matile , Nat. Chem. 2010, 2, 533–538; 20571570 10.1038/nchem.657

[4b] S. T. Schneebeli , M. Frasconi , Z. Liu , Y. Wu , D. M. Gardner , N. L. Strutt , C. Cheng , R. Carmieli , M. R. Wasielewski , J. F. Stoddart , Angew. Chem. Int. Ed. 2013, 52, 13100–13104; 10.1002/anie.201307984 24227594

[4c] A. Vargas Jentzsch , A. Hennig , J. Mareda , S. Matile , Acc. Chem. Res. 2013, 46, 2791–2800; 23547885 10.1021/ar400014r

[4d] L. Adriaenssens , G. Gil-Ramirez , A. Frontera , D. Quinonero , E. C. Escudero-Adan , P. Ballester , J. Am. Chem. Soc. 2014, 136, 3208–3218; 24494711 10.1021/ja412098v

[4e] B. Jiang , W. Wang , Y. Zhang , Y. Lu , C. W. Zhang , G. Q. Yin , X. L. Zhao , L. Xu , H. Tan , X. Li , G. X. Jin , H. B. Yang , Angew. Chem. Int. Ed. 2017, 56, 14438–14442; 10.1002/anie.201707209 28961361

[4f] L. Li , Y. J. Hong , Y. Lin , W. C. Xiao , M. J. Lin , Chem. Commun. 2018, 54, 11941–11944; 10.1039/c8cc06522e 30288505

[4g] J. Luo , Y. F. Ao , Q. Q. Wang , D. X. Wang , Angew. Chem. Int. Ed. 2018, 57, 15827–15831; 10.1002/anie.201810836 30295403

[4h] A. O. Ortolan , I. Ostrom , G. F. Caramori , R. L. T. Parreira , E. H. da Silva , F. M. Bickelhaupt , J. Phys. Chem. A 2018, 122, 3328–3336; 29542924 10.1021/acs.jpca.8b01866

[4i] T. L. D. Tam , C. K. Ng , X. Lu , Z. L. Lim , J. Wu , Chem. Commun. 2018, 54, 7374–7377; 10.1039/c8cc03941k 29911219

[4j] J. Wang , X. Gu , H. Ma , Q. Peng , X. Huang , X. Zheng , S. H. P. Sung , G. Shan , J. W. Y. Lam , Z. Shuai , B. Z. Tang , Nat. Commun. 2018, 9, 2963; 30054473 10.1038/s41467-018-05298-y PMC6063922

[4k] D. I. Alexandropoulos , B. S. Dolinar , H. Xie , K. R. Vignesh , K. R. Dunbar , Chem. Commun. 2019, 55, 12356–12359; 10.1039/c9cc04151f 31560017

[4l] C. S. Anstöter , J. P. Rogers , J. R. R. Verlet , J. Am. Chem. Soc. 2019, 141, 6132–6135; 30938520 10.1021/jacs.9b01345

[4m] D. H. Tuo , W. Liu , X. Y. Wang , X. D. Wang , Y. F. Ao , Q. Q. Wang , Z. Y. Li , D. X. Wang , J. Am. Chem. Soc. 2019, 141, 1118–1125; 30562462 10.1021/jacs.8b12018

[4n] H. Zeng , P. Liu , G. Feng , F. Huang , J. Am. Chem. Soc. 2019, 141, 16501–16511; 31525999 10.1021/jacs.9b09582

[4o] S. Kepler , M. Zeller , S. V. Rosokha , J. Am. Chem. Soc. 2019, 141, 9338–9348; 31083908 10.1021/jacs.9b03277

[4p] D. X. Wang , M. X. Wang , Acc. Chem. Res. 2020, 53, 1364–1380. 32559061 10.1021/acs.accounts.0c00243

[5] Selected accounts and recent examples:

[5a] A. Robertazzi , F. Krull , E.-W. Knapp , P. Gamez , CrystEngComm 2011, 13, 3293–3300;

[5b] U. Mayerhöffer , F. Wurthner , Angew. Chem. Int. Ed. 2012, 51, 5615–5619; 10.1002/anie.201200897 22528197

[5c] H. T. Chifotides , I. D. Giles , K. R. Dunbar , J. Am. Chem. Soc. 2013, 135, 3039–3055; 23414294 10.1021/ja3082473

[5d] H. I. Althagbi , A. J. Edwards , B. K. Nicholson , D. A. Reason , G. C. Saunders , S. A. Sim , D. A. van der Heijden , Cryst. Growth Des. 2016, 16, 174–188;

[5e] A. Bauzá , T. J. Mooibroek , A. Frontera , CrystEngComm 2016, 18, 10–23;

[5f] X. Sun , L. Y. Ji , W. W. Chen , X. Guo , H. H. Wang , M. Lei , Q. Wang , Y. F. Li , J. Mater. Chem. A 2017, 5, 20720–20728;

[5g] J. Wang , X. Gu , P. Zhang , X. Huang , X. Zheng , M. Chen , H. Feng , R. T. K. Kwok , J. W. Y. Lam , B. Z. Tang , J. Am. Chem. Soc. 2017, 139, 16974–16979; 29083164 10.1021/jacs.7b10150

[5h] D. Dutta , S. Chetry , A. Gogoi , B. Choudhury , A. K. Guha , M. K. Bhattacharyya , Polyhedron 2018, 151, 381–393;

[5i] E. Escrivà , J.-V. Folgado , A. Sancho , R. Ortíz , L. Perelló , C. R. de Arellano , Polyhedron 2019, 171, 137–146;

[5j] L. Li , Y. J. Hong , D. Y. Chen , W. C. Xiao , M. J. Lin , Chem. Commun. 2019, 55, 2364–2367; 10.1039/c8cc09834d 30724917

[5k] P. Pal , K. Das , A. Hossain , R. M. Gomila , A. Frontera , S. Mukhopadhyay , New J. Chem. 2021, 45, 11689–11696;

[5l] M. Samaniyan , M. Mirzaei , R. M. Gomila , H. Eshtiagh-Hosseini , N. Lotfian , J. T. Mague , A. N. Pour , A. Frontera , Dalton Trans. 2021, 50, 1895–1900. 33475649 10.1039/d0dt03891a

[6] Selected examples:

[6a] C. Estarellas , A. Frontera , D. Quinonero , P. M. Deya , Angew. Chem. Int. Ed. 2011, 50, 415–418; 10.1002/anie.201005635 21132687

[6b] C. Estarellas , A. Frontera , D. Quinonero , P. M. Deya , Chem. Asian J. 2011, 6, 2316–2318; 21717578 10.1002/asia.201100285

[6c] J. P. Schwans , F. Sunden , J. K. Lassila , A. Gonzalez , Y. Tsai , D. Herschlag , Proc. Natl. Acad. Sci. USA 2013, 110, 11308–11313; 23798413 10.1073/pnas.1206710110 PMC3710852

[6d] S. Z. Borozan , M. V. Zlatovic , S. D. Stojanovic , J. Biol. Inorg. Chem. 2016, 21, 357–368; 26910415 10.1007/s00775-016-1346-y

[6e] M. Giese , M. Albrecht , K. Rissanen , Chem. Commun. 2016, 52, 1778–1795; 10.1039/c5cc09072e 26697947

[6f] K. Kapoor , M. R. Duff , A. Upadhyay , J. C. Bucci , A. M. Saxton , R. J. Hinde , E. E. Howell , J. Baudry , Biochemistry 2016, 55, 6056–6069; 27753291 10.1021/acs.biochem.6b00624

[6g] X. Lucas , A. Bauza , A. Frontera , D. Quinonero , Chem. Sci. 2016, 7, 1038–1050; 29899893 10.1039/c5sc01386k PMC5967298

[6h] M. S. Smith , E. E. K. Lawrence , W. M. Billings , K. S. Larsen , N. A. Becar , J. L. Price , ACS Chem. Biol. 2017, 12, 2535–2537; 28886246 10.1021/acschembio.7b00768

[6i] V. R. Ribić , S. D. Stojanović , M. V. Zlatović , Int. J. Biol. Macromol. 2018, 106, 559–568; 28811207 10.1016/j.ijbiomac.2017.08.050

[6j] R. W. Newberry , R. T. Raines , ACS Chem. Biol. 2019, 14, 1677–1686; 31243961 10.1021/acschembio.9b00339 PMC6995338

[6k] R. Esmaeeli , M. L. N. Pina , A. Frontera , A. Perez , A. Bauza , J. Chem. Theory Comput. 2021, 17, 6624–6633; 34586810 10.1021/acs.jctc.1c00756 PMC8515804

[6l] S. Khemaissa , S. Sagan , A. Walrant , Crystals 2021, 11, 1032.

[7] Y. Zhao , Y. Domoto , E. Orentas , C. Beuchat , D. Emery , J. Mareda , N. Sakai , S. Matile , Angew. Chem. Int. Ed. 2013, 52, 9940–9943; 10.1002/anie.201305356 23946201

[8] Selected reviews:

[8a] Y. Zhao , Y. Cotelle , L. Liu , J. Lopez-Andarias , A. B. Bornhof , M. Akamatsu , N. Sakai , S. Matile , Acc. Chem. Res. 2018, 51, 2255–2263; 30188692 10.1021/acs.accounts.8b00223

[8b] A. J. Neel , M. J. Hilton , M. S. Sigman , F. D. Toste , Nature 2017, 543, 637–646. 28358089 10.1038/nature21701 PMC5907483

[9] Selected examples:

[9a] T. Lu , S. E. Wheeler , Org. Lett. 2014, 16, 3268–3271; 24915527 10.1021/ol501283u

[9b] Y. Zhao , C. Beuchat , Y. Domoto , J. Gajewy , A. Wilson , J. Mareda , N. Sakai , S. Matile , J. Am. Chem. Soc. 2014, 136, 2101–2111; 24456523 10.1021/ja412290r

[9c] Y. Zhao , N. Sakai , S. Matile , Nat. Commun. 2014, 5, 3911; 24845120 10.1038/ncomms4911

[9d] Y. Zhao , S. Benz , N. Sakai , S. Matile , Chem. Sci. 2015, 6, 6219–6223; 30090238 10.1039/c5sc02563j PMC6054047

[9e] Y. Cotelle , S. Benz , A. J. Avestro , T. R. Ward , N. Sakai , S. Matile , Angew. Chem. Int. Ed. 2016, 55, 4275–4279; 10.1002/anie.201600831 26916316

[9f] L. Liu , Y. Cotelle , A. B. Bornhof , C. Besnard , N. Sakai , S. Matile , Angew. Chem. Int. Ed. 2017, 56, 13066–13069; 10.1002/anie.201707730 28884964

[10] Selected examples:

[10a] A. Berkessel , S. Das , D. Pekel , J. M. Neudorfl , Angew. Chem. Int. Ed. 2014, 53, 11660–11664; 10.1002/anie.201403778 25208746

[10b] A. Franconetti , L. Contreras-Bernal , S. Jatunov , M. Gomez-Guillen , M. Angulo , R. Prado-Gotor , F. Cabrera-Escribano , Phys. Chem. Chem. Phys. 2014, 16, 18442–18453; 25070226 10.1039/c4cp01977f

[10c] N. Luo , Y. F. Ao , D. X. Wang , Q. Q. Wang , Chem. Asian J. 2021, 16, 3599–3603. 34464026 10.1002/asia.202100920

[11a] Y. Zhao , Y. Cotelle , A. J. Avestro , N. Sakai , S. Matile , J. Am. Chem. Soc. 2015, 137, 11582–11585; 26347381 10.1021/jacs.5b07382

[11b] Y. Cotelle , V. Lebrun , N. Sakai , T. R. Ward , S. Matile , ACS Cent. Sci. 2016, 2, 388–393; 27413782 10.1021/acscentsci.6b00097 PMC4919773

[11c] L. Liu , Y. Cotelle , A. J. Avestro , N. Sakai , S. Matile , J. Am. Chem. Soc. 2016, 138, 7876–7879; 27327089 10.1021/jacs.6b04936

[11d] C. Wang , F. N. Miros , J. Mareda , N. Sakai , S. Matile , Angew. Chem. Int. Ed. 2016, 55, 14422–14426; 10.1002/anie.201608842 27739617

[11e] L. Liu , Y. Cotelle , J. Klehr , N. Sakai , T. R. Ward , S. Matile , Chem. Sci. 2017, 8, 3770–3774; 28580108 10.1039/c7sc00525c PMC5436548

[11f] A. T. Pham , S. Matile , Chem. Asian J. 2020, 15, 1562–1566; 32311232 10.1002/asia.202000309

[11g] N. Luo , Y. F. Ao , D. X. Wang , Q. Q. Wang , Angew. Chem. Int. Ed. 2021, 60, 20650–20655; 10.1002/anie.202106509 34050685

[12a] J. López-Andarias , A. Frontera , S. Matile , J. Am. Chem. Soc. 2017, 139, 13296–13299; 28902995 10.1021/jacs.7b08113

[12b] A. B. Bornhof , M. Vazquez-Nakagawa , L. Rodriguez-Perez , M. Angeles Herranz , N. Sakai , N. Martin , S. Matile , J. Lopez-Andarias , Angew. Chem. Int. Ed. 2019, 58, 16097–16100; 10.1002/anie.201909540 31550074

[13a] A. B. Bornhof , A. Bauza , A. Aster , M. Pupier , A. Frontera , E. Vauthey , N. Sakai , S. Matile , J. Am. Chem. Soc. 2018, 140, 4884–4892; 29608309 10.1021/jacs.8b00809

[13b] J. López-Andarias , A. Bauza , N. Sakai , A. Frontera , S. Matile , Angew. Chem. Int. Ed. 2018, 57, 10883–10887; 10.1002/anie.201804092 PMC6120490 29806724

[14] Selected reviews on the synthesis and applications of interlocked molecules:

[14a] J. E. Beves , B. A. Blight , C. J. Campbell , D. A. Leigh , R. T. McBurney , Angew. Chem. Int. Ed. 2011, 50, 9260–9327; 10.1002/anie.201007963 21928462

[14b] S. Erbas-Cakmak , D. A. Leigh , C. T. McTernan , A. L. Nussbaumer , Chem. Rev. 2015, 115, 10081–10206; 26346838 10.1021/acs.chemrev.5b00146 PMC4585175

[14c] M. Xue , Y. Yang , X. Chi , X. Yan , F. Huang , Chem. Rev. 2015, 115, 7398–7501; 25734835 10.1021/cr5005869

[14d] C. J. Bruns , J. F. Stoddart , The Nature of the Mechanical Bond: From Molecules to Machines, Wiley, Hoboken, 2016;

[14e] X. Hou , C. Ke , J. Fraser Stoddart , Chem. Soc. Rev. 2016, 45, 3766–3780; 27030885 10.1039/c6cs00055j

[14f] J. E. Lewis , M. Galli , S. M. Goldup , Chem. Commun. 2017, 53, 298–312; 10.1039/c6cc07377h 27819362

[14g] J. R. J. Maynard , S. M. Goldup , Chem 2020, 6, 1914–1932.

[15a] D. A. Leigh , V. Marcos , M. R. Wilson , ACS Catal. 2014, 4, 4490–4497;

[15b] C. Kwamen , J. Niemeyer , Chem. Eur. J. 2021, 27, 175–186; 32705740 10.1002/chem.202002876 PMC7821015

[15c] J. de Maria Perez , J. Puigcerver , T. Orlando , A. Pastor , M. A. P. Martins , M. Alajarin , A. Martinez-Cuezva , J. Berna , Org. Chem. Front. 2021, 8, 4202–4210;

[15d] A. W. Heard , J. Meijide Suarez , S. M. Goldup , Nat. Chem. Rev. 2022, 10.1038/s41570-021-00348-4.

[16] Selected examples:

[16a] V. Blanco , A. Carlone , K. D. Hanni , D. A. Leigh , B. Lewandowski , Angew. Chem. Int. Ed. 2012, 51, 5166–5169; 10.1002/anie.201201364 22504844

[16b] J. Beswick , V. Blanco , G. De Bo , D. A. Leigh , U. Lewandowska , B. Lewandowski , K. Mishiro , Chem. Sci. 2015, 6, 140–143; 28553462 10.1039/c4sc03279a PMC5424444

[16c] K. Eichstaedt , J. Jaramillo-Garcia , D. A. Leigh , V. Marcos , S. Pisano , T. A. Singleton , J. Am. Chem. Soc. 2017, 139, 9376–9381; 28627882 10.1021/jacs.7b04955

[16d] M. Dommaschk , J. Echavarren , D. A. Leigh , V. Marcos , T. A. Singleton , Angew. Chem. Int. Ed. 2019, 58, 14955–14958; 10.1002/anie.201908330 31454135

[17] Selected examples:

[17a] Y. J. Lee , K. S. Liu , C. C. Lai , Y. H. Liu , S. M. Peng , R. P. Cheng , S. H. Chiu , Chem. Eur. J. 2017, 23, 9756–9760; 28577323 10.1002/chem.201702525

[17b] R. Mitra , H. Zhu , S. Grimme , J. Niemeyer , Angew. Chem. Int. Ed. 2017, 56, 11456–11459; 10.1002/anie.201704647 28574220

[17c] J. Y. C. Lim , N. Yuntawattana , P. D. Beer , C. K. Williams , Angew. Chem. Int. Ed. 2019, 58, 6007–6011; 10.1002/anie.201901592 PMC6519244 30861303

[17d] A. Martinez-Cuezva , M. Marin-Luna , D. A. Alonso , D. Ros-Niguez , M. Alajarin , J. Berna , Org. Lett. 2019, 21, 5192–5196; 31247790 10.1021/acs.orglett.9b01791

[17e] N. Pairault , H. Zhu , D. Jansen , A. Huber , C. G. Daniliuc , S. Grimme , J. Niemeyer , Angew. Chem. Int. Ed. 2020, 59, 5102–5107; 10.1002/anie.201913781 PMC7154720 31793163

[18] Selected examples:

[18a] Y. Tachibana , N. Kihara , T. Takata , J. Am. Chem. Soc. 2004, 126, 3438–3439; 15025467 10.1021/ja039461l

[18b] S. Hoekman , M. O. Kitching , D. A. Leigh , M. Papmeyer , D. Roke , J. Am. Chem. Soc. 2015, 137, 7656–7659; 26061430 10.1021/jacs.5b04726

[18c] M. Galli , J. E. Lewis , S. M. Goldup , Angew. Chem. Int. Ed. 2015, 54, 13545–13549; 10.1002/anie.201505464 PMC4678423 26387887

[18d] F. C. Hsueh , C. Y. Tsai , C. C. Lai , Y. H. Liu , S. M. Peng , S. H. Chiu , Angew. Chem. Int. Ed. 2020, 59, 11278–11282; 10.1002/anie.202001398 32249512

[19a] A. Martinez-Cuezva , D. Bautista , M. Alajarin , J. Berna , Angew. Chem. Int. Ed. 2018, 57, 6563–6567; 10.1002/anie.201803187 29659140

[19b] F. Modicom , E. M. G. Jamieson , E. Rochette , S. M. Goldup , Angew. Chem. Int. Ed. 2019, 58, 3875–3879; 10.1002/anie.201813950 PMC6589916 30600892

[19c] A. Martinez-Cuezva , A. Pastor , M. Marin-Luna , C. Diaz-Marin , D. Bautista , M. Alajarin , J. Berna , Chem. Sci. 2021, 12, 747–756; 10.1039/d0sc05757f PMC8178992 34163808

[19d] B. Leforestier , M. R. Gyton , A. B. Chaplin , Angew. Chem. Int. Ed. 2020, 59, 23500–23504; 10.1002/anie.202009546 PMC7756736 32929831

[20a] E. M. G. Jamieson , F. Modicom , S. M. Goldup , Chem. Soc. Rev. 2018, 47, 5266–5311; 29796501 10.1039/c8cs00097b PMC6049620

[20b] N. Pairault , J. Niemeyer , Synlett 2018, 29, 689–698;

[20c] N. H. Evans , Chem. Eur. J. 2018, 24, 3101–3112;29053181

[20d] K. Nakazono , T. Takata , Symmetry 2020, 12, 144.

[21a] Y. Cakmak , S. Erbas-Cakmak , D. A. Leigh , J. Am. Chem. Soc. 2016, 138, 1749–1751; 26835978 10.1021/jacs.6b00303 PMC4805306

[21b] A. W. Heard , S. M. Goldup , Chem 2020, 6, 994–1006. 32309674 10.1016/j.chempr.2020.02.006 PMC7153771

[22] A similar π-stacking interaction was observed previously in a naphthalene imide rotaxane: M. Denis , L. Qin , P. Turner , K. A. Jolliffe , S. M. Goldup , Angew. Chem. Int. Ed. 2018, 57, 5315–5319; 10.1002/anie.201713105 PMC5947583 29393993

[23a] J. D. Crowley , S. M. Goldup , A. L. Lee , D. A. Leigh , R. T. McBurney , Chem. Soc. Rev. 2009, 38, 1530–1541; 19587949 10.1039/b804243h

[23b] M. Denis , S. M. Goldup , Nat. Chem. Rev. 2017, 1, 0061.

[24a] V. Aucagne , K. D. Hanni , D. A. Leigh , P. J. Lusby , D. B. Walker , J. Am. Chem. Soc. 2006, 128, 2186–2187; 16478152 10.1021/ja056903f

[24b] H. Lahlali , K. Jobe , M. Watkinson , S. M. Goldup , Angew. Chem. Int. Ed. 2011, 50, 4151–4155; 10.1002/anie.201100415 21462287

[24c] J. E. M. Lewis , R. J. Bordoli , M. Denis , C. J. Fletcher , M. Galli , E. A. Neal , E. M. Rochette , S. M. Goldup , Chem. Sci. 2016, 7, 3154–3161. 29997807 10.1039/c6sc00011h PMC6005271

[25] It has previously been shown that the mechanical bond can enhance the basicity of functional groups, see:

[25a] N. Kihara , Y. Tachibana , H. Kawasaki , T. Takata , Chem. Lett. 2000, 29, 506–507;

[25b] G. Ragazzon , C. Schafer , P. Franchi , S. Silvi , B. Colasson , M. Lucarini , A. Credi , Proc. Natl. Acad. Sci. USA 2018, 115, 9385–9390. 29255033 10.1073/pnas.1712783115 PMC6156658

[26] For examples see Ref. [24b] and:

[26a] J. E. Lewis , J. Winn , L. Cera , S. M. Goldup , J. Am. Chem. Soc. 2016, 138, 16329–16336; 27700073 10.1021/jacs.6b08958

[26b] J. E. M. Lewis , P. D. Beer , S. J. Loeb , S. M. Goldup , Chem. Soc. Rev. 2017, 46, 2577–2591; 28447678 10.1039/c7cs00199a

[26c] M. A. Jinks , A. de Juan , M. Denis , C. J. Fletcher , M. Galli , E. M. G. Jamieson , F. Modicom , Z. Zhang , S. M. Goldup , Angew. Chem. Int. Ed. 2018, 57, 14806–14810; 10.1002/anie.201808990 PMC6220991 30253008

[26d] A. de Juan , D. Lozano , A. W. Heard , M. A. Jinks , J. M. Suarez , G. J. Tizzard , S. M. Goldup , Nat. Chem. 2021, 10.1038/s41557-021-00825-9. PMC7612332 34845345

[27] Reactions are reported at 30 °C rather than under ambient conditions as the observed selectivity was temperature dependent. Although reactions conducted in parallel at rt (i.e. under the same ambient conditions) always reproduced the selectivity trend **13**>**4 b**>**4 a**>**4 c** and the activity trend **13**>**4 b**>**4 a**–**4 c**) the absolute selectivity values varied slightly on different days. When the reaction temperature was controlled, the results became consistent. See Supporting Information section S4.

[28] Selected examples:

[28a] S. Demeshko , S. Dechert , F. Meyer , J. Am. Chem. Soc. 2004, 126, 4508–4509; 15070355 10.1021/ja049458h

[28b] D. Quiñonero , A. Frontera , P. M. Deya , ChemPhysChem 2008, 9, 397–399; 18236488 10.1002/cphc.200700788

[28c] C. Biswas , M. G. B. Drew , D. Escudero , A. Frontera , A. Ghosh , Eur. J. Inorg. Chem. 2009, 2238–2246;

[28d] M. Giese , M. Albrecht , T. Repenko , J. Sackmann , A. Valkonen , K. Rissanen , Eur. J. Org. Chem. 2014, 2435–2442.

[29] TURBOMOLE V7.2 2017, a development of University of Karlsruhe and Forschungszentrum Karlsruhe GmbH, **1989**–**2007**, TURBOMOLE GmbH, since 2007; available from http://www.turbomole.com.

[30a] C. M. Cardona , W. Li , A. E. Kaifer , D. Stockdale , G. C. Bazan , Adv. Mater. 2011, 23, 2367–2371; 21462372 10.1002/adma.201004554

[30b] A. Bolag , J. Lopez-Andarias , S. Lascano , S. Soleimanpour , C. Atienza , N. Sakai , N. Martin , S. Matile , Angew. Chem. Int. Ed. 2014, 53, 4890–4895; 10.1002/anie.201402042 24692344

[31] Z. Yuan , J. Li , Y. Xiao , Z. Li , X. Qian , J. Org. Chem. 2010, 75, 3007–3016. 20364870 10.1021/jo100231j

[32] X. Zhang , L. Liu , J. López-Andarias , C. Wang , N. Sakai , S. Matile , Helv. Chim. Acta 2018, 101, e1700288.

