# Supplementary material for: Anion–π Catalysis Enabled by the Mechanical Bond
Source: Angew Chem Int Ed Engl. 2022 Feb 3;61(12):e202115961. doi: 10.1002/anie.202115961 (PMC9303940; doi:10.1002/anie.202115961)
Supplement: Supplementary file 2 — Supporting Information [file ANIE-61-0-s003.pdf]

Angewandte Chemie
Eine Zeitschrift der Gesellschaft Deutscher Chemiker

Supporting Information

Anion–π Catalysis Enabled by the Mechanical Bond

J. R. J. Maynard, B. Galmés, A. D. Stergiou, M. D. Symes, A. Frontera, S. M. Goldup**

Contents

S1. GENERAL EXPERIMENTAL INFORMATION 3

S2. PROCEDURES AND CHARACTERISATION DATA FOR NOVEL COMPOUNDS REPORTED 5

S2.1. ROTAXANES 4 AND 6 AND RELATED COMPOUNDS (SCHEME 1) 5
S2.2. ROTAXANE 13 AND RELATED COMPOUNDS 28

S3. X-RAY ANALYSIS OF 4A AND $4AH^{+}$ 35

S3.1. SINGLE CRYSTAL X-RAY CRYSTALLOGRAPHIC DATA FOR 4A 35
S3.2. SINGLE CRYSTAL X-RAY CRYSTALLOGRAPHIC DATA FOR $4AH^{+}$ 36

S4. CATALYTIC EXPERIMENTS 37

S5. COMPARISON BETWEEN 4B, 13 AND PREVIOUSLY REPORTED ANION-Π CATALYSTS 39

S6. NMR ANALYSIS OF ROTAXANES 4 AND THEIR RELATIVE BASICITY 40

S7. COMPUTATIONAL RESULTS 42

S7.1 THEORETICAL METHODS 42
S7.2 COMPLEMENTARY RESULTS AND DISCUSSION 42
S7.3 PKA AND MEP CALCULATIONS 49

S8. ELECTROCHEMISTRY RESULTS 50

S9. OTHER ANION-Π CATALYSED REACTIONS INVESTIGATED WITH ROTAXANE 13 53

S10. CARTESIAN COORDINATES OF MODELLED STRUCTURES 56

S10.1. NEUTRAL CATALYST STRUCTURES 56
S10.2. PROTONATED CATALYST STRUCTURES 70
S10.3. CATALYST-SUBSTRATE COMPLEXES 85

S11. REFERENCES 113

S1. General Experimental Information

Unless otherwise stated, all reagents were purchased from commercial sources (Acros Organics, Alfa Aesar, Fisher Scientific, FluoroChem, Sigma Aldrich and VWR) and used without further purification. $[Cu(MeCN)_4]PF_6$ was prepared as described by Pigorsch and Köckerling.[1] Anhydrous solvents were purchased from Acros Organics. Petrol refers to the fraction of petroleum ether boiling in the range 40-60 °C. IPA refers to isopropanol. THF refers to tetrahydrofuran. EDTA-NH_3 solution refers to an aqueous solution of NH_3 (17% w/w) saturated with sodium-ethylenediaminetetraacetate. $CDCl_3$ (without stabilising agent) was distilled over $CaCl_2$ and K_2CO_3 prior to use. Unless otherwise stated, all reaction mixtures were performed in oven dried glassware under an inert N_2 atmosphere with purchased anhydrous solvents. Unless otherwise stated experiments carried out in sealed vessels were performed in CEM microwave vials, with crimped aluminium caps, with PTFE septa. Young's tap vessels and Schlenk techniques were used where specified.

Flash column chromatography was performed using Biotage Isolera-4 or Isolera-1 automated chromatography system. SiO_2 cartridges were purchased commercially Biotage (SNAP or ZIP (50 µm), or Sfär (60 µm) irregular silica, default flow rates). Neutralised SiO_2 refers to ZIP cartridges which were eluted with petrol-NEt_3 (99 : 1, 5 column volumes), followed by petrol (5 column volumes). Analytical TLC was performed on pre-coated silica gel plates on aluminum (0.25 mm thick, 60F254, Merck, Germany) and observed under UV light (254 nm) or visualised with $KMnO_4$ stain.

All melting points were determined using a Griffin apparatus. NMR spectra were recorded on Bruker AV400 or AV500 instrument, at a constant temperature of 298 K. Chemical shifts are reported in parts per million from low to high field and referenced to residual solvent. Coupling constants (*J*) are reported in Hertz (Hz). Standard abbreviations indicating multiplicity were used as follows: m = multiplet, quint = quintet, q = quartet, t = triplet, d = doublet, s = singlet, app. = apparent, br = broad, sept = septet. Signal assignment was carried out using 2D NMR methods (COSY, NOESY, TOCSY, HSQC, HMBC or ^{31}P-^{1}H HMBC) where necessary. In some cases, complex multiplets with multiple contributing proton signals, exact assignment was not possible. In interlocked compounds, all proton signals corresponding to axle components are in lower case, and all proton signals corresponding to the macrocycle components are in upper case.

Low resolution mass spectrometry was carried out by the mass spectrometry services at University of Southampton (Waters TQD mass spectrometer equipped with a triple quadrupole analyser with UHPLC injection [BEH C18 column; MeCN-H_2O gradient {0.2% formic acid}]). High resolution mass spectrometry was carried out either by the mass spectrometry service at the University of Edinburgh (ThermoElectron MAT 900) or by the mass spectrometry services at the University of Southampton (MaXis, Bruker Daltonics, with a Time of Flight (TOF) analyser; samples were introduced to the mass spectrometer via a Dionex Ultimate 3000 autosampler and uHPLC pump in a gradient of 20% MeCN in hexane to 100% acetonitrile (0.2% formic acid) over 5-10 min at 0.6 mL/min; column: Acquity UPLC BEH C18 (Waters) 1.7 micron 50 × 2.1mm).

Electrochemical studies were performed in a three-electrode configuration using CH Instruments CHI600D potentiostat. A glassy carbon button electrode was used as the working electrode, a graphite rod was used as the counter electrode and the reference electrode was Ag/$AgNO_3$. The glassy carbon working electrode was polished

using polishing powder and then washed with acetone and deionized water prior to use. Graphite rod electrodes were not re-used.

The following compounds were synthesized according to literature procedures: 1,[2] **3**,[3] **S1**,[3] **S2**,[3] **S3**,[4] **7**[5] and **S5**.[6]

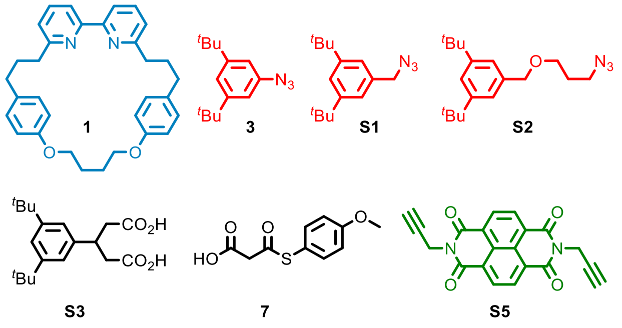

S2. PROCEDURES AND CHARACTERISATION DATA FOR NOVEL COMPOUNDS REPORTED

Rotaxanes 4 and 6 and related compounds (Scheme 1)

NDI half axle 2

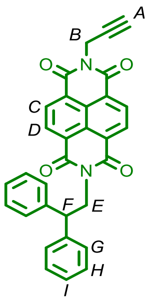

CEM vial was charged with 1,4,5,8-naphthaelentetracarboxylic acid dianhydride (500 mg, 1.86 mmol, 1 eq). DMF (10 mL) was then added, followed by propargyl amine (120 µL, 1.86 mmol, 1 eq.). Following sonication, the reaction mixture was heated for 5 minutes at 75 °C then 15 minutes at 140 °C. After cooling solvents were removed *in vacuo* and the crude mixture was suspended in acetone then poured onto 1 M HCl. The precipitated solids were isolated by filtration, washed with 1 M HCl and dried. The isolated solids were transferred to a CEM vial with 2,2-diphenylethylamine (367 mg, 1.86 mmol, 1 eq). DMF (10 mL) was added, followed by Et_3N (187 µL, 1.86 mmol, 1 eq) and the suspension was sonicated. The reaction mixture was then heated for 5 minutes at 75 °C and 15 minutes at 140 °C. After cooling, the solvents were removed *in vacuo* and the resulting solids were suspended in acetone and poured onto 1 M HCl. The precipitated solids were isolated by filtration and dried. The product was purified by flash chromatography (SiO_2, 1:1 petrol/CH_2Cl_2 with 10% Et_2O) to give propargyl NDI **2** as a yellow solid (360 mg, 0.74 mmol, 40%). ^{1}H NMR (400 MHz, $CDCl_3$, 298 K) δ 8.76 (d, *J*=7.6, 1H, H_D), 8.67 (d, *J*=7.6, 1H, H_E), 7.34 (d, *J*=7.2, 4H, H_H), 7.27 – 7.20 (m, 4H, H_I), 7.15 (t, *J*=7.3, 2H, H_J), 4.96 (d, *J*=2.6, 2H, H_C), 4.88 (d, *J*=7.8, 2H, H_F), 4.78 (t, *J*=7.8, 1H, H_G), 2.22 (t, *J*=2.4, 1H, H_A). ^{13}C NMR (100 MHz, $CDCl_3$) δ 162.8, 162.2, 141.4, 131.4, 131.1, 128.6, 128.6, 127.0, 126.8, 126.4, 78.0, 71.3, 48.8, 45.0, 30.0. MP: 265 - 268 °C. HR-ESI-MS (+ve) m/z = 507.1317 $[M+Na]^+$ (calc. m/z for $C_{31}H_{20}N_2NaO_4$ 507.1315).

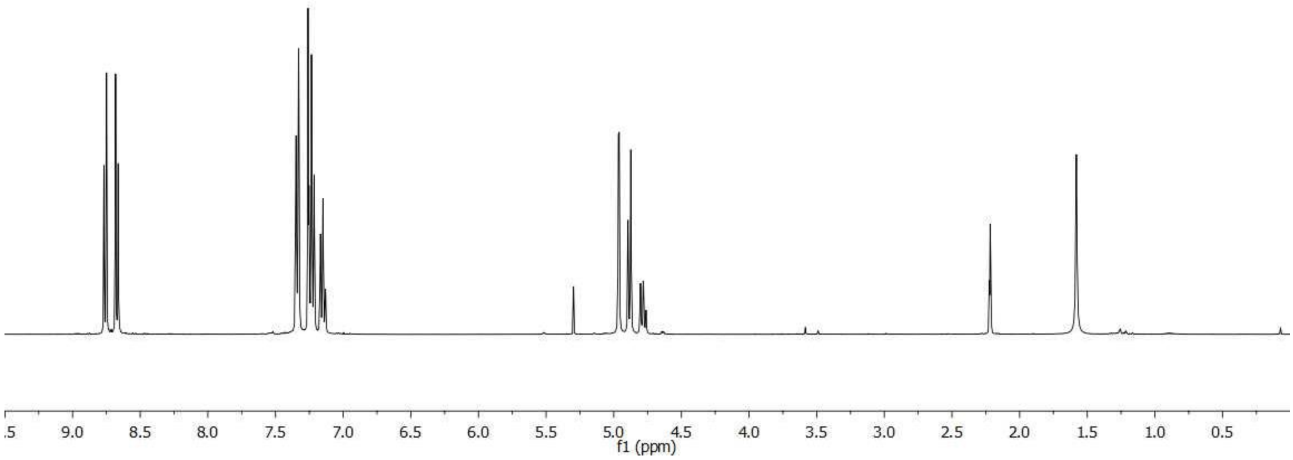

Figure S1 - ^{1}H NMR (400 MHz, $CDCl_3$, 298 K) of **2.**

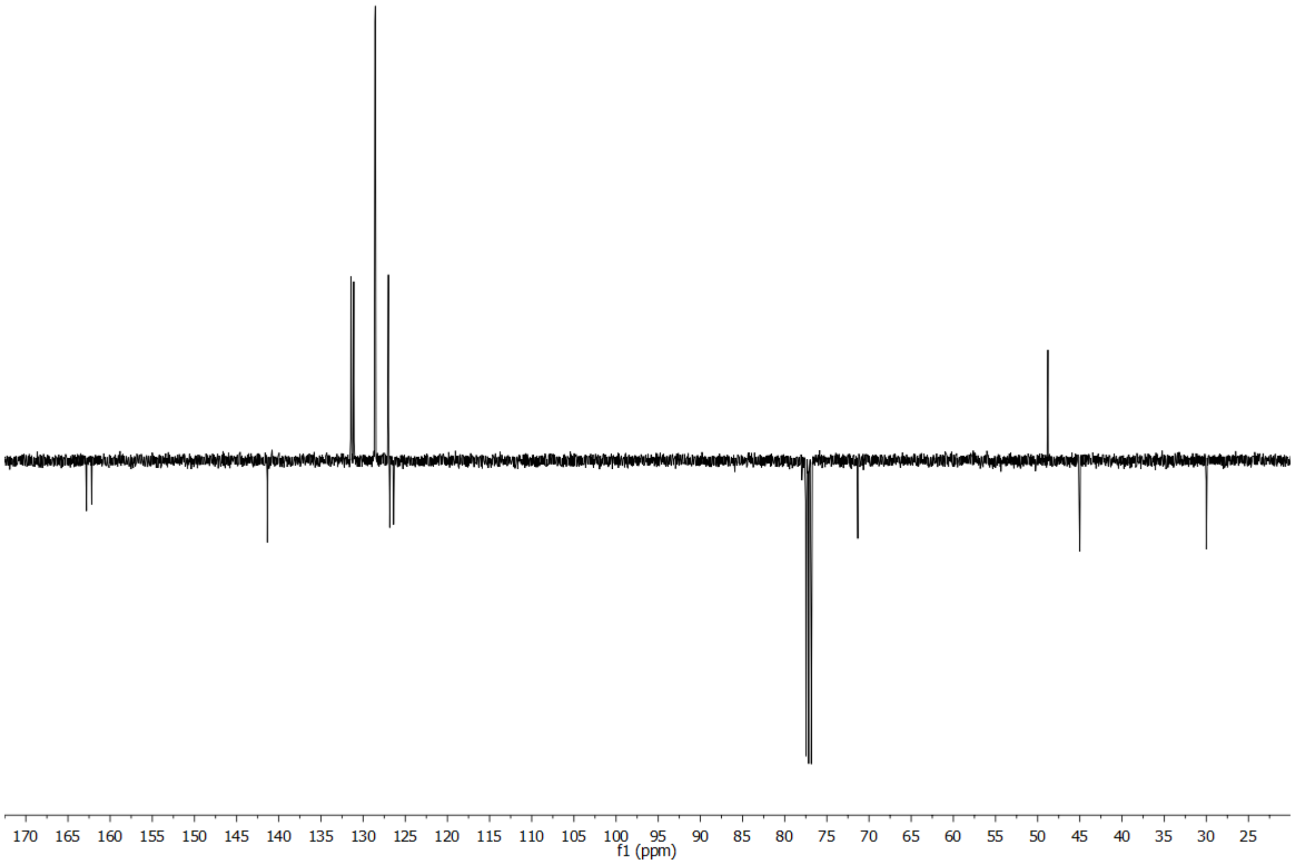

Figure S2 - ^{13}C-JMOD NMR (100 MHz, $CDCl_3$, 298 K) of **2.**

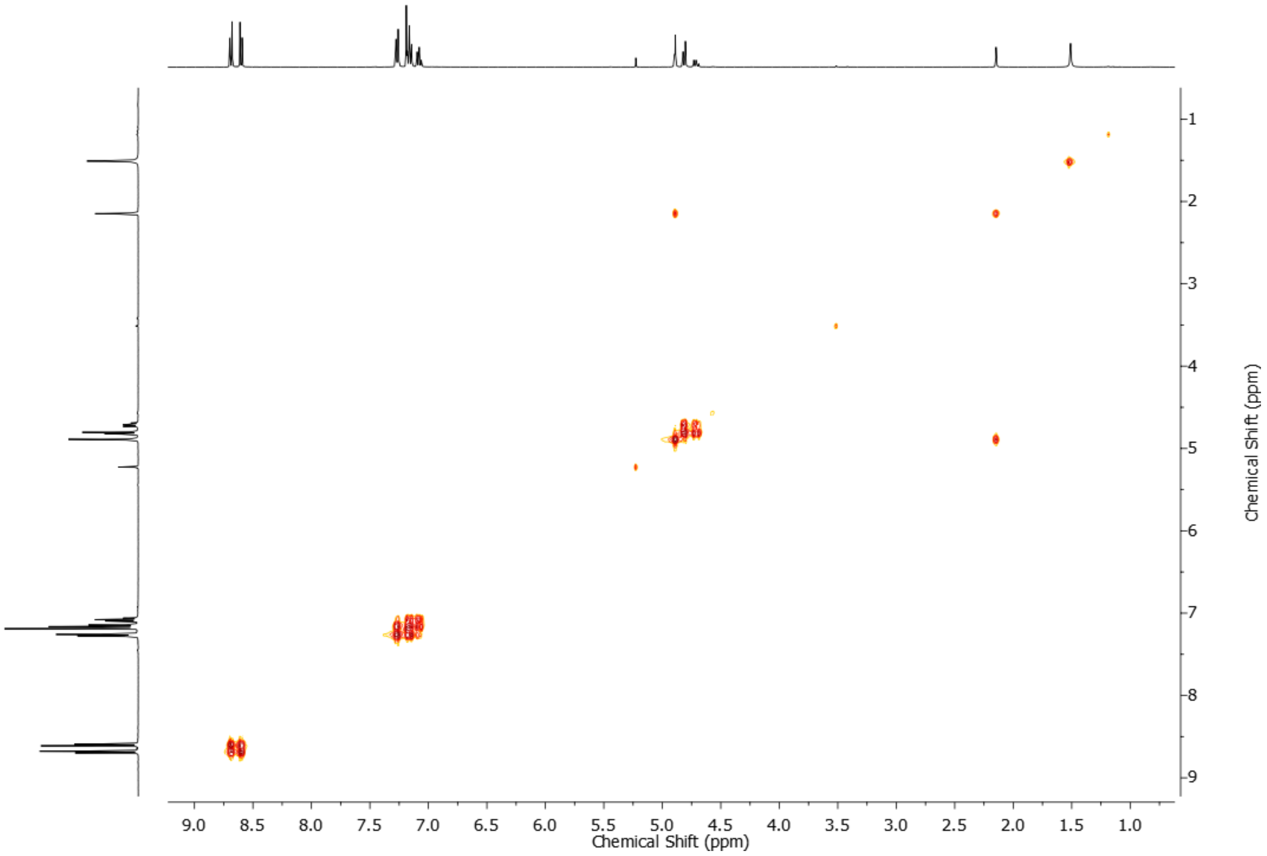

Figure S3 - ^{1}H COSY NMR (400 MHz, $CDCl_3$, 298 K) of **2**.

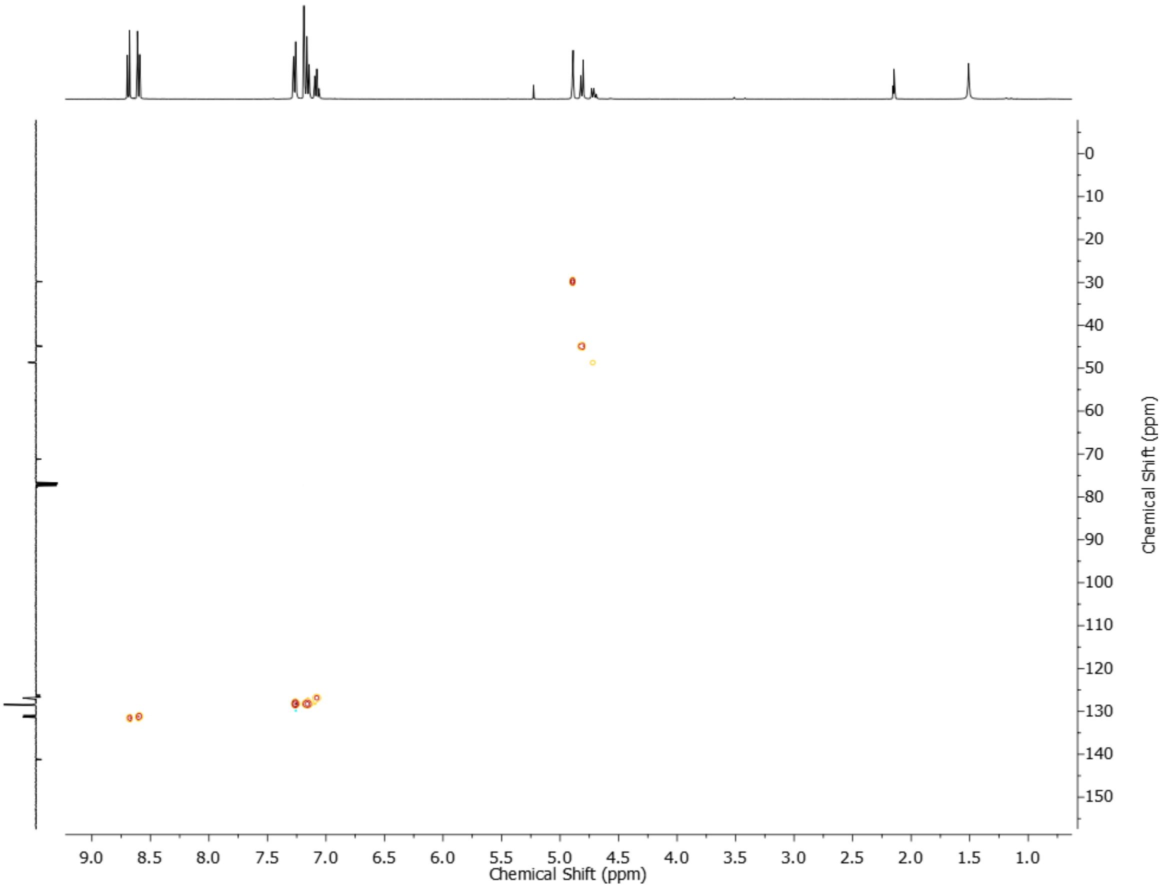

Figure S4 - ^{1}H-^{13}C HSQC NMR (400 MHz, $CDCl_3$, 298 K) of **2**.

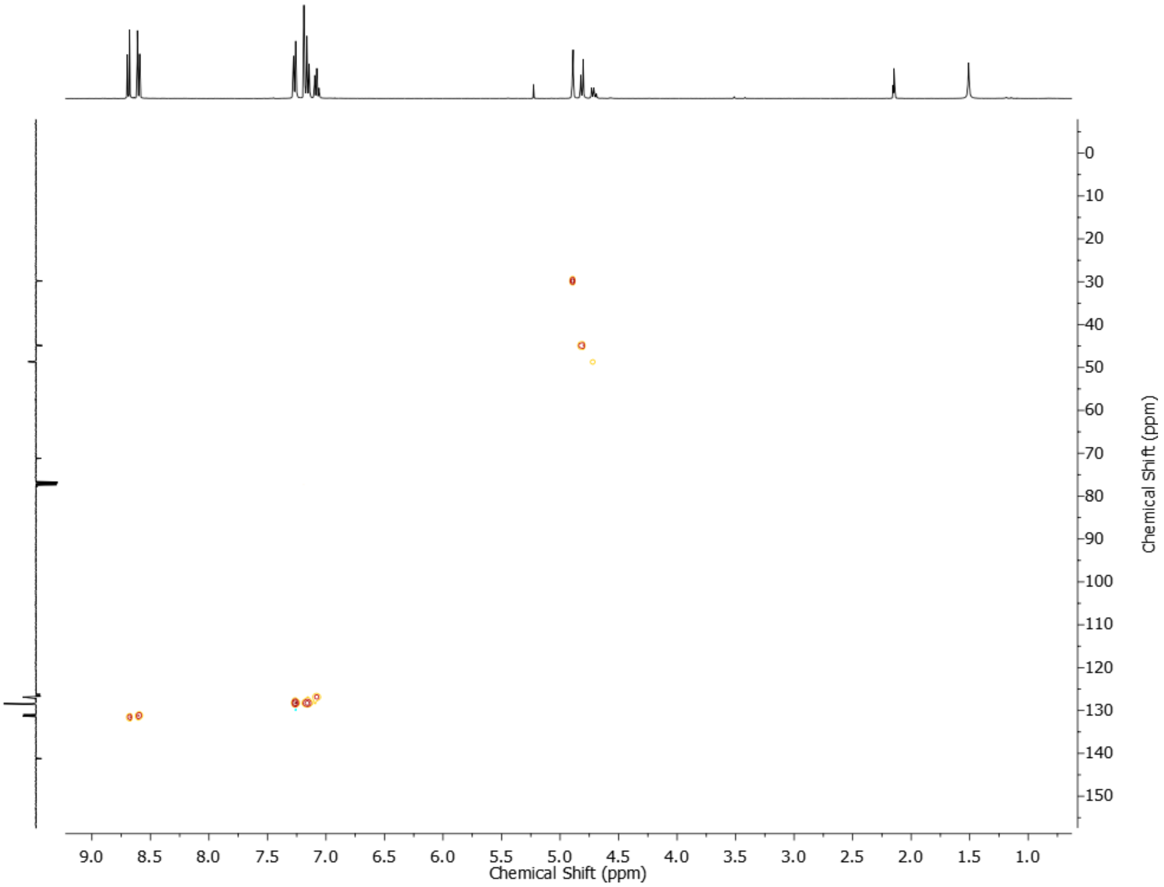

Figure S5 - ^{1}H-^{13}C HMBC NMR (400 MHz, $CDCl_3$, 298 K) of **2**.

Rotaxane 4a

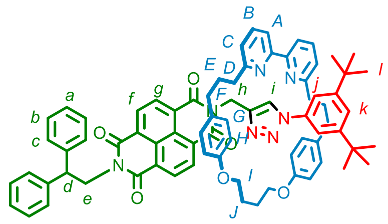

In a CEM vial, propargyl NDI **2** (55 mg, 0.113 mmol, 1.1 eq), bipyridine macrocycle **1** (49 mg, 0.103 mmol, 1.0 eq), azide **3** (29 mg, 0.124 mmol, 1.2 eq) and $[Cu(MeCN)_4]PF_6$ (37 mg, 0.099 mmol, 0.96 eq.) were dissolved in CH_2Cl_2 (2.6 mL). DIPEA (55 µ, 0.21 mmol, 3.0 eq.) was added and the reaction was left for 16 h, after which TFA (16 µL, 0.206 mmol, 2.0 eq.) was added. After 3 h, the reaction mixture was diluted with CH_2Cl_2 (10 mL) and washed with a saturated EDTA/NH_3 solution (5 mL), water (5 mL) and brine (5 mL). The organic layers were dried with $MgSO_4$, filtered and concentrated. Column chromatography (0 to 50% MeCN in CH_2Cl_2) gave **4a** as a yellow foam (115 mg, 0.096 mmol, 93%). ^{1}H NMR (400 MHz, $CDCl_3$) δ 10.18 (s, 1H, H_i), 8.32 (d, J = 7.5, 2H, H_g), 8.05 (d, J = 7.5, 2H, H_f), 7.48 (d, J = 7.6, 4H, H_c), 7.42 (d, J = 1.7, 2H, H_j), 7.34 (t, J = 7.6, 4H, H_b), 7.23 (t, J = 7.4, 2H, H_a), 7.06 (t, J = 1.7, 1H, H_k), 6.95 (t, J = 7.7, 2H, H_B), 6.78 (d, J = 7.7, 2H, H_A), 6.56 (d, J = 1.5, 8H, H_G, H_H), 6.31 (d, J = 7.7, 2H, H_C), 5.48 (s, 2H, H_h), 5.02 – 4.88 (m, 3H, H_d, H_e), 4.80 (q, J = 7.7, 2H, H_I), 4.27 – 4.20 (m, 2H, H_I), 2.54 – 2.35 (m, 6H, H_F, H_J), 2.21 – 1.97 (m, 6H, H_D, H_J), 1.82 – 1.70 (m, 2H, H_E), 1.50 – 1.38 (m, 2H, H_E), 0.99 (s, 18H, H_l). ^{13}C NMR (100 MHz, $CDCl_3$) δ 163.8, 163.1, 161.8, 157.4, 154.5, 150.6, 141.9, 141.4, 136.8, 136.2, 131.8, 130.6, 130.0, 128.8, 128.6, 128.3, 127.2, 126.5, 126.2, 126.1, 125.6, 122.1, 120.6, 117.5, 115.0, 114.0, 66.3, 49.0, 44.7, 37.1, 36.9, 35.7, 35.0, 31.3, 31.2, 25.0. LR-MS (ESI) m/z = 1194.9 $[M+H]^+$.

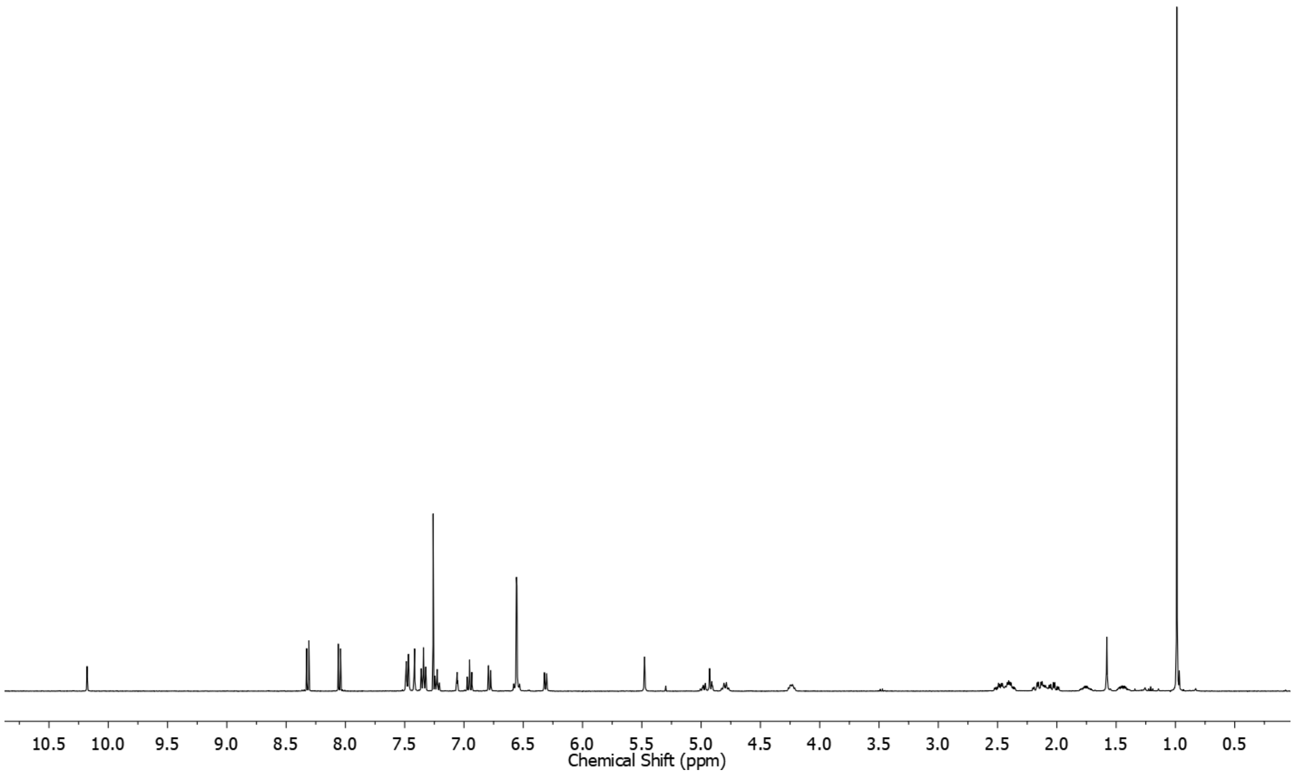

Figure S6 - ^{1}H NMR (400 MHz, $CDCl_3$, 298 K) of **4a.**

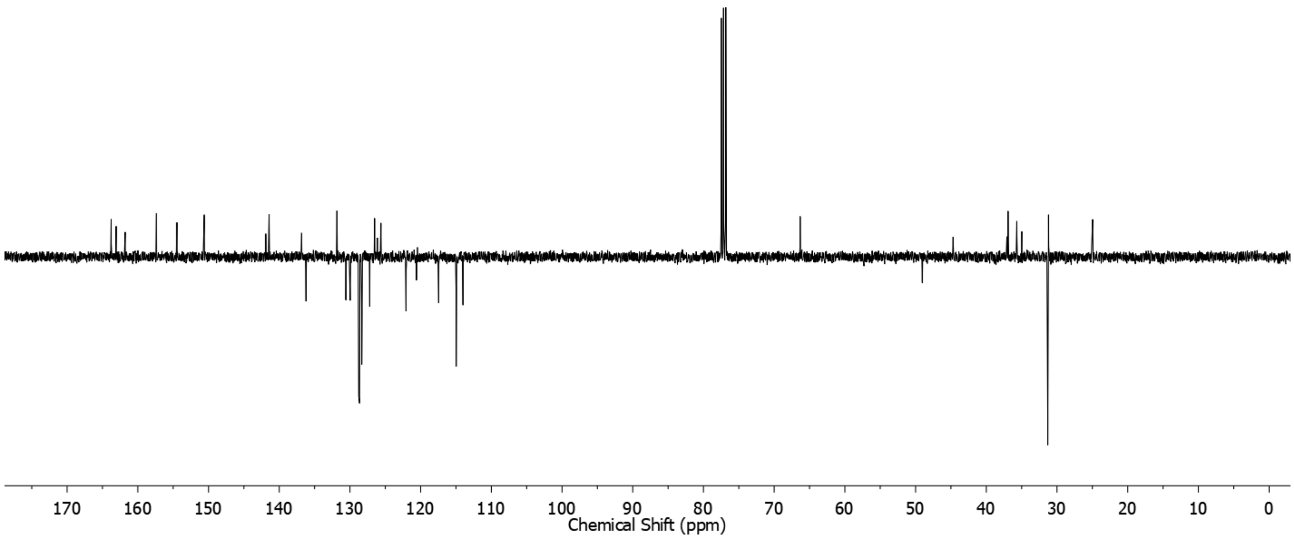

Figure S7 - ^{13}C-JMOD NMR (100 MHz, $CDCl_3$, 298 K) of **4a.**

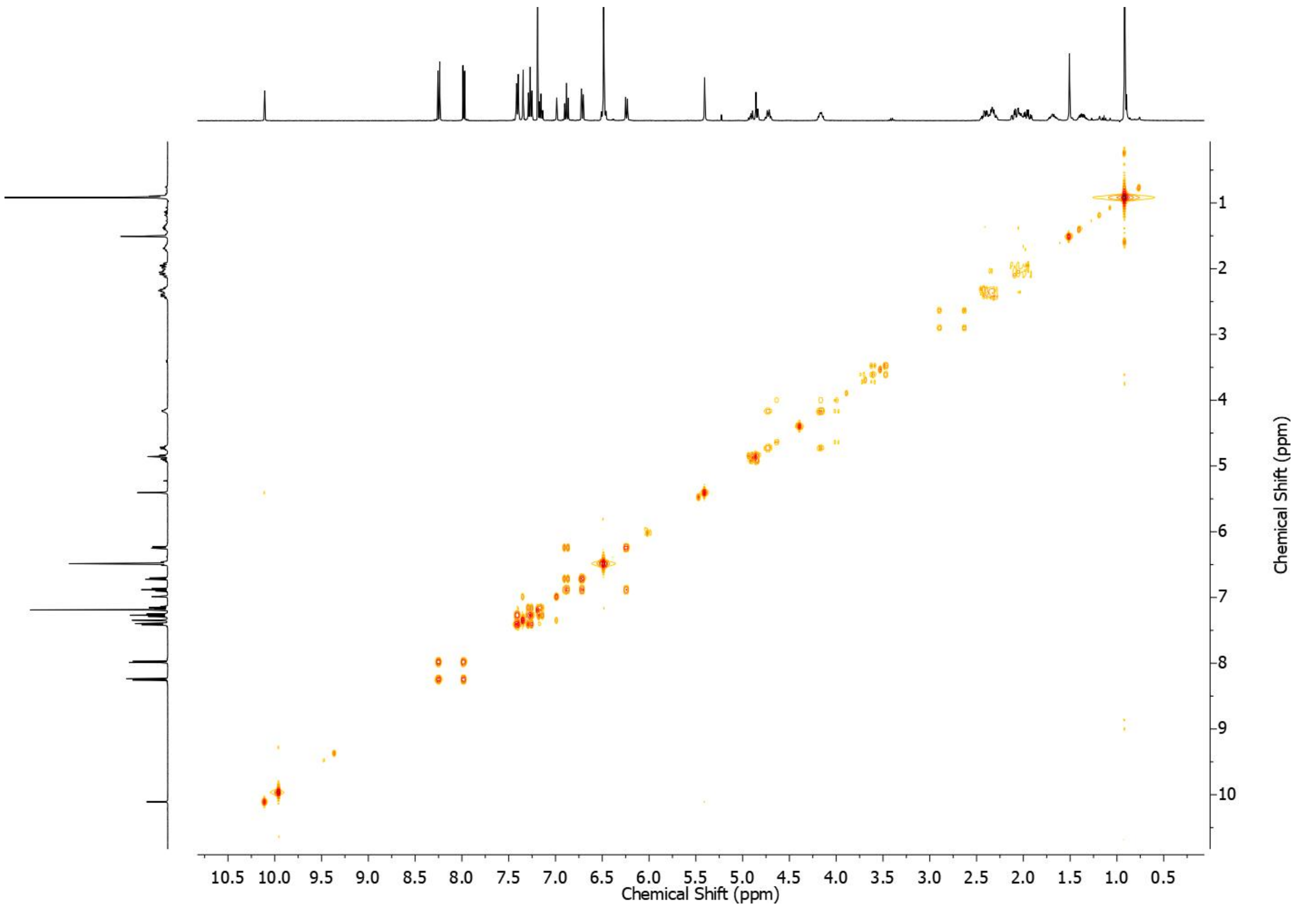

Figure S8 - ^{1}H COSY NMR (400 MHz, $CDCl_3$, 298 K) of **4a**.

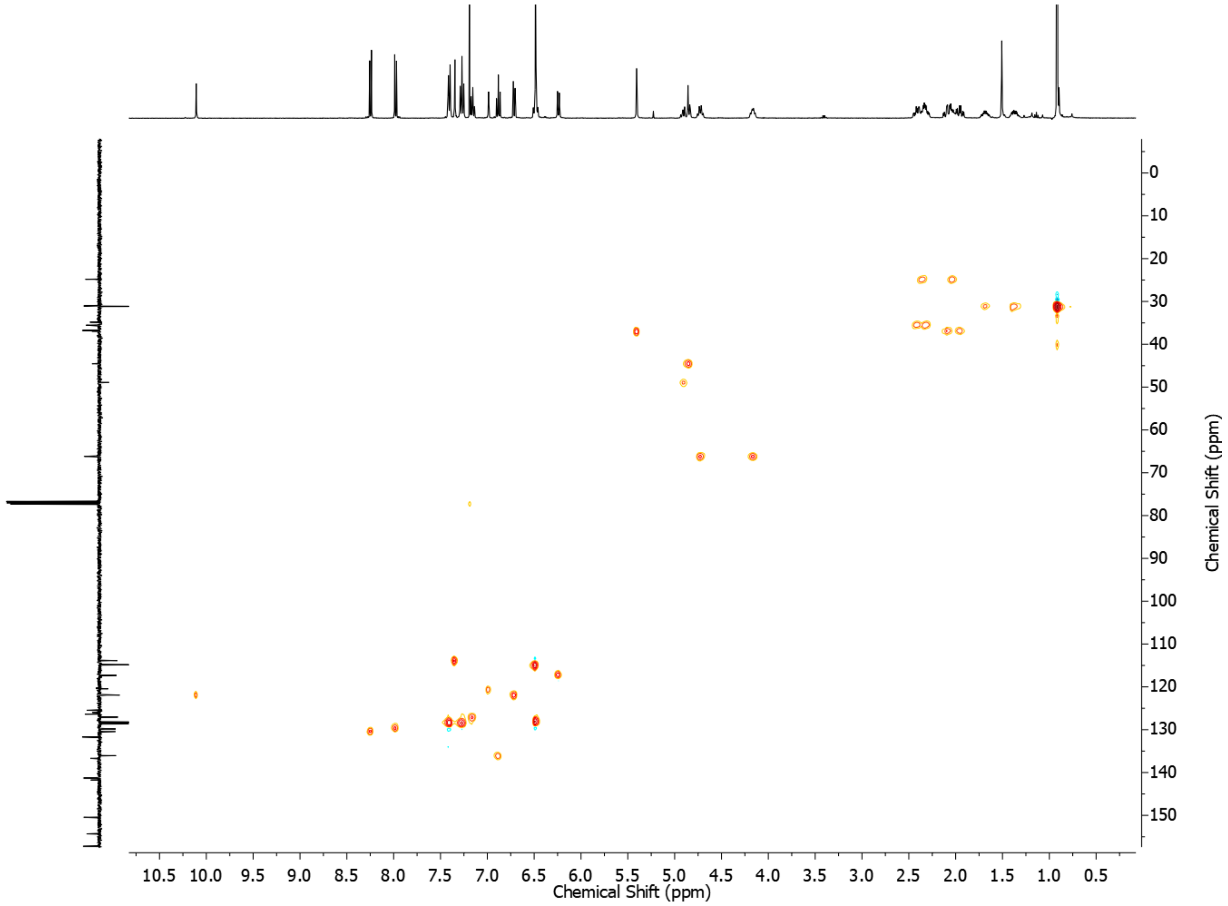

Figure S9 - ^{1}H-^{13}C HSQC NMR (400 MHz, $CDCl_3$, 298 K) of **4a**.

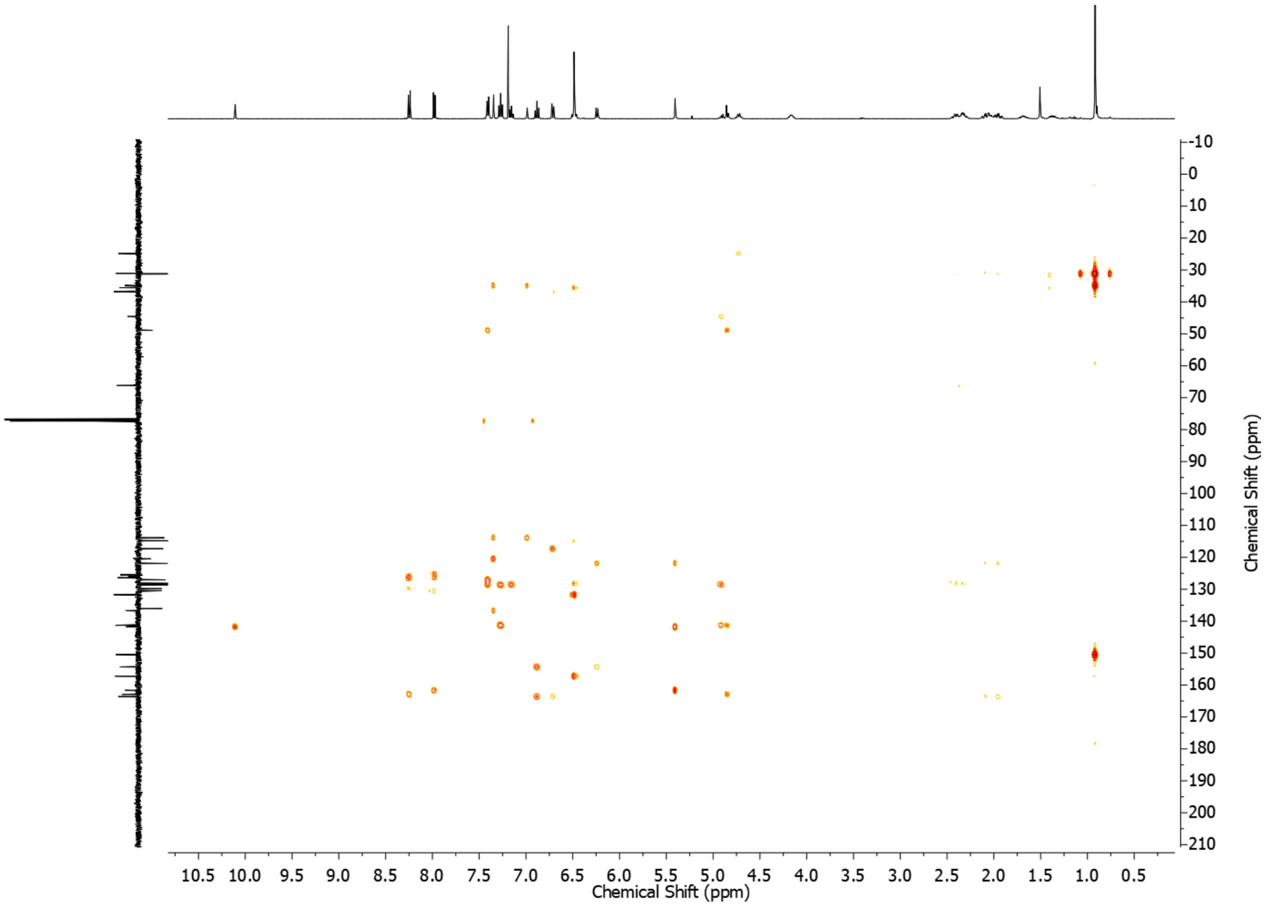

Figure S10 - ^{1}H-^{13}C HMBC NMR (400 MHz, $CDCl_3$, 298 K) of **4a**.

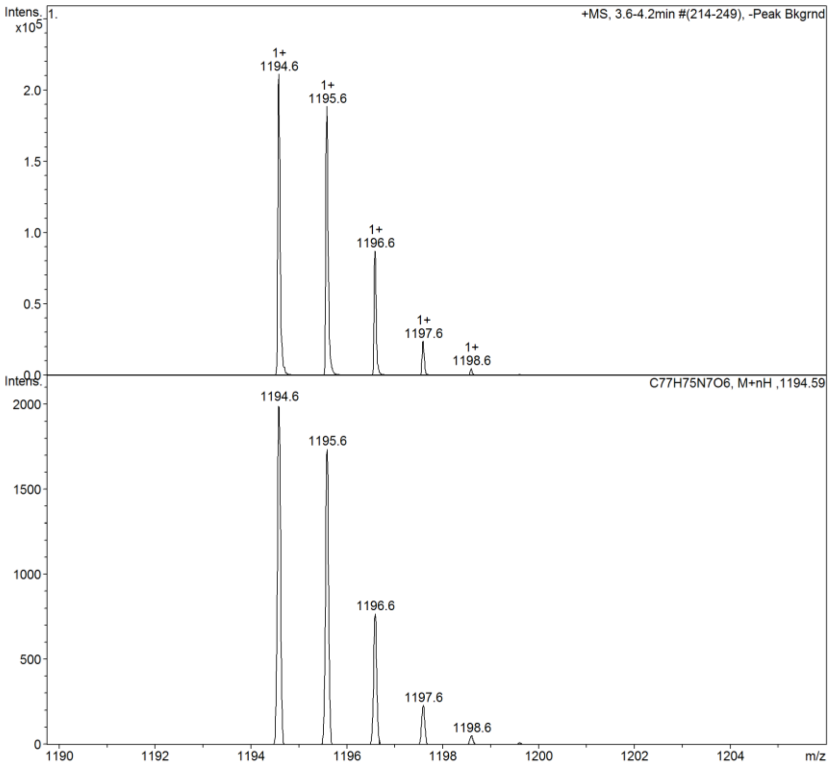

Figure S11 - Observed (top) and calculated (bottom) isotopic patterns for **4a**.

Rotaxane 4b

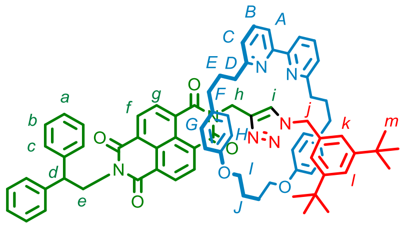

In a CEM vial, propargyl NDI **2** (96 mg, 0.20 mmol, 1.1 eq), bipyridine macrocycle **1** (86 mg, 0.18 mmol, 1.0 eq), azide **S1** (48.5 mg,0.2 mmol, 1.1 eq) and $[Cu(MeCN)_4]PF_6$ (64 mg, 0.17 mmol, 0.95 eq) were dissolved in CH_2Cl_2 (4.5 mL). DIPEA (75 µL, 0.54 mmol, 3.0 eq) was added and the reaction was left for 16 h, after which TFA (28 µL, 0.36 mmol, 2.0 eq) was added. After 3 h, the reaction mixture was diluted with CH_2Cl_2 and washed with a saturated EDTA/NH_3 solution, water and brine. The organic layers were dried with $MgSO_4$, filtered and concentrated. Column chromatography (0 to 50% MeCN in CH_2Cl_2) gave **4b** as a yellow foam (169 mg, 0.14 mmol, 75%).^{1}H NMR (400 MHz, $CDCl_3$) δ 9.41 (s, 1H, H_i), 8.38 (d, J = 7.6, 2H, H_g), 8.18 (d, J = 7.6, 2H, H_f), 7.47 – 7.40 (m, 4H, H_c), 7.34 – 7.27 (m, 5H, H_b, H_l), 7.23 – 7.11 (m, 4H, H_a, H_B), 6.94 (d, J = 1.8, 2H, H_k), 6.87 (d, J = 7.8, 2H, H_A), 6.68 (d, J = 7.8, 2H, H_C), 6.59 (d, J = 2.2, 8H, H_G, H_H), 5.02 (s, 2H, H_h), 4.96 – 4.87 (m, 3H, H_d, H_e), 4.50 – 4.40 (m, 4H, H_j, H_I), 4.36 – 4.28 (m, 2H, H_I), 2.63 – 2.54 (m, 2H, H_F), 2.45 – 2.36 (m, 2H, H_F), 2.26 – 2.02 (m, 8H, H_J, H_D), 1.85 – 1.73 (m, 2H, H_E), 1.71 – 1.55 (m, 2H, H_E), 1.14 (s, 18H, H_m). ^{13}C NMR (100 MHz, $CDCl_3$) δ 163.0, 162.9, 161.6, 157.5, 155.6, 150.5, 141.3, 140.4, 136.2, 133.8, 131.8, 130.5, 129.9, 128.7, 128.6, 128.5, 127.0, 126.7, 126.1, 126.1, 125.5, 124.3, 123.0, 121.1, 118.3, 114.9, 66.5, 53.6, 48.9, 44.6, 36.5, 36.3, 35.0, 34.6, 31.3, 30.4, 25.0. LR-MS (ESI) m/z = 1208.6 [M+H]+.

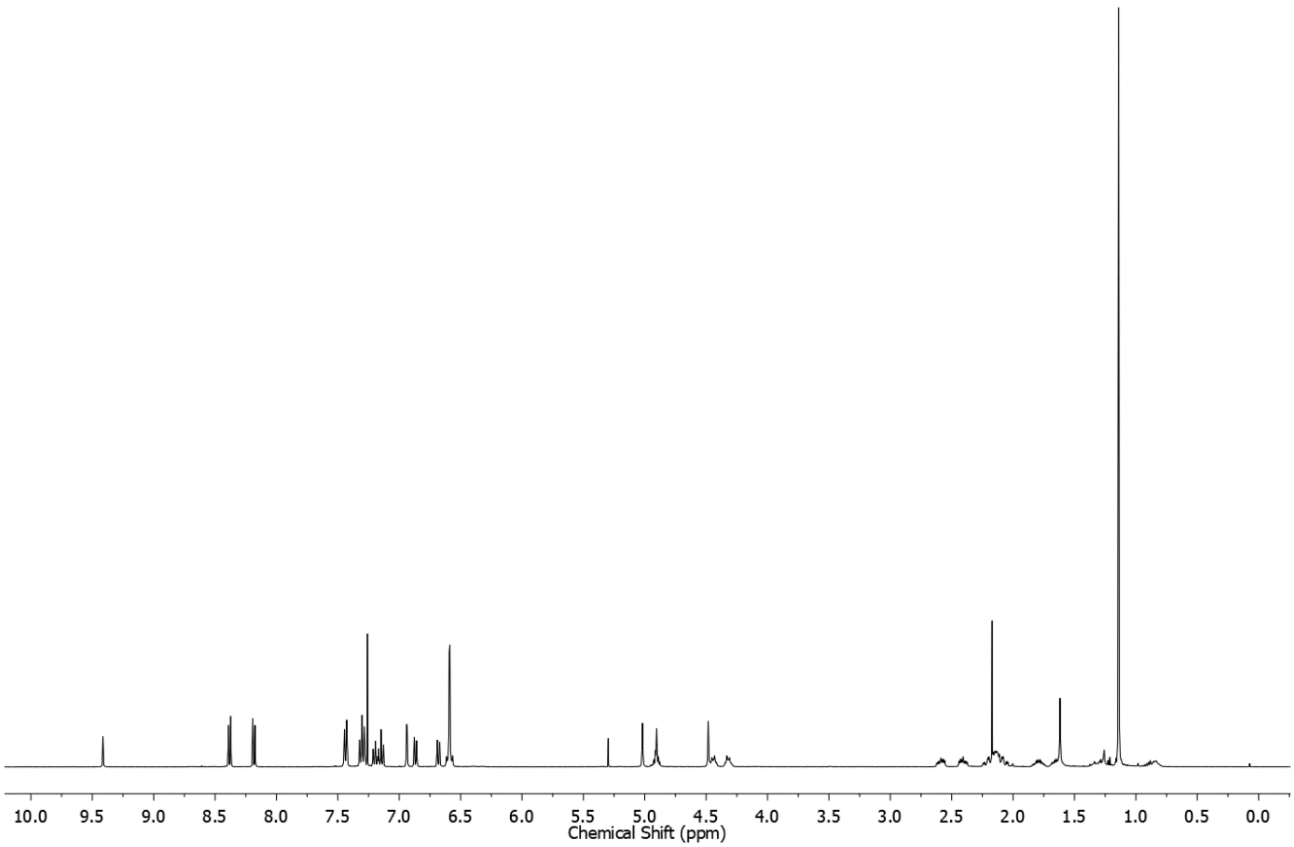

Figure S12 - ^{1}H NMR (400 MHz, $CDCl_3$, 298 K) of **4b.**

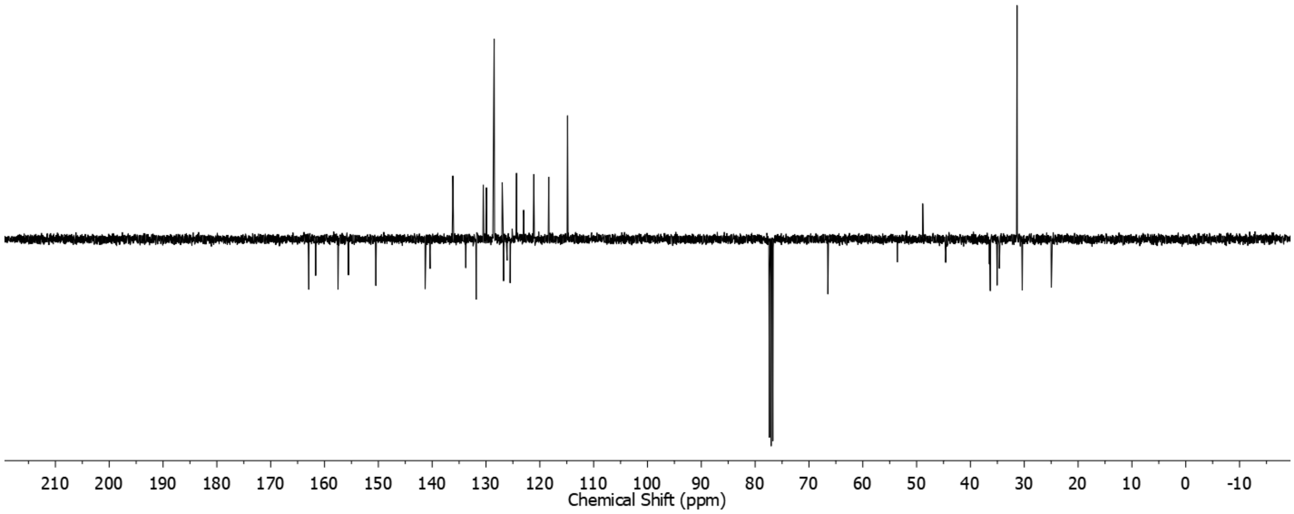

Figure S13 - ^{13}C-JMOD NMR (100 MHz, $CDCl_3$, 298 K) of **4b.**

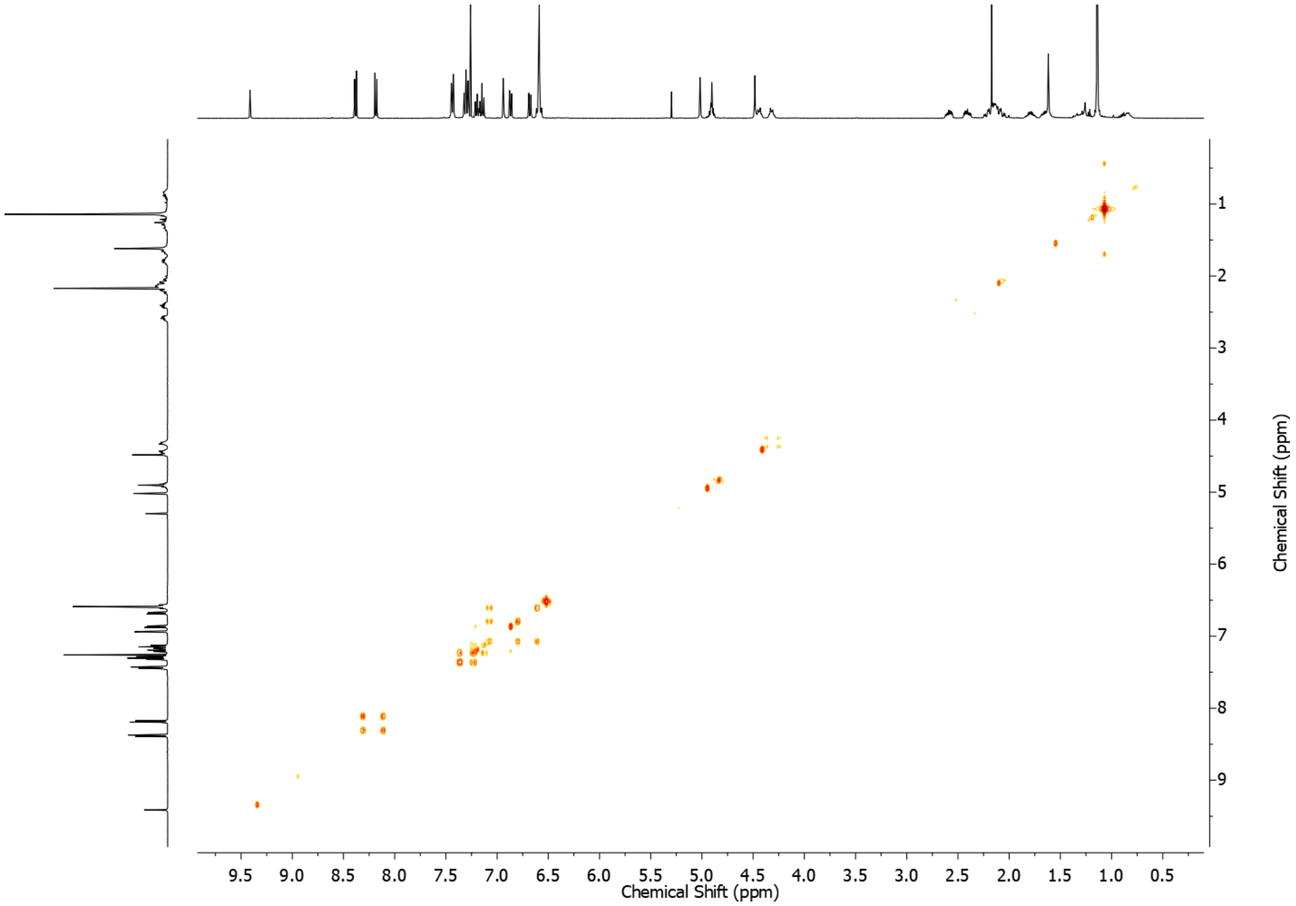

Figure S14 - ^{1}H COSY NMR (400 MHz, $CDCl_3$, 298 K) of **4b**.

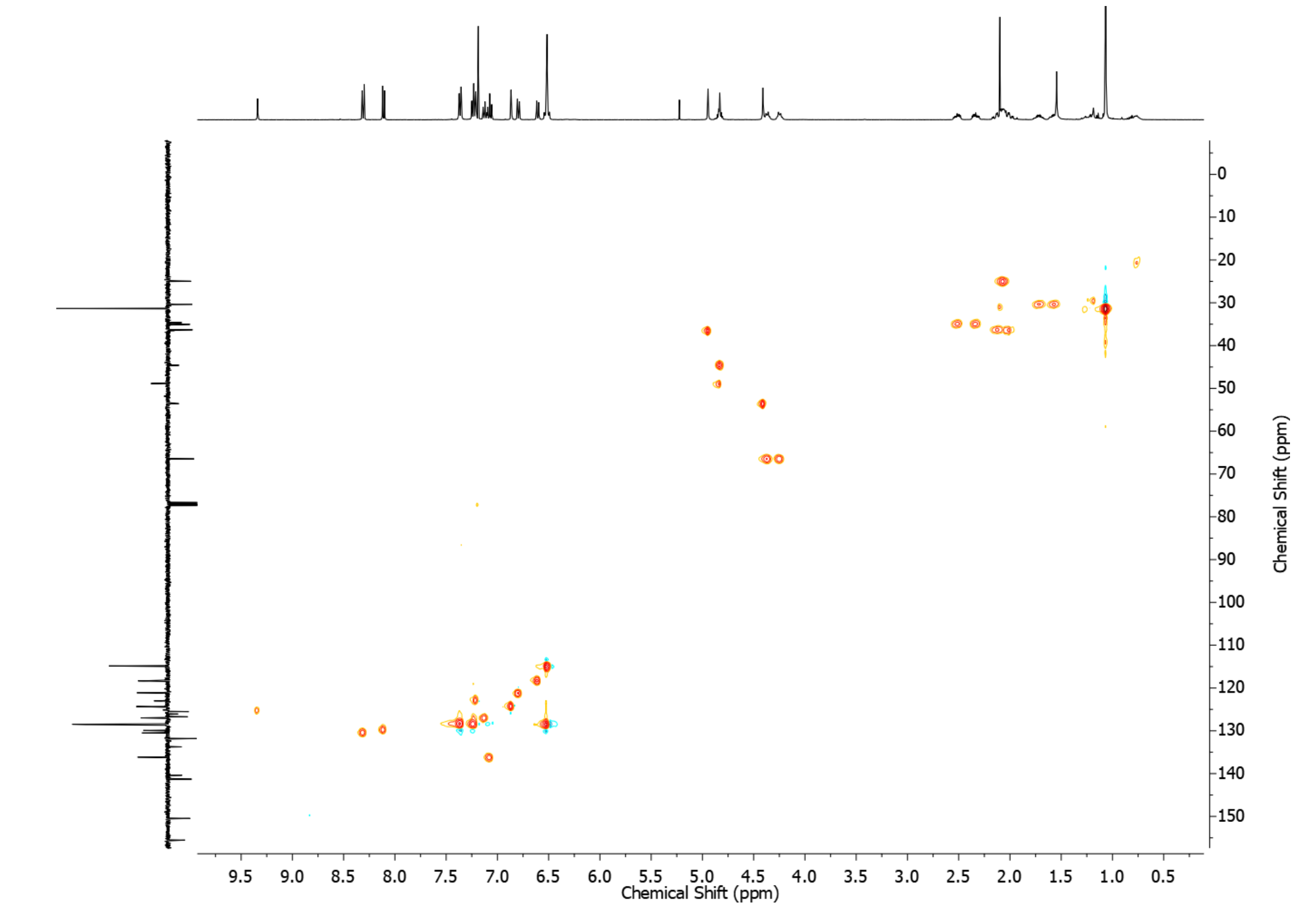

Figure S15 - ^{1}H-^{13}C HSQC NMR (400 MHz, $CDCl_3$, 298 K) of **4b**.

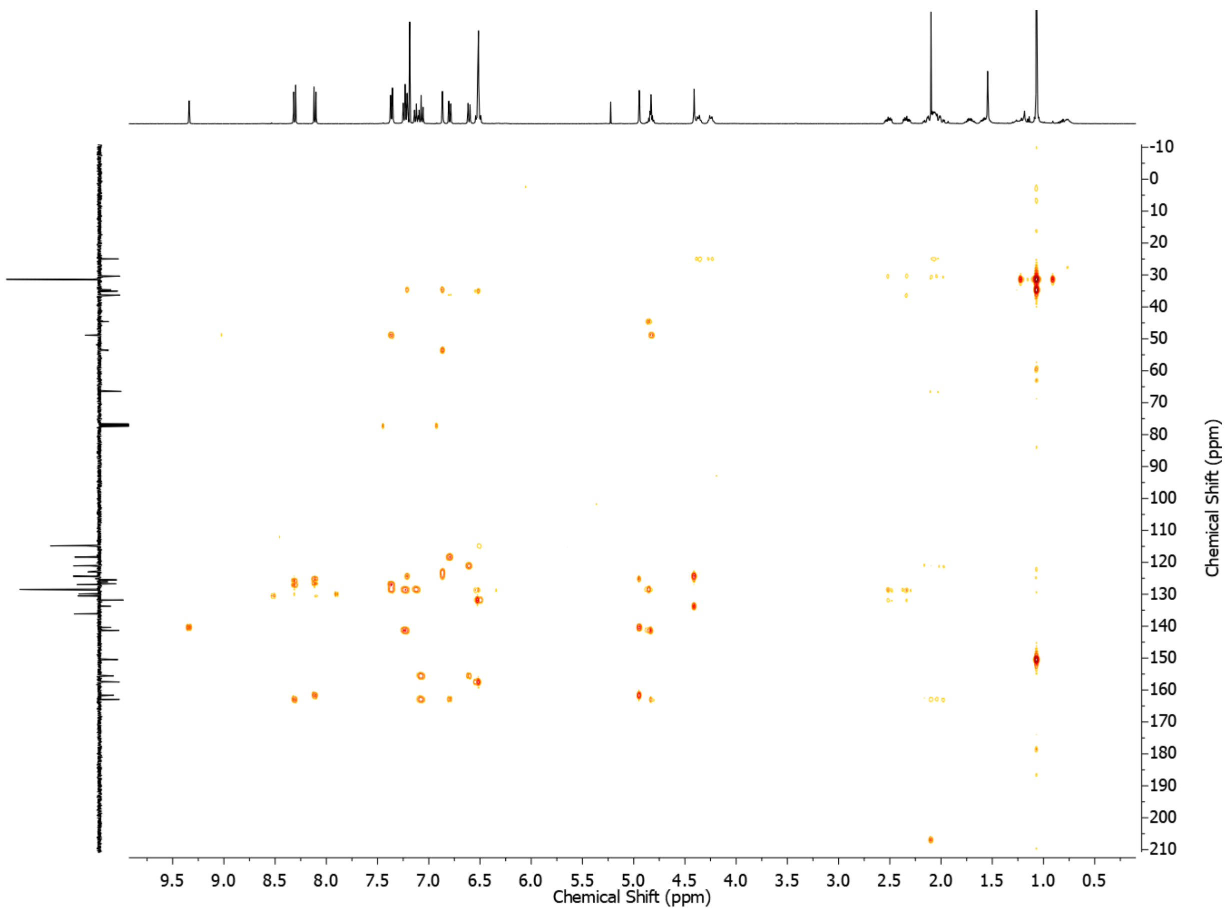

Figure S16 - ^{1}H-^{13}C HMBC NMR (400 MHz, $CDCl_3$, 298 K) of **4b**.

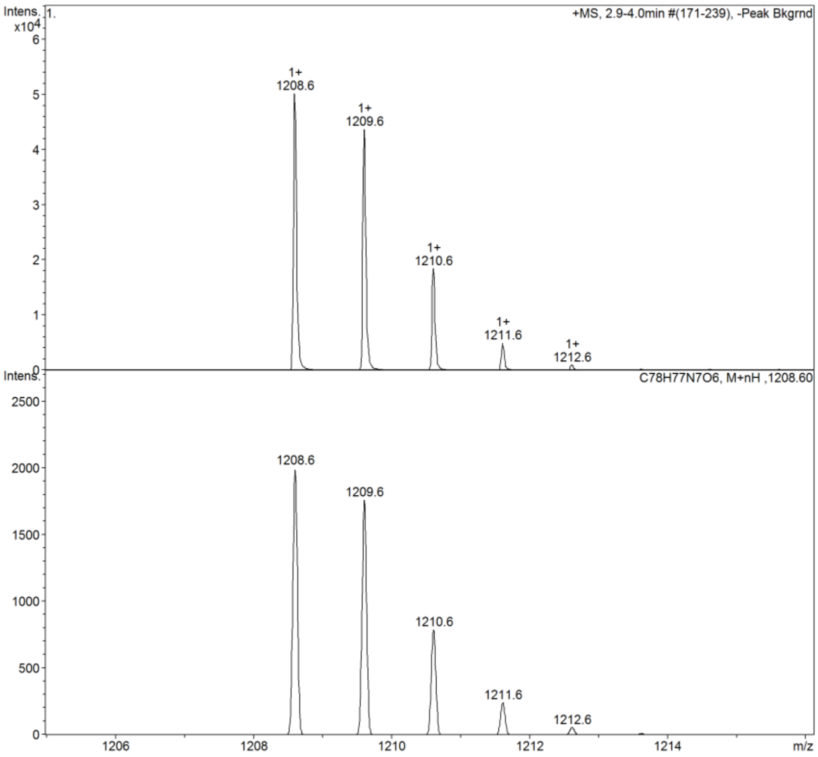

Figure S17 - Observed (top) and calculated (bottom) isotopic patterns for **4b**.

Rotaxane 4c

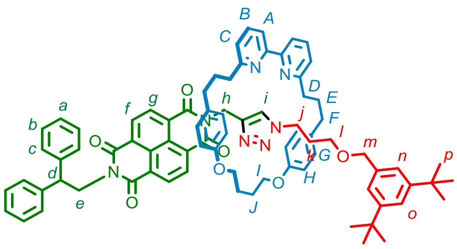

In a CEM vial, propargyl NDI **2** (87 mg, 0.18 mmol, 1.2 eq), bipyridine macrocycle **1** (72 mg, 0.15 mmol, 1.0 eq), azide **S2** (51 mg, 0.18 mmol, 1.2 eq) and $[Cu(MeCN)_4]PF_6$ (52 mg, 0.14 mmol, 0.96 eq) were dissolved in CH_2Cl_2 (3.75 mL). DIPEA (80 µL, 0.45 mmol, 3 eq) was added and the reaction was left for 16 h, after which TFA (23 µL, 0.30 mmol, 2 eq) was added. After 3 h, the reaction mixture was diluted with CH_2Cl_2 and washed with a saturated EDTA/NH_3 solution, water and brine. The organic layers were dried with $MgSO_4$, filtered and concentrated. Column chromatography (0 to 50% MeCN in CH_2Cl_2) gave **4c** as an orange foam (163 mg, 0.13 mmol, 86%). ^{1}H NMR (400 MHz, $CDCl_3$) δ 9.09 (s, 1H, H_i), 8.37 (d, *J* = 7.6, 2H, H_g), 8.17 (d, *J* = 7.6, 2H, H_f), 7.51 – 7.39 (m, 4H, H_c), 7.30 (t, *J* = 7.6, 4H, H_b), 7.27 – 7.25 (m, 1H, H_o), 7.22 – 7.15 (m, 2H, H_a), 7.06 (t, *J* = 7.8, 2H, H_B), 6.89 – 6.83 (m, 4H, H_A, H_n), 6.74 (d, *J* = 8.7, 4H, H_G), 6.69 (d, *J* = 8.7, 4H, H_H), 6.43 (d, *J* = 7.8, 2H, H_C), 5.44 (s, 2H, H_h), 5.03 – 4.86 (m, 3H, H_d, H_e), 4.53 (q, *J* = 7.7, 2H, H_l), 4.06 – 3.97 (m, 2H, H_I), 3.94 (s, 2H, H_m), 2.99 – 2.86 (m, 4H, H_j, H_l), 2.74 – 2.61 (m, 2H, H_F), 2.53 – 2.32 (m, 4H, H_F, H_D), 2.24 – 2.12 (m, 4H, H_J, H_D), 2.04 – 1.80 (m, 4H, H_J, H_D), 1.66 – 1.47 (m, 2H, H_E), 1.23 (s, 18H, H_p), 1.12 – 1.00 (m, 2H, H_k). ^{13}C NMR (100 MHz, $CDCl_3$) δ 163.0, 163.0, 161.9, 157.5, 155.2, 150.6, 142.2, 141.4, 137.7, 136.2, 132.8, 130.7, 130.2, 129.3, 128.7, 128.6, 127.1, 126.4, 126.2, 125.8, 123.7, 121.8, 121.5, 121.3, 118.4, 114.9, 72.5, 67.6, 66.1, 49.0, 46.8, 44.7, 37.0, 36.5, 35.2, 34.8, 31.6, 31.4, 27.7, 24.8. LR-MS (ESI) m/z = 1266.6 $[M+H]^+$

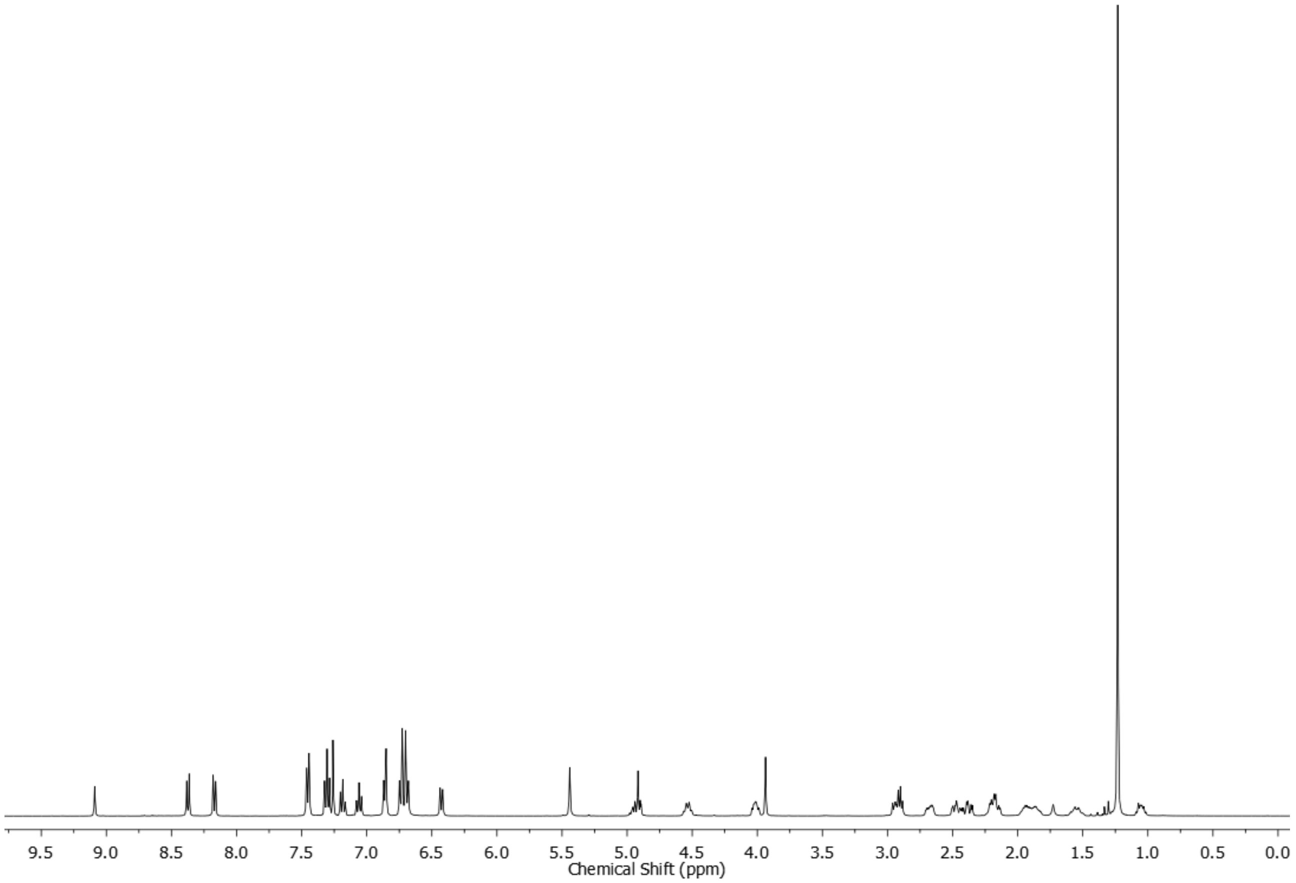

Figure S18 - ^{1}H NMR (400 MHz, $CDCl_3$, 298 K) of **4c**

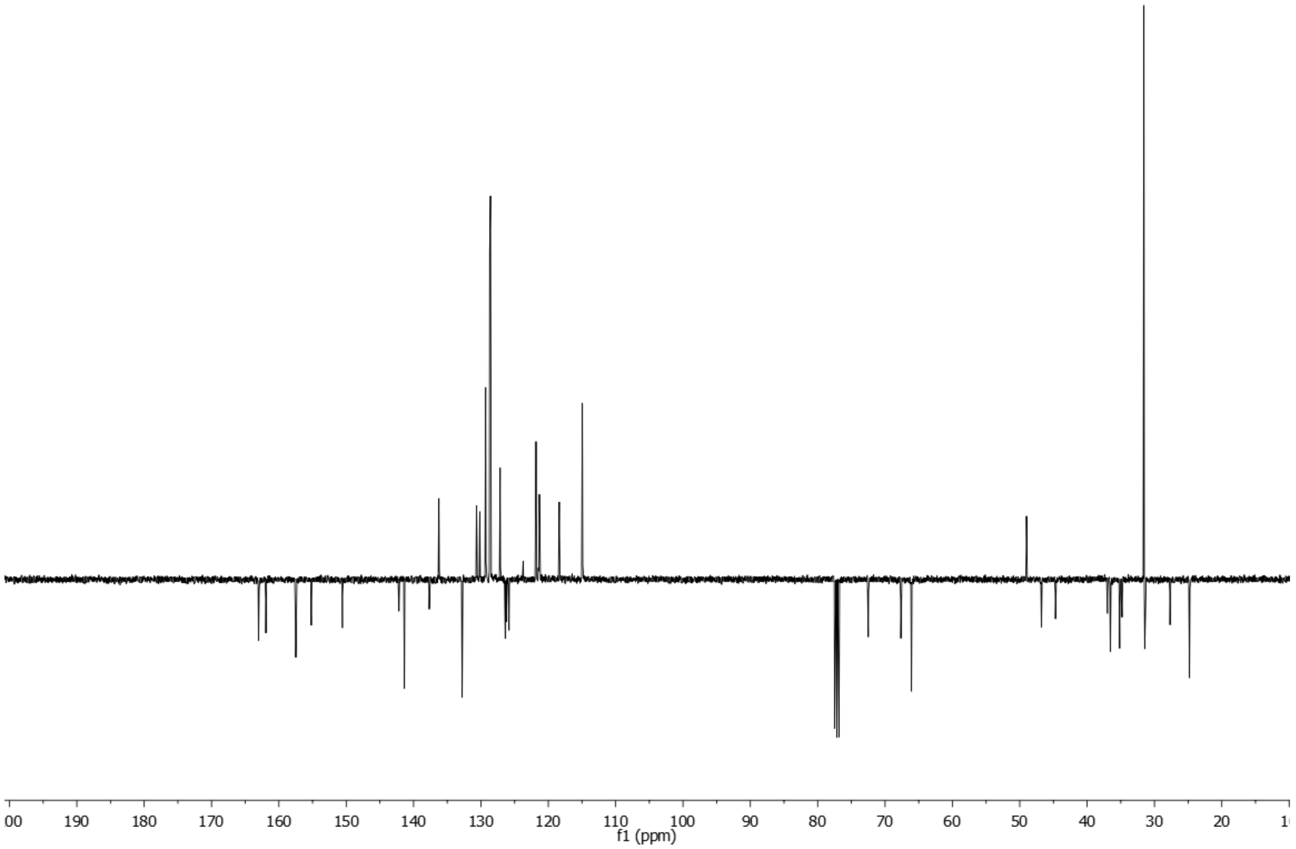

Figure S19 - ^{13}C-JMOD NMR (100 MHz, $CDCl_3$, 298 K) of **4c**

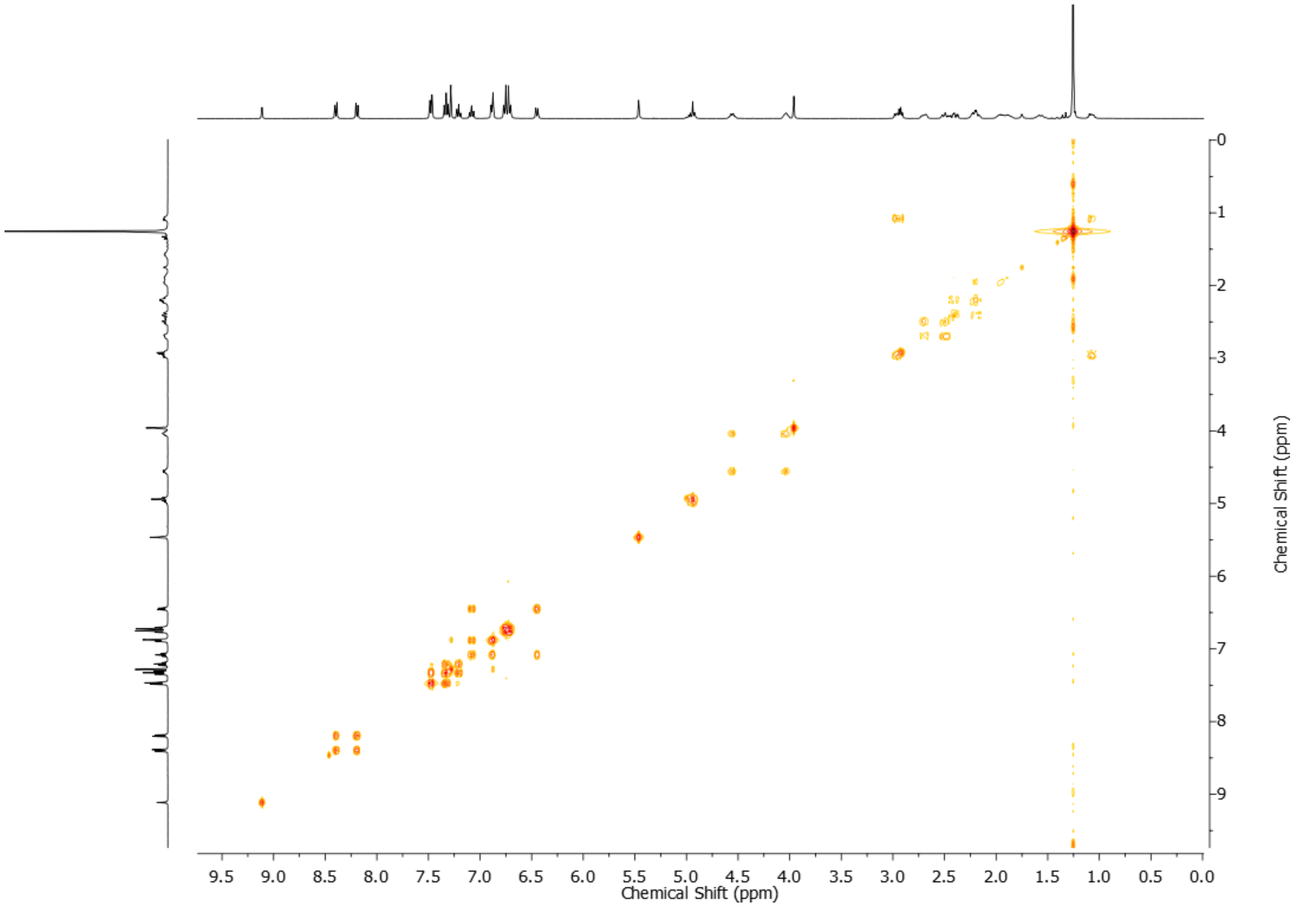

Figure S20 - ^{1}H COSY NMR (400 MHz, $CDCl_3$, 298 K) of **4c**

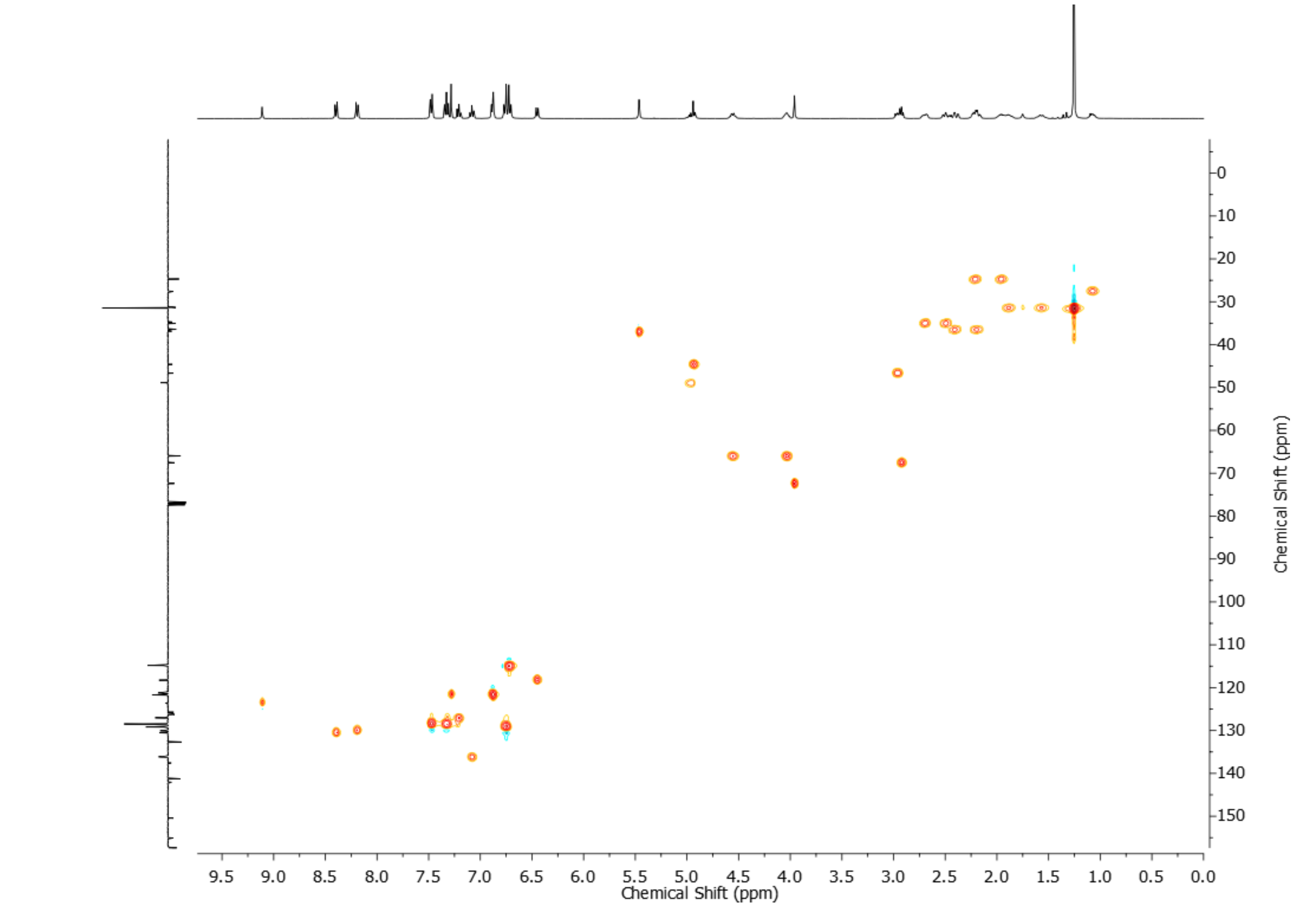

Figure S21 - ^{1}H-^{13}C HSQC NMR (400 MHz, $CDCl_3$, 298 K) of **4c**

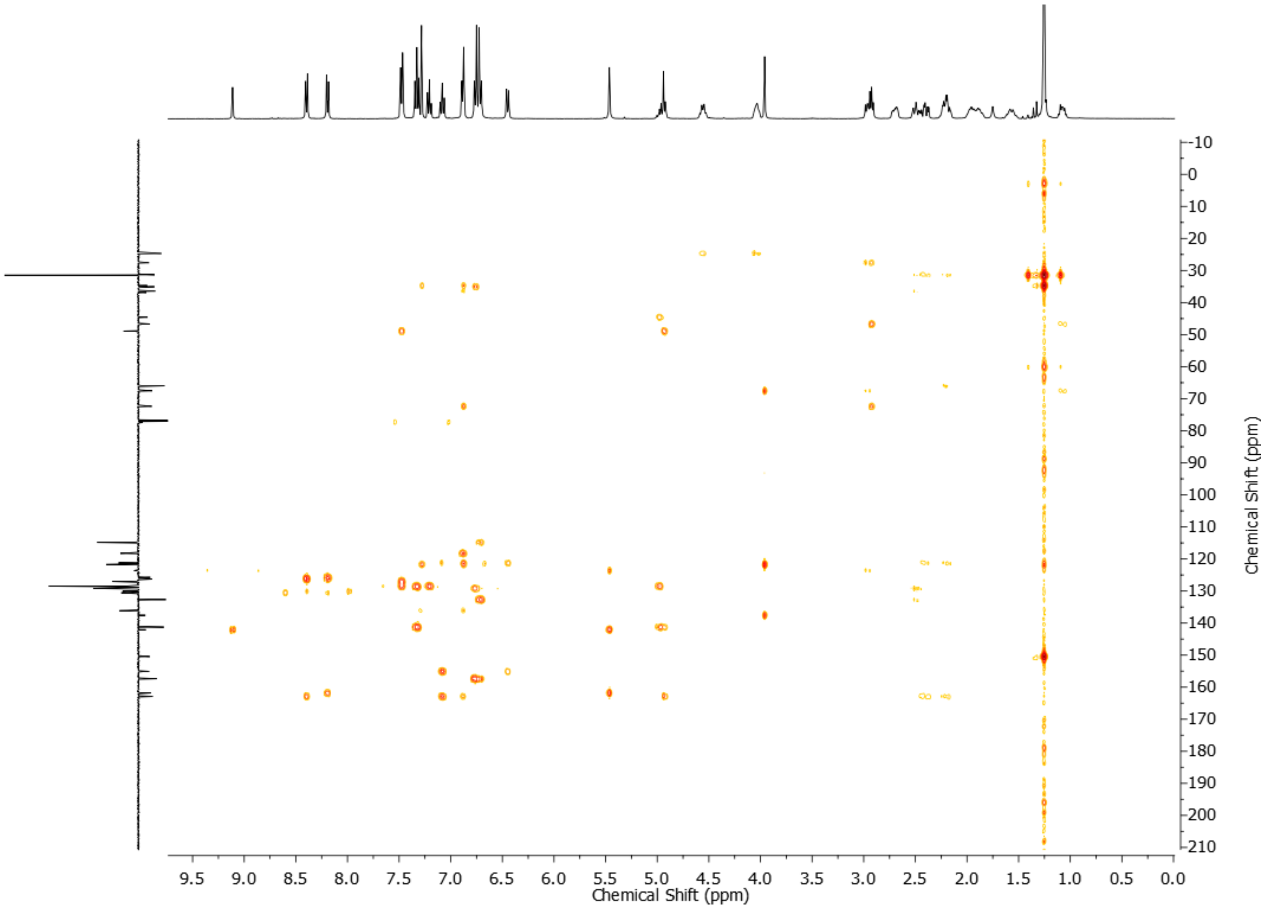

Figure S22 - ^1H-^{13}C HMBC NMR (400 MHz, $CDCl_3$, 298 K) of **4c**

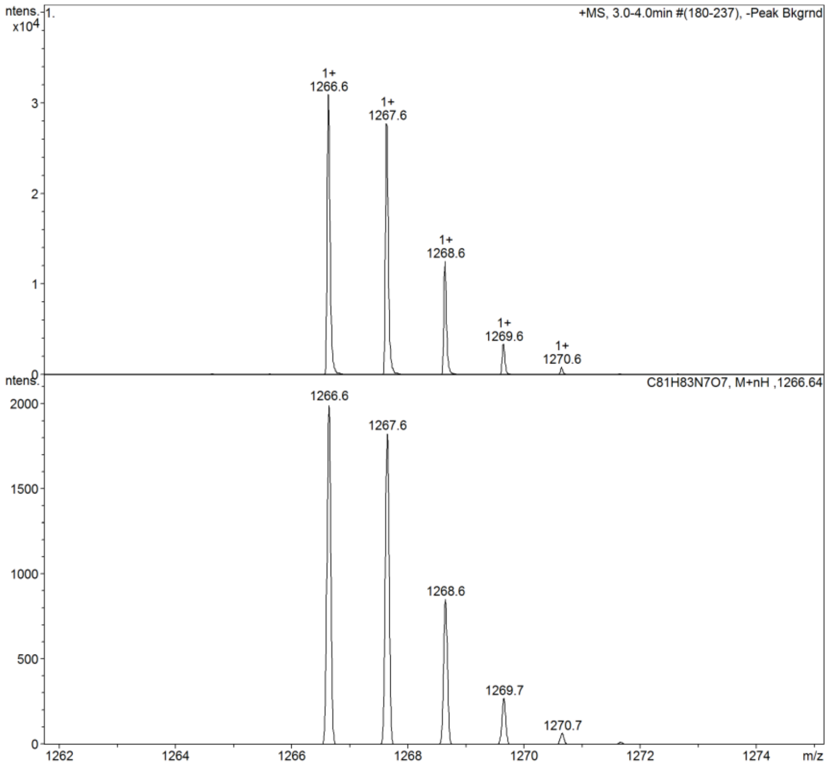

Figure S23 - Observed (top) and calculated (bottom) isotopic patterns for **4c**.

Axle 5

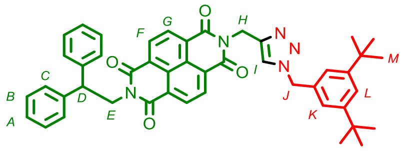

To a CEM vial charged with propargyl NDI **2** (90 mg, 0.19 mmol, 1 eq) and azide **S1** (68.6 mg, 0.28 mmol, 1.5 eq) was added $[Cu(MeCN)_4]PF_6$ (14.9 mg, 0.04 mmol, 0.2 eq). The solids were then dissolved in CH_2Cl_2 (3 mL) before addition of DIPEA (66 µL, 0.38 mmol, 2 eq). After 16 h the solution was washed with a saturated EDTA/NH_3 solution, the organics were dried with $MgSO_4$ and concentrated *in vacuo*. Purification by column chromatography (0 to 8% MeOH in CH_2Cl_2) yielded axle **5** as a pale purple foam (120 mg, 0.16 mmol, 87%). ^1H NMR (400 MHz, $CDCl_3$) δ 8.70 (d, *J* = 7.6, 2H, H_G), 8.63 (d, *J* = 7.6, 2H, H_F), 7.60 (s, 1H, H_I), 7.39 (t, *J* = 1.8, 1H, H_L), 7.36 – 7.31 (m, 4H, H_C), 7.25 – 7.20 (m, 4H, H_B), 7.17 – 7.11 (m, 2H, H_A), 7.06 (d, *J* = 1.8, 2H, H_K), 5.48 (s, 2H, H_H), 5.46 (s, 2H, H_J), 4.88 (d, *J* = 8.0, 2H, H_E), 4.77 (t, *J* = 8.0, 1H, H_D), 1.27 (s, 18H, H_M). ^{13}C NMR (100 MHz, $CDCl_3$) δ 162.8, 162.6, 151.8, 143.1, 141.3, 133.8, 131.2, 131.0, 128.5, 126.9, 126.8, 126.6, 126.6, 126.5, 123.4, 122.8, 122.4, 54.8, 48.7, 44.9, 35.6, 34.9, 31.4. HR-ESI-MS (+ve) m/z = 730.3395 $[M+H]^+$ (calc. m/z for $C_{46}H_{44}N_5O_4$ 730.3388).

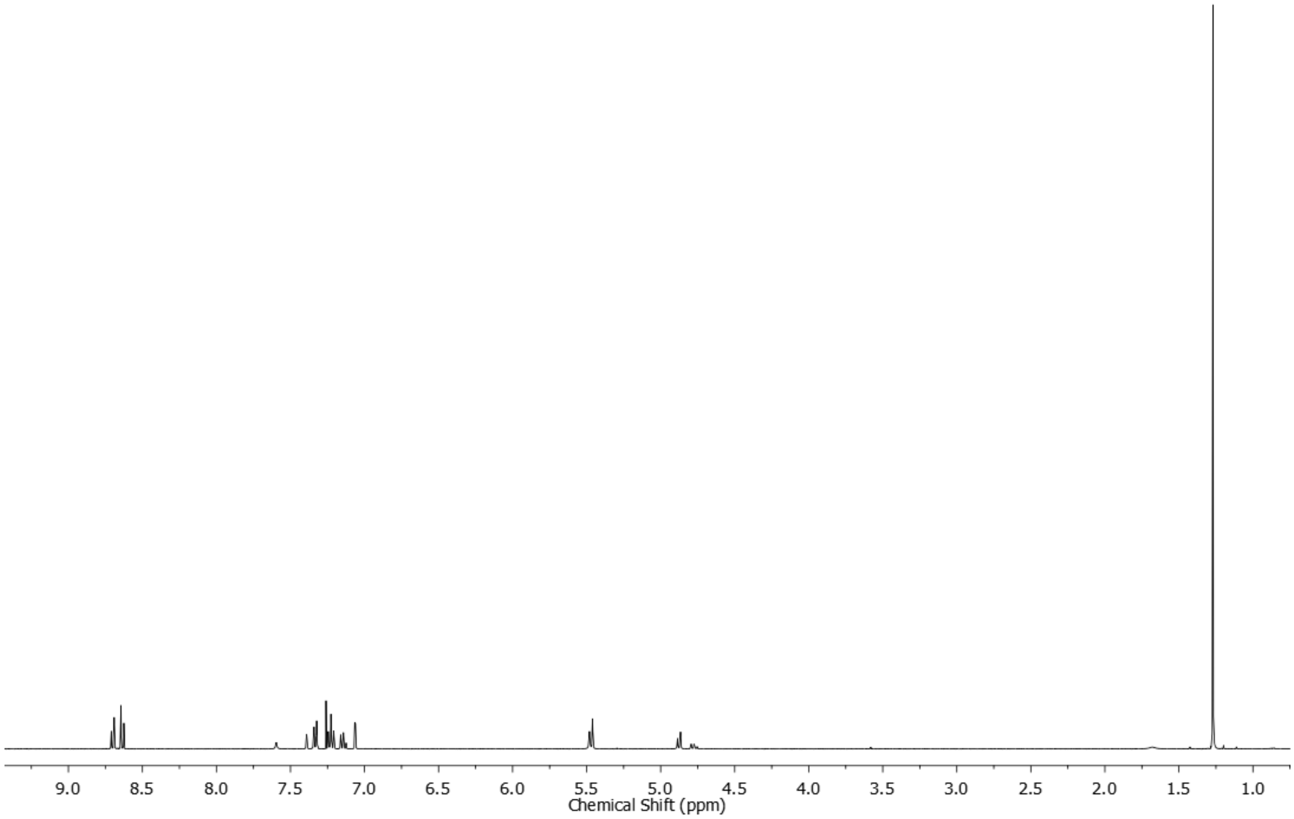

Figure S24 - ^1H NMR (400 MHz, $CDCl_3$, 298 K) of axle **5**.

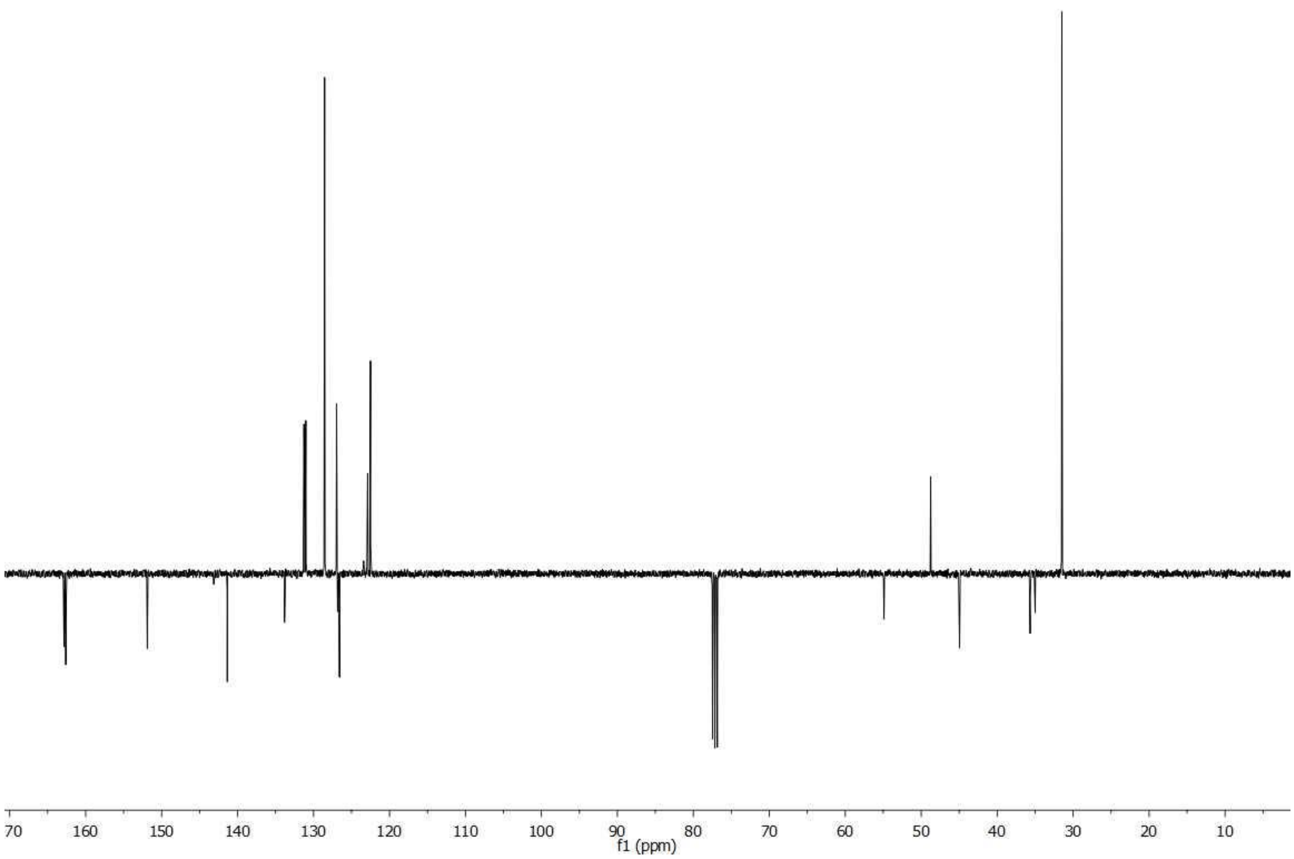

Figure S25 - ^{13}C-JMOD NMR (100 MHz, $CDCl_3$, 298 K) of axle **5**

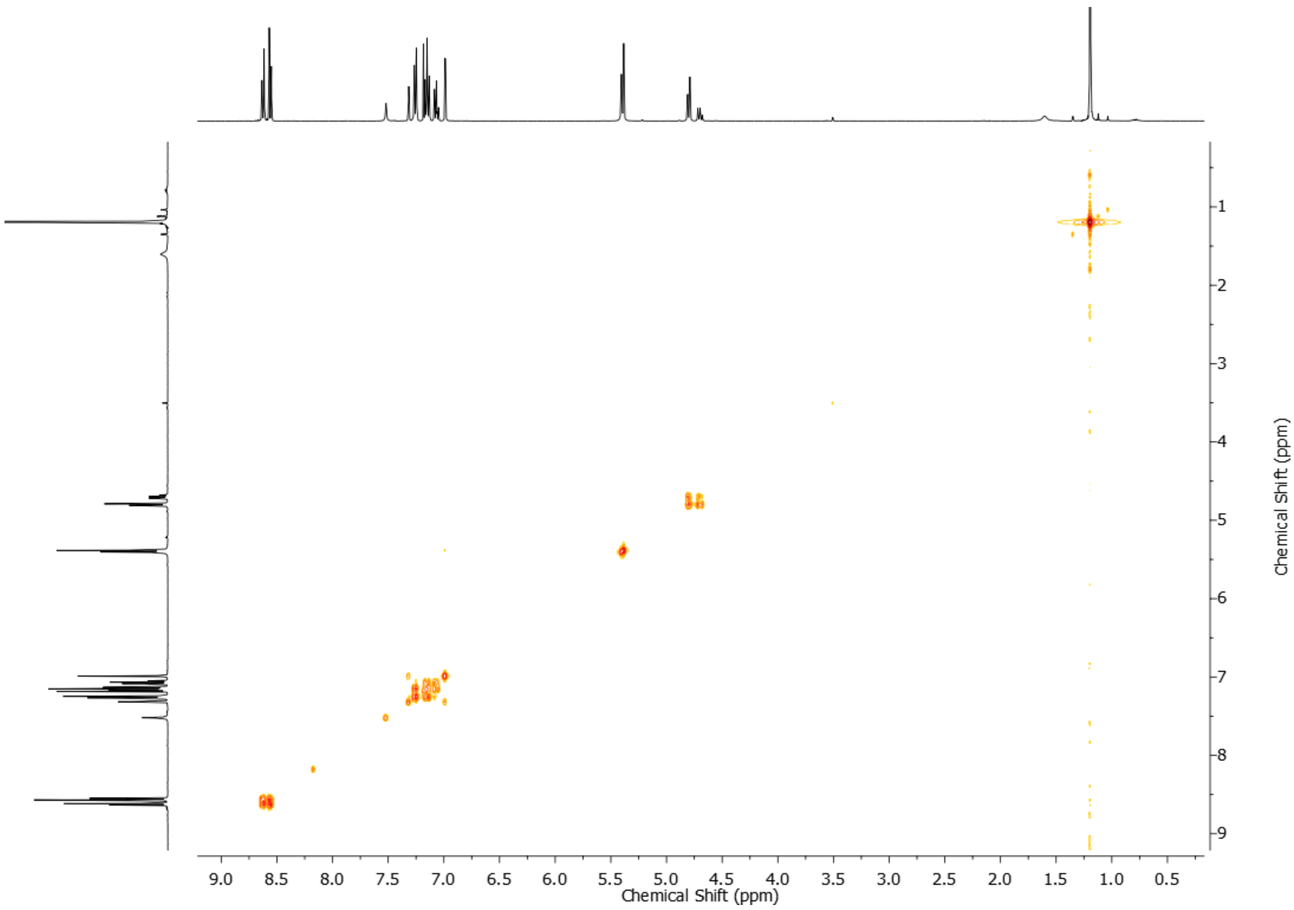

Figure S26 - ^{1}H COSY NMR (400 MHz, $CDCl_3$, 298 K) of axle **5**

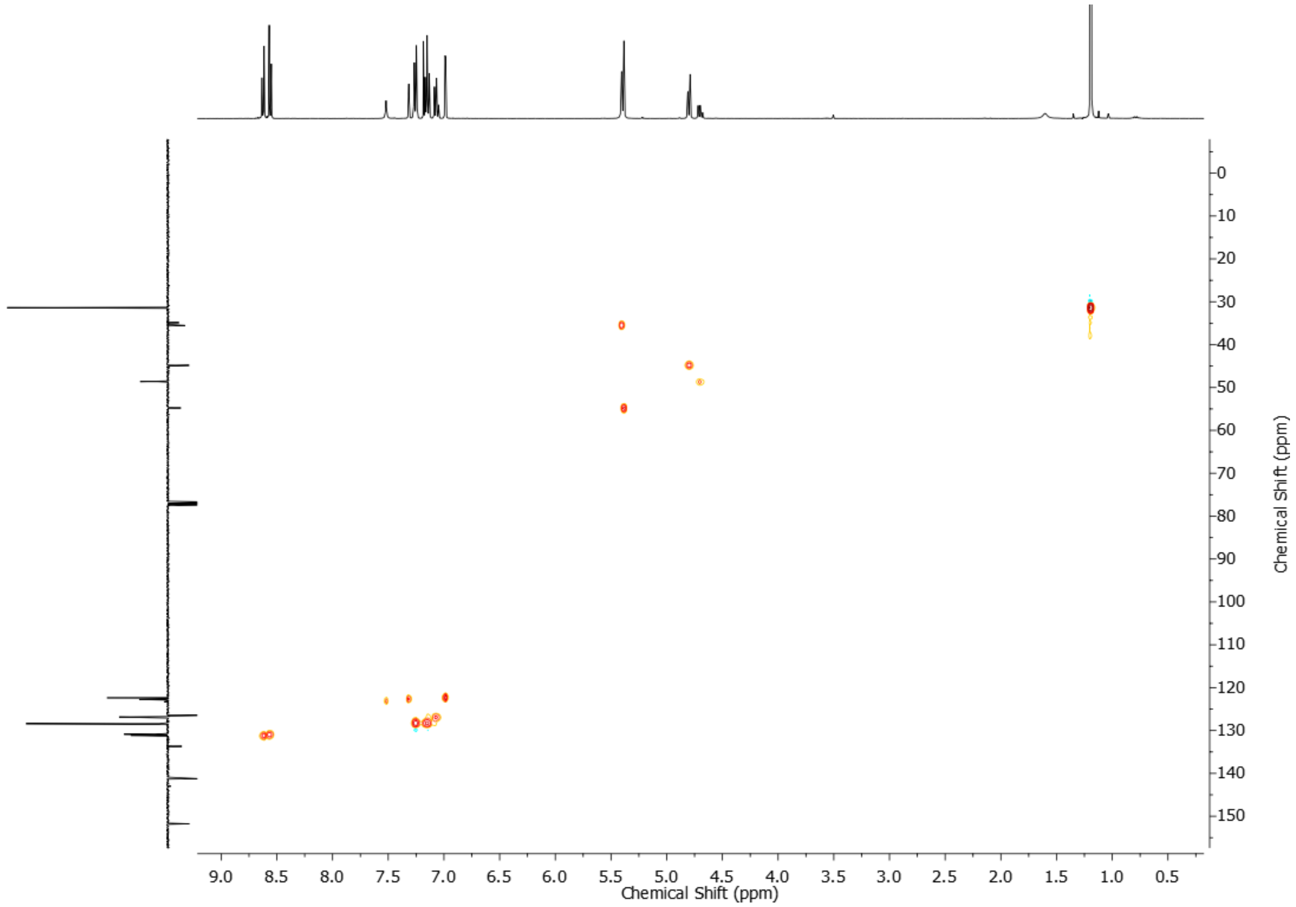

Figure S27 - ^{1}H-^{13}C HSQC NMR (400 MHz, $CDCl_3$, 298 K) of axle **5**

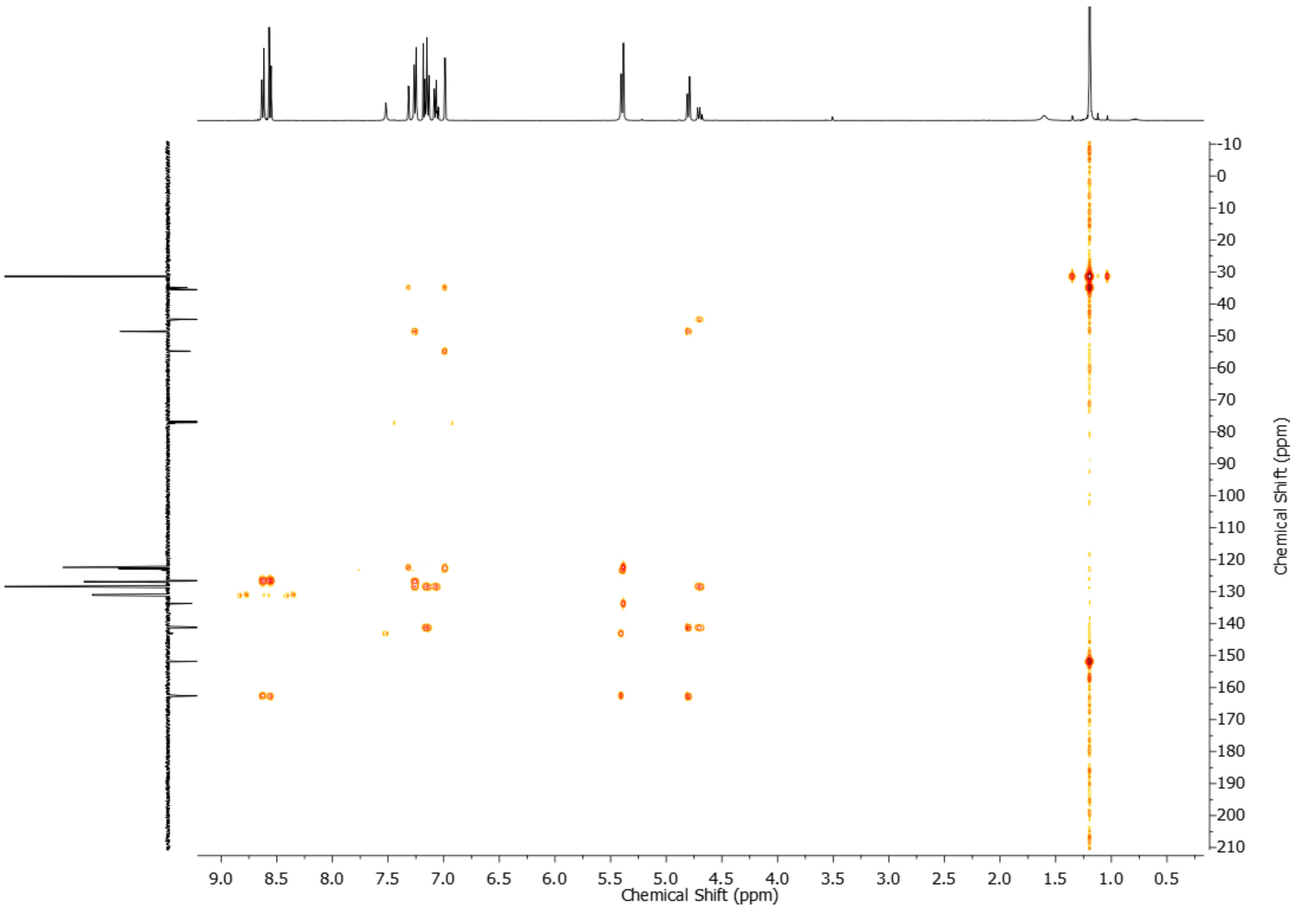

Figure S28 - ^{1}H-^{13}C HMBC NMR (400 MHz, $CDCl_3$, 298 K) of axle **5**

Imide half axle S4

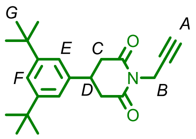

To a solution of dicyclohexyl carbodiimide (182 mg, 0.88 mmol. 1.10 eq.) in DMF (1.6 mL) at 0 °C was added bis-carboxylic acid **S3** (257 mg, 0.80 mmol, 1.00 eq.), and the reaction mixture was stirred for 1.5 h. Propargyl amine (54 µL, 0.84 mmol, 1.05 eq.), and NEt_3 (117 µL, 0.84 mmol, 1.05 eq.) were then added, and the reaction was stirred at ambient temperature for 16 h. The reaction mixture was then filtered through Celite®, and concentrated *in vacuo*. NaOAc (197 mg, 2.40 mmol, 3.00 eq.) and acetic anhydride (4 mL) were added, and the reaction mixture was heated at 100 °C for 2 h. After cooling to ambient temperature, the crude reaction mixture was poured onto iced water (8 mL) and stirred for 30 min. The suspension was then made alkaline with 5 M NaOH, whilst cooling at 0 °C, stirred for 15 min, before extracting the aqueous with CH_2Cl_2 (3 x 10 mL).The combined organics were dried over $MgSO_4$, filtered, and concentrated *in vacuo*. Column chromatography (0 to 20% EA in CH_2Cl_2) yielded **S4** as a colourless foam (134 mg, 0.38 mmol, 49%). ^{1}H NMR (400 MHz, $CDCl_3$) δ 7.36 (t, *J* = 1.8, 1H, H_F), 7.03 (dd, *J* = 1.7, 0.5, 2H, H_E), 4.58 (d, *J* = 2.4, 2H, H_B), 3.47 – 3.32 (m, 1H, H_D), 3.17 – 3.00 (m, 2H, H_C), 2.96 – 2.78 (m, 2H, H_C), 2.14 (t, *J* = 2.5, 1H, H_A), 1.32 (s, 18H, H_G). ^{13}C NMR (101 MHz, $CDCl_3$) δ 171.1, 151.8, 139.7, 122.0, 120.5, 78.6, 70.6, 40.1, 35.1, 31.6, 29.0. HR-ESI-MS (+ve) m/z = 362.2096 $[M+Na]^+$ (calc. m/z for $C_{22}H_{29}NNaO_2$ 362.2091).

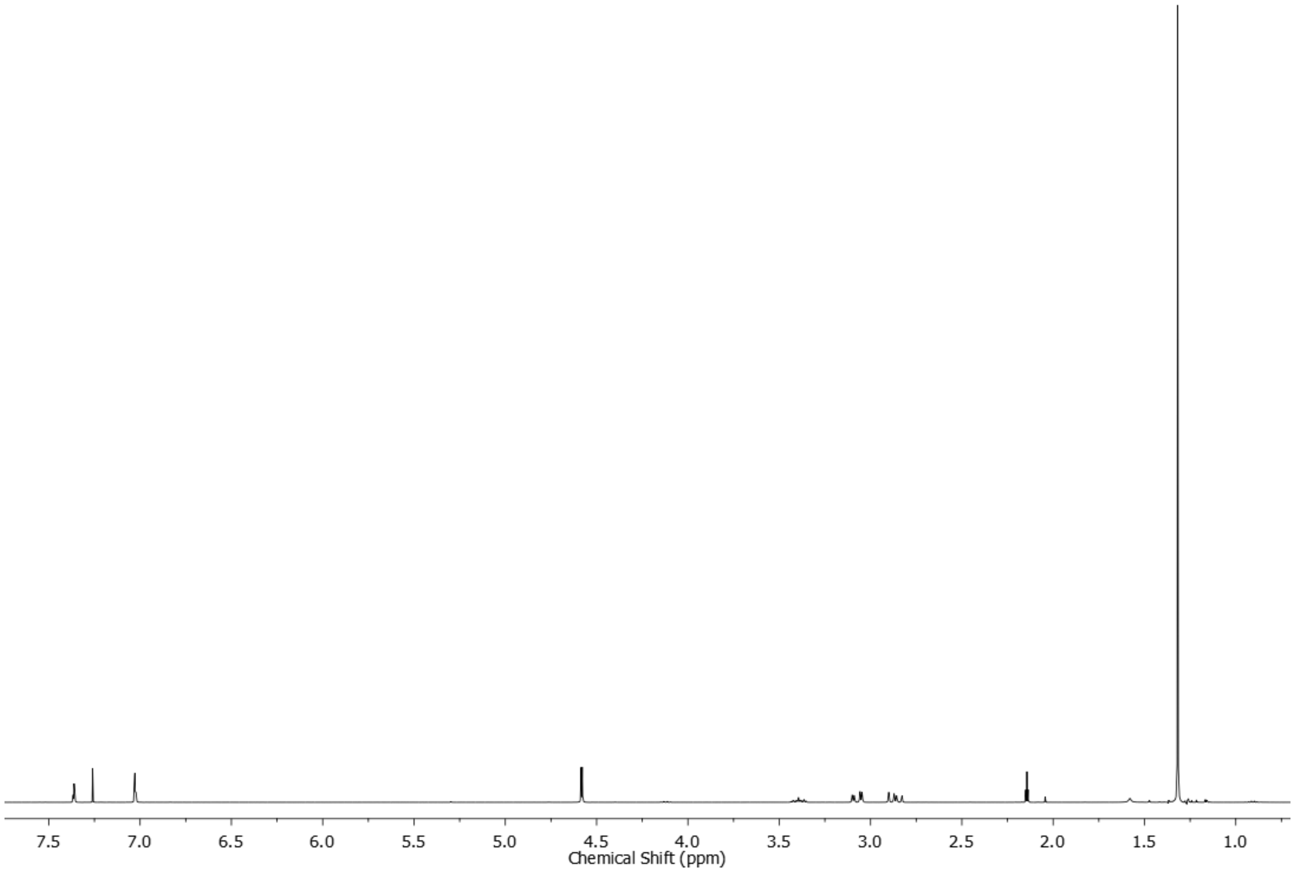

Figure S29 - ^{1}H NMR (400 MHz, $CDCl_3$, 298 K) of **S4**

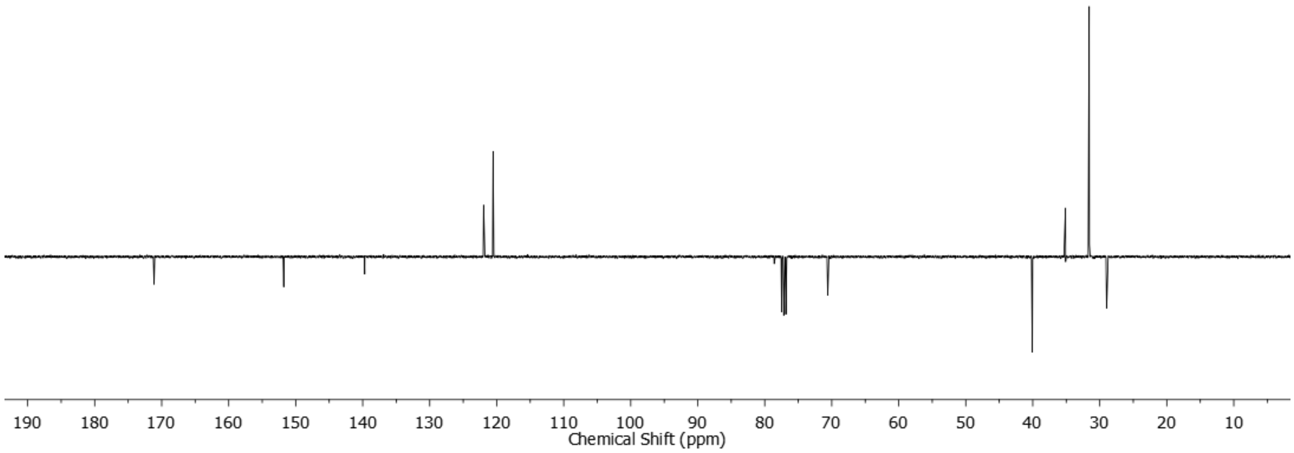

Figure S30 - ^{13}C-JMOD NMR (100 MHz, $CDCl_3$, 298 K) of **S4**

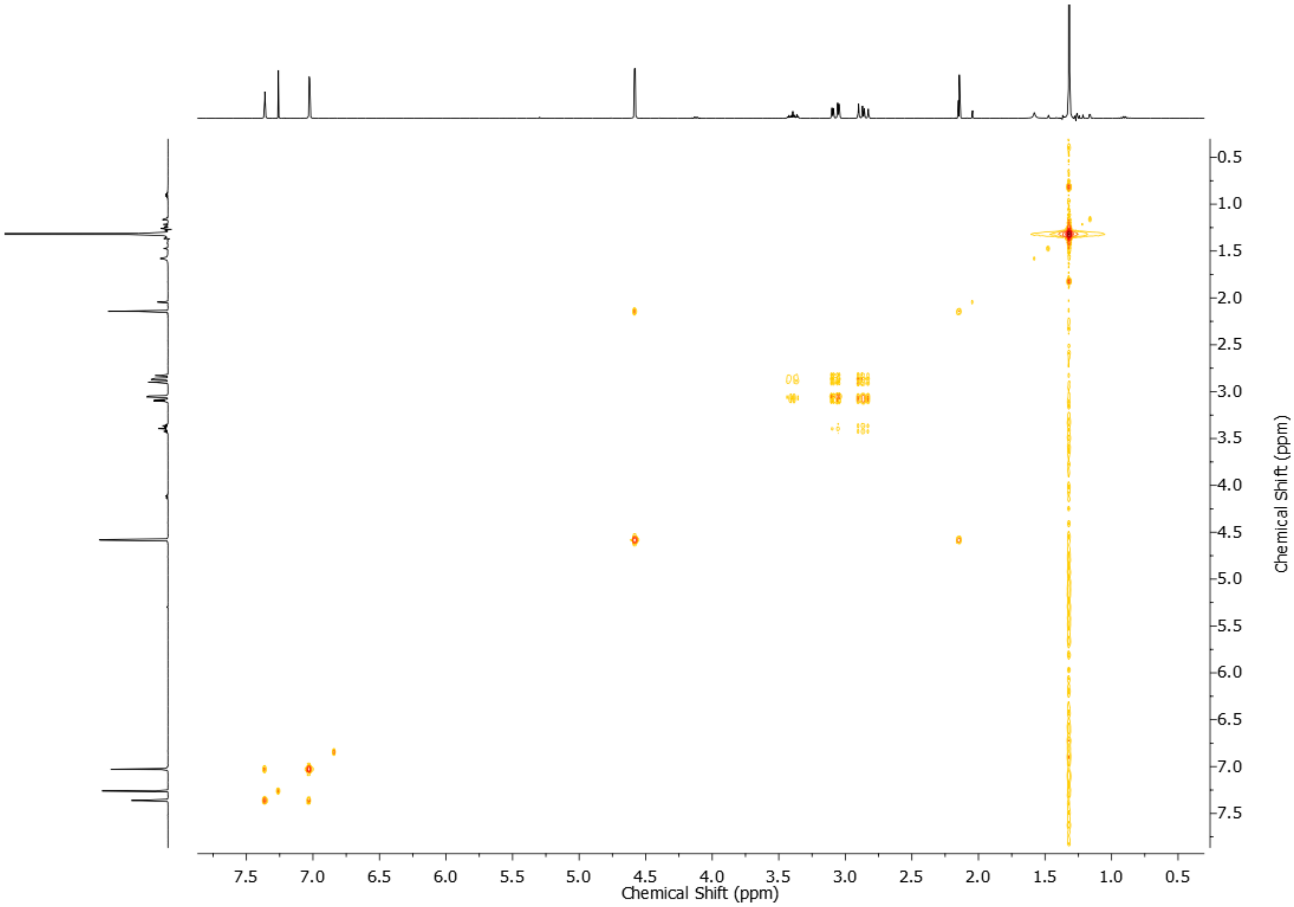

Figure S31 - ^{1}H COSY NMR (400 MHz, $CDCl_3$, 298 K) of **S4**

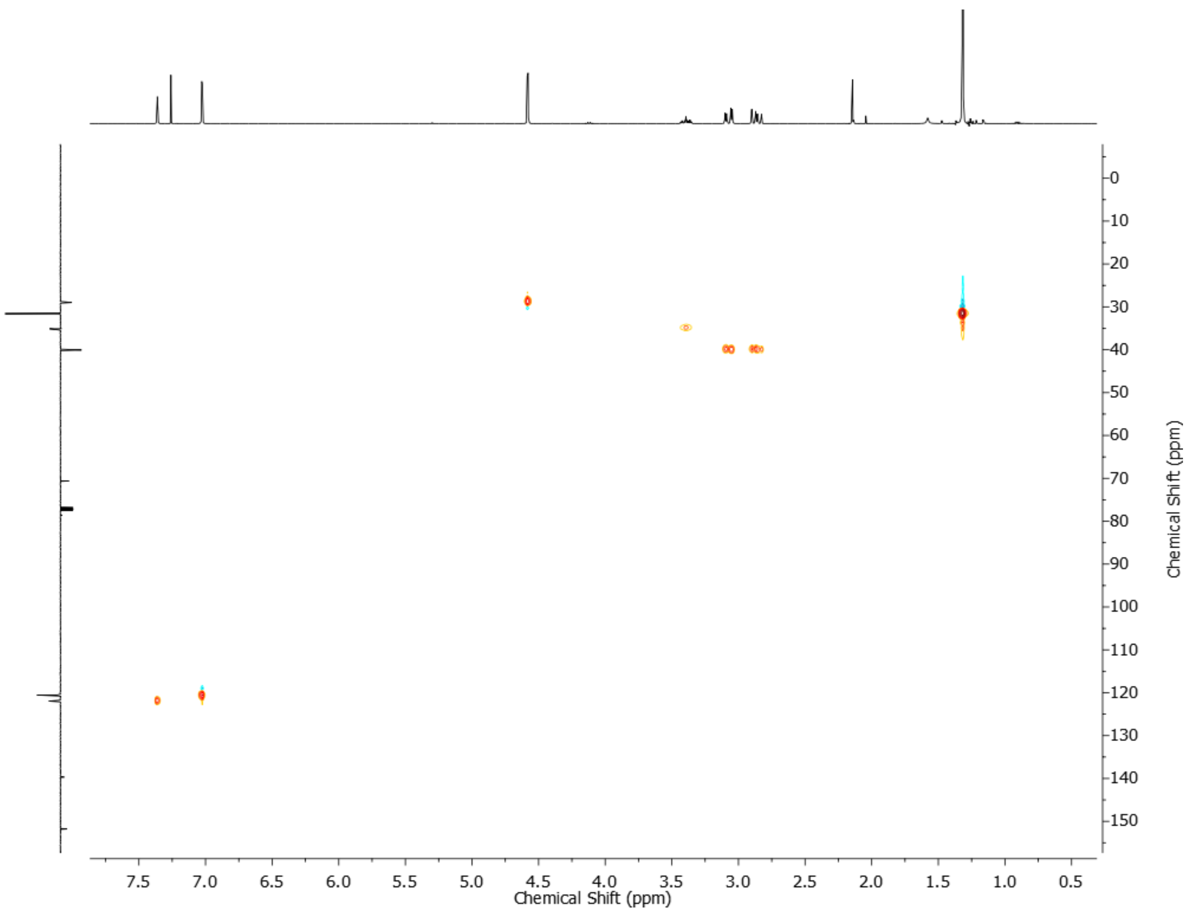

Figure S32 - ^{1}H-^{13}C HSQC NMR (400 MHz, $CDCl_3$, 298 K) of **S4**

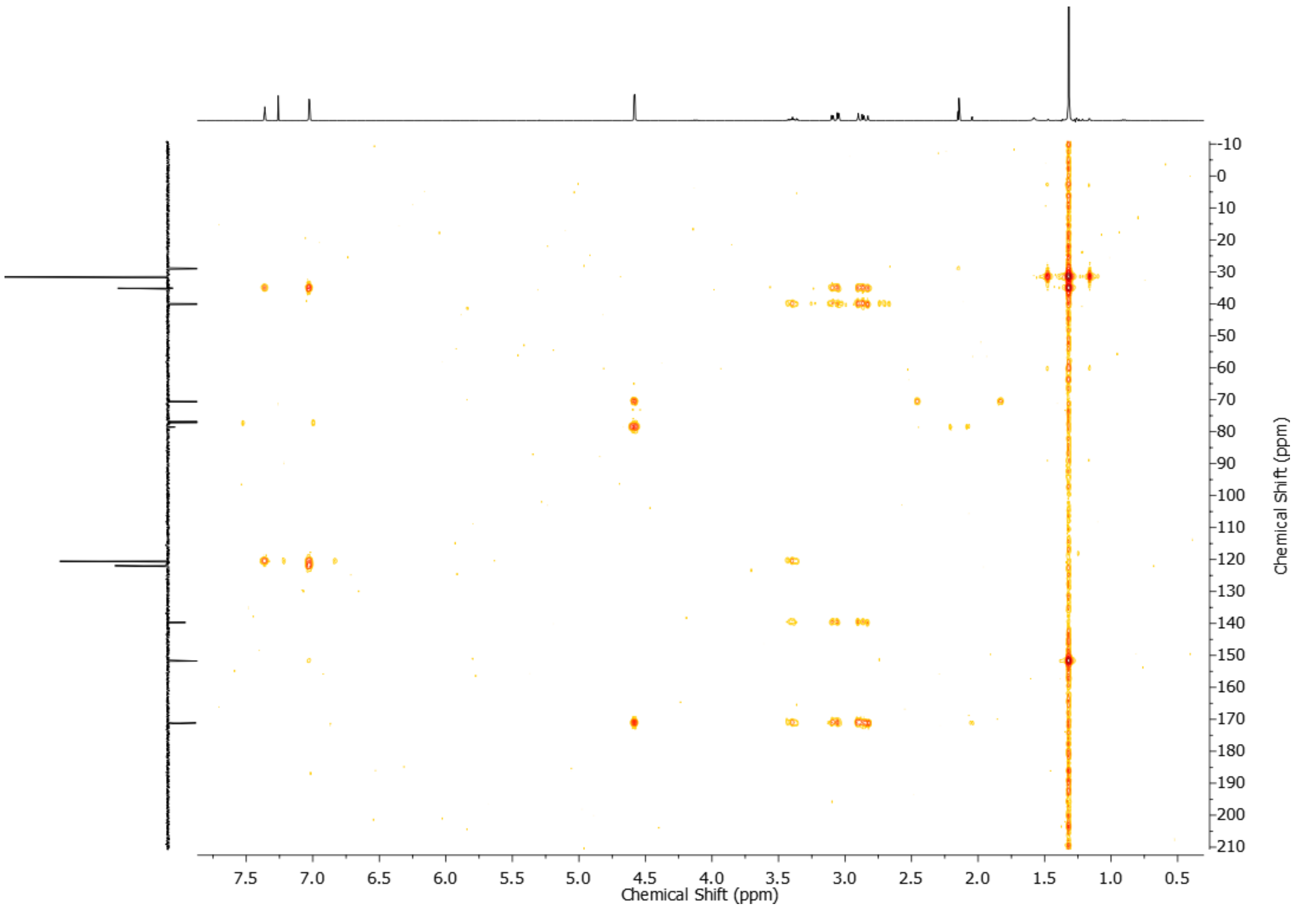

Figure S33 - ^{1}H-^{13}C HMBC NMR (400 MHz, $CDCl_3$, 298 K) of **S4**

Rotaxane 6

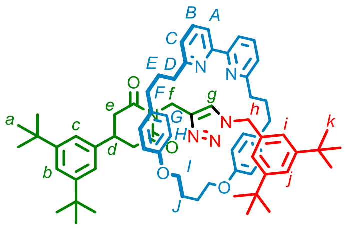

In a CEM vial, alkyne **S4** (28 mg, 0.082 mmol, 1.1 eq), bipyridine macrocycle **1** (36 mg, 0.075 mmol, 1.0 eq), azide **S1** (22 mg, 0.090 mmol, 1.2 eq) and $[Cu(MeCN)_4]PF_6$ (27 mg, 0.072 mmol, 0.95 eq) were dissolved in CH_2Cl_2 (1.9 mL). DIPEA (40 µL, 0.225 mmol, 3.0 eq) was added and the reaction was left for 16 h, after which TFA (11 µL, 0.15 mmol, 2.0 eq) was added. After 3 h, the reaction mixture was diluted with CH_2Cl_2 and washed with a saturated EDTA/NH_3 solution, water and brine. The organic layers were dried with $MgSO_4$, filtered and concentrated. Column chromatography (0 to 50% MeCN in CH_2Cl_2) gave **6** as a colourless foam (44 mg, 0.042 mmol, 56%). ^{1}H NMR (400 MHz, $CDCl_3$) δ 8.70 (s, 1H, H_g), 7.56 (t, *J* = 7.7, 2H, H_B), 7.34 (d, *J* = 7.5, 2H, H_A), 7.27 – 7.20 (m, 2H, H_b, H_j), 7.11 (d, *J* = 7.6, 2H, H_C), 6.87 (s, 2H, H_i), 6.71 (s, 2H, H_c), 6.54 (d, *J* = 8.4, 4H, H_G), 6.47 (d, *J* = 8.3, 4H, H_H), 4.62 (s, 2H, H_f), 4.54 – 4.41 (m, 1H, H_I), 4.17 (s, 2H, H_h), 4.14 – 4.02 (m, 2H, H_I), 3.05 (t, *J* = 12.6, 1H, H_d), 2.68 – 2.54 (m, 2H, H_e), 2.51 – 2.33 (m, 8H, H_e, H_D, H_F), 2.34 – 2.21 (m, 2H, H_D), 2.19 – 2.07 (m, 2H, H_J), 1.98 (dd, *J* = 12.1, 5.9, 2H, H_J), 1.85 – 1.48 (m, 4H, H_E), 1.20 (s, 18H,), 1.15 (s, 18H). ^{13}C NMR (101 MHz, $CDCl_3$) δ 171.1, 140.6, 140.3, 136.7, 133.5, 132.3, 128.7, 124.7, 123.9, 122.9, 121.5, 121.4, 120.3, 119.8, 115.0, 66.6, 53.2, 40.0, 36.7, 35.5, 34.9, 34.8, 34.7, 31.5, 31.1, 25.0. LR-ESI-MS (+ve) m/z = 1064.0 $[M+H]^+$

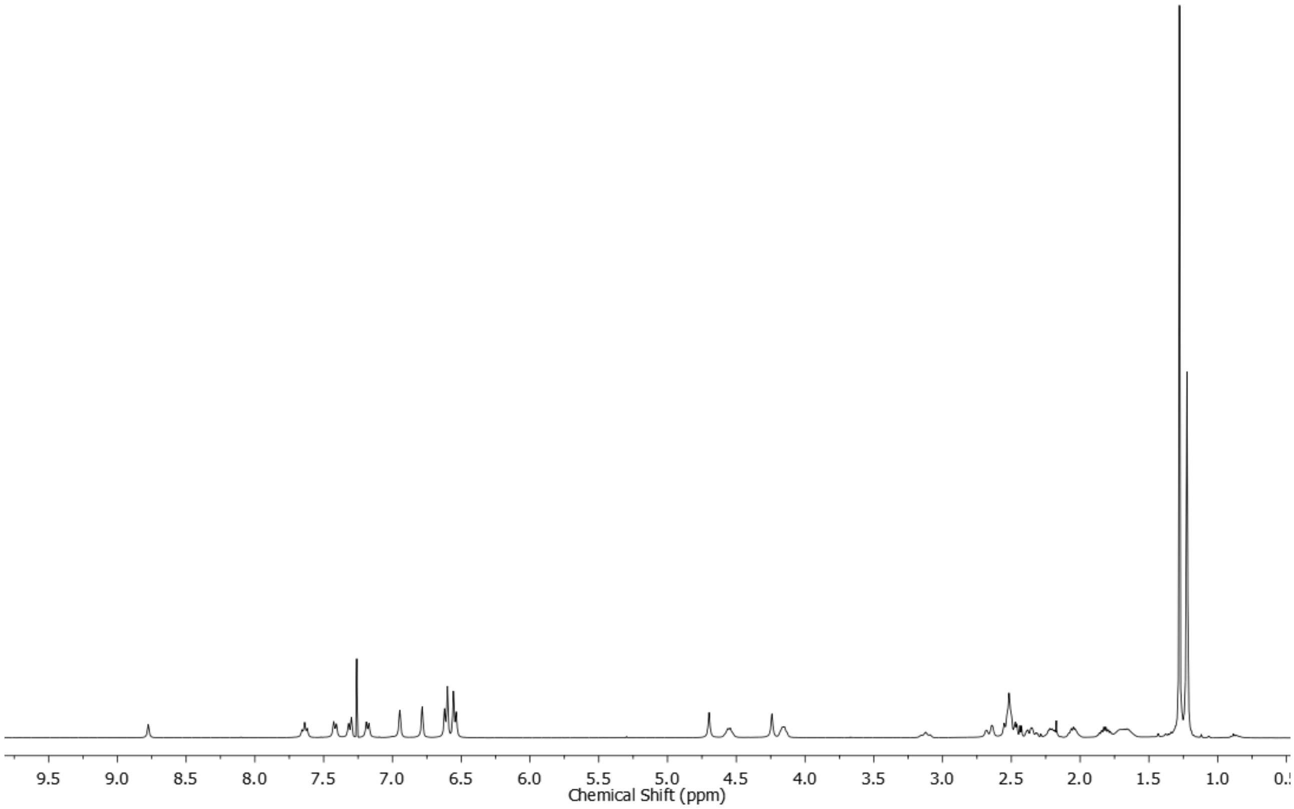

Figure S34 - ^{1}H NMR (400 MHz, $CDCl_3$, 298 K) of **6**.

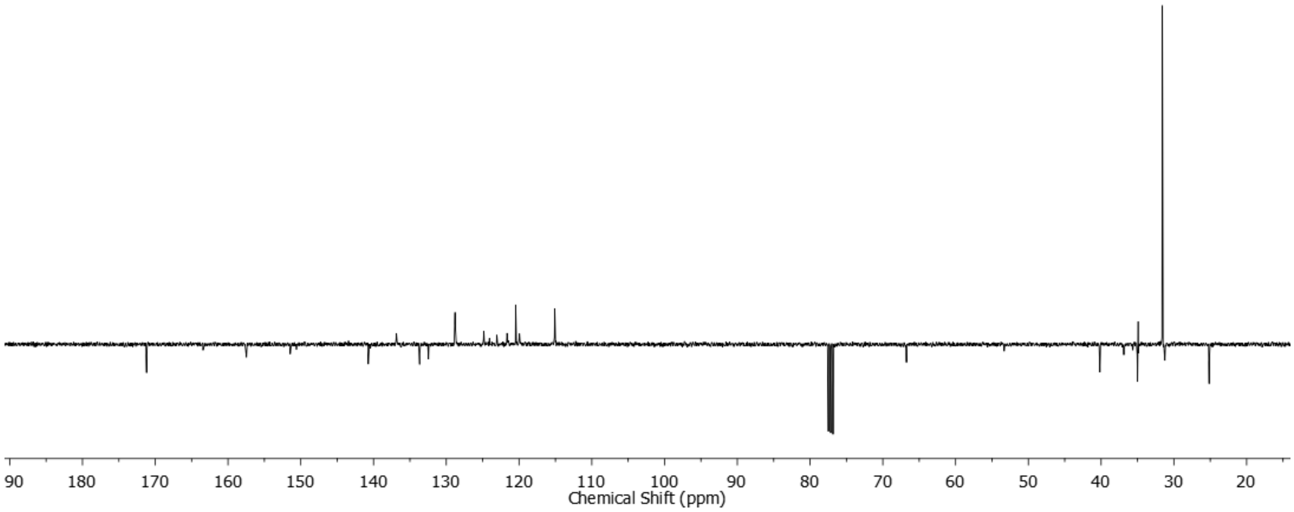

Figure S35 - ^{13}C-JMOD NMR (100 MHz, $CDCl_3$, 298 K) of **6**.

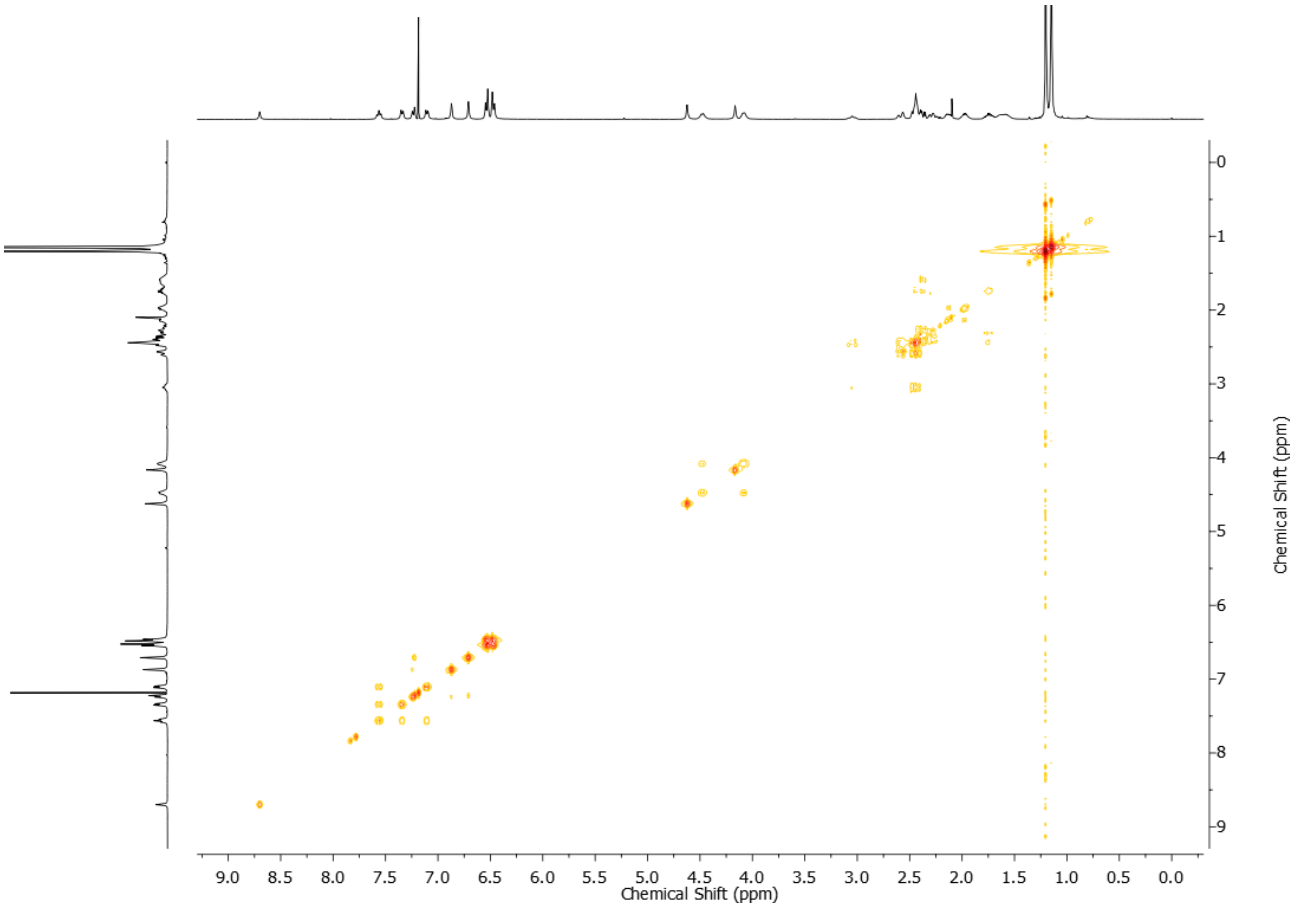

Figure S36 - ^{1}H COSY NMR (400 MHz, $CDCl_3$, 298 K) of **6**.

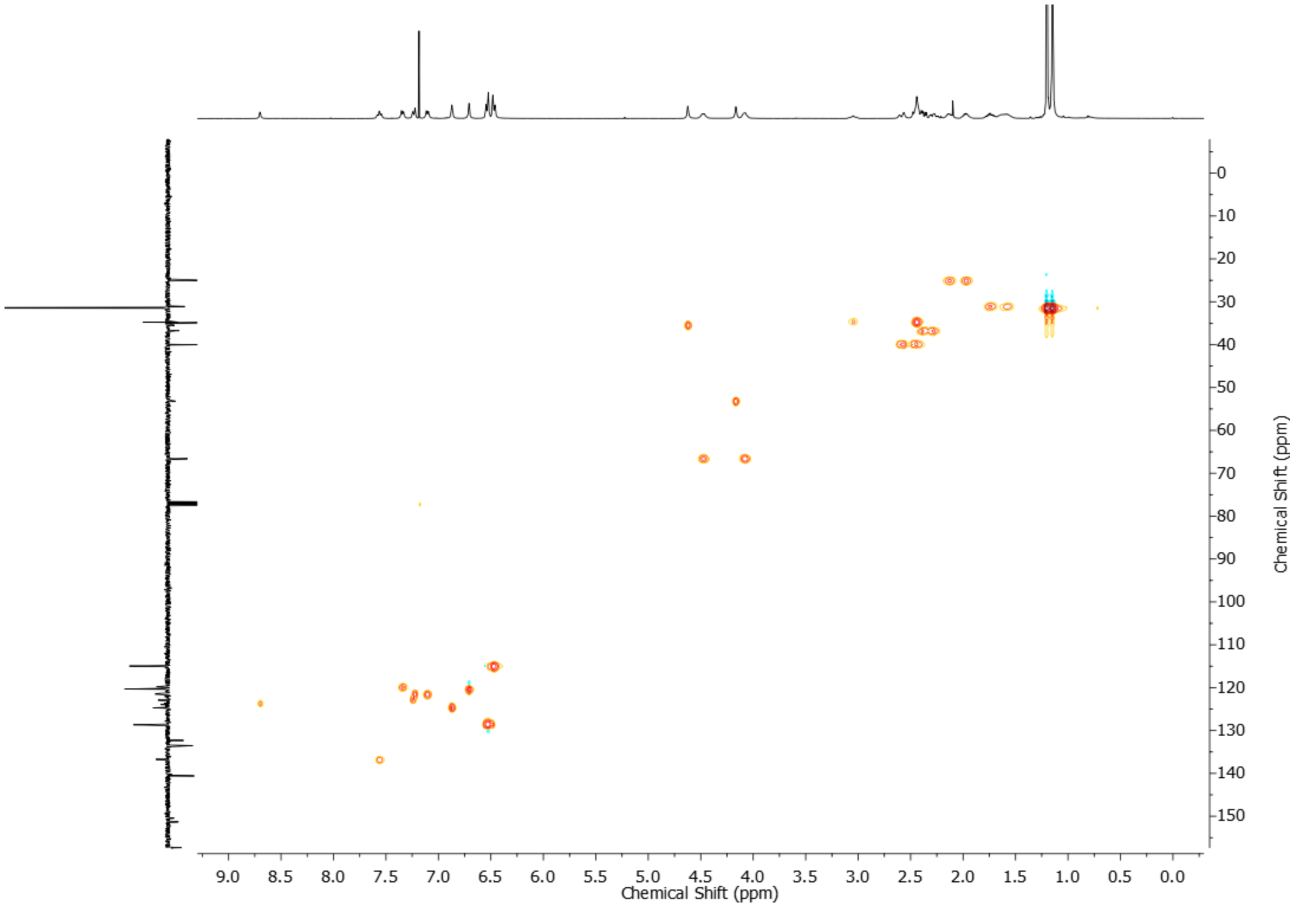

Figure S37 - ^{1}H-^{13}C HSQC NMR (400 MHz, $CDCl_3$, 298 K) of **6**.

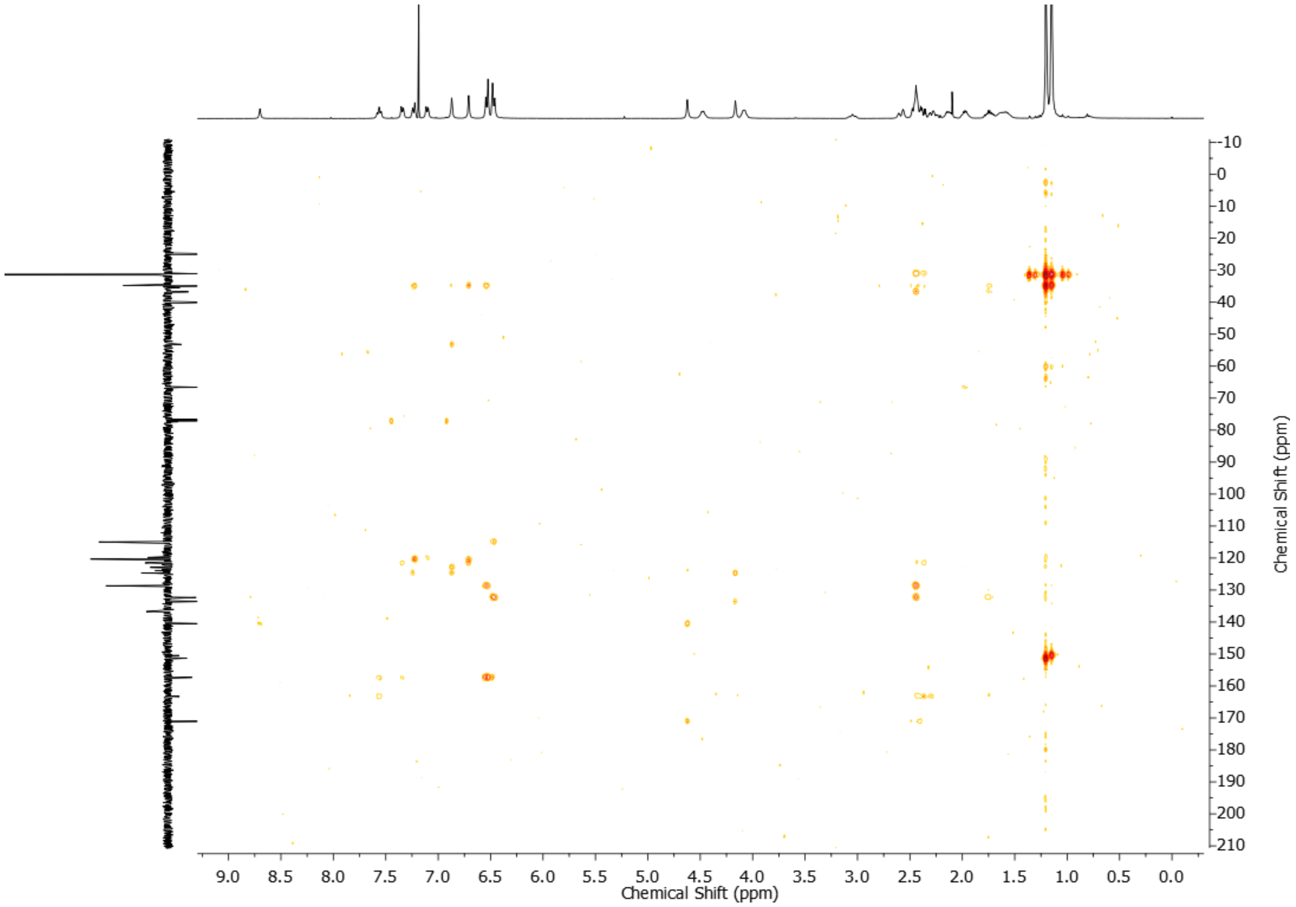

Figure S38 - ^{1}H-^{13}C HMBC NMR (400 MHz, $CDCl_3$, 298 K) of **6**.

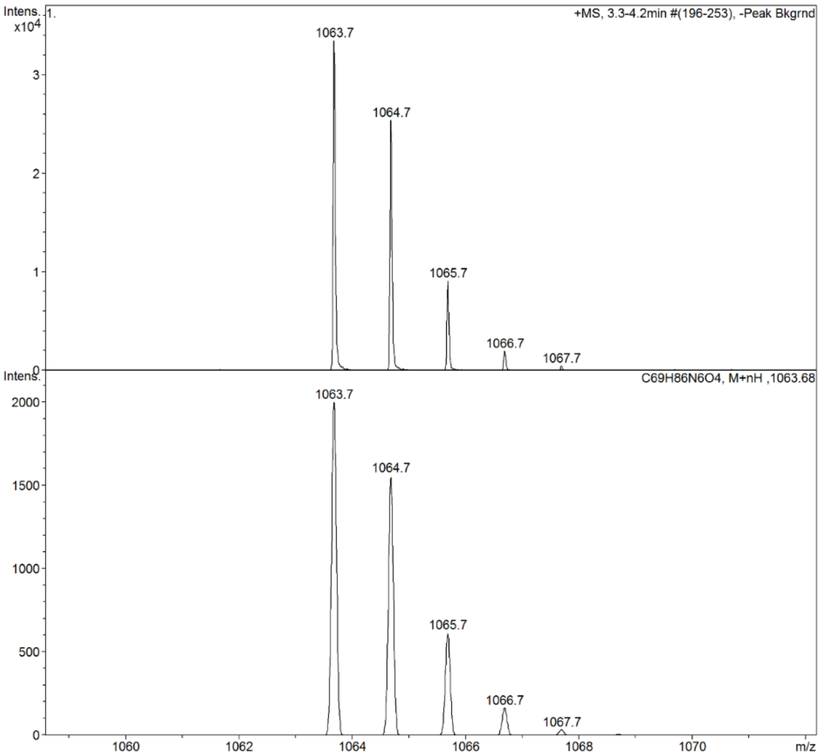

Figure S39 - Observed (top) and calculated (bottom) isotopic patterns for **6**.

Rotaxane 13 and related compounds

Axle of 13 (S6)

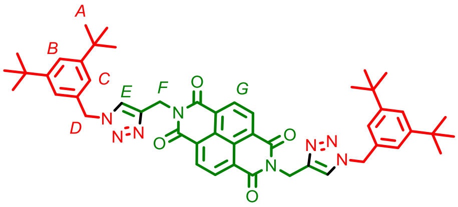

To a CEM vial charged with bispropargyl NDI **S5** (100 mg, 0.29 mmol, 1.0 eq) and azide **S1** (157 mg, 0.64 mmol, 2.2 eq) was added $[Cu(MeCN)_4]PF_6$ (44.7 mg, 0.12 mmol, 0.4 eq). The solids were then dissolved in CH_2Cl_2 (1.45 mL) before addition of DIPEA (257 µL, 1.45 mmol, 5.0 eq). After 16 h the solution was washed with a saturated EDTA/NH_3 solution, the organics were dried with $MgSO_4$ and concentrated *in vacuo*. Purification by column chromatography (0 to 20% EtOH in CH_2Cl_2) yielded axle as a yellow foam (219 mg, 0.26 mmol, 91%).^{1}H NMR (400 MHz, $CDCl_3$) δ 8.72 (s, 4H, H_G), 7.62 (s, 2H, H_E), 7.38 (t, *J* = 1.8, 2H, H_B), 7.06 (d, *J* = 1.8, 4H, H_C), 5.54 – 5.40 (m, 8H, H_D), 1.26 (s, 36H, H_A). ^{13}C NMR (101 MHz, $CDCl_3$) δ 162.5, 151.8, 143.1, 133.8, 131.2, 126.8, 126.7, 123.5, 122.8, 122.5, 54.9, 35.7, 35.0, 31.5. HR-ESI-MS (+ve) m/z = 833.4483 $[M+H]^+$ (calc. m/z for $C_{50}H_{57}N_8O_4$ 833.4497).

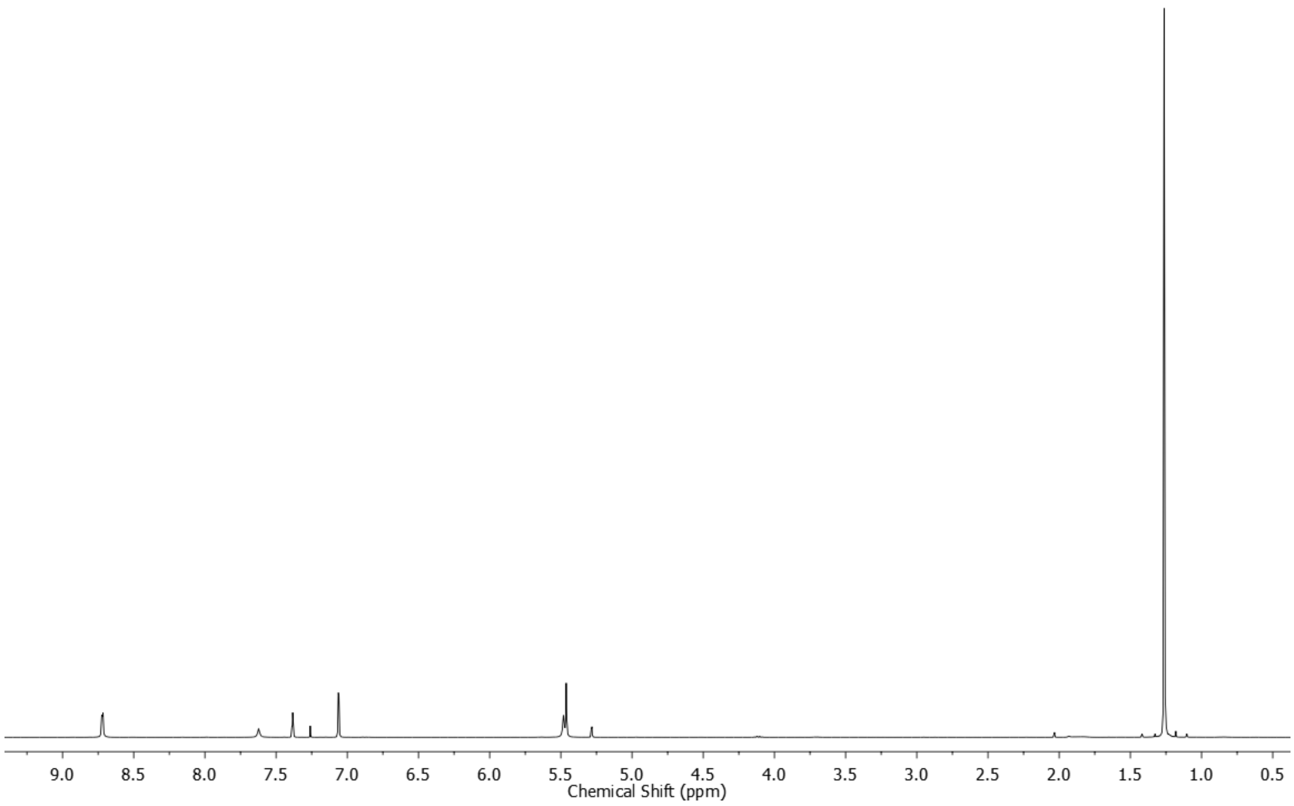

Figure S40 - ^{1}H NMR (400 MHz, $CDCl_3$, 298 K) of axle **S6**.

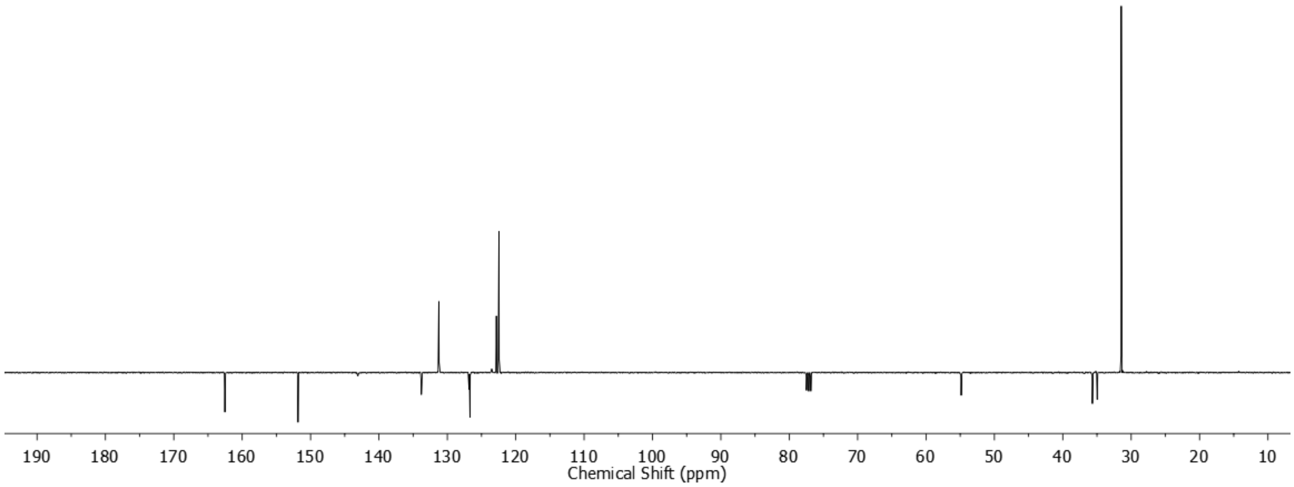

Figure S41 - ^{13}C-JMOD NMR (100 MHz, $CDCl_3$, 298 K) of [3]rotaxane axle **S6**.

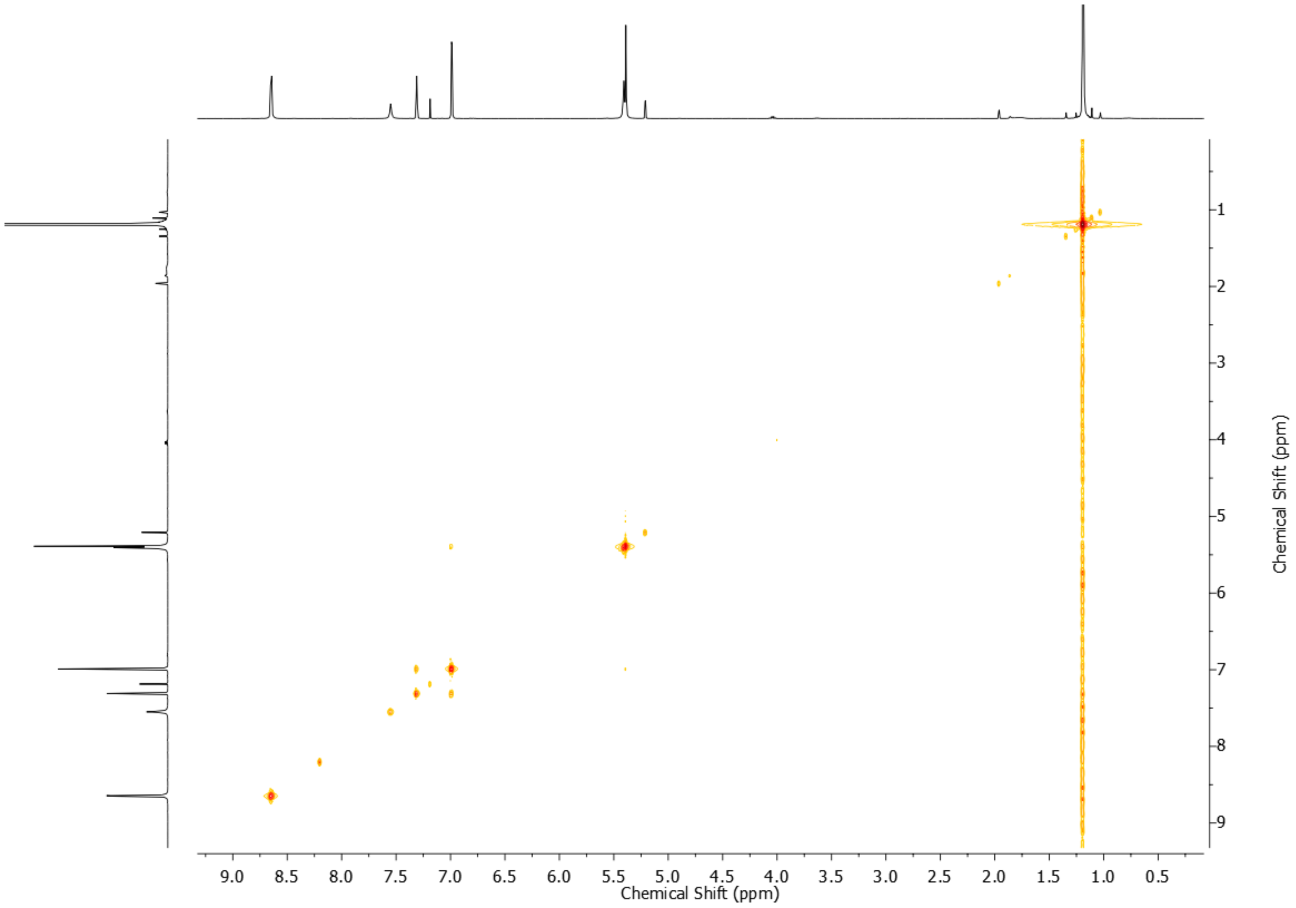

Figure S42 - ^{1}H COSY NMR (400 MHz, $CDCl_3$, 298 K) of axle **S6**.

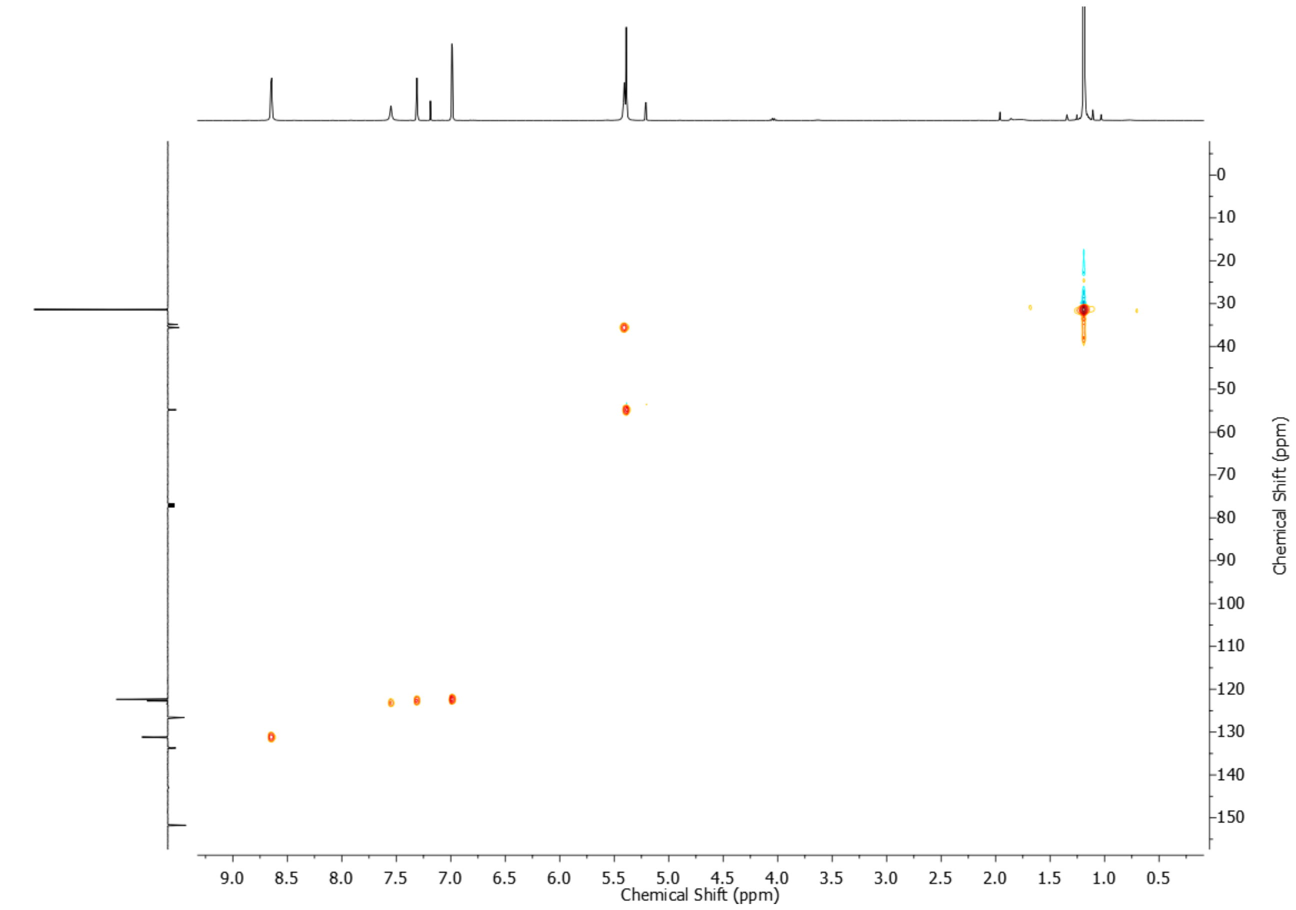

Figure S43 - ^{1}H-^{13}C HSQC NMR (400 MHz, $CDCl_3$, 298 K) of axle **S6**.

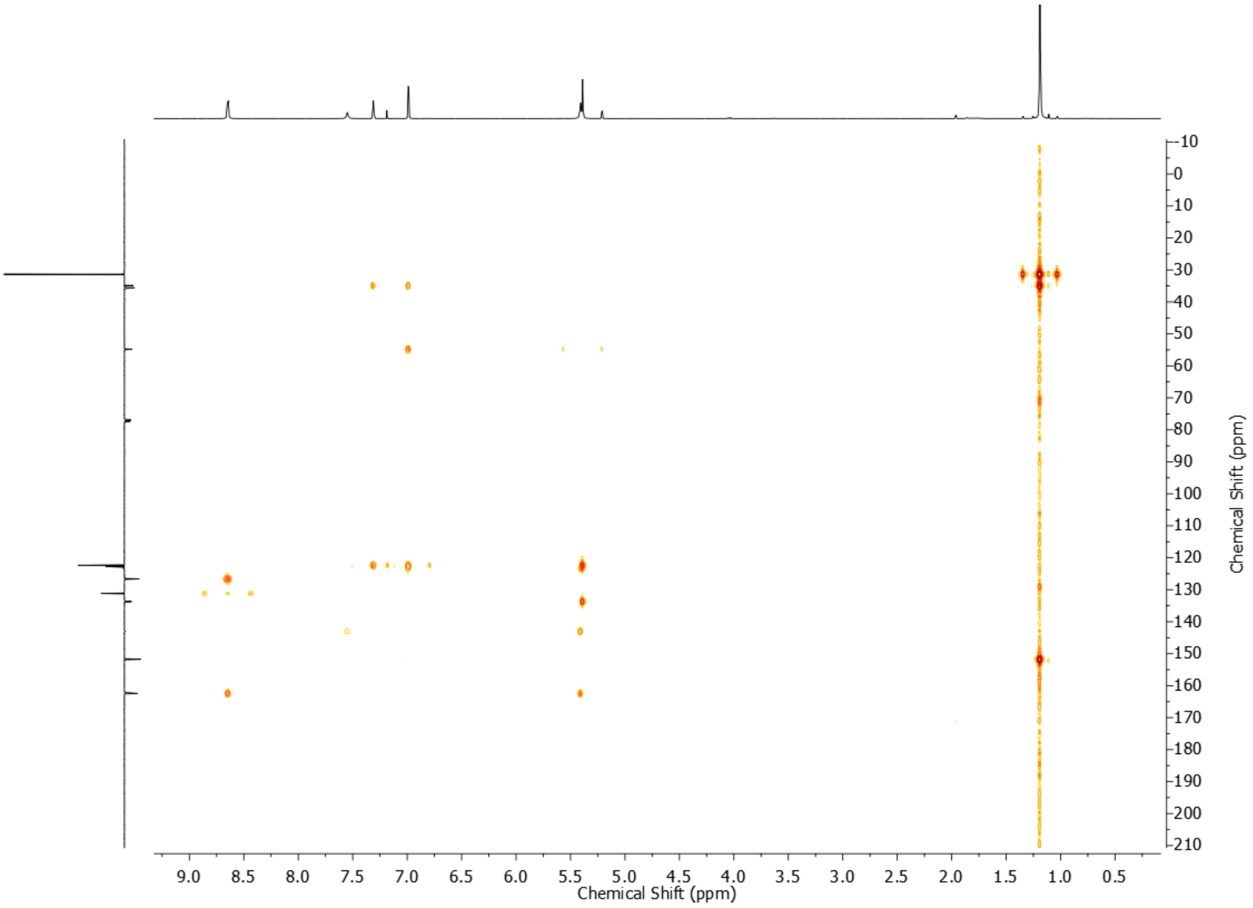

Figure S44 - ^{1}H-^{13}C HMBC NMR (400 MHz, $CDCl_3$, 298 K) of axle **S6**.

Rotaxane 13

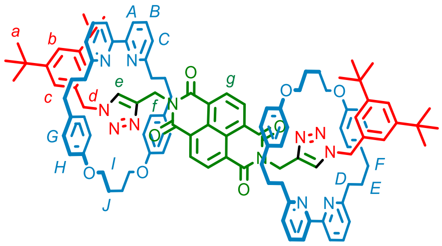

In a CEM vial, bispropargyl NDI **S5** (38 mg, 0.11 mmol, 1.0 eq), bipyridine macrocycle **1** (105.9 mg, 0.22 mmol, 2.0 eq), azide **S1** (60 mg, 0.24 mmol, 2.2 eq) and $[Cu(MeCN)_4]PF_6$ (80.6 mg, 0.22 mmol,1.95 eq) were dissolved in CH_2Cl_2 (2.8 mL). DIPEA (97 µL, 0.56 mmol, 5.0 eq) was added and the reaction was left for 16 h, after which TFA (34 µL, 0.44 mmol, 4 eq) was added. After 16 h, the reaction mixture was diluted with CH_2Cl_2 and washed with a saturated EDTA/NH_3 solution, water and brine. The organic layers were dried with $MgSO_4$, filtered and concentrated. Column chromatography (0 to 50% MeCN in CH_2Cl_2, then 10% MeOH in CH_2Cl_2) gave **13** as a yellow foam (140 mg, 0.078 mmol, 70%). ^{1}H NMR (400 MHz, $CDCl_3$) δ 9.20 (s, 1H, H_e), 8.19 (s, 2H, H_g), 7.46 (t, J = 7.8, 2H, H_B), 7.27 (t, J = 1.9, 1H, H_b), 7.14 (d, J = 7.8, 2H, H_A), 6.97 – 6.90 (m, 4H, H_C, H_c), 6.63 (d, J = 8.7, 4H, H_G), 6.58 (d, J = 8.7, 4H, H_H), 5.01 (s, 2H, H_f), 4.53 – 4.39 (m, 4H, H_d, H_I), 4.30 – 4.16 (m, 2H, H_I), 2.64 – 2.52 (m, 2H, H_F), 2.50 – 2.39 (m, 2H, H_F), 2.34 – 2.00 (m, 8H, H_J, H_D), 1.88 – 1.73 (m, 2H, H_E), 1.73 – 1.59 (m, 2H, H_E), 1.15 (s, 18H, H_a). ^{13}C NMR (100 MHz, $CDCl_3$) 163.0, 162.1, 157.5, 156.4, 150.6, 140.3, 136.5, 133.6, 132.2, 130.0, 128.8, 126.4, 126.2, 125.1, 124.5, 123.1, 121.3, 119.1, 115.0, 66.6, 53.6, 36.6, 36.5, 35.2, 34.8, 31.5, 30.8, 25.1. LR-ESI-MS (ESI) m/z = 896.0 $[M+2H]^{2+}$.

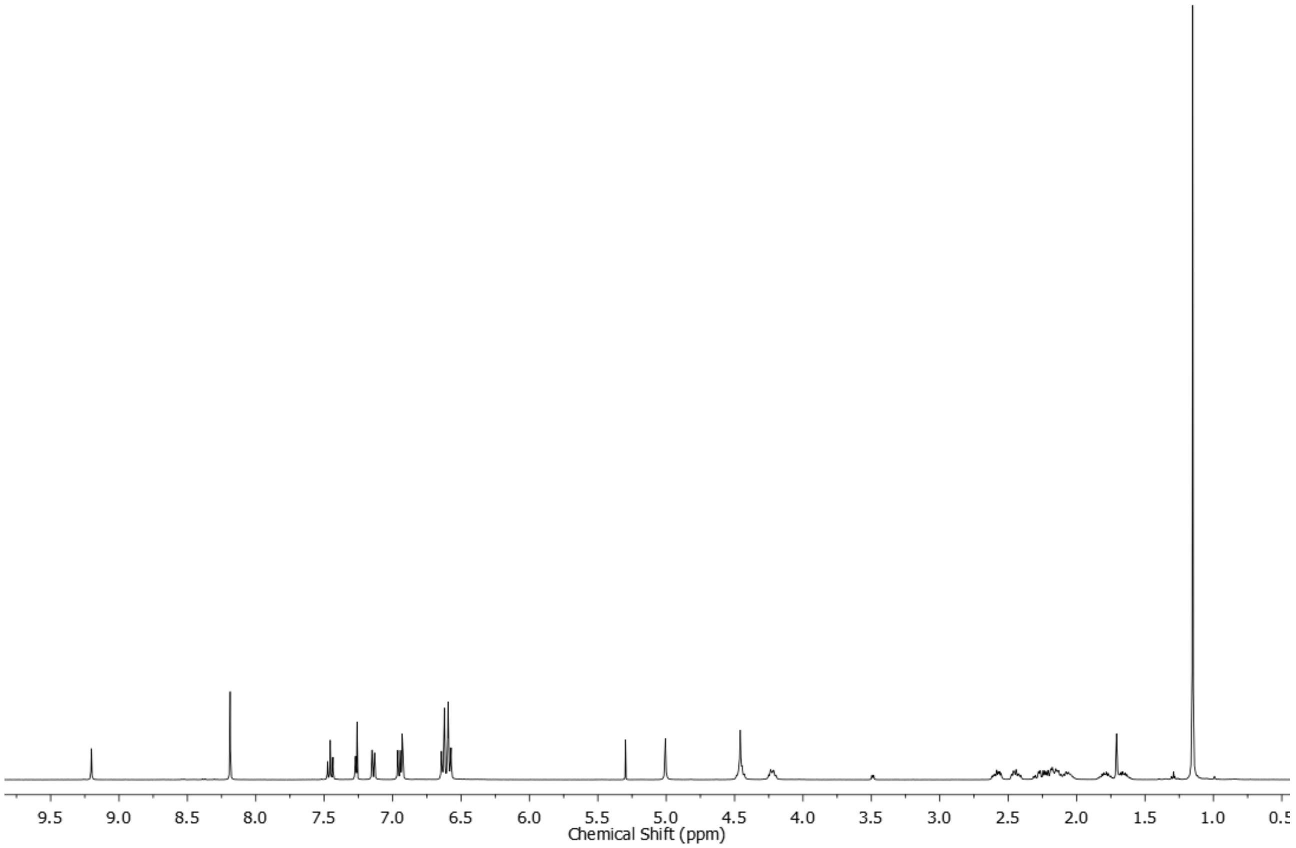

Figure S45 - ^{1}H NMR (400 MHz, $CDCl_3$, 298 K) of **13**.

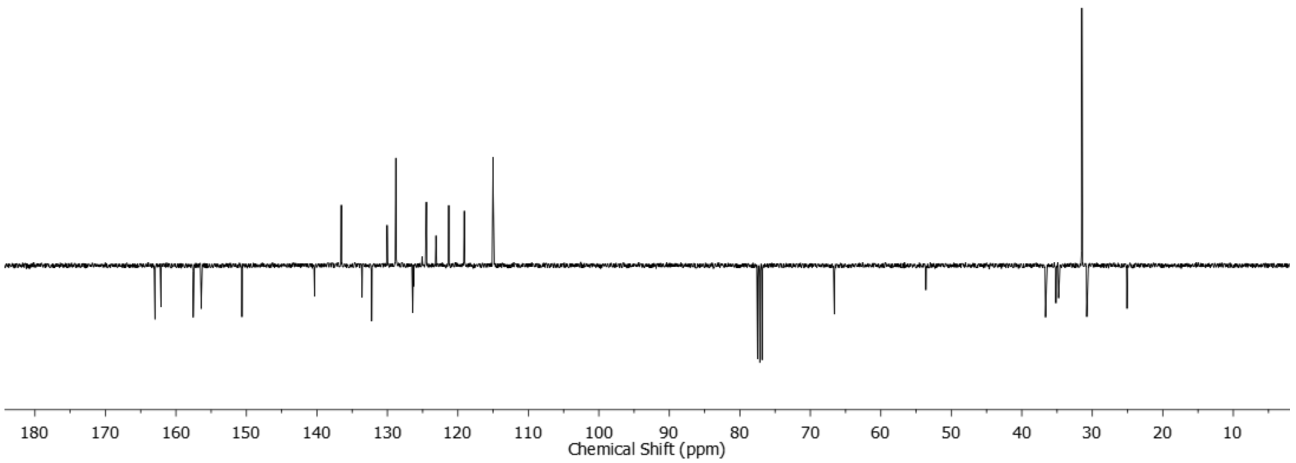

Figure S46 - JMOD NMR (100 MHz, $CDCl_3$, 298 K) of **13**.

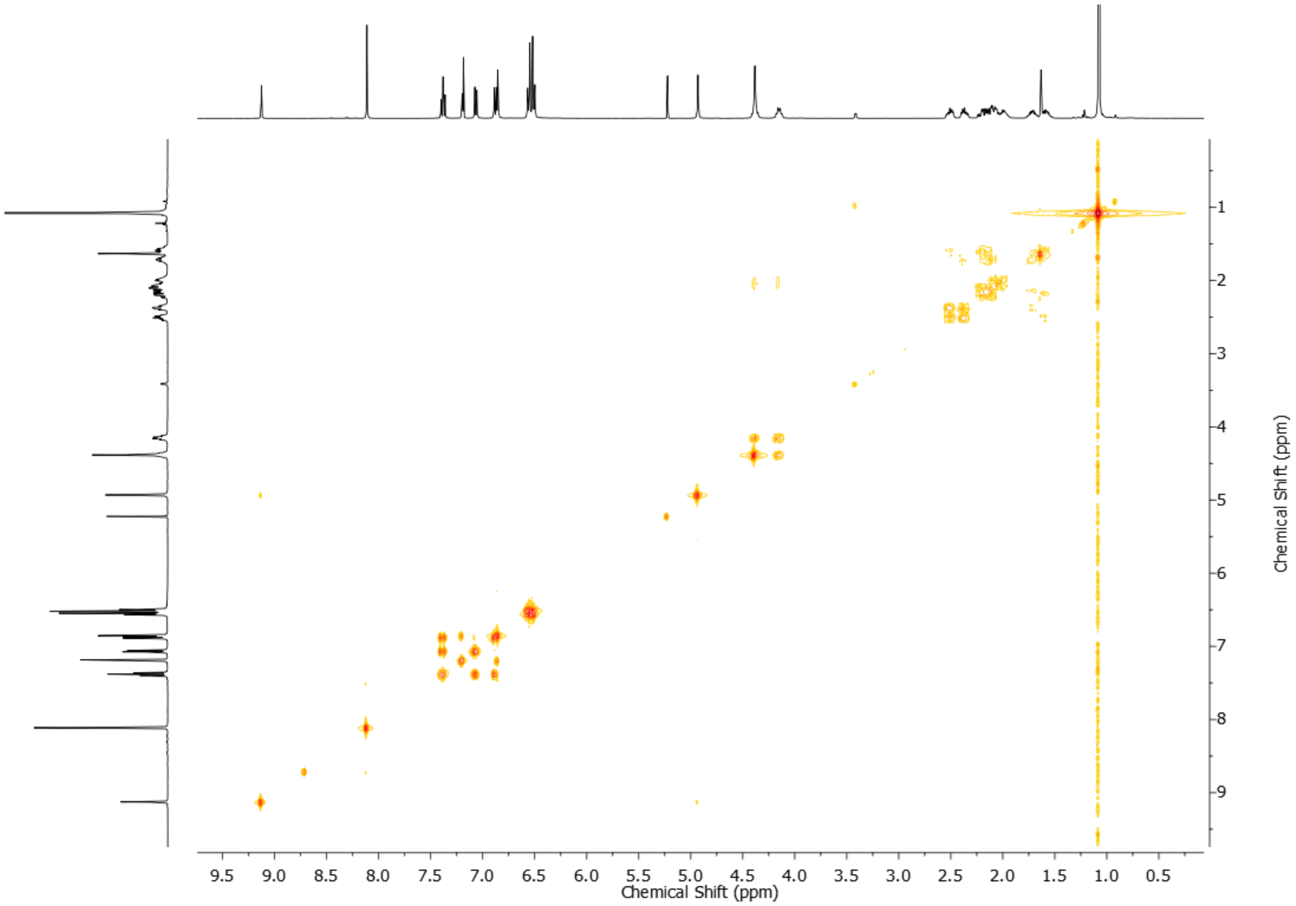

Figure S47 - ^{1}H COSY NMR (400 MHz, $CDCl_3$, 298 K) of **13**.

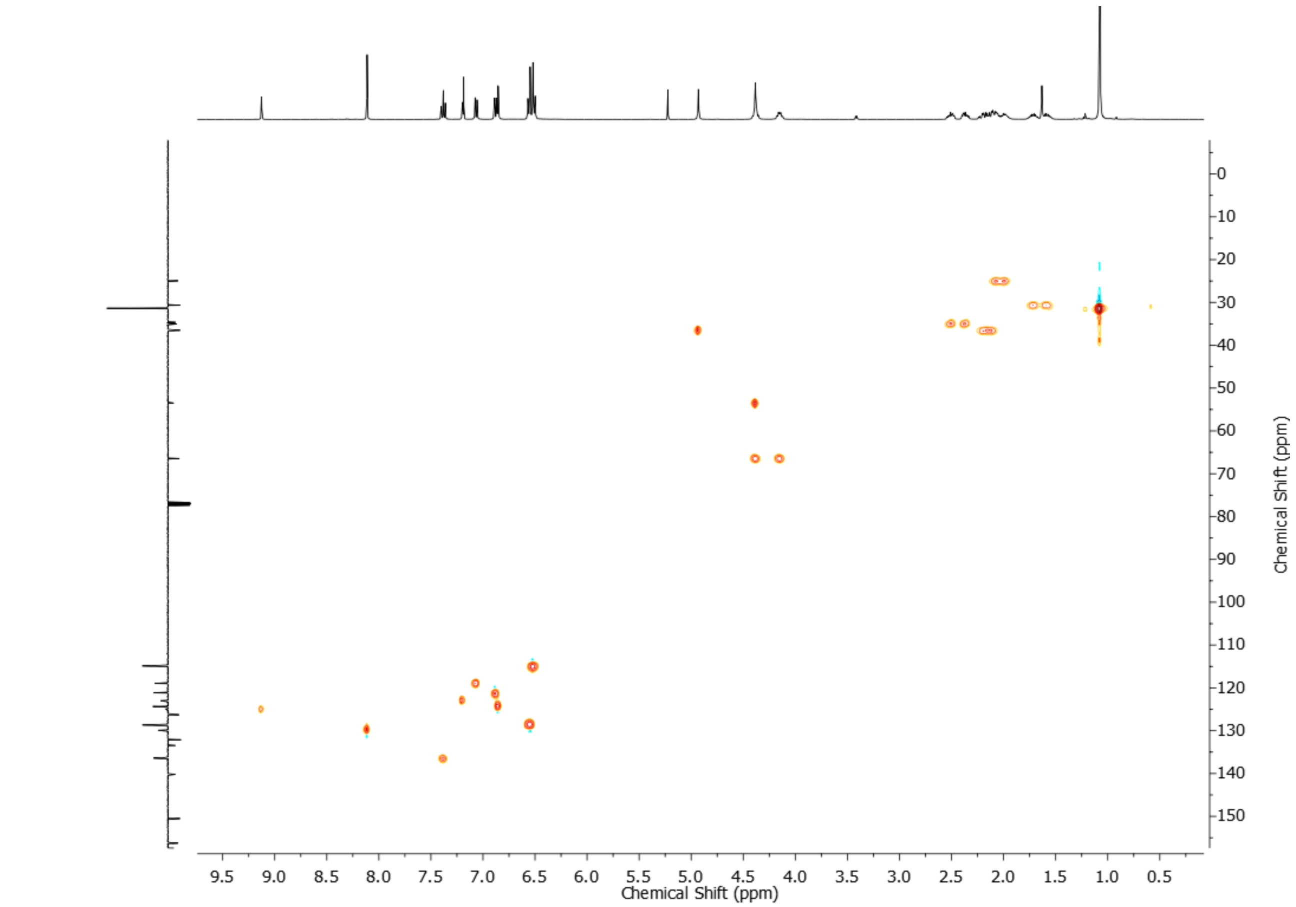

Figure S48 - ^{1}H-^{13}C HSQC NMR (400 MHz, $CDCl_3$, 298 K) of **13**.

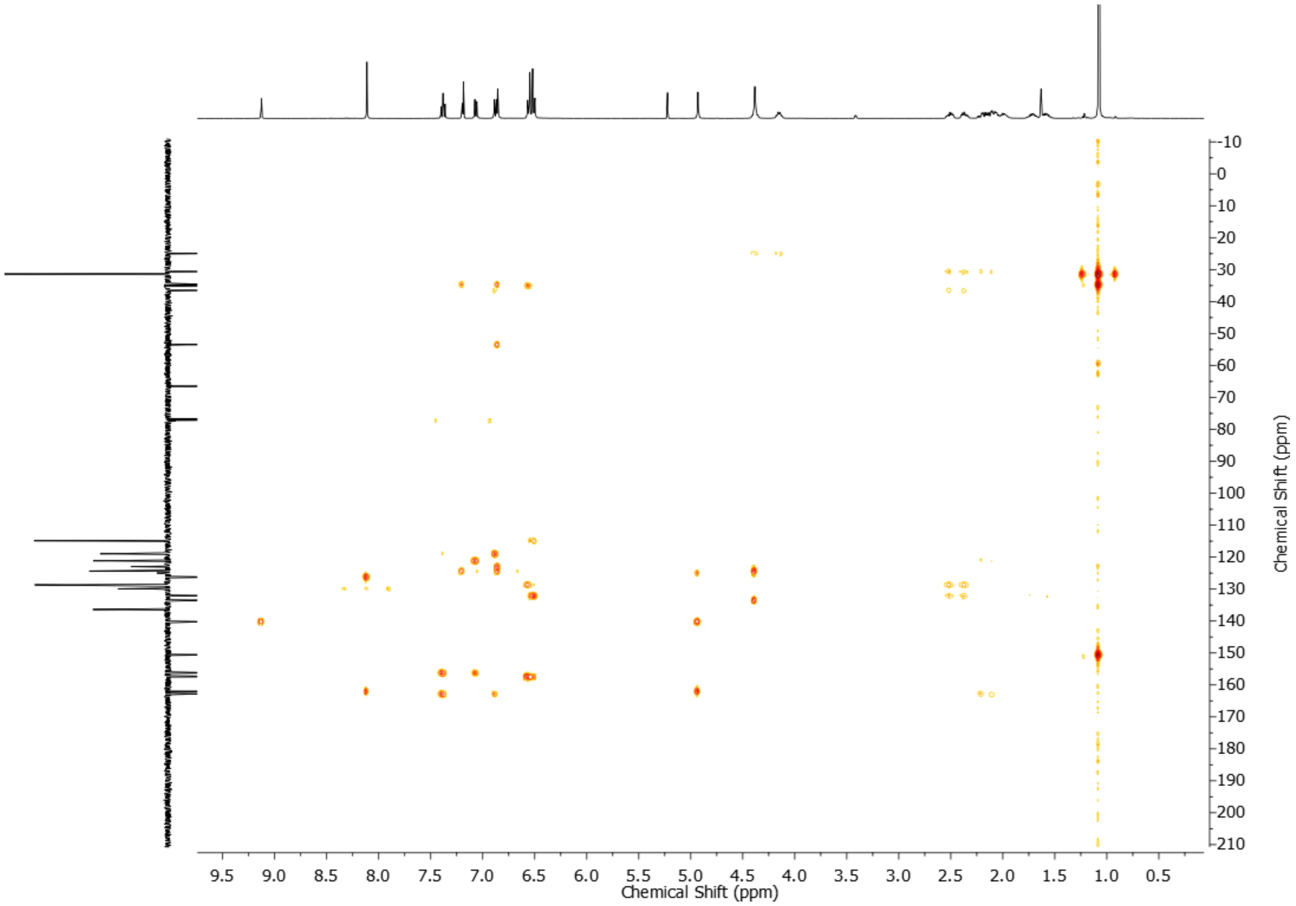

Figure S49 - ^{1}H-^{13}C HMBC NMR (400 MHz, $CDCl_3$, 298 K) of **13**.

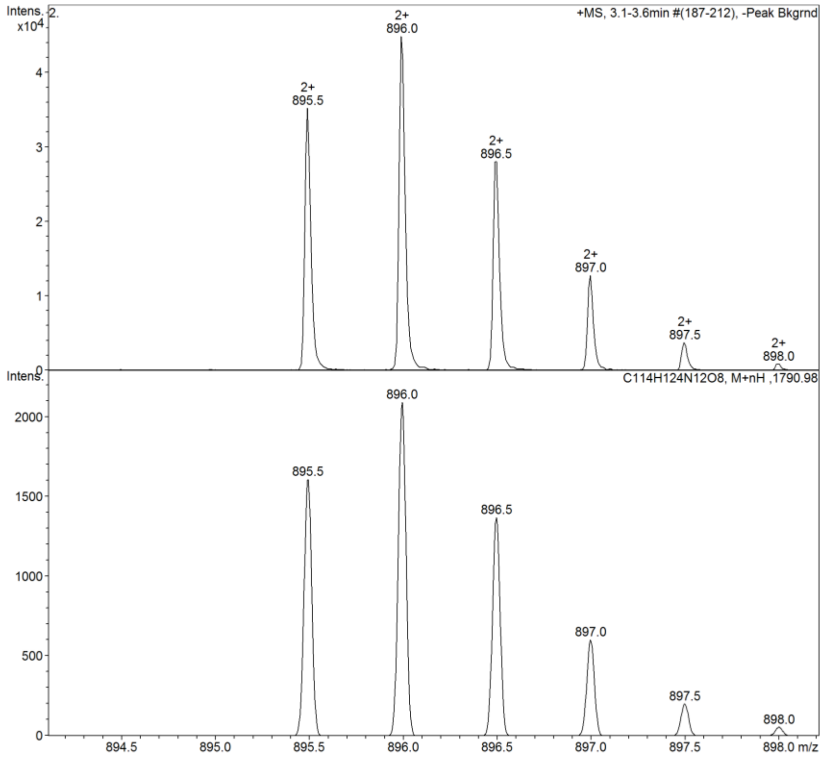

Figure S50 - Observed (top) and calculated (bottom) isotopic patterns for **13**.

S3. X-RAY ANALYSIS OF 4A AND 4AH$^+$

Single crystal X-ray crystallographic data for 4a

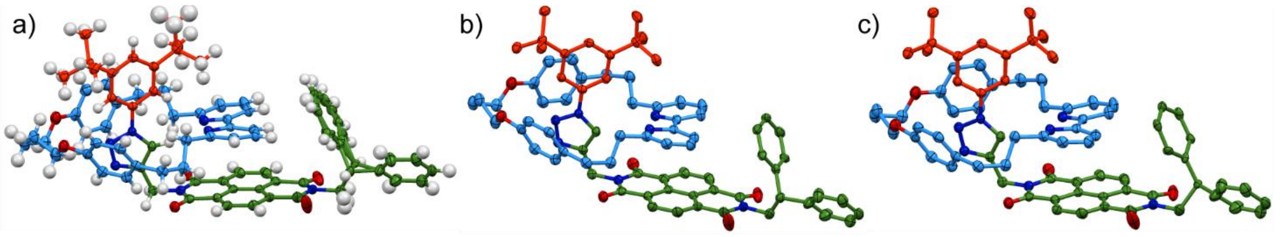

Figure S51 - Ellipsoid plot of the asymmetric unit of 4a. (a) side view showing disordered diphenyl stoppering unit, (b) side view with H-atoms omitted of one stoppering unit orientation, and (c) side view with H-atoms omitted of the other stoppering unit orientation. Ellipsoids are shown at the 50% probability level.

Single crystals of **4a** were grown by slow evaporation of MeOH-CH_2Cl_2. Data was collected at 100 K using a Rigaku 007 HF diffractometer equipped with a HyPix 6000HE hybrid pixel array detector. Cell determination, data collection, data reduction, cell refinement and absorption correction were performed with CrysalisPro[18]. Using Olex2[19] the structure was solved with the SHELXT[20] program using dual methods and refined with the SHELXL refinement package. H atoms were placed in calculated positions and refined using a riding model.

Table S1 - Crystal data and structure refinement for **4a**

Identification code	[2]Ph-E4-NDI
Empirical formula	$C_{77}H_{75}N_7O_6$
Formula weight	1194.44
Temperature/K	100
Crystal system	monoclinic
Space group	$P2_1/c$
a/Å	26.9977(3)
b/Å	13.30610(10)
c/Å	17.8430(2)
α/°	90
β/°	101.8010(10)
γ/°	90
Volume/Å^3	6274.33(11)
Z	4
ρ_{calc}g/cm^3	1.264
μ/mm^{-1}	0.638
F(000)	2536.0
Crystal size/mm^3	0.1 × 0.1 × 0.1
Radiation	CuKα (λ = 1.54184)
2Θ range for data collection/°	7.438 to 129.286
Index ranges	$-30 \le h \le 31, -15 \le k \le 15, -20 \le l \le 19$
Reflections collected	59346
Independent reflections	10505 [R_{int} = 0.0442, R_{sigma} = 0.0361]
Data/restraints/parameters	10505/0/821
Goodness-of-fit on F^2	1.053
Final R indexes [I>=2σ (I)]	R_1 = 0.0580, wR_2 = 0.1368
Final R indexes [all data]	R_1 = 0.0644, wR_2 = 0.1408

Single crystal X-ray crystallographic data for 4aH⁺

Single crystals of **4a** were grown by slow evaporation of a catalysis experiment with 20mol% **4a,** 1,2-cyclohexandione, *E*-nitrovinylbenzene, and $TBAPF_6$ in 1:1 $CDCl_3$-C_6F_6. Data was collected at 100 K using a Rigaku 007 HF diffractometer equipped with a HyPix 6000HE hybrid pixel array detector. Cell determination, data collection, data reduction, cell refinement and absorption correction were performed with CrysalisPro[18]. Using Olex2[19] the structure was solved with the SHELXT[20] program using dual methods and refined with the SHELXL refinement package. H atoms were placed in calculated positions and refined using a riding model.

Table S2 - Crystal data and structure refinement for **4a·HPF$_6$**

Identification code	JMIV026-GJTz
Empirical formula	$C_{78}H_{77}Cl_3F_6N_7O_6P$
Formula weight	1459.78
Temperature/K	100.00(10)
Crystal system	triclinic
Space group	P-1
a/Å	12.9744(2)
b/Å	14.1072(2)
c/Å	22.0084(3)
α/°	78.0800(10)
β/°	76.0050(10)
γ/°	81.2180(10)
Volume/Å^3	3801.95(10)
Z	2
ρ_{calc}g/cm^3	1.275
μ/mm^{-1}	0.212
F(000)	1524.0
Crystal size/mm^3	0.27 × 0.16 × 0.04
Radiation	Mo Kα (λ = 0.71075)
2Θ range for data collection/°	2.968 to 61.018
Index ranges	-18 ≤ h ≤ 18, -20 ≤ k ≤ 20, -31 ≤ l ≤ 31
Reflections collected	116560
Independent reflections	23150 [R_{int} = 0.0341, R_{sigma} = 0.0282]
Data/restraints/parameters	23150/3/947
Goodness-of-fit on F^2	1.058
Final R indexes [I>=2σ (I)]	R_1 = 0.0689, wR_2 = 0.1895
Final R indexes [all data]	R_1 = 0.0894, wR_2 = 0.2074

S4. CATALYTIC EXPERIMENTS

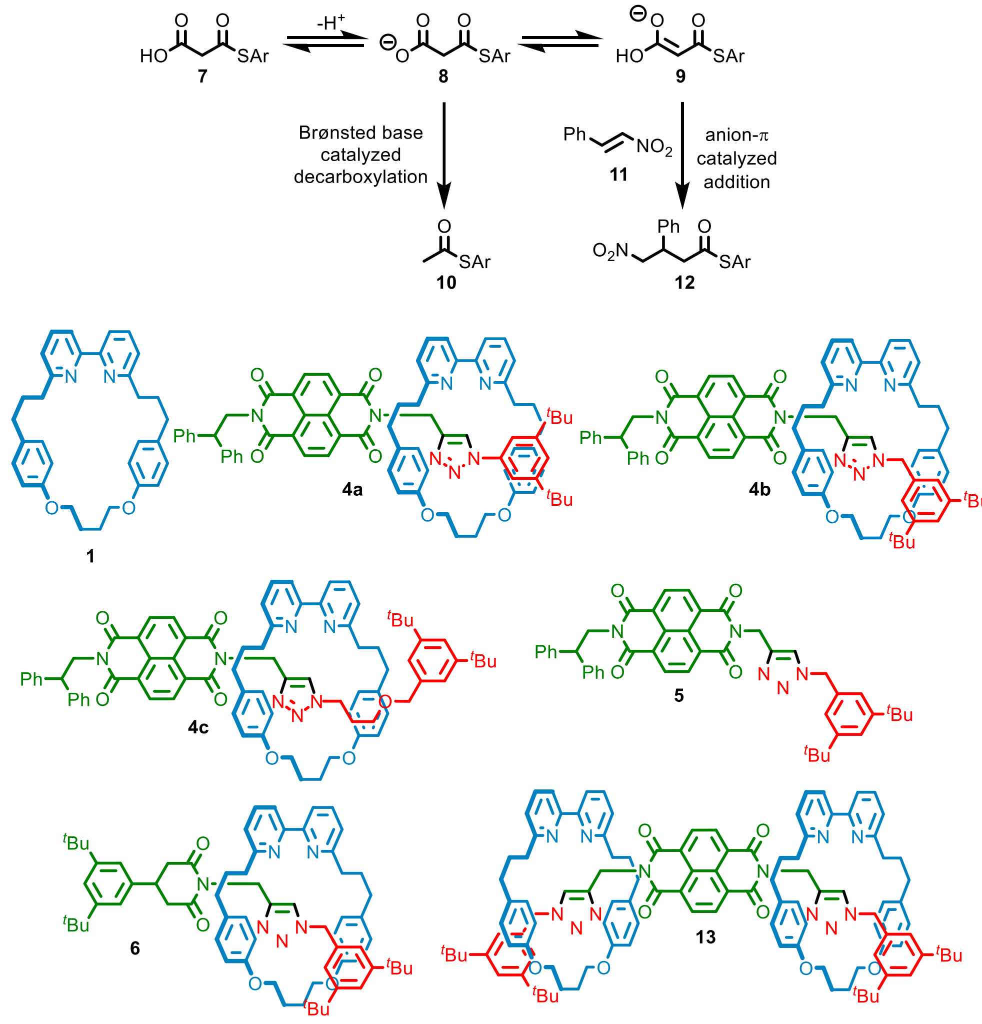

Scheme S1 - Catalytic reaction showing the catalysts examined

For catalyst benchmarking, the catalyst (40 mM, 0.2 eq) was added to a solution of malonic acid half thioester **7** (0.2 M, 1.0 eq.) and *E*-nitrovinylbenzene (2.0 M, 10.0 eq.) in a sealed vial. Reaction progress was monitored by removal of 20 µL aliquots and dilution to 600 µL with $CDCl_3$. Selectivity was assessed at >95% conversion by integration of signals corresponding to decarboxylation –OMe (H_D, 2.39 ppm) and addition product Hα (H_A, 3.08 ppm) after peak fitting to correct for overlapping signals using the default algorithm in MNova,[7] and inputting into the formula below:

$$\mathbf{12/10} = \frac{3H_A}{2H_D}$$

The selectivity of the benchmark MAHT reaction shows strong temperature dependence (e.g. **13** = 18.6 *vs* 62.3 at 30 °C and 7 °C respectively). Thus, during the optimisation of our procedure, we noted variation in the observed selectivity for a given catalyst when reactions were run under ambient conditions on different days. However, it should be noted that when reactions with different catalysts (e.g. **4a**-**c**) were run in parallel (i.e. identical ambient conditions) the relative performance of catalysts was always consistent (e.g. selectivity: **13** > **4b** > **4a** > **4c**, activity **13** > **4b** > **4a** ~ **4c**). When the reaction temperature was controlled, the outcome of reactions run in triplicate were consistent (**12/10** = ±0.1). In addition, all comparative studies were performed in a single batch (i.e. concurrently in the same heating bath), ensuring that all catalyst comparisons are like-for-like.

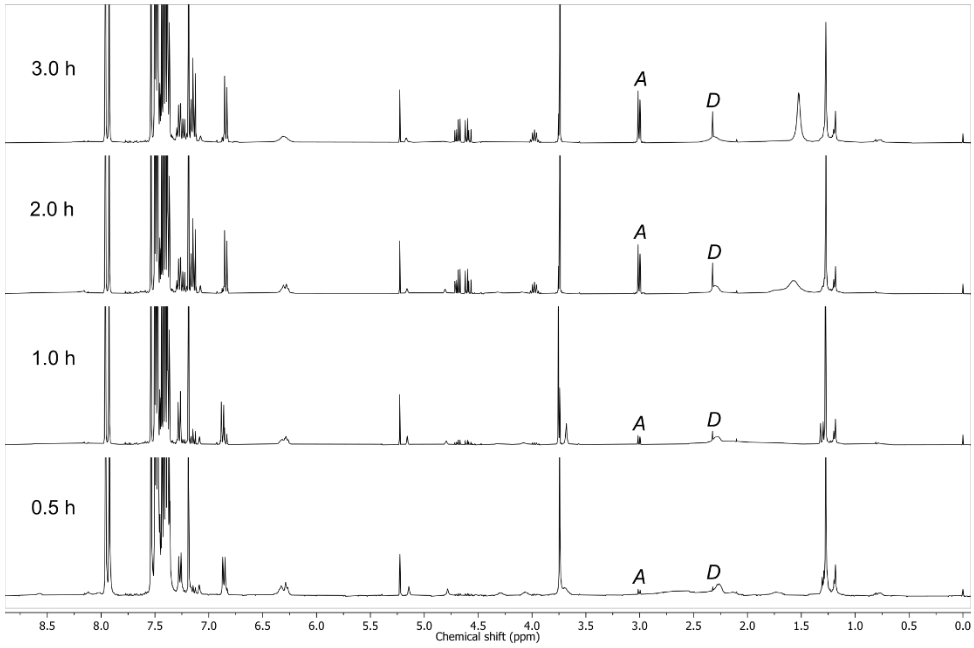

Figure S52 - Illustrative catalytic reaction with 20 mol% rotaxane **13** In $CDCl_3$ at 30 °C. Characteristic signals corresponding to addition (*A*) and decarboxylation (D) products used to calculate catalyst chemoselectivity and conversion indicated.

Table S3 - Addition/decarboxylation ratios obtained with rotaxane anion-π catalysts and controls in benchmarking Michael addition reaction.

Catalyst	Solvent	Temperature (°C)	**12/10**
**4a**	THF-d_8	30	-[a]
**4b**	THF-d_8	30	4.4
**4c**	THF-d_8	30	-[a]
**1**	THF-d_8	30	-[a]
**5**	THF-d_8	30	n.r.[b]
**1** & **5** (1:1)	THF-d_8	30	-[a]
NEt_3	THF-d_8	30	0.4
**6**	THF-d_8	30	2.4
**13**	THF-d_8	30	5.9
**4a**	$CDCl_3$	30	10.7
**4b**	$CDCl_3$	30	11.9
**4c**	$CDCl_3$	30	10.3
**1**	$CDCl_3$	30	7.1
**5**	$CDCl_3$	30	n.r.[b]
**1** & **5** (1:1)	$CDCl_3$	30	7.2
NEt_3	$CDCl_3$	30	3.2
**6**	$CDCl_3$	30	8.0
**13**	$CDCl_3$	30	18.6
**4b**	$CDCl_3$	7	46.2
**13**	$CDCl_3$	7	62.3
**6**	$CDCl_3$	7	33.3
NEt_3	$CDCl_3$	7	8.7

[a]Less than 10% conversion was observed, which prevented accurate measurement of A/D. [b]No reaction was observed.

S5. Comparison between 4b, 13 and previously reported anion-π catalysts

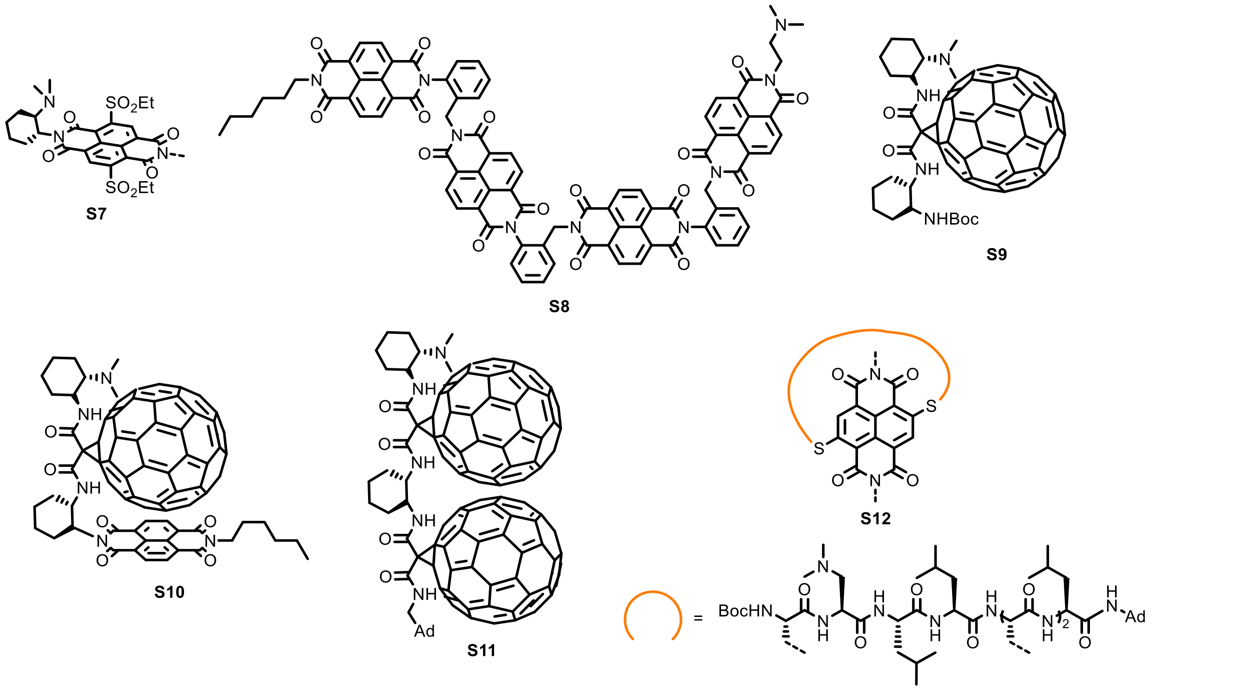

Figure S53 - Previous examples of anion-π catalysts applied to the benchmarking reaction used to assess performance of rotaxane catalysts.

Table S4 - Comparison of addition/decarboxylation ratios obtained with rotaxane catalysts **4b** and **13** against those obtained with other anion-π catalysts shown above in Figure S53.

Entry	Catalyst	Solvent	Temperature (°C)	A/D ratio
1	**4b**	THF-d_8	30	4.4
2	**13**	THF-d_8	30	5.9
3	**4b**	$CDCl_3$	30	11.9
4	**13**	$CDCl_3$	30	18.6
5	**4b**	$CDCl_3$	7	46.2
6	**13**	$CDCl_3$	7	62.3
7	**S7**[8]	THF-d_8	20	4.4
8	**S7**[8]	THF-d_8	7	9.6
9	**S8**[9]	THF-d_8/$CDCl_3$ 3:1	20	10.4
10	**S9**[10]	THF-d_8/$CDCl_3$ 1:1	20	8.2
11	**S10**[10]	THF-d_8/$CDCl_3$ 1:1	20	9.2
12	**S11**[10]	THF-d_8/$CDCl_3$ 1:1	20	22.5
13	**S12**[11]	CD_2Cl_2	20	63

S6. NMR ANALYSIS OF ROTAXANES 4 AND THEIR RELATIVE BASICITY

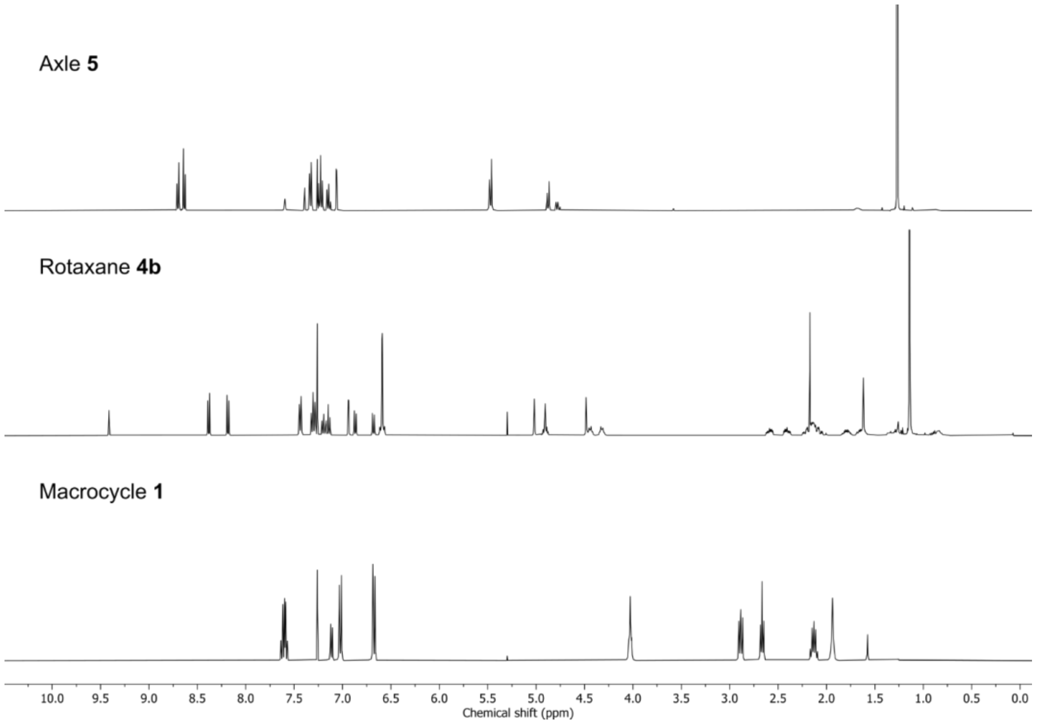

Figure S54 - Stacked ^{1}H-NMR spectra (400 MHz, $CDCl_3$, 298 K) of macrocycle **1**, rotaxane **4b** and related axle **5** revealing significant shifts in the interlocked compound.

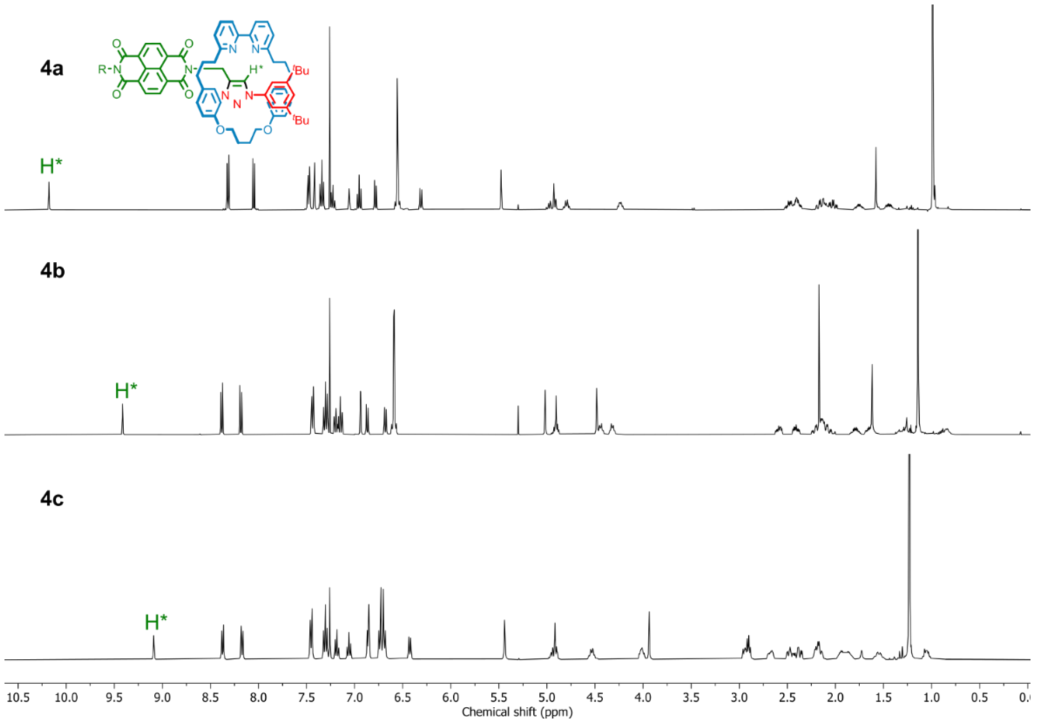

Figure S55 - Stacked ^{1}H-NMR spectra (400 MHz, $CDCl_3$, 298 K) of rotaxanes **4a-c** with increasing lengths of axle. The signals corresponding to the triazole protons in the interlocked products have been labelled to highlight the relationship between the triazole chemical shift and axle length.

Significant differences were observed in the rates of conversion in the benchmarking Michael addition for our series of [2]rotaxane catalysts **4a**-**c** with differing axle lengths, and it was hypothesised that this was as a result of differing basicities. To qualitiatively assess the differences in basicity between our series of [2]rotaxanes, **4a-c** were combined in approximately 1:1:1 ratio and dissolved in $CDCl_3$, MsOH was added, and the ^{1}H-NMR spectrum of the resulting mixture of protonated/neutral rotaxanes was acquired. The ^{1}H-NMR spectra are shown below in Figure S56, cropped to show only the most informative regions of the spectra; i) the region containing the signals

corresponding to the neutral triazole CH and ii) the region containing signals corresponding to the tert-butyl substituents of the azide derived stoppering unit of both the neutral and protonated rotaxanes. In line with the observed trends in catalyst activity (**4b**>**4a**/**4c**), after the addition of 0.5 equivalents of MsOH to the mixture of rotaxanes, catalyst **4b** appears to be protonated to a greater extent than either **4a** or **4c**. After the addition of 1.5 equivalents of MsOH to the rotaxane mixture, catalyst **4b** appears to be completely protonated, whereas signals corresponding to neutral **4a** and **4c** still remain, suggesting that it is the most basic of the three. The positive correlation observed between basicity and catalytic rate would support the hypothesis that the observed differences in catalytic activity result from differing basicities.

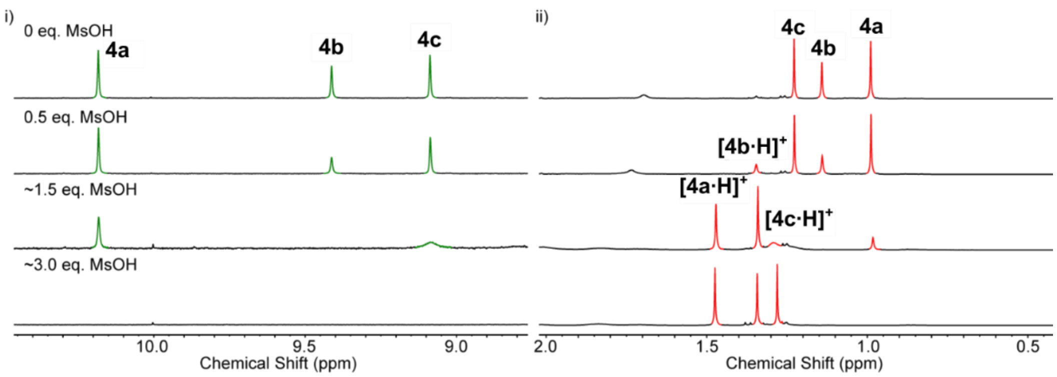

Figure S56 - Partial ^{1}H-NMR (400 MHz, $CDCl_3$, 298 K) of an approximately 1:1:1 mixture of [2]rotaxanes **4a**-**c**, with MsOH added as a solution in CDCl3, in the regions i) 10.5-9.0 ppm, showing triazole protons of neutral rotaxanes, and ii) 2.0-0.5 ppm, showing *t*Bu signals for both protonated and neutral [2]rotaxanes.

S7. COMPUTATIONAL RESULTS

S7.1 Theoretical Methods

The geometries included in this study have been fully optimized at the BP86-D3/def2-TZVP level of theory using the program TURBOMOLE version 7.0.[12] For the calculations we have used the BP86 functional with the latest available correction for dispersion (D3).[13] In order to reproduce solvent effects, we have used the conductor-like screening model COSMO-RS,[14] which is a variant of the dielectric continuum solvation models.[15] This level of theory is a good compromise between the size of the systems (up tp 283 atoms) and the accuracy of the results. This level of theory has been previously used to rationalize anion-π catalysis in NDI derivatives, see ref [13] in the main text. The MEP surfaces and polarizability values were computed at the same level of theory by using the Gaussian-16 program[16] and using the TURBOMOLE optimized geometries. To estimate the polarizability perpendicular to the π-acidic surface, we have first calculated the mean plane of either the NDI or the bipy moiety (depending on the catalyst) and then the vector perpendicular to the mean plane. Secondly, the cartesian coordinate system of the optimized catalyst have been adjusted in such a way that the direction of the Z-axis coincides with the vector perpendicular to the mean plane. The keywords used In Gaussian-16 were polar (to print out the polarizability tensor) and NoSymmetry that prevents molecule reorientation and causes all computations to be performed in the input orientation.

The isosurface used to construct the MEP surfaces was 0.01 a.u. as best estimate of the van der Waals envelop. The MEP values given In the Figures and Table 2 (main text) do not correspond to the MEP maxima, that are located at the H-atoms of the NDI in the neutral catalysts and and the H-atoms of the protonated bipy units in the protonated catalysts.

The cartesian coordinates of all optimized compounds are given at the end of this document.

S7.2 Complementary Results and discussion

We have optimized the geometries (Figure S57) of catalysts **4a-c** and computed their MEP surfaces, which are shown in Figure S58. In all catalysts, the maximum MEP value at the NDI is located over the center of one of the imidic six-membered rings. The MEP value is slightly larger in catalyst **4a**, likely due to the extra effect of the t-Bu group (it forms C–H···π interaction that further contributes to enhance the π-acidity of the NDI via the bipy, i.e. C–H···π/π-π communication). This result is in disagreement with the experimental results, since catalyst **4b** is better than **4a** and **4c**. This strongly suggests that the catalyst is protonated at the bipy (or tetrazole ring) and, upon protonation, it becomes active. The MEP value over the bipy ligand in the unprotonated state is very small, revealing that it is not competitive compared to the NDI (as acidic surface).

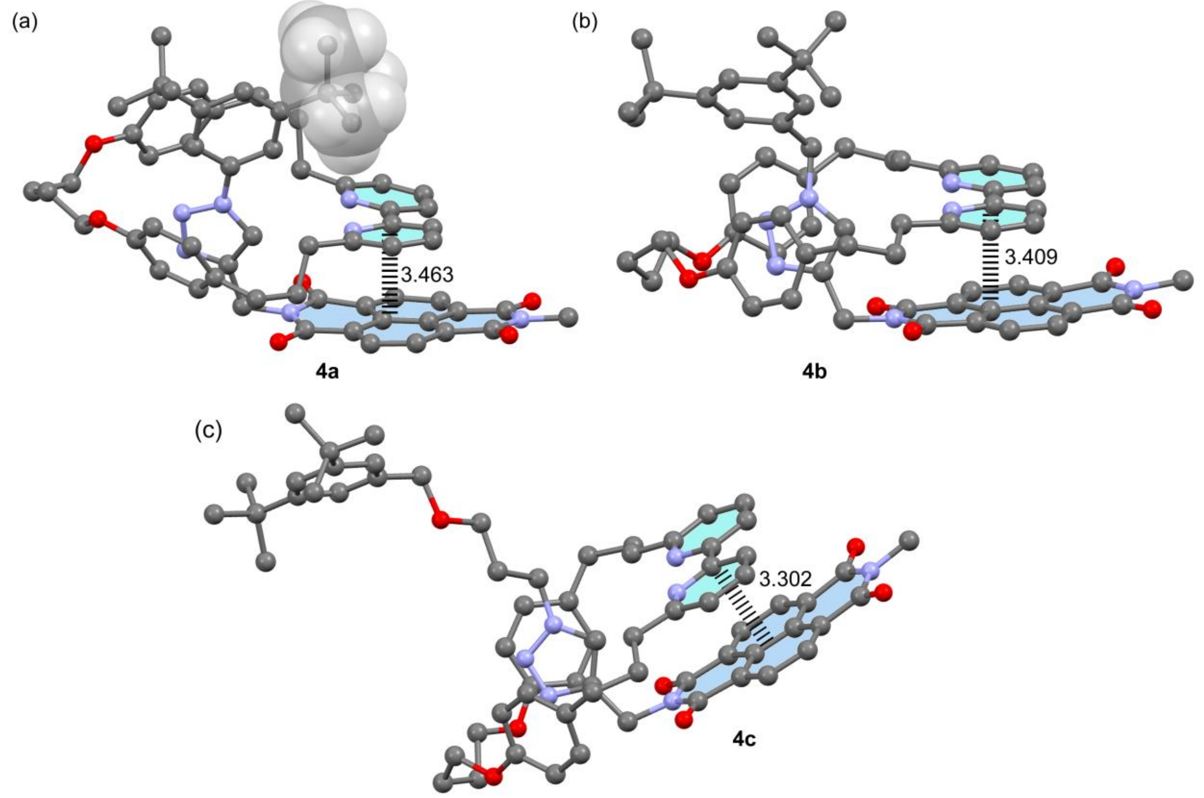

Figure S57 - BP86-D3/def2-TZVP optimized geometries of **4a** (a), **4b** (b) and **4c** (c). Distances in Å. H-atoms omitted for clarity.

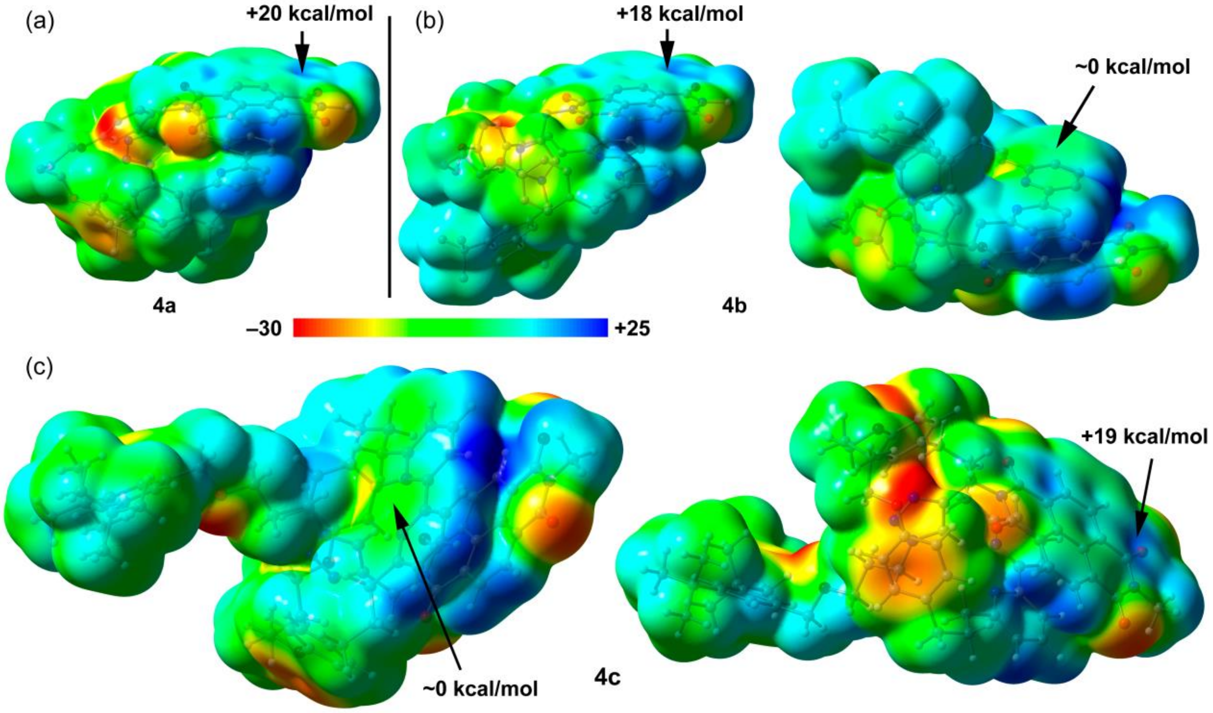

Figure S58 - MEP surfaces (isosurface 0.001 a.u.) of catalysts **4a** (a), **4b** (b) and **4c (c)**.

We have also optimized the protonated catalysts **4a-c** (denoted as **4a**H$^+$, **4b**H$^+$ and **4c**H$^+$, respectively), as shown in Figure S58, where the MEP surfaces in two different orientations are also represented. It can be observed that the protonation site is the bipy moiety in all three catalysts and points to one N-atom of the triazole ring (only this H-atom is represented in the molecule of Figures S58a,c,e distance ~1.8 Å). The MEP surface of the protonated

catalysts **4a-c**H+ (Figure S59) indicates that the bipy surface is slightly more acidic than the NDI one. Therefore, either may be the catalytic site. The possibility of protonated bipy to be the catalytic site is supported by the fact that catalyst **13** is active. In this molecule, both sides of the NDI are blocked by the bipy moieties (see Figure S60) and consequently only the bipy surface can act as an anion–π catalyst. It is also worth mentioning that the orientation of the di-tert-butylbenzyl moiety in catalyst **4c** (see FIgure S57c) drastically changes upon protonation, establishing CH···π interactions with the bipyridine macrocycle that is represented in Figure S59e.

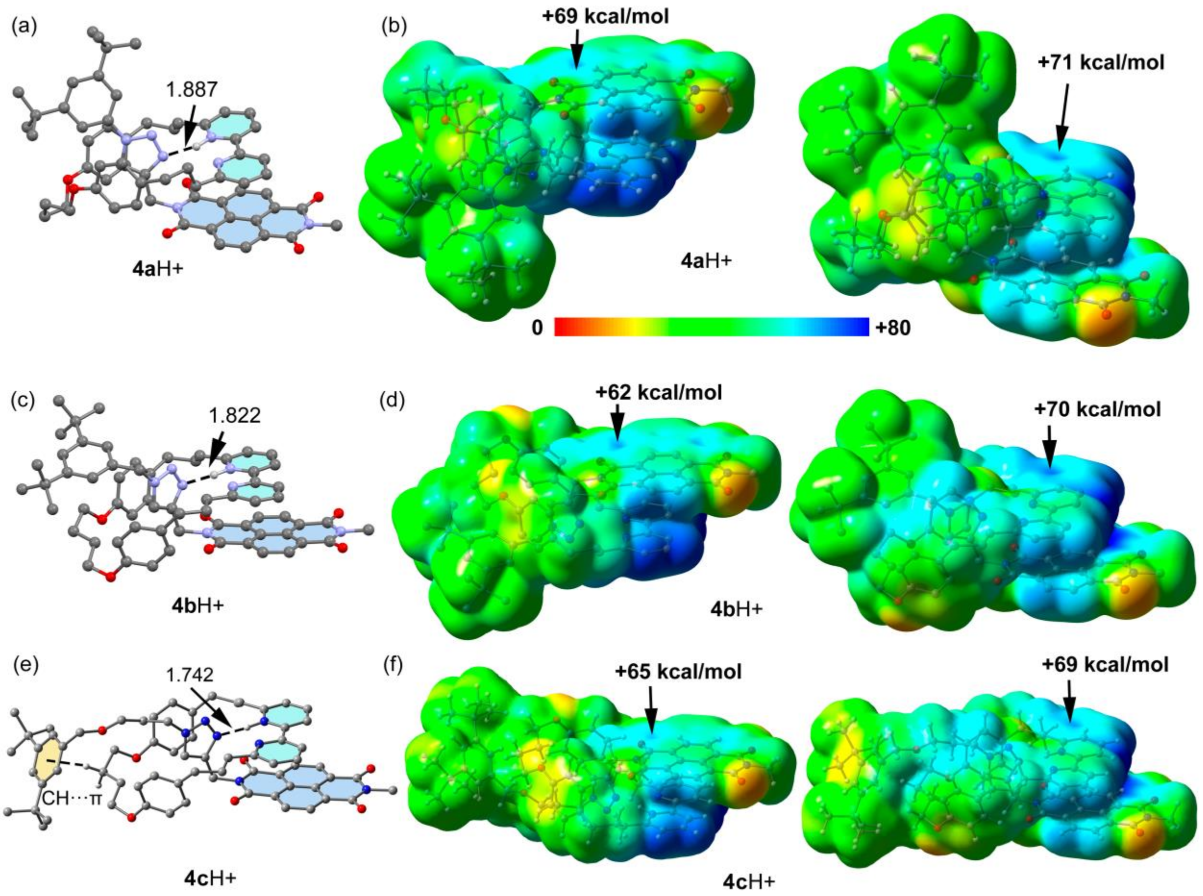

Figure S59 - (a,c,e) BP86-D3/def2-TZVP optimized geometries of **4a**H+ , **4b**H+ and **4c**H+. (b,d,f) MEP surfaces (isosurface 0.001 a.u.) of catalyst **4aH+ , 4bH+** and **4cH+.** H-atoms omitted for clarity apart from the H-atom of the protonated NH group in panels (a,c,e). Distances in Å.

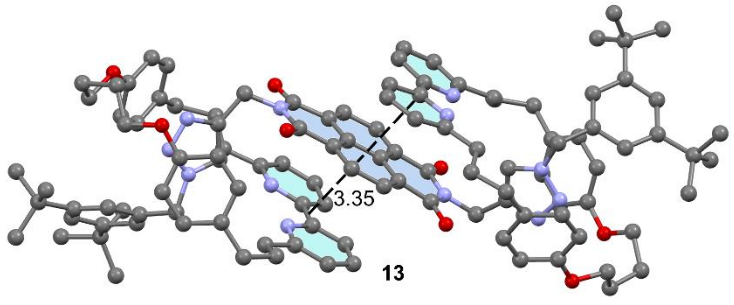

Figure S60 - Optimized geometry of catalyst **13** showing that the NDI surface is not accessible. H-atoms omitted for clarity. P-stacking distance in Å, measured using the C–C centroids of bipy and NDI fragments (C–C located at the geometric center in both moieties).

The geometry of the mono- and di-protonated catalyst **13**H+ and **13**H_2^{2+} are given in Figure S61. In both compounds the NDI is not available (or at least not entirely available) for interacting with the substrate, therefore in this case the catalytic π-surface is likely the protonated bipy moiety.

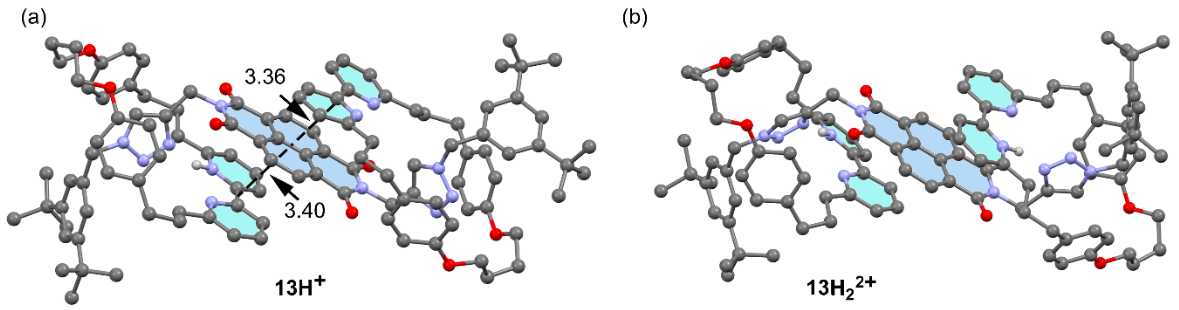

Figure S61 - Optimized complexes of **13**H^{+} (a) and **13**H$_2^{2+}$ (b) forms of catalyst **13**. Distances in Å.

The MEP surfaces of catalysts **13**H^{+} and **13**H$_2^{2+}$ have been calculated (Figure S62). In case of the monoprotonated catalyst, the MEP value over the bipyridine (+69 kcal/mol) ring is similar to those observed in **4a-c**H^{+} catalysts. For the **13**H$_2^{2+}$ catalyst, the MEP value over the protonated bipyridine is significantly more positive due to the dicationic nature of the system.

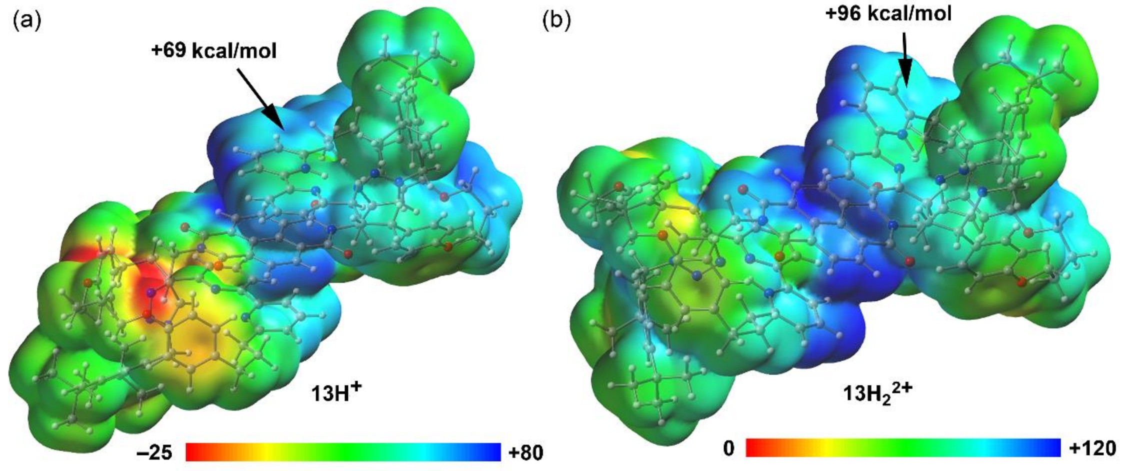

Figure S62 - MEP surfaces (isosurface 0.001 a.u.) of catalyst **13**H^{+} (a) and **13**H$_2^{2+}$ (b).

In addition, we have computed the MEP surfaces of the macrocycle (**1**) and its protonated form (**1**H^{+}), represented in Figure S63. As expected only the protonated form presents positive MEP over the π-system of the bipyridine moiety. The MEP maximum is located at the NH bond (100 kcal/mol). Moreover, the MEP value over the center of the protonated pyridine ring is larger (+87 kcal/mol) than those observed for the monoprotonated catalysts **4a-c**H^{+} and **13**H^{+}. This is likely due to lack of charge transfer effect in **1H^{+}**. That is, the strong N–H···N H-bond that is formed in the catalysts reduces the π-acidity of the ring (electron donation from the triazole N-atom to the NH bond). Moreover, MEP value over the center of the protonated pyridine ring is reduced due to presence of the π-cloud of the NDI underneath the bipyridine moiety in catalysts catalysts **4a-c**H^{+} and **13**H^{+}.

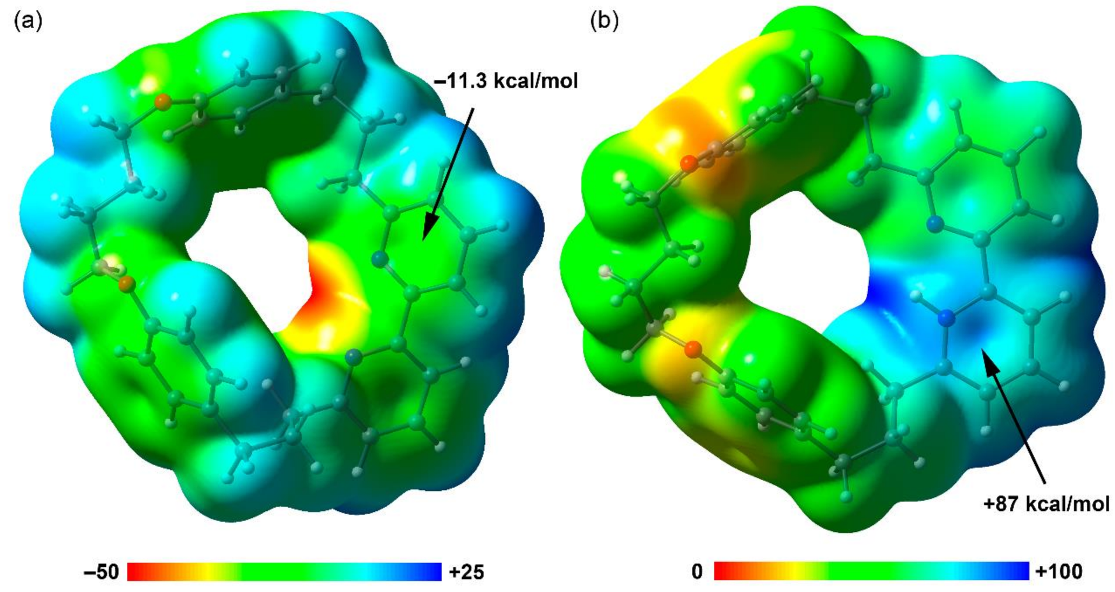

Figure S63 - MEP surfaces (isosurface 0.001 a.u.) of catalyst **1** (a) and **1**H⁺ (b) at the BP86-D3/def2-TZVP level of theory.

Figure S64 shows the MEP surfaces of the axle (**5**) and catalyst **6**H⁺ that shows catalytic activity in $CHCl_3$ in spite of the absence of the NDI ring, which supports the fact that the protonated bipyridine moiety can also act as a catalytic surface. The MEP value over the NDI of the isolated axle is +23 kcal/mol, in line with its non-catalytic bahaviour. The MEP value over the protonated bipyridine ring in catalyst **6**H⁺ is slightly smaller (in absolute value) than the values obtained for catalysts **4a-c**H⁺, in agreement with the smaller selectivity.

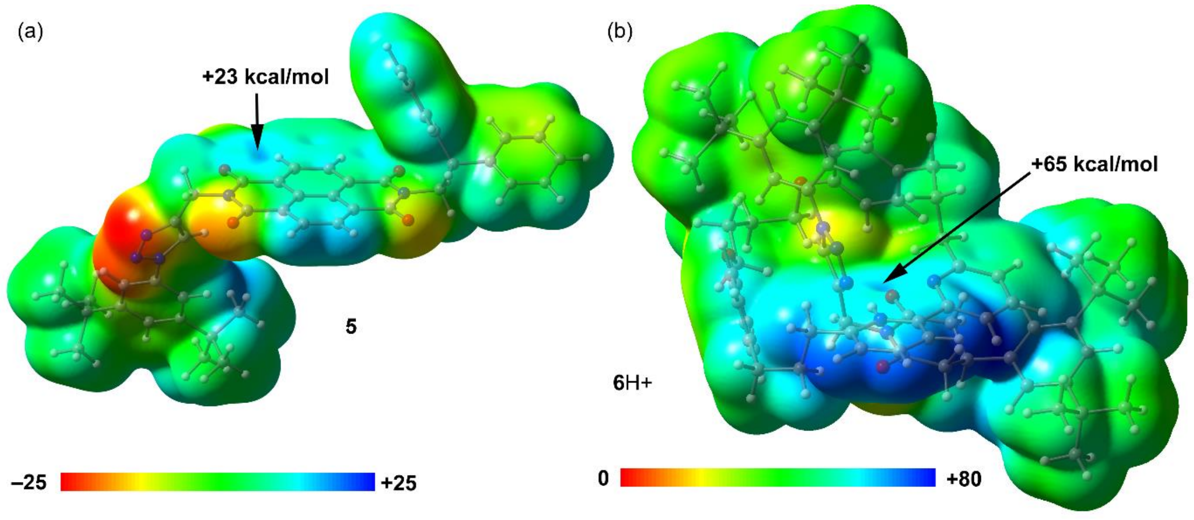

Figure S64 - MEP surfaces (isosurface 0.001 a.u.) of axle **5** (a) and catalyst **6**H⁺ (b) at the BP86-D3/def2-TZVP level of theory.

We computed the complexes of **4a-c**H⁺ with enolate **9**, the structures of which which are represented in Figure S65. In all cases, the enolate interacts with the aromatic surfaces by means of anion–π and π···π stacking interactions with distances ranging 3.07 to 3.59 Å. The interaction energies via the bipy surface are very similar for all three catalysts (–23.7 to –33.7 kcal/mol). In case of catalysts **4a**H⁺and **4b**H⁺, the interaction with the enolate is stronger via the NDI and, conversely, for catalyst **4c**H⁺ is stronger via the bipy moiety. This diffference can be attributed to the larger flexibility of the axle that affects the geometry of the supramolecular complex with the anolate. In fact, the π···π stacking distance "b" is the longest one for the complex of **9** with catalyst **4c**H⁺.

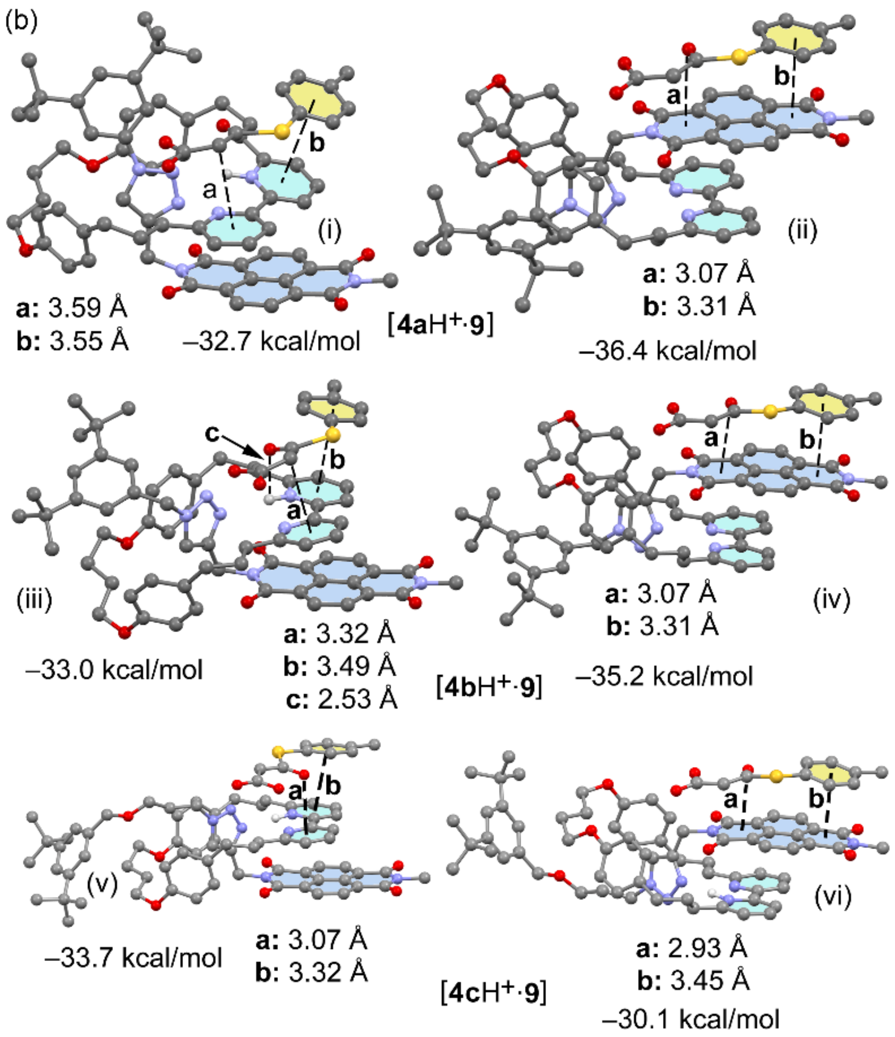

Figure S65 Optimized complexes of **4a-c**H⁺and **9** via the bipy moiety (i, iii and v respectively) or the NDI moiety (ii, iv and vi respectively).

We have also computed the complexes of axle **5** and catalyst **6**H⁺ with the enolate **9**, which are represented in Figure S66. For the axle, the interaction is significantly weaker than that of catalyst **6**H⁺, that is comparable (slightly smaller) to those obtained for for catalysts **4a-c**H⁺ also in agreement with the MEP surface analysis and its smaller selectivity.

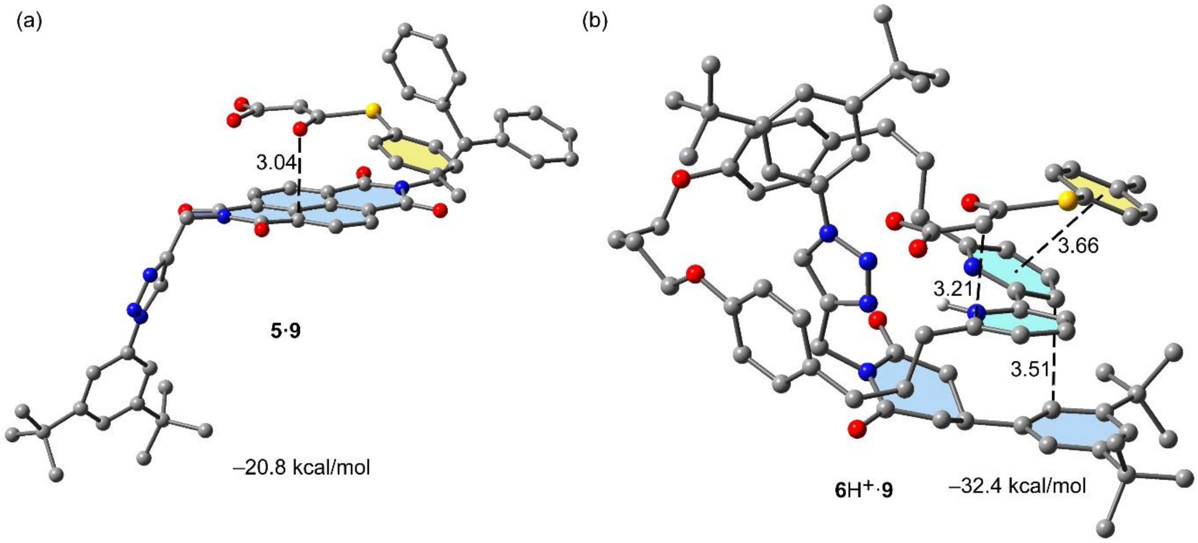

Figure S66 - Optimized complexes of **5·9** (a) and **6H⁺·9** (b). Distances in Å. The interaction energies are also indicated in $CHCl_3$.

The geometries of the optimized complexes of catalysts **13**H$^+$ and **13**H_2^{2+} with the substrate are given in Figure S67 and those of **1**H$^+$ with the substrate in two orientations (anion–π and HB) are shown in Figure S68. The complexes with catalysts **13**H$^+$ and **13**H_2^{2+} show the substrate interacting with the bipyridine moiety at distances that are shorter than those obtained for the complexes with catalysts **4a-c**H$^+$ in line with the larger interaction energy. It is worthy to comment the shortening of the π-stacking distance between the NDI and the bipyridine moiety, also suggesting a reinforcement of the π-stacking upon complexation. In case of the isolated macrocycle, the anion–π complex is less favorable than the **13**H$^+$ complex but more favorable than **4a-c**H$^+$ complexes, in agreement with the MEP energies, but in disagreement with the experimental A/D value. A likely explanation if that the formation of the H-bonded complex via the NH bond is more favorable. In this binding mode the approximation of the electrophile is not possible, thus favoring the decarboxylation reaction.

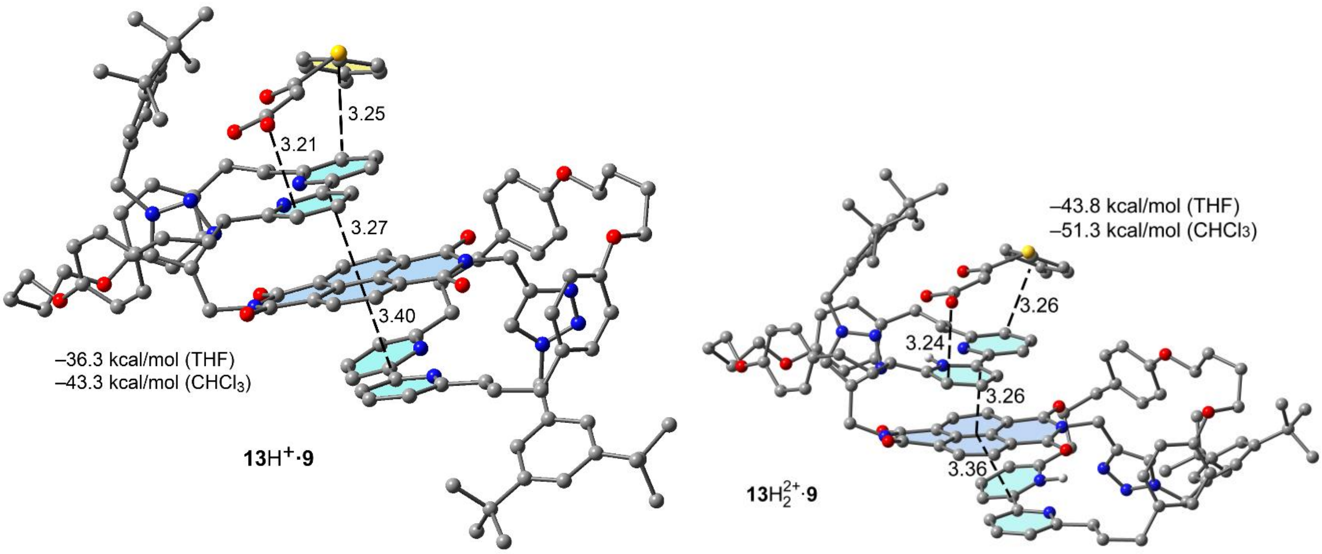

Figure S67 - Optimized complexes of catalysts **13**H$^+$ (left) and **13**H_2^{2+} (right) with the malonate half-thioester (**9**) interacting with the bipyridine moiety. Distances in Å. H-atoms omitted for clarity. Interaction energies are indicated (PB86-D3/def2-TZVP, COSMO=THF and $CHCl_3$).

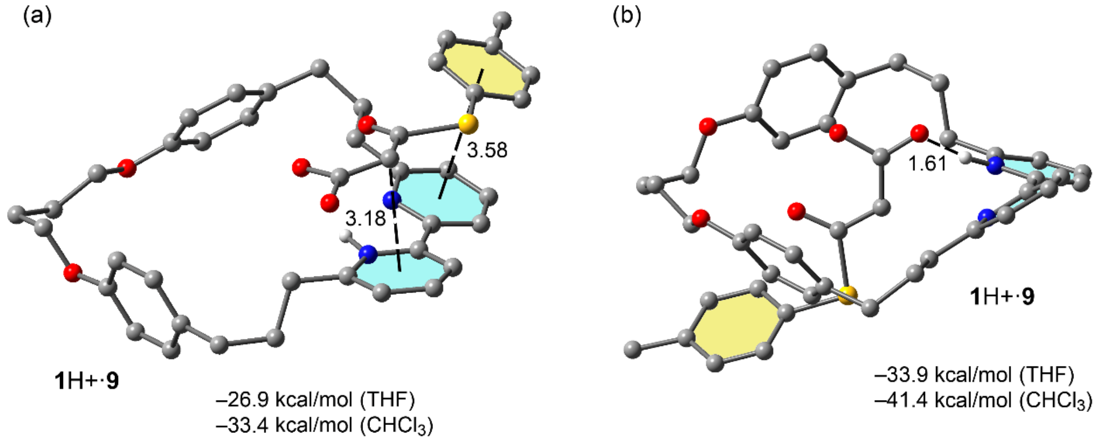

Figure S68 - Optimized complexes of **1**H$^+$ and the malonate half-thioester interacting via anion–π and H-bonfding (b). Distances in Å. H-atoms omitted for clarity apart from the one of the NH group. Interaction energies are indicated (PB86-D3/def2-TZVP, COSMO=THF and $CHCl_3$).

S7.3 pKa and related discussion

First, the pKa difference between catalysts **4a** and **4b** has been computed, using the methodology proposed by Ho *et al.* that uses the ΔG values in the gas phase and solvent.[17] The reaction considered was **4a**H^+ + **4b** → **4a** + **4b**H^+ The theoretical calculations at the PB86-D3(COSMO-RS)/def2-TZVP level of theory confirm that catalyst **4b** is 1.45 pKa units more basic than catalyst **4a** in THF and 1.35 pKa units in $CHCl_3$, due to the formation of a stronger H-bond upon protonation (shorter H-bond distance and better directionality, see Figure S59).

Figure S69 shows the MEP surfaces of 1-phenyl-1*H*-1,2,3-triazole and 1-benzyl-1*H*-1,2,3-triazole that have been computed in order to investigate if the electronic nature of the triazole ring changes depending on the substituent in N1. The MEP minimum is located in both compounds at the N3 atom of triazole, thus revealing that it is most nucleophillic one. The MEP value is more negative at the N3 atom of 1-benzyl-1*H*-1,2,3-triazole, suggesting that the latter is better H-bond acceptor than the former, in line with the stronger basicity of catalyst **4b.**

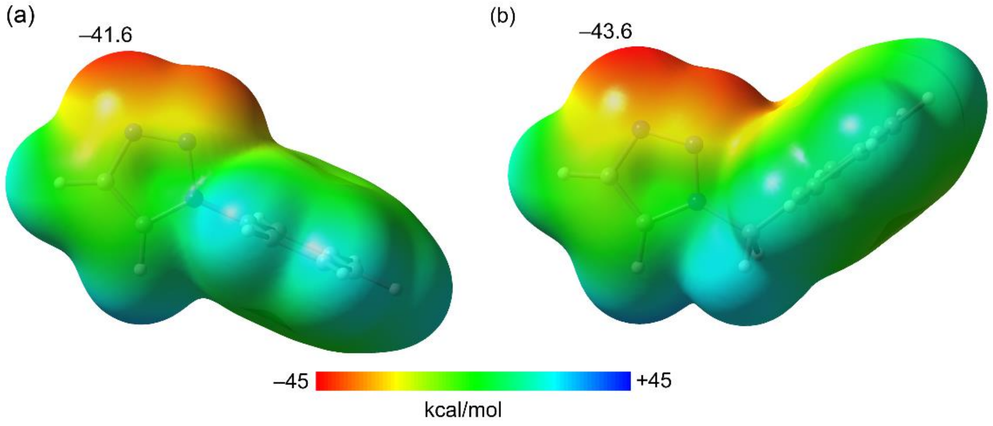

Figure S69 - MEP surfaces (isosurface 0.001 a.u.) of 1-phenyl-1H-1,2,3-triazole (a) and 1-benzyl-1H-1,2,3-triazole (b).

S8. ELECTROCHEMISTRY RESULTS

Electrochemical measurements were performed using a glassy carbon button working electrode (area = 0.071 cm^2), a graphite rod counter electrode and a Ag/$AgNO_3$ pseudo-reference electrode. The substrates were dissolved in a solution of NBu_4PF_6 (0.1 M) in dry CH_2Cl_2 to a concentration of 5 mM. A volume of solution was removed and transferred to the measurement vessel, and the volume of solvent was doubled with dry CH_2Cl_2. This additional solvent was then removed by bubbling argon through the solutions, typically for a period of approximately 20 min. The purpose of this exercise was to ensure a fully degassed solution of known concentration. The CV (scan rate = 0.1 V s^{-1}) was then measured. To measure cyclic voltammograms of the protonated species, as these are the active forms of the catalysts, one equivalent of TsOH·H_2O per triazole unit in the substrate was added prior to degassing. Measured values of $E_{1/2}$ (Table S5) have an experimental precision of ±20 mV.

The cyclic voltammograms (Figure S71) of axle **5** (the axle of rotaxane **4b**) and axle **S6** (the axle of rotaxane **13**) suggest that the NDI reduction event occurs at less negative values compared with the corresponding interlocked structures, albeit the effect is small in the case of **5**/**4b**. This suggests the NDI unit is more electron rich in the interlocked structures which could result from the donation of electron density from the bipyridine macrocycle into the NDI through π-π stacking, as observed in the solid-state structure of **4a**, and in computational models of the series of rotaxanes. Upon the addition of TsOH (1 equiv. per triazole moiety), a positive shift was observed in the values of the first and second reduction potentials for the interlocked species **4a**, **4b**, and **13**, but not for the isolated axles. These different behaviours indicate a reduction in electron density in the NDI unit upon protonation of the bipyridine macrocycle, potentially through π-π stacking as observed in the solid-state structure of [**4a**H]PF_6 and computational models of the protonated catalysts. In the case of **4c**, the cyclic voltammogram in the presence of TsOH exhibited significant broadening.

To determine if catalyst selectivity was controlled by the inherent π-acidity of the NDI unit, the E_{LUMO} values for the interlocked catalysts **4a**-**c** and **13** were calculated (Table S5), relative to the ferrocenium/ferrocene couple according to the equation $E_{LUMO} = -5.1 - E_{1/2}$.[18] Within the series of [2]rotaxanes, decreasing axle length led to a less negative value for E_{LUMO} in the neutral form, suggesting an increase in electron density for the NDI unit with decreasing axle length through strengthened π-π stacking. Comparison between **4b** and **13** in the neutral form demonstrates that the presence of an additional macrocycle in the [3]rotaxane **13** led to a less negative value of E_{LUMO}, suggesting that the second macrocycle is also engaged in π-π stacking with the NDI unit in solution. Upon protonation, **4a**, **4b**, and **13** showed more negative values for E_{LUMO}, with **13** possessing the greatest shift of -230 meV vs -170 meV. No trend could be found to link the absolute values of E_{LUMO} with catalyst selectivity in either neutral or protonated forms, suggesting that the inherent π-acidity of the NDI unit does not directly determine catalyst selectivity, consistent with the proposal from the computational results that polarizability is a key parameter.

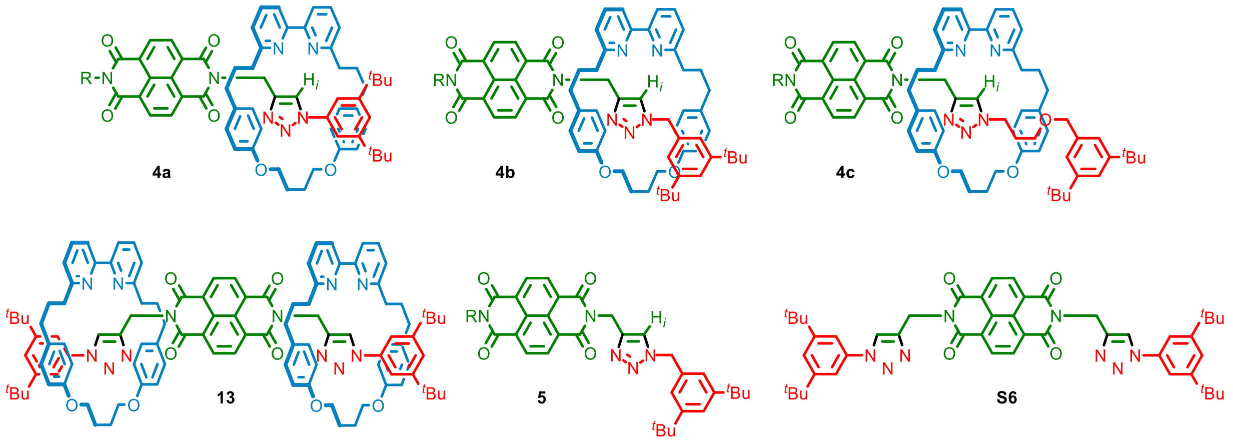

Figure S70 - Structures of compounds examined by cyclic voltammetry.

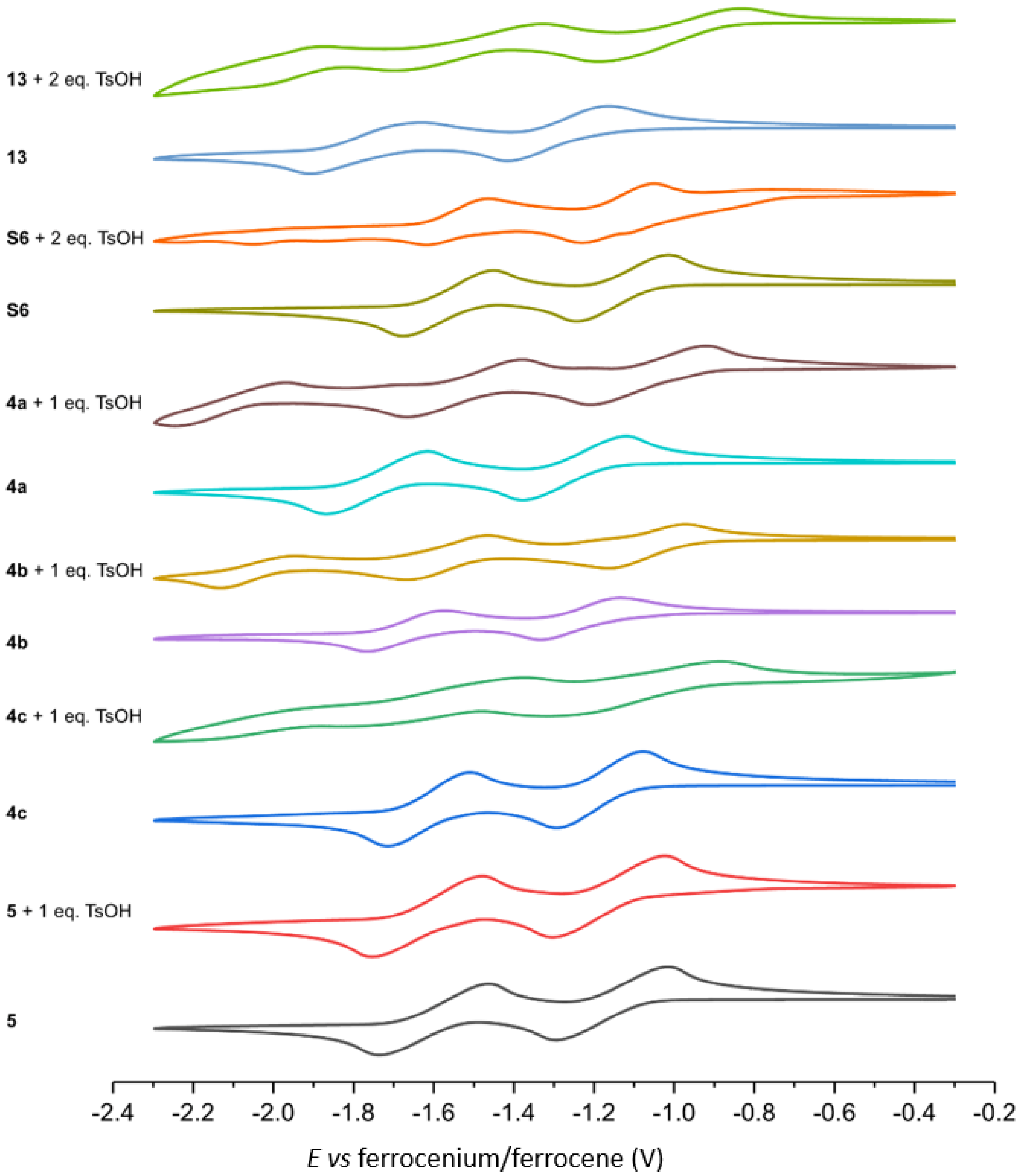

Figure S71 - Normalised cyclic voltammograms of rotaxanes **4a**-**c** and **13**, and axles **5** and **S6** (5 mM in CH_2Cl_2, 0.1 M NBu_4PF_6) in the absence and presence of TsOH (1 equiv. per triazole) with ferrocene as an internal standard (*E* = 0 V).

Table S5 - Tabulated values of $E_{1/2}$ and E_{LUMO} in the absence or presence of TsOH of rotaxanes **4** and **13**, and selected axles.[a]

	In the absence of TsOH		In the presence of TsOH		
Catalyst	$E_{1/2}$ (V)	E_{LUMO}[b] (eV)	$E_{1/2}$ (V)	E_{LUMO}[b] (eV)	$\Delta E_{1/2}$[c] (mV)
**5**	-1.15 ± 0.02	-3.95 ± 0.02	-1.16 ± 0.02	-3.94 ± 0.02	-10 ± 40
**4a**	-1.25 ± 0.02	-3.85 ± 0.02	-1.06 ± 0.02	-4.04 ± 0.02	+190 ± 40
**4b**	-1.23 ± 0.02	-3.87 ± 0.02	-1.07 ± 0.02	-4.03 ± 0.02	+160 ± 40
**4c**	-1.19 ± 0.02	-3.91 ± 0.02	-1.10 ± 0.02	-4.00 ± 0.02	+90 ± 40
**S6**	-1.13 ± 0.02	-3.97 ± 0.02	-1.14 ± 0.02	-3.96 ± 0.02	-10 ± 40
**13**	-1.29 ± 0.02	-3.81 ± 0.02	-1.01 ± 0.02	-4.09 ± 0.02	+280 ± 40

[a]Experiments were conducted at 5 mM in CH_2Cl_2 (0.1 M NBu_4PF_6) in the absence or presence of TsOH (1 equiv. per triazole) using ferrocene as an internal standard (E = 0 V). [b]Calculated using literature value for ferrocene (-5.1 eV) as reference. [c]Note that $\Delta E_{1/2}$ is numerically equivalent to ΔE_{LUMO}.

S9. OTHER ANION-Π CATALYSED REACTIONS INVESTIGATED WITH ROTAXANE 13

When best performing rotaxane **13** was applied to the domino Michael-Henry reaction previously studied by Matile and co-workers (Scheme S2), reaction progress was incredibly slow. Almost no progress was detected after 2 days, and after 400 h, significant quantities of the 1,2-cyclohexanedione substrate still remained. Although conversion is slow, the same major diastereomer was observed as by Matile and co-workers with their NDI derived anion-π catalysts.[19]

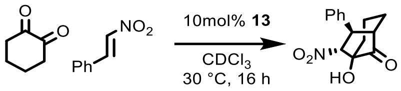

Scheme S2 - Domino reaction of 1,2-cyclohexanedione and *E*-nitrovinylbenzene previously studied under anion-π catalysis.

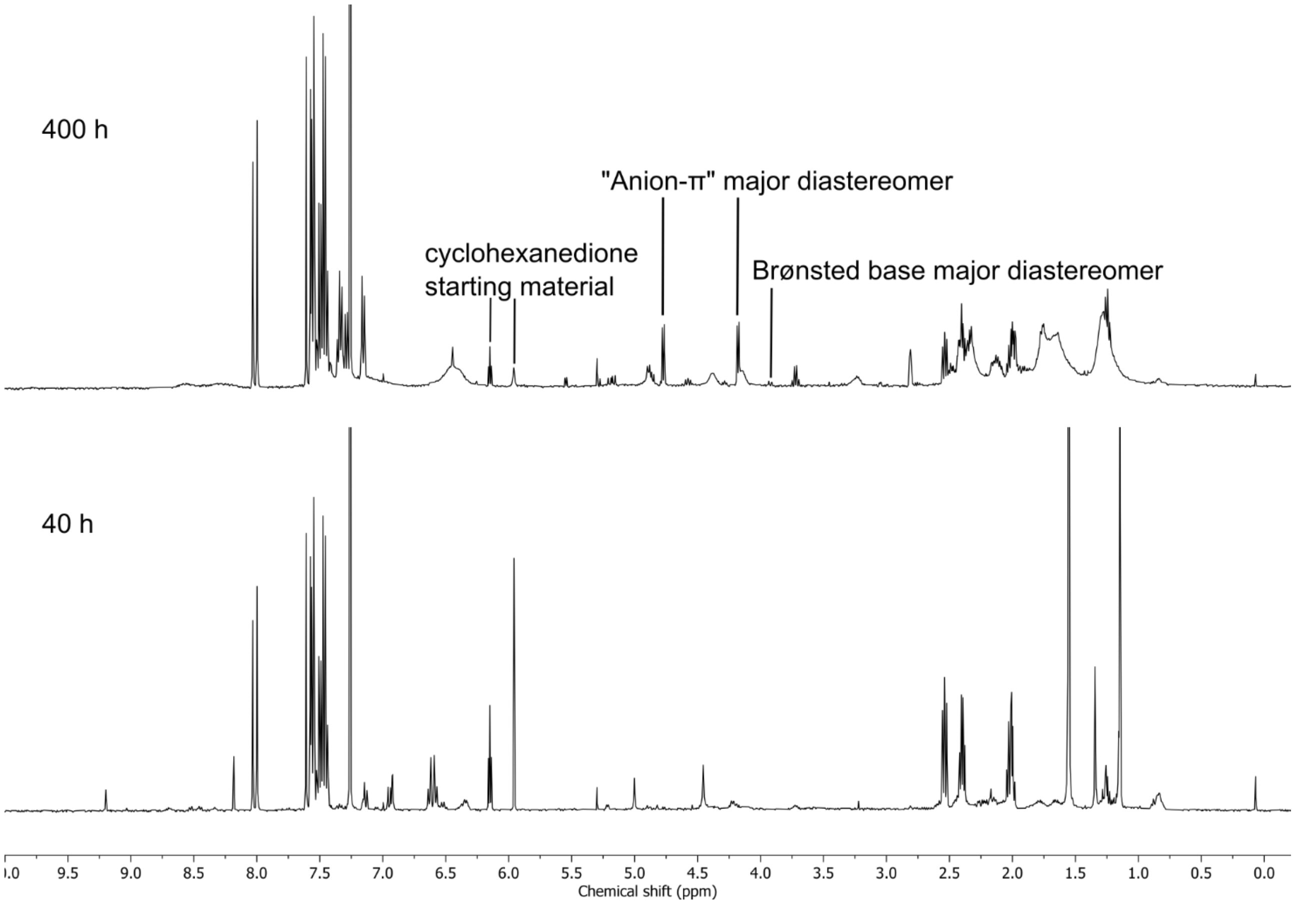

Figure S72 - Crude ^{1}H-NMR of the cascade reaction shown in Scheme S2, with signals corresponding to the two observed diastereomers of the bicyclic product.

In the case of the Michael addition shown below in Scheme S3, previously studied with chiral, bifunctional amine-NDI or amine-fullerene catalysts to examine the potential utility of anion-π catalysis in the formation of products containing adjacent stereocentres,[20] no conversion of starting materials was observed in 88 h, therefore reaction monitoring was abandoned.

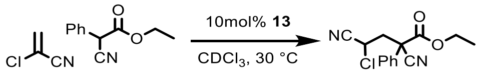

Scheme S3 - Michael addition resulting in the formation of adja previously studied under anion-π catalysis.

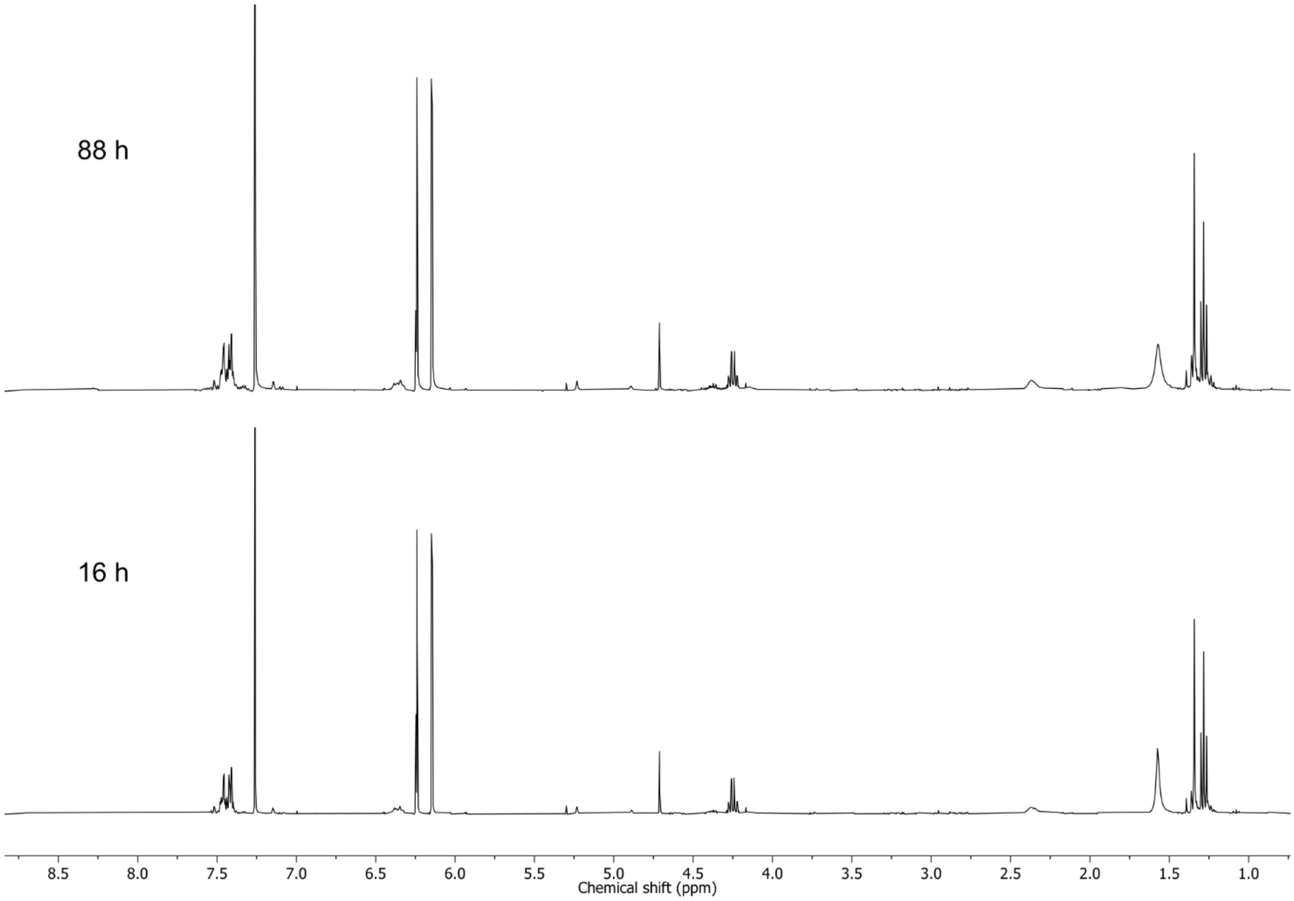

Figure S73 -^{1}H NMR spectra of aliquots taken from reaction shown in Scheme S3, with no conversion of starting materials detected.

In the case of the Kemp elimination (Scheme S4), previously studied by Matile and co-workers with NDI derived anion-π catalysts,[21] no conversion was detected after 40 h, and so reaction monitoring was abandoned. Whilst no conversion is observed, significant shifts in the ^{1}H-NMR signals of rotaxane **13** are observed in solution. An approximate titration was then performed with 0.5, 1.0 and 5.0 equivalents of benzisoxazole into a solution of **13** in $CDCl_3$, in which ttwo sets of signals for **13** can be seen in slow exchange, with a single species formed at 5 equivalents of benzisoxazole, as in the catalysis crudes.

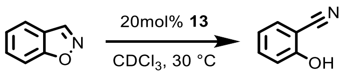

Scheme S4 - Kemp elimination with rotaxane **13**.

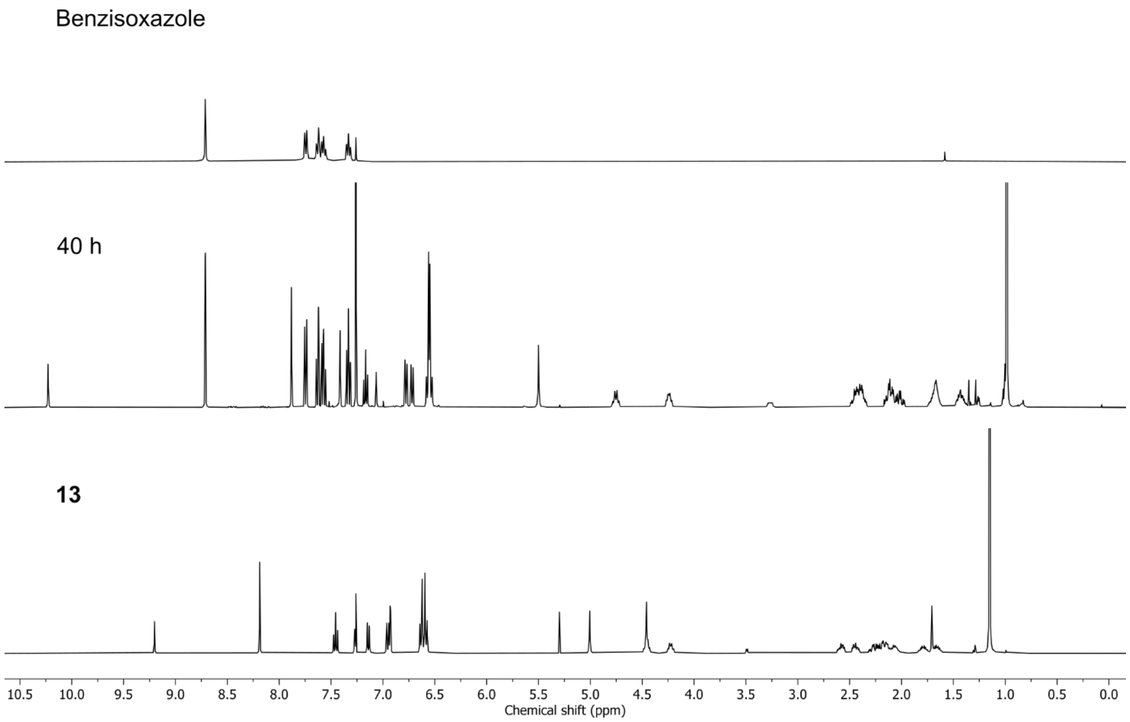

Figure S74 - Stacked ^{1}H-NMR (400 MHz, $CDCl_3$, 298 K) spectra of benzisoxazole substrate, Kemp elimination reaction crude after 40 h, and catalyst **13**. Whilst no conversion is detected, significant shifts in the ^{1}H-NMR of catalyst **13** are observed in the crude reaction mixture.

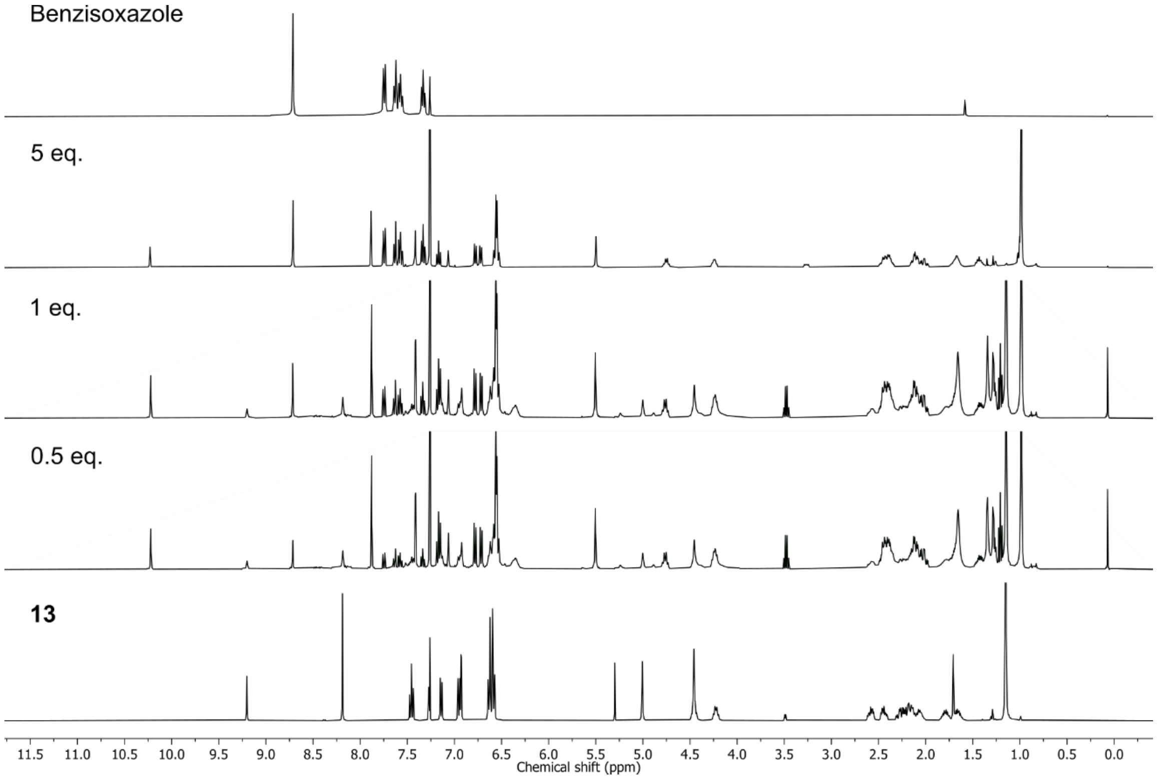

Figure S75 - ^{1}H-NMR (400 MHz, CDCl3, 298 K) of benzisoxazole, rotaxane **13**, and increasing equivalents of benzisoxazole.

S10. CARTESIAN COORDINATES OF MODELLED STRUCTURES

Neutral catalyst structures

```
1
O     0.5421288    1.2123080    4.2896675
N     1.8987820   -2.6176384   -1.6457392
N    -0.0017388   -1.0525189   -3.1195402
O    -2.8795963    4.3753381    1.9393281
C     1.1124747   -1.5536803   -3.6773564
C     2.6781563   -3.4620182    0.4922475
H     2.6051329   -4.4369464    1.0017679
H     1.8775514   -2.8167002    0.8799040
C     2.5616914    0.6776742    3.2100853
H     2.6939575    1.7485959    3.0541181
C     1.7672563   -2.7011680   -2.9821330
C    -0.6321289   -0.0290745   -3.7268972
C     3.3140984   -1.6245116    2.9209223
C     1.4156606    0.2344365    3.8883179
C     2.4814615   -3.6363178   -0.9915100
C     4.0455720   -2.8285495    0.8049748
H     4.8453227   -3.4702904    0.3989653
H     4.1136721   -1.8630732    0.2785185
C     3.4889085   -0.2423666    2.7353167
H     4.3668113    0.1182895    2.1940216
C     1.2325438   -1.1368255    4.1082521
H     0.3663504   -1.5075969    4.6536909
C     2.2133997   -3.8097378   -3.7190557
H     2.0673672   -3.8481140   -4.7988860
C     1.6570934   -1.0473685   -4.8699741
H     2.5758130   -1.4674080   -5.2798956
C    -4.0345252    1.8327286   -0.5079214
H    -4.4504536    0.8232568   -0.5349030
C     2.1795120   -2.0450034    3.6211192
H     2.0194857   -3.1128310    3.7874084
C    -3.7566387    2.4140871    0.7339934
H    -3.9707900    1.8540757    1.6426708
C    -1.8876894    0.4548540   -3.0347461
H    -2.4936069   -0.4351738   -2.7956234
H    -1.5953046    0.8695280   -2.0554319
C     2.9442025   -4.7858954   -1.6536334
H     3.4033204   -5.5957495   -1.0844163
C    -0.1484083    0.5368244   -4.9172155
H    -0.6687385    1.3682293   -5.3907612
C    -3.2476800    3.7975511   -1.6303356
H    -3.0478837    4.3525489   -2.5501062
C    -3.2010479    3.6994090    0.7898252
C    -3.7851732    2.5012058   -1.7097636
C    -2.9560802    4.3909694   -0.4076505
H    -2.5336930    5.3945863   -0.3515140
C     1.0137026    0.0196157   -5.4899943
H     1.4150930    0.4497716   -6.4090665
C     2.8060969   -4.8710468   -3.0366100
H     3.1463224   -5.7563614   -3.5762567
C     4.2831728   -2.6099060    2.3136976
H     4.2074261   -3.5774515    2.8358557
H     5.3157102   -2.2532650    2.4589550
C    -4.0330356    1.8487535   -3.0497358
H    -4.6223341    2.5267574   -3.6887269
H    -4.6431956    0.9418442   -2.9095152
C    -1.7344502    2.6743193    3.3097225
H    -1.8393383    1.8849669    2.5491681
H    -0.7930580    3.1936034    3.0835302
C    -2.7356361    1.4748558   -3.7949619
H    -2.9987440    1.0780638   -4.7896458
H    -2.1508578    2.3938552   -3.9683540
C    -0.7621552    0.8121883    4.7287713
H    -0.7056289    0.3771721    5.7431122
H    -1.1627878    0.0417337    4.0442832
C    -2.8886614    3.6648620    3.1870352
H    -3.8636326    3.1664846    3.3418776
H    -2.8017378    4.4548087    3.9470154
C    -1.6607622    2.0396136    4.7035159
H    -2.6633496    1.7186603    5.0350866
H    -1.3047081    2.7761616    5.4411345
```

4a

O	-3.4096718	-2.3735691	0.8112595
O	-4.1614692	2.1241850	1.3114058
N	0.3795098	-0.0751259	1.6674611
N	-3.8035226	-0.1327691	1.0697831
N	0.3632530	-0.0479669	3.0276190
N	-0.8908230	-0.0852393	3.4066827
O	-6.7213163	2.4552660	-5.2525365
N	-5.9943394	0.3041001	-5.6263717
C	1.6033538	-0.0341749	0.9437825
O	-5.2226001	-1.8108038	-6.0172357
C	-1.6878219	-0.1374486	2.2974987
C	-0.8892635	-0.1347689	1.1720340
H	-1.0999439	-0.1586081	0.1044772
C	-4.8052152	-0.0013375	-1.5641603
C	3.9877364	-0.3832914	0.8974638
C	-4.2726541	-1.2205706	-1.0846227
C	-3.8042000	-1.3213780	0.3170011
C	2.7760258	-0.4452027	1.5836246
H	2.7151195	-0.7954650	2.6096253
C	1.6017270	0.4261263	-0.3726218
H	0.6604346	0.7362945	-0.8280443
C	3.9788333	0.0818386	-0.4299172
H	4.9244077	0.1202572	-0.9673663
C	-4.8778855	1.1428380	-0.7343392
C	-5.2552237	0.0831189	-2.9121142
C	-4.2656901	1.1188276	0.6180545
C	-3.1803319	-0.1954430	2.4007382
H	-3.4961567	-1.1325227	2.8773164
H	-3.5574123	0.6529888	2.9832632
C	5.3107331	-0.8309133	1.5353141
C	2.8108542	0.4872192	-1.0851533
C	-4.1799649	-2.3211588	-1.9285508
H	-3.7780900	-3.2516238	-1.5322031
C	-5.8317536	1.2883977	-3.3786165
C	2.8225542	0.9320695	-2.5575968
C	-5.0877726	-1.0305632	-3.7682252
C	-4.5628506	-2.2189742	-3.2742483
H	-4.4405543	-3.0587098	-3.9572728
C	-5.9733902	2.3691047	-2.5160957
H	-6.4298228	3.2820521	-2.8977858
C	-5.4776336	2.3045080	-1.2033099
H	-5.5235598	3.1697510	-0.5431582
C	5.1473757	-1.1805006	3.0226828
H	4.7772617	-0.3218408	3.6023047
H	6.1209401	-1.4717469	3.4432102
H	4.4531330	-2.0157798	3.1813534
C	5.8138533	-2.0874991	0.7888152
H	5.0698462	-2.8947629	0.8551831
H	6.7563034	-2.4451321	1.2303185
H	5.9934468	-1.8804754	-0.2759367
C	2.2953443	-0.2385777	-3.4171536
H	2.9089389	-1.1391575	-3.2703242
H	2.3166323	0.0262079	-4.4855807
H	1.2613139	-0.4828743	-3.1471709
C	6.3578635	0.2980675	1.4164411
H	6.5708530	0.5586529	0.3707131
H	7.3046268	-0.0139701	1.8822911
H	6.0100700	1.2084108	1.9261767
C	-6.2349931	1.4232014	-4.8010679
C	1.9127438	2.1592692	-2.7708378
H	0.8716697	1.9608590	-2.4862062
H	1.9175465	2.4482501	-3.8324464
H	2.2673570	3.0177926	-2.1831525
C	-5.4290136	-0.9138284	-5.2047770
C	-6.3340204	0.3955264	-7.0511124
H	-7.0306077	-0.4092233	-7.3142603
H	-6.7866579	1.3739768	-7.2278538
C	4.2369473	1.2999617	-3.0420083
H	4.6865680	2.0853420	-2.4167200
H	4.1854200	1.6759162	-4.0738618
H	4.9121444	0.4326268	-3.0456313
O	2.5374562	-3.0568643	4.3073567
O	2.6916385	1.1429678	5.3048716
N	-1.1496358	-0.6928304	-2.1903995
N	-1.5252911	1.8595545	-1.3678934
C	-1.6963938	0.1631204	-3.0809893
C	-2.0087982	1.5344803	-2.5869928
C	0.4649421	-4.4376490	0.9364005
C	1.8043874	-3.4749862	3.2296693

C	-0.7742048	-1.9151460	-2.5988193
C	-1.7485050	3.0857967	-0.8744220
C	-0.2154023	-3.7014980	1.9156623
H	-1.2792435	-3.4848223	1.7942330
C	-0.9940512	-2.3577792	-3.9158049
H	-0.6838674	-3.3613525	-4.2094992
C	-1.0678203	3.4573694	0.4131532
H	-0.9356371	2.5674081	1.0432213
H	-1.6973030	4.1679561	0.9712298
C	1.8369775	-4.6614993	1.1223407
H	2.3960466	-5.2197013	0.3675487
C	2.5048858	-4.1916726	2.2500108
H	3.5710939	-4.3699816	2.3931695
C	1.3921056	3.8588364	2.3911904
C	-1.9219963	-0.2065254	-4.4167618
H	-2.3465718	0.4950425	-5.1322829
C	0.4350002	-3.2255256	3.0549473
H	-0.1281440	-2.6537018	3.7881697
C	-0.0886828	-2.8109227	-1.6048710
H	0.2830945	-2.2081027	-0.7655552
H	0.7929060	-3.2647596	-2.0884520
C	0.5841943	3.5925979	3.5013022
H	-0.3848701	4.0888084	3.5884900
C	-0.2610707	-4.9877527	-0.2698462
H	-0.9860860	-5.7539332	0.0534748
H	0.4629484	-5.5037542	-0.9222031
C	0.9790481	2.7028420	4.5028891
H	0.3059528	2.5005111	5.3327571
C	-1.5805739	-1.4920296	-4.8312395
H	-1.7653457	-1.8045678	-5.8592931
C	2.2151466	2.0533991	4.3995382
C	-2.7370175	2.4501440	-3.3597288
H	-3.1329613	2.1794461	-4.3364518
C	-2.4987892	4.0431504	-1.5823938
H	-2.6760702	5.0261158	-1.1445819
C	-1.0116875	-3.9269425	-1.0882474
H	-1.5092435	-4.4185372	-1.9414632
H	-1.8047373	-3.4739298	-0.4751220
C	-2.9893669	3.7186095	-2.8412528
H	-3.5773408	4.4367835	-3.4143144
C	0.2973430	4.1050445	0.0984571
H	0.1638352	4.8409265	-0.7124335
H	0.9732746	3.3271585	-0.2887682
C	0.9510992	4.8042816	1.3022285
H	1.8225620	5.3726867	0.9395131
H	0.2450339	5.5447757	1.7137781
C	3.0427797	2.3156283	3.2987807
H	3.9971571	1.7959983	3.2246834
C	2.6280230	3.1966929	2.3077261
H	3.2748248	3.3663313	1.4440390
C	2.9872267	-1.5812045	6.1320948
H	3.4217343	-2.3957501	6.7310743
H	3.7926232	-1.1749570	5.5061088
C	2.4646658	-0.4734581	7.0579783
H	3.3040469	-0.0818846	7.6524664
H	1.7312598	-0.8795576	7.7748023
C	1.9095186	-2.1329605	5.2164423
H	1.4422638	-1.3238940	4.6340948
H	1.1201388	-2.6512174	5.7925300
C	1.7890762	0.6786904	6.3207707
H	1.5562395	1.4987753	7.0237145
H	0.8453270	0.3556189	5.8481247
H	-5.4276844	0.2740950	-7.6571973

4b

O	4.2697344	-1.7046303	-7.5654648
O	2.2323738	-5.7472076	-6.8424774
O	-0.7598977	1.4999172	-3.7486609
N	3.2223226	-3.7100610	-7.2466804
O	-2.4743553	-2.6082818	-2.6270799
N	-1.6081531	-0.5491490	-3.1709384
N	-0.2900407	1.1955052	0.4362112
C	1.2216391	-2.4345552	-5.7197386
C	3.3127622	-2.3141255	-7.0948844
N	-2.2527061	1.6773413	-0.2855401
C	2.2312245	-1.6537062	-6.3296045
C	1.1772494	-3.8389164	-5.8877230
N	-1.3675988	1.9858767	0.6343733
C	0.3018755	-0.3961270	-4.7173572
C	2.2184559	-4.5298778	-6.6879060
C	1.2852529	0.3528368	-5.3515761
H	1.2969183	1.4315428	-5.1979186
C	-0.7196785	-2.5829315	-4.2287249
C	-0.7642980	-3.9557652	-4.4352999
H	-1.5233686	-4.5358795	-3.9124522
C	0.2558086	-1.7993222	-4.8890782
C	-1.7464336	0.6815997	-1.0651586
C	2.2530019	-0.2757715	-6.1495953
H	3.0521382	0.2972235	-6.6186605
C	-0.7044036	0.2813711	-3.8650467
C	0.5871172	1.0542446	2.7301277
C	-0.4789827	0.3565891	-0.6136318
H	0.2781206	-0.3542976	-0.9449503
C	0.1776881	-4.5803736	-5.2685833
H	0.1645919	-5.6599781	-5.4163737
C	0.8470347	-0.1936718	3.2894717
H	1.2988099	-0.9650606	2.6619594
C	-0.0014099	2.0613839	3.5077295
H	-0.2039315	3.0197365	3.0316574
C	-2.5119152	0.1194335	-2.2186171
H	-3.2395642	-0.6306897	-1.8848795
H	-3.0377629	0.9316073	-2.7369719
C	0.8986115	1.3290653	1.2790033
H	1.2832423	2.3507740	1.1397139
H	1.6384353	0.6224267	0.8829365
C	-1.6715783	-1.9487790	-3.2805953
C	0.5454812	-0.4626421	4.6367628
C	-0.3365015	1.8299295	4.8421780
C	-0.9622950	2.9100876	5.7366718
C	-0.0506394	0.5595317	5.3806756
H	-0.2929786	0.3773785	6.4263502
C	0.9282466	-1.8205780	5.2488695
C	-2.2695223	2.3747472	6.3614715
H	-2.9836429	2.0797502	5.5805533
H	-2.7399546	3.1504006	6.9839909
H	-2.0898568	1.4988230	6.9992385
C	2.4651335	-1.9760286	5.1726910
H	2.8213936	-1.9642043	4.1333327
H	2.7761824	-2.9297393	5.6256689
H	2.9660330	-1.1582765	5.7104881
C	-1.2899660	4.1947849	4.9563461
H	-0.3879035	4.6553996	4.5285183
H	-1.7487142	4.9307427	5.6320339
H	-1.9977536	4.0053388	4.1382124
C	0.0350002	3.2668721	6.8629168
H	0.2774588	2.3907052	7.4803263
H	-0.3913422	4.0380772	7.5226725
H	0.9738405	3.6538410	6.4417171
C	0.2633451	-2.9718347	4.4637302
H	-0.8305771	-2.9319363	4.5606654
H	0.6030898	-3.9430294	4.8532482
H	0.5097335	-2.9365516	3.3933617
C	0.5029776	-1.9422530	6.7218014
H	0.9954135	-1.1882729	7.3529825
H	0.7875286	-2.9319692	7.1067081
H	-0.5856848	-1.8394411	6.8420202
N	0.9899251	-2.6385080	-1.3486158
N	2.6055692	-0.5854784	-2.0901146
C	1.9981785	-2.9280243	-2.1951280
C	2.8558552	-1.7883775	-2.6399421
C	3.8783972	-1.9659403	-3.5892673
H	4.0613978	-2.9348785	-4.0493241
C	0.1983578	-3.6237286	-0.8898633
O	-4.6769285	1.6299768	1.9057774

C	3.3502997	0.4749951	-2.4467144
C	4.6422261	-0.8693835	-3.9713063
H	5.4154397	-0.9785152	-4.7327345
C	2.2419015	-4.2416525	-2.6287445
H	3.0624910	-4.4773555	-3.3025006
C	4.3787112	0.3718431	-3.3975202
H	4.9589109	1.2460380	-3.6882661
C	-0.8092944	-3.2202956	0.1580173
H	-1.1289516	-2.1886908	-0.0462027
H	-0.2642108	-3.1639369	1.1198058
C	-4.1541898	0.3703097	1.8551445
C	1.3978375	-5.2577826	-2.1929382
H	1.5504929	-6.2815402	-2.5373745
C	-3.2831594	-2.3115188	1.6549335
C	0.3595616	-4.9527797	-1.3177746
H	-0.3097679	-5.7341871	-0.9628049
C	-2.6458160	-1.3996140	2.5010805
H	-1.7934414	-1.7228186	3.0989383
C	-3.0667779	-0.0729849	2.6183559
H	-2.5296893	0.5934894	3.2907953
C	-2.8430693	-3.7545640	1.5864677
H	-3.7250523	-4.4131941	1.6400898
H	-2.2297305	-3.9823004	2.4738314
C	-4.3468257	-1.8357576	0.8701405
H	-4.8516578	-2.5152002	0.1791333
C	-2.0353845	-4.1165347	0.3259449
H	-1.7421971	-5.1751876	0.4108431
H	-2.6640131	-4.0251835	-0.5725441
C	-4.7778765	-0.5171355	0.9627969
H	-5.6078594	-0.1480581	0.3594981
C	3.0183024	1.7642231	-1.7358178
H	3.4144391	1.6906142	-0.7063609
H	1.9242848	1.8096898	-1.6231498
O	-2.2962966	4.9813929	-0.2550651
C	-4.6410721	3.9427374	2.4776385
H	-4.3316881	4.6445465	3.2711453
H	-5.7388423	3.8816246	2.5225011
C	1.3805403	4.8690479	0.1050095
H	2.1546877	5.0408371	0.8578087
C	1.7550224	4.4738573	-1.1835536
C	0.7302190	4.2546408	-2.1201840
H	0.9712411	3.9081058	-3.1267218
C	-0.6065553	4.4289570	-1.7873700
H	-1.3971633	4.2069009	-2.5026520
C	-0.9630638	4.8398174	-0.4944047
C	0.0410456	5.0582304	0.4590545
H	-0.2123017	5.3674254	1.4722645
C	3.5316662	3.0503605	-2.3833753
H	3.0992619	3.1464142	-3.3931149
H	4.6235891	3.0064559	-2.5218732
C	-2.7376634	4.8872901	1.1089479
H	-2.6121838	5.8614189	1.6185889
H	-2.1169046	4.1375201	1.6237881
C	-4.1943525	4.4476026	1.0982749
H	-4.2909938	3.6310612	0.3683141
H	-4.8341370	5.2764567	0.7604532
C	4.2949092	-4.3442230	-8.0214805
H	4.3633598	-3.8686451	-9.0069410
H	4.0594203	-5.4068367	-8.1162071
C	3.2135379	4.3006598	-1.5466445
H	3.5679449	5.1888729	-2.0981601
H	3.8066511	4.2656650	-0.6182592
C	-4.0688412	2.5693437	2.8009060
H	-4.2917820	2.2797041	3.8437948
H	-2.9742819	2.5529739	2.6636635
H	5.2564858	-4.2092580	-7.5099497

4c

O	3.5271096	-1.9504158	-8.7063031
O	1.4284828	-5.9305375	-7.8297055
O	-1.3387897	1.4514055	-4.8577081
N	2.4501777	-3.9255247	-8.3085990
O	-3.1610062	-2.5815319	-3.6498877
N	-2.2095019	-0.5645808	-4.2045910
N	-0.5777390	0.9105590	-0.6092652
C	0.4875150	-2.5663689	-6.8051896
C	2.5665466	-2.5274745	-8.2031826
N	-2.5778577	1.4881601	-1.1340815
C	1.5086452	-1.8238350	-7.4433548
C	0.4127025	-3.9732387	-6.9337580
N	-1.6222492	1.6951135	-0.2561585
C	-0.3669641	-0.4854797	-5.8353608
C	1.4357956	-4.7081868	-7.7169071
C	0.6218567	0.2263238	-6.5021120
H	0.6642959	1.3070032	-6.3670128
C	-1.4302357	-2.6347131	-5.2783214
C	-1.5063962	-4.0116236	-5.4473642
H	-2.2657414	-4.5629172	-4.8943868
C	-0.4511062	-1.8896721	-5.9760169
C	-2.1514129	0.5717987	-2.0471160
C	1.5641840	-0.4426866	-7.2975385
H	2.3714682	0.0999720	-7.7880132
C	-1.3202630	0.2303630	-4.9560201
C	-0.8615259	0.1877334	-1.7238583
H	-0.1465420	-0.5026077	-2.1717544
C	-0.5911154	-4.6771479	-6.2779434
H	-0.6251072	-5.7603361	-6.3921958
C	-3.0142759	0.1348112	-3.1883842
H	-3.7904477	-0.5621543	-2.8484373
H	-3.4858495	1.0120115	-3.6488351
C	0.6884177	0.9940202	0.1069385
H	1.1183656	1.9894137	-0.0931848
H	1.3513158	0.2418946	-0.3426565
C	-2.3407270	-1.9590885	-4.3176509
N	0.4576123	-2.8658161	-2.5786055
N	2.0120917	-0.7872568	-3.4066005
C	1.4651869	-3.1501108	-3.4290691
C	2.3033198	-2.0051461	-3.9003569
C	3.3513575	-2.1936408	-4.8207267
H	3.5727444	-3.1747844	-5.2356013
C	-0.3110688	-3.8599869	-2.0993046
O	-5.4625188	1.3074779	0.3336346
C	2.7372086	0.2781073	-3.7915315
C	4.0957154	-1.0943917	-5.2311640
H	4.8879750	-1.2138441	-5.9707580
C	1.7270431	-4.4653385	-3.8482765
H	2.5368562	-4.6950700	-4.5368514
C	3.7884425	0.1620774	-4.7149541
H	4.3518292	1.0382804	-5.0308058
C	-1.3448395	-3.4680573	-1.0697548
H	-1.6637969	-2.4359903	-1.2739196
H	-0.8289084	-3.4258009	-0.0917571
C	-4.9152041	0.0584571	0.3629530
C	0.9162037	-5.4933176	-3.3796141
H	1.0886384	-6.5195657	-3.7065168
C	-3.9493468	-2.5993475	0.2780909
C	-0.1163823	-5.1943801	-2.4966445
H	-0.7596169	-5.9849422	-2.1148877
C	-3.4580347	-1.6761405	1.2057730
H	-2.6910700	-1.9910671	1.9178045
C	-3.9272990	-0.3612219	1.2630535
H	-3.5103757	0.3246293	1.9969935
C	-3.4546552	-4.0258484	0.2542768
H	-4.3150997	-4.7149227	0.2687138
H	-2.8856842	-4.2224931	1.1780891
C	-4.9234725	-2.1532275	-0.6303529
H	-5.3085550	-2.8405725	-1.3862334
C	-2.5702218	-4.3775196	-0.9564541
H	-2.2683170	-5.4315592	-0.8493877
H	-3.1520680	-4.2995168	-1.8870173
C	-5.4044215	-0.8492225	-0.5912637
H	-6.1663873	-0.5058092	-1.2920755
C	2.3554360	1.5890311	-3.1469667
H	2.7014850	1.5572520	-2.0978933
H	1.2564122	1.6292320	-3.0861951
O	-3.0239312	4.6240870	-1.8893182
C	-5.4866069	3.6491405	0.7765532

H	-5.2320140	4.3992037	1.5445896
H	-6.5847005	3.5788534	0.7567742
C	0.6427324	4.5610142	-1.3971964
H	1.3849067	4.6739285	-0.6018807
C	1.0729893	4.2901687	-2.6999432
C	0.0892062	4.1535085	-3.6931780
H	0.3771559	3.9121012	-4.7179745
C	-1.2617035	4.2739991	-3.3969798
H	-2.0194808	4.1153081	-4.1626672
C	-1.6762653	4.5460658	-2.0847376
C	-0.7118921	4.6985462	-1.0792880
H	-1.0056759	4.8976149	-0.0508984
C	2.8869692	2.8578829	-3.8141930
H	2.4811344	2.9261645	-4.8371306
H	3.9825090	2.8123345	-3.9209951
C	-3.5135140	4.5393489	-0.5408924
H	-3.4204136	5.5248422	-0.0461705
H	-2.9069534	3.8105910	0.0165222
C	-4.9640068	4.0843573	-0.6007374
H	-5.0192153	3.2303922	-1.2903085
H	-5.5905307	4.8886530	-1.0146116
C	3.5051687	-4.6031854	-9.0708244
H	3.5646878	-4.1705034	-10.0766006
H	3.2581492	-5.6663084	-9.1177349
C	2.5418714	4.1315487	-3.0215252
H	2.9014434	5.0057570	-3.5912592
H	3.1129349	4.1269959	-2.0787605
C	-4.9268352	2.3029796	1.2195934
H	-5.2249209	2.0701657	2.2581824
H	-3.8265192	2.2937872	1.1615898
C	0.5098443	0.7692454	1.6071462
H	-0.2049139	1.5049858	2.0004862
H	0.0832112	-0.2288989	1.7869461
C	1.8291520	0.9118253	2.3500304
H	2.2961165	1.8938711	2.1201335
H	2.5490747	0.1285146	2.0317275
O	1.5641156	0.8103253	3.7435981
C	2.7451198	0.9746013	4.5402877
H	3.4622064	0.1573693	4.3273858
H	3.2410107	1.9306456	4.2751109
C	2.3476088	0.9731394	5.9910558
C	2.4272665	-0.2026077	6.7496330
C	1.8518848	2.1375697	6.5752676
C	2.0201637	-0.2263212	8.0862396
H	2.8116346	-1.1021695	6.2686729
C	1.4269245	2.1588066	7.9134496
H	1.7904304	3.0406287	5.9648650
C	1.5250199	0.9684209	8.6411902
H	1.2067150	0.9602142	9.6824733
C	0.8772835	3.4603284	8.5176132
C	1.9637643	4.5565551	8.4429988
H	2.8582133	4.2573718	9.0085014
H	2.2707409	4.7545530	7.4069260
H	1.5859350	5.4988981	8.8676620
C	0.4590084	3.2970868	9.9888200
H	0.0725452	4.2532153	10.3699224
H	-0.3370526	2.5473069	10.1063821
H	1.3073182	3.0049717	10.6252557
C	-0.3617930	3.9092883	7.7101988
H	-1.1482162	3.1412771	7.7436233
H	-0.7712140	4.8418182	8.1269128
H	-0.1163356	4.0909390	6.6548724
C	2.0873874	-1.4904692	8.9571455
C	2.9972841	-1.2235607	10.1773101
H	2.6175722	-0.3994464	10.7972788
H	3.0589764	-2.1207460	10.8119745
H	4.0145360	-0.9595733	9.8537570
C	0.6649483	-1.8492598	9.4438526
H	-0.0023283	-2.0367908	8.5904468
H	0.6913417	-2.7557100	10.0673529
H	0.2244312	-1.0411841	10.0438439
C	2.6524384	-2.6990029	8.1918263
H	2.0293155	-2.9595861	7.3238466
H	3.6781911	-2.5154700	7.8398636
H	2.6808745	-3.5754075	8.8552137
H	4.4753305	-4.4560098	-8.5790256

5

O	3.1002815	-3.0099743	1.4370829
O	2.4736917	1.5240612	1.7259979
N	0.4532320	-0.4359642	5.1648204
N	2.8328746	-0.7317933	1.5652669
N	1.3355129	-1.1543659	5.9222155
N	2.4076385	-1.3390066	5.1939692
O	0.1159789	1.6353368	-4.9360843
N	0.6084147	-0.6028859	-5.1570030
C	-0.8257143	-0.0605063	5.6649821
O	0.8799529	-2.8725453	-5.2944030
C	2.2395994	-0.7412985	3.9743519
C	0.9895878	-0.1576870	3.9441500
H	0.4851564	0.4581767	3.2116600
C	1.8887857	-0.6951024	-1.1002865
C	-2.1798353	0.7680527	7.4842567
C	2.2815162	-1.9251980	-0.5192287
C	2.7667818	-1.9690176	0.8863847
C	-0.9361793	0.3707315	6.9911508
H	-0.0363244	0.3894746	7.6008236
C	-1.9310620	-0.1171376	4.8218285
H	-1.8044561	-0.4973529	3.8081283
C	-3.2864334	0.7202447	6.6141945
H	-4.2548346	1.0366706	6.9967654
C	1.9433506	0.5125636	-0.3637836
C	1.4288191	-0.6706670	-2.4488856
C	2.4288725	0.5075810	1.0389111
C	3.3345523	-0.7343934	2.9532417
H	3.9567895	0.1633683	3.0699090
H	3.9530554	-1.6284347	3.0830460
C	-2.3720350	1.2654651	8.9249416
C	-3.1929519	0.2836360	5.2894725
C	2.2160250	-3.0963979	-1.2654275
H	2.5213032	-4.0308195	-0.7943968
C	1.0198736	0.5559251	-3.0236330
C	-4.4066571	0.1933148	4.3521884
C	1.3840834	-1.8742944	-3.1916296
C	1.7715752	-3.0704845	-2.5992744
H	1.7274718	-3.9838636	-3.1925615
C	1.0747119	1.7268420	-2.2768100
H	0.7549139	2.6576389	-2.7448503
C	1.5395142	1.7060357	-0.9509454
H	1.5933044	2.6209609	-0.3607926
C	-1.0566664	1.2649907	9.7220638
H	-0.6266758	0.2561925	9.8003622
H	-1.2444312	1.6274175	10.7428213
H	-0.3049986	1.9276399	9.2687927
C	-2.9215713	2.7098249	8.8952755
H	-2.2225466	3.3821080	8.3775196
H	-3.0628152	3.0832472	9.9205852
H	-3.8902012	2.7676922	8.3804795
C	-4.1263733	0.9937955	3.0607798
H	-3.9317571	2.0515207	3.2891430
H	-4.9941658	0.9408059	2.3867086
H	-3.2576222	0.6014746	2.5138119
C	-3.3803162	0.3446405	9.6490238
H	-4.3614870	0.3453139	9.1546553
H	-3.5265931	0.6832870	10.6857025
H	-3.0136909	-0.6913302	9.6731119
C	0.5400930	0.6023619	-4.4284847
C	0.2199013	-0.5696954	-11.6090553
H	-0.1210801	-0.5120698	-12.6435097
C	0.1578373	-1.7803719	-10.9143603
H	-0.2321105	-2.6740187	-11.4040345
C	-4.6509790	-1.2907367	3.9953384
H	-3.7816745	-1.7356517	3.4921403
H	-5.5165645	-1.3846719	3.3223508
H	-4.8530801	-1.8801356	4.9011737
C	0.9444938	-1.8555449	-4.6106379
C	0.7297352	0.5639764	-10.9713651
H	0.7882237	1.5123925	-11.5077788
C	1.1164652	-0.7191984	-8.9424830
C	0.2951540	-0.5668580	-6.5969574
H	-0.4607043	-1.3332568	-6.8084573
H	-0.1329126	0.4187314	-6.8041508
C	1.1741174	0.4895930	-9.6490395
H	1.5868089	1.3780103	-9.1700769
C	0.6018990	-1.8511423	-9.5924871
H	0.5543768	-2.7997851	-9.0524457

C	-5.6860787	0.7543296	4.9955208
H	-5.9744614	0.1908314	5.8946720
H	-6.5184965	0.6815470	4.2812835
H	-5.5731560	1.8133132	5.2701228
C	5.0656747	1.4553465	-6.4183851
H	5.9595748	2.0213176	-6.1530180
C	3.8197726	2.0854413	-6.4604760
H	3.7354172	3.1472442	-6.2241524
C	2.6734215	1.3620045	-6.7989191
H	1.7082717	1.8681805	-6.7999263
C	2.7508542	-0.0065529	-7.1019833
C	4.0076461	-0.6271261	-7.0483557
H	4.0835259	-1.6934280	-7.2730515
C	5.1559696	0.0928332	-6.7132793
H	6.1220545	-0.4130579	-6.6781305
C	1.5396900	-0.8387222	-7.4821542
H	1.8114938	-1.8909408	-7.3160271

6

O	-3.2860540	-2.1437311	1.5468062
O	-3.0054169	2.4057426	1.8838240
N	1.0301387	-0.2731048	2.3964464
N	-3.1430470	0.1297714	1.7352158
N	0.9836087	-0.1821446	3.7527497
N	-0.2739274	-0.0686088	4.0974765
C	2.2849244	-0.3811138	1.7307886
C	-1.0489074	-0.0900045	2.9734099
C	-0.2276651	-0.2203714	1.8686655
H	-0.4082259	-0.2734711	0.7971517
C	4.6105382	-1.0125581	1.8387882
C	-3.3851366	-1.0476415	1.0104604
C	3.3524763	-0.9470455	2.4343559
H	3.1751785	-1.3097332	3.4428822
C	2.4305495	0.1083976	0.4331241
H	1.5673778	0.5485515	-0.0694360
C	4.7516579	-0.5156813	0.5317936
H	5.7317749	-0.5755955	0.0623544
C	-3.3219920	1.4245845	1.2256045
C	-2.5408554	-0.0028933	3.0673730
H	-2.9504058	-0.9093464	3.5307449
H	-2.8291296	0.8716578	3.6621341
C	5.8216455	-1.6289595	2.5526454
C	3.6907403	0.0434817	-0.1885370
C	3.8774862	0.5255414	-1.6378439
C	5.5183962	-1.9534678	4.0232129
H	5.2292428	-1.0493379	4.5799084
H	6.4151172	-2.3702969	4.5043613
H	4.7088254	-2.6874666	4.1322173
C	6.2068180	-2.9371885	1.8260911
H	5.3664594	-3.6466076	1.8411857
H	7.0696778	-3.4104049	2.3189336
H	6.4729133	-2.7514014	0.7756695
C	3.2831025	-0.5454905	-2.5759532
H	3.7730583	-1.5182049	-2.4242163
H	3.4075501	-0.2529946	-3.6300548
H	2.2127959	-0.6717184	-2.3830251
C	7.0124977	-0.6460191	2.5135480
H	7.3296833	-0.4168537	1.4871686
H	7.8760251	-1.0785912	3.0405829
H	6.7523526	0.3027858	3.0054657
C	3.1442811	1.8595632	-1.8780360
H	2.0681106	1.7835103	-1.6807462
H	3.2693325	2.1718689	-2.9258590
H	3.5537080	2.6532786	-1.2370967
C	5.3600349	0.7307558	-1.9996974
H	5.8491687	1.4385177	-1.3144888
H	5.4347334	1.1388466	-3.0181055
H	5.9259119	-0.2112100	-1.9856460
O	2.4906336	-3.3408994	5.1175543
O	3.2615121	0.7956324	6.0817973
N	-0.2881990	-0.8800440	-1.5743622
N	-0.3720973	1.7779837	-0.8418061
C	-0.4794230	0.0586807	-2.5242675
C	-0.8403756	1.4238680	-2.0571727
C	0.1396567	-4.4885896	1.8400129
C	1.6702339	-3.6820953	4.0745508
C	0.1241984	-2.1062907	-1.9435492
C	-0.6184133	3.0125159	-0.3732187
C	-0.4151873	-3.6856611	2.8449651
H	-1.4519346	-3.3532003	2.7549684

C	0.3156669	-2.4475753	-3.2930660
H	0.6497453	-3.4518520	-3.5551500
C	-0.0034938	3.3878152	0.9462566
H	0.0199158	2.5109112	1.6083548
H	-0.6273525	4.1497431	1.4376267
C	1.4845334	-4.8658655	1.9726291
H	1.9462899	-5.4835674	1.1986424
C	2.2468431	-4.4704540	3.0688902
H	3.2928048	-4.7627548	3.1669402
C	2.3445347	3.5848271	3.0889062
C	-0.2851109	-0.2038552	-3.8879780
H	-0.4101429	0.5867559	-4.6265153
C	0.3285984	-3.2901969	3.9579049
H	-0.1369804	-2.6606474	4.7121237
C	0.3729036	-3.1033653	-0.8495554
H	0.8818915	-2.6019733	-0.0126541
H	1.0530279	-3.8881555	-1.2175729
C	1.4981930	3.4471621	4.1934930
H	0.5969804	4.0610430	4.2567475
C	-0.6800034	-4.8977426	0.6399562
H	-1.6558084	-5.2881218	0.9701677
H	-0.1698002	-5.7209035	0.1130370
C	1.7693603	2.5402603	5.2211247
H	1.0684472	2.4449206	6.0470485
C	0.1111937	-1.4822990	-4.2729257
H	0.2777468	-1.7157763	-5.3250548
C	2.9144114	1.7379219	5.1493959
C	-1.6031616	2.2933863	-2.8508970
H	-2.0381450	1.9576529	-3.7921113
C	-1.3536218	3.9485166	-1.1243583
H	-1.5403220	4.9423640	-0.7166159
C	-0.9310257	-3.7442679	-0.3481626
H	-1.5118165	-4.1212470	-1.2072107
H	-1.5463763	-2.9754504	0.1390026
C	-1.8518207	3.5793594	-2.3701927
H	-2.4537456	4.2760146	-2.9548213
C	1.4226500	3.9307403	0.7285287
H	1.4030756	4.6787125	-0.0828010
H	2.0618670	3.1056181	0.3816086
C	2.0458451	4.5673617	1.9824194
H	2.9794985	5.0713838	1.6835750
H	1.3735042	5.3557028	2.3591934
C	3.7787524	1.8665441	4.0525152
H	4.6573024	1.2249079	3.9985675
C	3.4878085	2.7691015	3.0367044
H	4.1591982	2.8346152	2.1773939
C	3.1479103	-1.9449491	6.9469696
H	3.4936104	-2.7908697	7.5601365
H	3.9813578	-1.6567477	6.2943388
C	2.7922376	-0.7546127	7.8502363
H	3.6804915	-0.4779731	8.4387383
H	2.0082592	-1.0343147	8.5734974
C	1.9889340	-2.3799393	6.0673579
H	1.5829956	-1.5208671	5.5143126
H	1.1769250	-2.8333123	6.6663686
C	2.2926338	0.4709717	7.0904493
H	2.1664285	1.3235091	7.7823944
H	1.3184095	0.2760430	6.6092869
C	-3.6724550	-0.8941161	-0.4615461
H	-2.6834859	-0.8297385	-0.9516078
H	-4.1343516	-1.8354214	-0.7795236
C	-3.8986110	1.5721236	-0.1622676
H	-3.0659754	1.9322851	-0.7800816
H	-4.6043260	2.4096872	-0.0984086
C	-4.5161257	0.3321266	-0.8220234
C	-4.6676459	0.5243843	-2.3310498
C	-5.0013910	1.7880460	-2.8497241
C	-4.4821809	-0.5296556	-3.2257541
C	-5.1215244	2.0095776	-4.2219687
H	-5.1463956	2.6179974	-2.1622620
C	-4.6312949	-0.3615050	-4.6143390
H	-4.2183329	-1.5153350	-2.8456671
C	-4.9333340	0.9161552	-5.0875147
C	-5.4283188	3.3940200	-4.8140400
C	-4.4731103	-1.5761652	-5.5427208
H	-5.0341418	1.0765369	-6.1595577
C	-5.6496495	4.4619880	-3.7298909
C	-4.2388022	3.8349889	-5.6972384
C	-6.7057027	3.3165660	-5.6795822
C	-3.0352171	-2.1226983	-5.4260122

C	-5.4776318	-2.6724440	-5.1202991
C	-4.7380351	-1.2270245	-7.0169866
H	-6.5050971	4.2152334	-3.0848467
H	-4.7642639	4.5896533	-3.0904488
H	-5.8580145	5.4319874	-4.2031656
H	-4.0661304	3.1322985	-6.5236262
H	-4.4289528	4.8281084	-6.1316737
H	-3.3119803	3.8915284	-5.1080216
H	-6.5928762	2.6093938	-6.5124426
H	-7.5652074	2.9924969	-5.0756482
H	-6.9375895	4.3032330	-6.1079410
H	-2.3100025	-1.3668918	-5.7570104
H	-2.7767153	-2.3931581	-4.3932967
H	-2.9122110	-3.0187622	-6.0528472
H	-5.3919409	-3.5442789	-5.7863049
H	-5.2977241	-3.0182884	-4.0933753
H	-6.5096897	-2.2974695	-5.1738717
H	-5.7588275	-0.8469053	-7.1688449
H	-4.0282037	-0.4759590	-7.3936541
H	-4.6233831	-2.1284672	-7.6353581
H	-5.5249411	0.1801615	-0.3966246

13

N	2.1391187	-1.2691109	-6.0793742
N	3.8410625	-2.1810937	-5.1431457
N	3.4236228	-1.6440340	-6.2664306
C	2.8201453	-2.1661559	-4.2422882
C	1.1549747	-1.6418093	-8.2965433
C	1.7112466	-1.5797601	-4.8291112
H	0.7066813	-1.3490430	-4.4721850
C	-0.0699987	-2.2871037	-8.4405152
H	-0.8754568	-2.0444289	-7.7440895
C	2.2115433	-1.9423617	-9.1689491
H	3.1622467	-1.4346066	-9.0140860
C	2.9838382	-2.7163664	-2.8631703
C	1.3813841	-0.6579407	-7.1716990
H	1.9526355	0.2199119	-7.5092357
H	0.4344474	-0.3129198	-6.7388968
C	-0.2742779	-3.2377704	-9.4580271
C	2.0572962	-2.8948627	-10.1763519
C	3.1890073	-3.2661711	-11.1465980
C	0.8036453	-3.5262542	-10.2985504
H	0.6722440	-4.2629588	-11.0892314
C	-1.6549906	-3.8921904	-9.6263547
C	3.4773398	-4.7804889	-11.0420773
H	3.7638994	-5.0582631	-10.0182596
H	4.2995336	-5.0599105	-11.7179476
H	2.5995521	-5.3817280	-11.3157354
C	-2.7010101	-2.7862904	-9.8971961
H	-2.7649136	-2.0738482	-9.0628763
H	-3.6982269	-3.2299092	-10.0375636
H	-2.4427820	-2.2210482	-10.8043165
C	4.4887647	-2.5011402	-10.8453423
H	4.3539340	-1.4143089	-10.9435898
H	5.2698431	-2.8046942	-11.5570269
H	4.8610603	-2.7101409	-9.8329754
C	2.7526301	-2.9268963	-12.5897921
H	1.8495395	-3.4804755	-12.8823214
H	3.5510655	-3.1857006	-13.3015360
H	2.5370078	-1.8532057	-12.6885699
C	-2.0361747	-4.6500388	-8.3367976
H	-1.3327655	-5.4722937	-8.1455504
H	-3.0455055	-5.0791206	-8.4267198
H	-2.0301067	-3.9907169	-7.4580007
C	-1.6934378	-4.8910328	-10.7950330
H	-1.4693022	-4.4044963	-11.7555398
H	-2.6987232	-5.3292586	-10.8720432
H	-0.9819802	-5.7169529	-10.6489282
N	-1.4059020	-1.9572124	-3.5027244
N	-0.8376129	0.6857536	-3.8089198
C	-2.1735392	-1.0789035	-2.8282765
C	-1.7838884	0.3599176	-2.9084594
C	-2.3772925	1.3261582	-2.0770199
H	-3.1137651	1.0502964	-1.3250884
C	-1.7180959	-3.2652146	-3.4887272
O	5.2011113	-4.8451599	-6.3052386
C	-0.4503482	1.9674136	-3.9253806
C	-1.9746731	2.6516804	-2.1902901
H	-2.3959451	3.4098804	-1.5292415
C	-3.2952196	-1.4930035	-2.0912293

H	-3.9234871	-0.7777294	-1.5649923
C	-0.9985050	2.9838773	-3.1266056
H	-0.6620901	4.0145637	-3.2276536
C	-0.8686458	-4.1452077	-4.3705645
H	0.1597730	-3.7586952	-4.3562543
H	-1.2122167	-3.9886363	-5.4103127
C	3.8921934	-5.1635481	-6.0837628
C	-3.5973746	-2.8496322	-2.0389662
H	-4.4500520	-3.1992947	-1.4551553
C	1.2558716	-5.9824421	-5.4665067
C	-2.8025074	-3.7521646	-2.7406269
H	-3.0241378	-4.8177356	-2.7155991
C	1.5418949	-5.2729433	-6.6372547
H	0.7381382	-5.0224720	-7.3324490
C	2.8394252	-4.8668793	-6.9586256
H	3.0021574	-4.3059119	-7.8756850
C	-0.1452397	-6.4519595	-5.1539363
H	-0.1230686	-7.5112777	-4.8503333
H	-0.7497007	-6.4049807	-6.0739466
C	2.3197587	-6.2379202	-4.5850732
H	2.1260140	-6.7670861	-3.6489722
C	-0.8591234	-5.6395731	-4.0569428
H	-1.8854355	-6.0312544	-3.9670239
H	-0.3664876	-5.7917824	-3.0843944
C	3.6180564	-5.8371581	-4.8820474
H	4.4445952	-6.0439673	-4.2013847
C	0.6014365	2.2284064	-4.9756817
H	0.1305399	2.1039486	-5.9673535
H	1.3454883	1.4193141	-4.9027192
O	6.4142532	-0.3183530	-5.7975533
C	6.9626528	-3.6659949	-7.4010284
H	7.2981260	-3.3307299	-8.3975509
H	7.5980983	-4.5219607	-7.1272818
C	3.9927322	2.1370439	-7.1305380
H	3.6192311	2.5101351	-8.0881374
C	3.4674562	2.6575491	-5.9424615
C	3.9712381	2.1472229	-4.7336412
H	3.5700983	2.4968558	-3.7806862
C	4.9505794	1.1618119	-4.7110481
H	5.2885468	0.7290462	-3.7704975
C	5.4702160	0.6580500	-5.9120672
C	4.9874443	1.1552502	-7.1305880
H	5.3685876	0.7716700	-8.0757435
C	1.3016389	3.5853188	-4.9117081
H	1.7302770	3.7261119	-3.9056400
H	0.5747373	4.4016595	-5.0501138
C	6.5806498	-1.2164778	-6.9078713
H	7.2711869	-0.7755381	-7.6516270
H	5.5999570	-1.3688546	-7.3862909
C	7.1134637	-2.5376338	-6.3700583
H	6.5309487	-2.7907874	-5.4724987
H	8.1649285	-2.4229274	-6.0682467
C	2.4015134	3.7329630	-5.9748407
H	2.8700936	4.7254921	-5.8564097
H	1.9354131	3.7382246	-6.9740728
C	5.5246328	-4.1531978	-7.5193539
H	5.4029546	-4.8402957	-8.3760548
H	4.8324724	-3.3055159	-7.6549492
O	3.1529644	-0.1326904	-2.0832873
O	0.9220341	-4.1240820	-1.6890533
O	-2.0047102	3.0486259	1.5450241
N	2.0059916	-2.1120072	-1.9428588
O	-3.6431443	-1.0607052	2.7570940
N	-2.7543544	0.9961975	2.2393801
N	-1.3665946	2.9597989	5.7086322
C	0.0779339	-0.8681126	-0.3053247
C	2.1616108	-0.7349594	-1.6829472
N	-3.3359892	3.3933085	4.9744372
C	1.0926437	-0.0828381	-0.8984546
C	0.0045263	-2.2628988	-0.5278973
N	-2.4367689	3.7713578	5.8537175
C	-0.8232495	1.1555095	0.7298051
C	0.9815140	-2.9261985	-1.4246479
C	0.1711133	1.9091883	0.1199130
H	0.1764546	2.9870280	0.2765518
C	-1.8626974	-1.0317623	1.1813254
C	-1.9133739	-2.4003505	0.9502666
H	-2.6785614	-2.9849308	1.4588251
C	-0.8755746	-0.2442198	0.5439626
C	-2.8505915	2.3284666	4.2788192

C	1.1359025	1.2890313	-0.6836682
H	1.9390356	1.8615960	-1.1449818
C	-1.8768821	1.8288201	1.5143929
C	-0.5335417	2.7514206	8.0073112
C	-1.5777270	2.0331704	4.7396419
H	-0.8308783	1.2904206	4.4577956
C	-0.9892941	-3.0125530	0.0883665
H	-1.0305001	-4.0827229	-0.1096567
C	-0.2029343	1.4988008	8.5165490
H	0.3336088	0.7967054	7.8745683
C	-1.2340316	3.6686735	8.8046149
H	-1.4944587	4.6312070	8.3670538
C	-3.6461482	1.6686814	3.1993349
H	-4.3147787	0.9015579	3.6100203
H	-4.2455551	2.4245865	2.6752816
C	-0.2004236	3.1150477	6.5783702
H	0.1297619	4.1606343	6.4911758
H	0.5837804	2.4669226	6.1685157
C	-2.8224377	-0.4036557	2.1219488
C	-0.5492608	1.1353525	9.8312585
C	-1.6174537	3.3388789	10.1052437
C	-2.3997914	4.3021992	11.0105253
C	-1.2602100	2.0661139	10.5929785
H	-1.5466361	1.8060239	11.6105036
C	-0.1164686	-0.2318854	10.3846260
C	-3.7316777	3.6404819	11.4312364
H	-4.3403802	3.3845476	10.5527790
H	-4.3142952	4.3246233	12.0661664
H	-3.5651113	2.7148978	11.9990626
C	1.4275087	-0.3094634	10.3668646
H	1.8258751	-0.2133998	9.3473185
H	1.7666279	-1.2750559	10.7717293
H	1.8645377	0.4940716	10.9769417
C	-2.7169284	5.6335287	10.3084820
H	-1.8009646	6.1696532	10.0196005
H	-3.2824337	6.2847538	10.9901565
H	-3.3268806	5.4869597	9.4065965
C	-1.5613935	4.6102287	12.2715886
H	-1.3417980	3.7004750	12.8474382
H	-2.1057702	5.3020100	12.9319672
H	-0.6032460	5.0754448	11.9982969
C	-0.6932484	-1.3613852	9.5041638
H	-1.7918695	-1.3512673	9.5254994
H	-0.3543822	-2.3421287	9.8699370
H	-0.3747737	-1.2671187	8.4572660
C	-0.5958003	-0.4606165	11.8281526
H	-0.1779737	0.2846077	12.5208651
H	-0.2678474	-1.4521171	12.1713830
H	-1.6928443	-0.4291635	11.9037504
N	-0.1535405	-1.0009957	4.0574733
N	1.5373724	0.9848660	3.2939111
C	0.8503192	-1.3370277	3.2235570
C	1.7485990	-0.2348161	2.7645227
C	2.7634266	-0.4612119	1.8169093
H	2.9113168	-1.4413183	1.3678156
C	-0.9842672	-1.9515527	4.5208547
O	-5.9002225	3.4188542	6.9317543
C	2.3136293	2.0140320	2.9151633
C	3.5649766	0.6023491	1.4181051
H	4.3320131	0.4559231	0.6568573
C	1.0503618	-2.6646330	2.8091863
H	1.8668063	-2.9371603	2.1443086
C	3.3412111	1.8607361	1.9704748
H	3.9485775	2.7111564	1.6647776
C	-1.9946435	-1.4913595	5.5420564
H	-2.3430660	-0.4886717	5.2587153
H	-1.4456606	-1.3389588	6.4899028
C	-5.3682900	2.1625957	6.9815470
C	0.1677186	-3.6439108	3.2518520
H	0.2859715	-4.6773279	2.9228498
C	-4.4649733	-0.5177835	6.9878195
C	-0.8669539	-3.2906223	4.1135054
H	-1.5686420	-4.0421073	4.4700166
C	-3.8447193	0.4604375	7.7714708
H	-2.9930663	0.1953732	8.4005879
C	-4.2836815	1.7864052	7.7838737
H	-3.7556947	2.5081046	8.4027184
C	-4.0088722	-1.9570999	7.0220492
H	-4.8837123	-2.6196969	7.1234440
H	-3.3939770	-2.1149277	7.9224819

C	-5.5289855	-0.1128116	6.1644032
H	-6.0202498	-0.8478516	5.5224306
C	-3.1960902	-2.4000137	5.7908328
H	-2.8740992	-3.4397884	5.9619854
H	-3.8306242	-2.4005644	4.8913682
C	-5.9765303	1.2040501	6.1549067
H	-6.8064930	1.5159542	5.5200562
C	2.0106379	3.3331267	3.5833266
H	2.4266140	3.3017062	4.6068500
H	0.9194205	3.3945616	3.7121035
O	-3.4087551	6.6211589	4.5799726
C	-5.8534391	5.7766258	7.2926681
H	-5.5687106	6.5393807	8.0377311
H	-6.9522010	5.7175855	7.3074901
C	0.2512203	6.6037715	5.1048397
H	0.9902331	6.8681394	5.8658140
C	0.6861742	6.0711410	3.8862001
C	-0.2954149	5.7359707	2.9379495
H	-0.0088948	5.2823611	1.9877353
C	-1.6475663	5.9275783	3.1915624
H	-2.4023156	5.6078340	2.4749159
C	-2.0639290	6.4757951	4.4132869
C	-1.1038072	6.8151125	5.3767098
H	-1.4042231	7.2306710	6.3374400
C	2.5238416	4.5837463	2.8686498
H	2.1199251	4.6066972	1.8430386
H	3.6194439	4.5459782	2.7629443
C	-3.9146473	6.6534121	5.9248637
H	-3.8401087	7.6785559	6.3343286
H	-3.3015543	5.9812962	6.5462306
C	-5.3589498	6.1716101	5.8930964
H	-5.4011820	5.2907823	5.2370304
H	-6.0041595	6.9482331	5.4561922
C	2.1623665	5.8803728	3.6095235
H	2.5431633	6.7345028	3.0226529
H	2.7080387	5.9064315	4.5669052
C	-5.2931124	4.4342677	7.7429012
H	-5.5227377	4.2403163	8.8064951
H	-4.1983029	4.4029195	7.6123036
H	3.9989273	-2.5019406	-2.5045222
H	2.8140900	-3.8002616	-2.8475190

Protonated catalyst structures

1H(+)

O	0.4878836	1.2512177	4.4818160
N	2.0215357	-2.4088483	-2.0951331
H	1.2055436	-1.8723471	-1.7449784
N	0.1874224	-0.9950464	-3.3188663
O	-2.3996188	4.6388393	1.7758598
C	1.1728887	-1.4440336	-4.1201365
C	2.5216555	-3.1676985	0.1963500
H	1.9461491	-4.0855330	0.4137672
H	1.8670454	-2.3252574	0.4683863
C	2.4349115	0.3272344	3.5632373
H	2.8456952	1.3370669	3.5707659
C	2.1671291	-2.3007606	-3.4450109
C	-0.7797632	-0.2159867	-3.8376388
C	2.6486310	-2.0546404	3.0909806
C	1.1334299	0.1288967	4.0585521
C	2.8123272	-3.1452616	-1.2754743
C	3.7875470	-3.1451073	1.0737947
H	4.4398446	-3.9936964	0.8195243
H	4.3577364	-2.2288462	0.8542395
C	3.1746630	-0.7497102	3.0934916
H	4.1918822	-0.5746321	2.7344210
C	0.5988496	-1.1670627	4.0836985
H	-0.3898209	-1.3598531	4.4953179
C	3.2090764	-3.0004121	-4.0482649
H	3.3539293	-2.9455136	-5.1240131
C	1.2633199	-1.1329437	-5.4808964
H	2.0780522	-1.5009654	-6.1020643
C	-3.9482025	1.8235382	-0.0779165
H	-4.4874999	0.8922927	0.1107326
C	1.3571599	-2.2357192	3.5926390
H	0.9304762	-3.2411147	3.6268499
C	-3.5470504	2.5995564	1.0133141
H	-3.7842249	2.2603816	2.0200210
C	-1.8744993	0.2327662	-2.9036110
H	-2.2529237	-0.6606980	-2.3788871
H	-1.4215772	0.8577233	-2.1143500
C	3.8548333	-3.8539635	-1.8762910
H	4.5103626	-4.4600190	-1.2563460
C	-0.7518260	0.1428886	-5.2009289
H	-1.5337149	0.7771601	-5.6127737
C	-3.0135944	3.4266508	-1.5943901
H	-2.8120778	3.7783821	-2.6088151
C	-2.8511346	3.7979601	0.7963015
C	-3.6929085	2.2112537	-1.3963701
C	-2.5918939	4.2057886	-0.5231254
H	-2.0637651	5.1464352	-0.6808254
C	0.2736743	-0.3151054	-6.0220617
H	0.3024834	-0.0397423	-7.0764221
C	4.0489380	-3.7808793	-3.2554259
H	4.8627249	-4.3401794	-3.7175510
C	3.4652985	-3.2170207	2.5776794
H	2.9367178	-4.1608859	2.7866409
H	4.4218428	-3.2715475	3.1213391
C	-4.1487149	1.3607600	-2.5611529
H	-4.9423926	1.8855724	-3.1181739
H	-4.6068645	0.4366551	-2.1749112
C	-1.4148987	3.1057837	3.4191636
H	-1.5631314	2.2859277	2.6984387
H	-0.4113037	3.5055835	3.2206010
C	-3.0329926	0.9929373	-3.5555612
H	-3.4718977	0.3883385	-4.3664730
H	-2.6505775	1.9146277	-4.0236796
C	-0.8802527	1.1478797	4.9133381
H	-0.9158178	0.7260418	5.9326877
H	-1.4349451	0.4738571	4.2363255
C	-2.4502765	4.1920190	3.1430160
H	-3.4699311	3.8512827	3.3973778
H	-2.2501080	5.0958515	3.7343306
C	-1.4924713	2.5377351	4.8432831
H	-2.5425673	2.4469987	5.1683923
H	-0.9964210	3.2071474	5.5618449

4aH(+)

O	2.1854242	-4.9563801	7.5585349
O	-2.2933465	-5.5260558	6.7652121
O	2.1686716	0.7899484	3.4534789
O	-2.3277005	0.2647089	2.7416854
N	-0.0785389	0.5038832	3.0840045
N	0.1091818	0.6664891	-1.4917321
N	-0.0775796	-5.2076508	7.2288302
N	-0.3718043	-0.3336169	0.3477169
N	-0.2980407	-0.5096317	-0.9563185
C	-1.3184093	-1.2376302	4.2867935
C	-1.3093207	-3.5386201	5.8998357
C	1.1051641	-0.9676993	4.6570423
C	-0.1080054	-1.6574860	4.8880824
C	1.1421816	0.1736847	3.7128137
C	2.2762989	-1.3948691	5.2731592
H	3.1946406	-0.8368686	5.0909492
C	1.1071434	-3.2622889	6.2874538
C	-0.0510064	1.5324025	2.0382587
H	0.8350245	2.1535312	2.2106996
H	-0.9559308	2.1464797	2.1400882
C	-0.1057103	-2.8189374	5.7103546
C	0.3143453	1.5904520	-0.5146486
H	0.6318725	2.6022823	-0.7211653
C	-1.2979499	-4.8179332	6.6564384
C	2.2778254	-2.5400810	6.0864777
H	3.1953980	-2.8951019	6.5554430
C	-1.3145967	-0.1082719	3.3310825
C	1.1459106	-4.5215475	7.0805791
C	-2.4949731	-3.0784040	5.3409369
H	-3.4092752	-3.6446231	5.5178827
C	-2.4994683	-1.9259777	4.5388694
H	-3.4187391	-1.5721952	4.0715160
C	-0.0044388	0.9449485	0.6603642
C	0.1763015	0.8631192	-2.9045905
C	-0.3736066	-0.1037980	-3.7535374
H	-0.8047241	-0.9976570	-3.3126249
C	-0.0930366	-6.4614014	7.9963958
H	0.8955167	-6.5995812	8.4400295
H	-0.3359150	-7.3018221	7.3342599
C	0.7365689	2.0354738	-3.4012330
H	1.1771074	2.7642514	-2.7228624
C	-0.3651767	0.1060796	-5.1330760
C	0.7456946	2.2765690	-4.7843893
C	1.3307594	3.5974247	-5.3052677
C	-0.9606007	-0.9085057	-6.1198231
C	0.1959361	1.3022785	-5.6216773
H	0.1946113	1.4723931	-6.6964507
C	1.3073680	3.6832557	-6.8402544
H	1.9015401	2.8816106	-7.3025611
H	1.7395045	4.6414225	-7.1607086
H	0.2841765	3.6347286	-7.2407498
C	2.7946906	3.7357798	-4.8313065
H	2.8804276	3.7470720	-3.7369616
H	3.2240851	4.6751140	-5.2094558
H	3.4075766	2.9061221	-5.2133434
C	0.4918564	4.7655131	-4.7380913
H	-0.5505071	4.7026880	-5.0827841
H	0.9043528	5.7283004	-5.0731685
H	0.4812665	4.7699288	-3.6392091
C	-2.1084108	-0.2361055	-6.9068616
H	-2.9017980	0.1039765	-6.2257943
H	-2.5488003	-0.9492176	-7.6191313
H	-1.7584661	0.6334007	-7.4795405
C	-1.5242930	-2.1514679	-5.4109532
H	-0.7473991	-2.6876896	-4.8470974
H	-1.9319696	-2.8465663	-6.1579245
H	-2.3403204	-1.8931905	-4.7198983
C	0.1394863	-1.3680030	-7.1031933
H	0.5499844	-0.5273910	-7.6781448
H	-0.2720852	-2.0941462	-7.8196060
H	0.9694207	-1.8484793	-6.5658229
O	3.0811622	3.3709852	-1.2916740
O	-0.5034615	4.9866989	-0.9268673
N	0.2550930	-3.0341202	1.9091144
N	-2.2472582	-2.3987528	1.1718105
C	-0.6952825	-3.9042785	2.2910690
C	-2.0604448	-3.5793760	1.8322082
C	4.1728394	-0.5140913	-0.1639961
C	1.5220666	-3.2352559	2.2948349

C	-1.5799474	4.1610144	-0.7294416
C	3.4595369	2.1252207	-0.8525783
C	-3.1640155	-4.4027811	2.0511892
H	-3.0398201	-5.3487722	2.5708524
C	2.5607285	-2.2688136	1.8110170
H	3.2854386	-2.0806893	2.6189218
H	2.0796967	-1.3124028	1.5679815
C	3.9454904	-0.1508047	-1.5015034
H	4.0509731	-0.9010393	-2.2884324
C	3.3081141	-2.8007142	0.5734416
H	2.6049336	-2.8468891	-0.2733183
H	3.6475591	-3.8320884	0.7649127
C	-0.4256019	-5.0242188	3.0911921
H	-1.2090145	-5.7071650	3.4142865
C	2.7986456	4.3844156	-0.3156535
H	3.7103555	4.6307969	0.2558788
H	2.0567169	3.9922740	0.4058389
C	4.0323698	0.4767660	0.8135458
H	4.2008107	0.2343061	1.8637480
C	-2.2203740	3.9669951	0.5028270
H	-1.9115845	4.5171730	1.3899064
C	4.5275185	-1.9342225	0.2038318
H	5.2289525	-1.9318708	1.0546129
H	5.0548685	-2.4131106	-0.6349641
C	3.6710799	1.7868822	0.4883367
H	3.5673810	2.5202153	1.2856432
C	2.2535733	5.5915178	-1.0725429
H	1.7323054	5.2142799	-1.9626012
H	3.0836454	6.2125564	-1.4364636
C	3.6042418	1.1504170	-1.8504655
H	3.4383850	1.4302977	-2.8899890
C	0.8916989	-5.2345612	3.4997296
H	1.1407646	-6.0840476	4.1359879
C	1.8743619	-4.3363995	3.1013675
H	2.9099378	-4.4676497	3.4158453
C	0.0347698	5.6925221	0.2026087
H	0.2860820	4.9679061	0.9993376
H	-0.7181871	6.3888981	0.6098717
C	1.2722867	6.4528464	-0.2651392
H	1.7579299	6.8827063	0.6256599
H	0.9559985	7.3006950	-0.8903379
C	-4.4196890	-4.0094967	1.5983330
H	-5.2840718	-4.6541112	1.7601048
C	-3.4567428	-1.9695613	0.7281685
C	-2.0187794	3.4543874	-1.8577463
H	-1.5064822	3.6046036	-2.8070504
C	-4.5655919	-2.7937199	0.9398401
H	-5.5385019	-2.4712355	0.5767320
C	-3.7450004	2.3597226	-0.5283020
C	-3.2891137	3.0700372	0.5881939
H	-3.7815606	2.9323199	1.5528690
C	-3.0809071	2.5634069	-1.7482868
H	-3.4079400	2.0205550	-2.6376300
C	-3.5670304	-0.6593427	0.0089513
H	-3.6835228	-0.8831099	-1.0671130
H	-2.6347823	-0.0974682	0.1099940
C	-4.7368133	0.2180727	0.4846116
H	-4.5277625	0.5375982	1.5156260
H	-5.6791026	-0.3499469	0.5068448
C	-4.9395308	1.4385342	-0.4291805
H	-5.2122299	1.0904720	-1.4384459
H	-5.8071791	2.0059479	-0.0555654
H	-1.4013453	-1.7694998	1.0119779
H	-0.8650112	-6.4008571	8.7723231

4bH(+)

O	7.1025053	-1.1735875	-6.5855502
O	4.0116154	0.1088065	-0.3671476
O	3.9006565	1.3416872	-8.6969230
O	0.7704618	2.5640194	-2.4761704
N	5.5457131	0.1289854	-7.6388144
N	-1.4330694	-0.3644732	0.2166689
N	2.4053260	1.3618972	-1.4227287
N	-0.6039654	-1.3166756	-0.2540987
N	0.5655961	-0.7405084	-0.4396732
C	3.7794662	1.2368269	-6.3259784
C	4.3418364	0.7040717	-5.1413669
C	3.7162194	0.9635499	-3.8892292
C	5.5099272	-0.0931984	-5.1834640
C	4.2796394	0.4259483	-2.7073584
C	6.0682739	-0.5756451	-4.0060190
H	6.9794230	-1.1708771	-4.0677790
C	6.1329318	-0.4299187	-6.4916414
C	2.5307478	1.7364170	-3.8512245
C	4.3882118	0.9335454	-7.6505840
C	5.4554288	-0.3136473	-2.7694270
H	5.8772876	-0.7010128	-1.8422685
C	0.4925826	0.5772057	-0.0875245
C	3.5935698	0.6109040	-1.4062056
C	2.6411615	2.0296780	-6.2573460
H	2.2344910	2.4379463	-7.1825615
C	2.0148000	2.2747522	-5.0228746
H	1.1061244	2.8730736	-4.9662299
C	1.8227191	1.9410532	-2.5662219
C	-0.7998582	0.8299541	0.3296641
H	-1.3052127	1.7362775	0.6398379
C	-2.5820889	-1.8562862	2.7248183
H	-1.9441253	-2.5717524	2.2059295
C	-3.1190509	-0.7875798	1.9952984
C	6.1856161	-0.2016778	-8.9207183
H	7.2447567	0.0766320	-8.8795740
H	5.6682748	0.3493272	-9.7092553
C	-2.8347028	-0.6731025	0.5142146
H	-3.0421633	-1.6177234	-0.0055298
H	-3.4410358	0.1214183	0.0593556
C	1.6632520	1.5036358	-0.1549266
H	1.3165050	2.5398474	-0.0698477
H	2.3683645	1.2805683	0.6559084
C	-2.8599679	-2.0003004	4.0887159
C	-3.9454988	0.1464193	2.6222596
H	-4.3527993	0.9636538	2.0273225
C	-4.2635777	0.0269140	3.9858286
C	-3.6970752	-1.0431530	4.6876371
H	-3.9346380	-1.1570687	5.7445126
C	-2.3239065	-3.1657168	4.9339896
C	-5.2866818	0.9502409	4.6643193
C	-3.5173166	-3.9585328	5.5155668
H	-4.1398770	-3.3387816	6.1746446
H	-3.1522728	-4.8108411	6.1069157
H	-4.1580576	-4.3467978	4.7109459
C	-1.4635324	-4.1359313	4.1085536
H	-2.0369176	-4.5959417	3.2905881
H	-1.0978930	-4.9459282	4.7546447
H	-0.5848091	-3.6364537	3.6784982
C	-1.4645090	-2.6089700	6.0920264
H	-0.5889742	-2.0643113	5.7109066
H	-1.1022758	-3.4329462	6.7238342
H	-2.0387672	-1.9252052	6.7318853
C	-5.4138130	2.2997045	3.9353892
H	-4.4415387	2.8086667	3.8728091
H	-6.1047695	2.9568800	4.4805235
H	-5.8139723	2.1852647	2.9181795
C	-6.6569341	0.2318460	4.6212908
H	-6.9605213	0.0296928	3.5841842
H	-7.4313807	0.8546336	5.0932373
H	-6.6137492	-0.7277151	5.1550111
C	-4.9171904	1.2305430	6.1355621
H	-4.9428513	0.3228919	6.7522437
H	-5.6382825	1.9357023	6.5719630
H	-3.9144983	1.6720731	6.2243731
O	0.5939626	1.4112844	3.9114330
N	2.3003149	-2.1613449	-2.2301358
H	1.5500104	-1.6081379	-1.7044243
N	0.3448610	-0.7955725	-3.5053227
O	-2.0069684	5.0116377	1.5956699

C	1.3034061	-1.3123130	-4.2902729
C	2.9935283	-2.8951596	0.0053494
H	2.4145399	-3.8081712	0.2380184
H	2.3546686	-2.0540256	0.3019797
C	2.6906203	0.5986022	3.2385484
H	3.0557425	1.6216265	3.3361483
C	2.3490903	-2.0923368	-3.5927570
C	-0.6580643	-0.0920685	-4.0586024
C	3.0768349	-1.7597579	2.7700531
C	1.3424008	0.3343118	3.5348143
C	3.1907106	-2.8581339	-1.4771902
C	4.2893493	-2.8761102	0.8277680
H	4.9239502	-3.7448652	0.5946466
H	4.8526416	-1.9697427	0.5631443
C	3.5381157	-0.4361234	2.8592275
H	4.5816251	-0.2120448	2.6311800
C	0.8566595	-0.9740714	3.4263902
H	-0.1868086	-1.2010925	3.6310323
C	3.3745678	-2.7689434	-4.2518637
H	3.4384638	-2.7359720	-5.3353010
C	1.3229599	-1.1382420	-5.6820077
H	2.1162594	-1.5437369	-6.3062709
C	-4.0876194	2.6669743	-0.3724462
H	-5.0033509	2.0780653	-0.2696485
C	1.7284608	-1.9978994	3.0483970
H	1.3427254	-3.0174861	2.9755770
C	-3.6781667	3.4647384	0.6959789
H	-4.2638142	3.4864933	1.6130391
C	-1.7056056	0.4246734	-3.1083678
H	-2.1401270	-0.4489789	-2.5929540
H	-1.1806352	0.9939117	-2.3261668
C	4.2265865	-3.5296709	-2.1317822
H	4.9503359	-4.0865987	-1.5421459
C	-0.7053805	0.1278837	-5.4473346
H	-1.5156589	0.7058136	-5.8874651
C	-2.2216899	3.4135701	-1.6791425
H	-1.6201613	3.4035139	-2.5866773
C	-2.5058709	4.2288240	0.5863148
C	-3.3683270	2.6124952	-1.5758999
C	-1.7875901	4.2039429	-0.6157105
H	-0.8746425	4.7938432	-0.6949272
C	0.2956593	-0.3984823	-6.2595636
H	0.2823506	-0.2283537	-7.3361929
C	4.3172729	-3.4838529	-3.5185487
H	5.1247870	-4.0040635	-4.0341046
C	3.9923035	-2.8811804	2.3408510
H	3.5396467	-3.8471131	2.6166359
H	4.9488668	-2.8186057	2.8816263
C	-3.8662910	1.7391619	-2.7112008
H	-4.6581764	2.2815713	-3.2550881
H	-4.3608123	0.8506277	-2.2837140
C	-1.5465095	3.1221673	3.0664420
H	-2.1603266	2.4108241	2.4919703
H	-0.5548039	3.1499124	2.5911563
C	-2.8136951	1.2778211	-3.7276731
H	-3.3331119	0.7073397	-4.5142581
H	-2.3718354	2.1559424	-4.2266681
C	-0.7402045	1.1931283	4.4105661
H	-0.6875718	0.6980858	5.3948669
H	-1.3112777	0.5414564	3.7270989
C	-2.1514058	4.5132608	2.9466789
H	-3.2090344	4.5389500	3.2628153
H	-1.6053554	5.2415476	3.5608573
C	-1.4021282	2.5615484	4.4796433
H	-2.3868038	2.4452545	4.9565782
H	-0.8038616	3.2301314	5.1178343
H	6.1200448	-1.2815785	-9.1022981

4c**H(+)**

O	7.7298126	-0.3125912	-6.2113187
O	3.7233430	-0.7955045	-0.4127705
O	4.5183066	2.2821618	-8.2100605
O	0.5364663	1.8439631	-2.3792076
N	6.1601371	1.0297621	-7.1929940
N	-1.5834204	-1.8578247	-0.5555858
N	2.1537335	0.5700107	-1.3818462
N	-0.6195083	-2.5668252	-1.1665133
N	0.4706134	-1.8308238	-1.1238478
C	4.1191438	1.6443230	-5.9556388
C	4.5887205	0.9111159	-4.8391990
C	3.7870059	0.8299314	-3.6652197
C	5.8409221	0.2519275	-4.8744433
C	4.2567207	0.0868559	-2.5553771
C	6.2999280	-0.4345994	-3.7565223
H	7.2768716	-0.9157531	-3.8053873
C	6.6652523	0.2862629	-6.1131364
C	2.5281767	1.4780907	-3.6368490
C	4.9200681	1.7001947	-7.2103865
C	5.5082985	-0.5172642	-2.5987955
H	5.8504434	-1.0709403	-1.7248907
C	0.2136039	-0.6527945	-0.4849115
C	3.3958892	-0.0881449	-1.3618095
C	2.8980326	2.3029610	-5.8890379
H	2.5644638	2.8700422	-6.7578279
C	2.1031687	2.2188440	-4.7323294
H	1.1347824	2.7155832	-4.6829545
C	1.6521707	1.3401541	-2.4501948
C	-1.1174726	-0.6576496	-0.1206013
H	-1.7442869	0.0816178	0.3657540
C	6.9959283	1.0636293	-8.4023727
H	7.9986167	1.4184209	-8.1384630
H	6.5216467	1.7331530	-9.1232820
C	-2.9302998	-2.3946064	-0.3804122
H	-2.9528577	-3.3586169	-0.9014444
H	-3.6233911	-1.7036413	-0.8812002
C	1.2604054	0.3811120	-0.2253399
H	0.7816960	1.3400041	0.0035772
H	1.8894093	0.0694548	0.6180045
O	-0.3268371	-0.8163708	3.9874220
N	2.4754231	-2.7056752	-2.8630960
H	1.6098200	-2.3260213	-2.3459521
N	0.5871717	-1.2360129	-4.0538378
O	-3.0620908	2.8238367	2.0435487
C	1.6681394	-1.4906053	-4.8062795
C	2.8781836	-3.9019956	-0.7700184
H	2.3527675	-4.8694291	-0.8791129
H	2.1211392	-3.1942125	-0.4103795
C	1.7489188	-1.2722728	3.0084381
H	1.9956768	-0.2275570	3.2024110
C	2.7038018	-2.3208856	-4.1508535
C	-0.4218441	-0.5027563	-4.5515961
C	2.3690414	-3.4891984	2.2089762
C	0.4827203	-1.7430291	3.4023763
C	3.3176165	-3.4859135	-2.1404135
C	3.9866861	-4.0311276	0.2790005
H	4.7342947	-4.7829874	-0.0178546
H	4.5071047	-3.0652847	0.3569686
C	2.6707539	-2.1328588	2.4256684
H	3.6473615	-1.7414685	2.1374329
C	0.1546587	-3.0865473	3.1770999
H	-0.8101847	-3.4851799	3.4852738
C	3.8844975	-2.7358856	-4.7640557
H	4.0985225	-2.4447806	-5.7885578
C	1.8081717	-1.0116344	-6.1165216
H	2.6933235	-1.2137054	-6.7163191
C	-4.4563059	0.7579442	-0.6948342
H	-5.2522505	0.0291529	-0.8704720
C	1.0965006	-3.9347740	2.5805446
H	0.8320343	-4.9836233	2.4268578
C	-4.2840037	1.2605782	0.5973570
H	-4.9156967	0.9001744	1.4093023
C	-1.5990622	-0.3128203	-3.6302532
H	-1.9841965	-1.3184453	-3.3876613
H	-1.2060026	0.0852161	-2.6833189
C	4.5136468	-3.8907780	-2.7393149
H	5.2088705	-4.5059329	-2.1731342
C	-0.3506661	0.0238076	-5.8537177
H	-1.1644805	0.6285273	-6.2500828

C	-2.6769663	2.1376908	-1.5135558
H	-2.0140097	2.4791039	-2.3083156
C	-3.2976895	2.2326350	0.8346185
C	-3.6620667	1.1725122	-1.7745212
C	-2.4954797	2.6605364	-0.2350416
H	-1.7236656	3.4050812	-0.0394029
C	0.7737135	-0.2364220	-6.6356615
H	0.8494258	0.1681237	-7.6451709
C	4.7939908	-3.5128512	-4.0495167
H	5.7264924	-3.8238580	-4.5212405
C	3.4055720	-4.4398396	1.6464838
H	2.9630359	-5.4454779	1.5631834
H	4.2408877	-4.5325350	2.3596806
C	-3.9282484	0.6236846	-3.1638713
H	-4.7274636	1.2238957	-3.6312628
H	-4.3447670	-0.3937598	-3.0685662
C	-2.5403659	0.7757441	3.2701398
H	-2.9969903	0.0596670	2.5719306
H	-1.5306819	1.0021278	2.8966631
C	-2.7352516	0.5765444	-4.1315475
H	-3.1073380	0.2086535	-5.1010656
H	-2.3567643	1.5955307	-4.3141271
C	-1.6267608	-1.1913759	4.4891831
H	-1.5073509	-1.7224602	5.4471470
H	-2.1423274	-1.8618164	3.7824103
C	-3.3417568	2.0668741	3.2458289
H	-4.4232188	1.8803291	3.3571461
H	-3.0356749	2.7424183	4.0553192
C	-2.4278160	0.0942793	4.6338051
H	-3.4212597	-0.1590905	5.0321031
H	-1.9369458	0.7587212	5.3625241
H	7.0872910	0.0536549	-8.8210125
C	-3.2536025	-2.4966065	1.1112859
H	-2.8006085	-1.6278096	1.6068796
H	-2.7774720	-3.3851543	1.5485554
C	-4.7366151	-2.4341811	1.4570988
H	-5.2674750	-1.7521880	0.7618294
H	-5.2302607	-3.4230928	1.3919755
O	-4.7925672	-1.9181109	2.7819092
C	-6.1282869	-1.7353652	3.2833350
H	-6.7153996	-1.1207203	2.5737611
H	-6.6286895	-2.7184612	3.3750796
C	-6.0078745	-1.0496993	4.6147513
C	-6.3073712	0.3125211	4.7463947
C	-5.5147738	-1.7594280	5.7091642
C	-6.1189403	0.9761165	5.9630348
H	-6.6986988	0.8454495	3.8789870
C	-5.2994137	-1.1316146	6.9461522
H	-5.2787213	-2.8174972	5.5802429
C	-5.6085413	0.2296582	7.0410221
H	-5.4570724	0.7374024	7.9921021
C	-4.7273146	-1.9379304	8.1211669
C	-6.4574615	2.4601905	6.1675466
C	-6.9740966	3.1283282	4.8821150
H	-7.2051962	4.1836556	5.0832428
H	-6.2255482	3.1062931	4.0761610
H	-7.8950598	2.6530126	4.5152364
C	-7.5548821	2.5799992	7.2499896
H	-7.2248588	2.1632370	8.2112572
H	-7.8150141	3.6362988	7.4127996
H	-8.4648987	2.0444604	6.9440937
C	-5.1943560	3.2177474	6.6344185
H	-5.4224457	4.2835379	6.7825725
H	-4.8077094	2.8230864	7.5832753
H	-4.3931401	3.1409975	5.8858591
C	-4.6356227	-1.1117975	9.4148096
H	-3.9665898	-0.2460060	9.3036013
H	-5.6220587	-0.7494108	9.7378073
H	-4.2311651	-1.7375975	10.2227358
C	-5.6266167	-3.1641432	8.3929120
H	-6.6497920	-2.8501116	8.6434407
H	-5.6829735	-3.8341573	7.5239723
H	-5.2297757	-3.7467282	9.2374070
C	-3.3049998	-2.4139466	7.7484256
H	-2.8738308	-3.0118278	8.5650724
H	-3.3102865	-3.0339829	6.8411511
H	-2.6447984	-1.5529382	7.5685826

5H(+)

O	1.5896094	-2.7428967	1.0872073
O	2.1502817	1.7889363	1.2616065
N	-0.5821797	-0.7013090	4.7417653
N	2.0225608	-0.4974214	1.1085559
N	-0.6439712	-1.9031505	4.1740567
N	0.4024159	-1.9249488	3.3691137
O	1.0508663	2.1904958	-5.7270543
N	0.9741727	-0.1065382	-5.8649696
C	-1.5860284	-0.2857881	5.6826284
O	0.6600352	-2.3750576	-5.9241168
C	1.1436624	-0.7818661	3.3915312
C	0.4913172	0.0269515	4.2961067
H	0.6828623	1.0443273	4.6110551
C	1.5459643	-0.3278144	-1.6428153
C	-2.1773396	1.0180899	7.6190569
C	1.5038007	-1.5966236	-1.0098558
C	1.7017821	-1.6888473	0.4423094
C	-1.2086244	0.5810102	6.7138897
H	-0.1671900	0.8768867	6.8245428
C	-2.8865640	-0.7498257	5.5240219
H	-3.1235014	-1.4165902	4.6968865
C	-3.4940537	0.5469368	7.4484815
H	-4.2517488	0.8874989	8.1509807
C	1.7718272	0.8674773	-0.9152804
C	1.3338028	-0.2517142	-3.0492037
C	1.9937054	0.8130661	0.5399106
C	2.3556461	-0.5936622	2.5369113
H	2.8565659	0.3388124	2.8164408
H	3.0398041	-1.4411704	2.6755520
C	-1.8541386	1.9772546	8.7720971
C	-3.8753335	-0.3341498	6.4298456
C	1.2609970	-2.7473779	-1.7571227
H	1.2331789	-3.7098122	-1.2463316
C	1.3473009	1.0074801	-3.6942438
C	-5.3108158	-0.8509850	6.2650601
C	1.0983922	-1.4356756	-3.7872662
C	1.0634776	-2.6672473	-3.1445533
H	0.8808860	-3.5619819	-3.7394862
C	1.5718982	2.1651241	-2.9589790
H	1.5784501	3.1216046	-3.4812290
C	1.7845422	2.0953298	-1.5728949
H	1.9639948	2.9996482	-0.9911924
C	-0.3603792	2.3377964	8.8288474
H	0.2705495	1.4507391	8.9887020
H	-0.1793778	3.0218225	9.6685566
H	-0.0260055	2.8518190	7.9149324
C	-2.6663791	3.2781018	8.5732717
H	-2.4076088	3.7636915	7.6212078
H	-2.4483577	3.9831451	9.3876330
H	-3.7482597	3.0890609	8.5749631
C	-5.8409477	-0.4435752	4.8714934
H	-5.8454576	0.6493914	4.7553940
H	-6.8707584	-0.8055976	4.7427285
H	-5.2370930	-0.8712288	4.0588485
C	-2.2494056	1.3184980	10.1132228
H	-3.3220850	1.0872482	10.1583793
H	-2.0225865	1.9998282	10.9451471
H	-1.6927612	0.3842689	10.2731002
C	1.1149221	1.1057880	-5.1648949
C	1.3811176	-0.5521128	-12.2956342
H	1.1780270	-0.5310384	-13.3669130
C	0.8830258	-1.5919193	-11.5055667
H	0.2903891	-2.3886242	-11.9573002
C	-5.3017289	-2.3924218	6.3861906
H	-4.6819012	-2.8630178	5.6102073
H	-6.3235778	-2.7827480	6.2771143
H	-4.9176895	-2.7083445	7.3663226
C	0.8909485	-1.3700604	-5.2627960
C	2.1490091	0.4539974	-11.7056352
H	2.5492492	1.2645079	-12.3162905
C	1.9190411	-0.6092756	-9.5332337
C	0.8794038	-0.0665967	-7.3396547
H	-0.0018624	-0.6458748	-7.6405588
H	0.7285964	0.9791523	-7.6230905
C	2.4164767	0.4259779	-10.3341221
H	3.0339734	1.2076214	-9.8912978
C	1.1509636	-1.6175458	-10.1362818
H	0.7655110	-2.4355833	-9.5226275

C	-6.2606262	-0.2830908	7.3324720
H	-5.9539187	-0.5682227	8.3488201
H	-7.2707430	-0.6829733	7.1708277
H	-6.3302190	0.8132107	7.2803575
C	5.9001187	1.0019894	-6.6604463
H	6.8547452	1.4112015	-6.3274511
C	4.8234705	1.8521846	-6.9245875
H	4.9326942	2.9301874	-6.7968158
C	3.6025179	1.3299235	-7.3584405
H	2.7720199	2.0100116	-7.5466995
C	3.4344264	-0.0523535	-7.5356466
C	4.5223971	-0.8943260	-7.2617564
H	4.4084694	-1.9720799	-7.3980445
C	5.7453540	-0.3762972	-6.8304676
H	6.5791710	-1.0506435	-6.6300187
C	2.1351445	-0.6655567	-8.0230483
H	2.1588295	-1.7304070	-7.7497216
H	0.5968554	-2.7041255	2.7172834

13H(+)

N	1.9616173	-1.4899523	-6.0896119
N	3.6424642	-2.4174340	-5.1293281
N	3.2469818	-1.8726744	-6.2560487
C	2.6053850	-2.4017993	-4.2476315
C	1.0350471	-1.8192485	-8.3403055
C	1.5107943	-1.8044626	-4.8488539
H	0.5065973	-1.5614212	-4.5018135
C	-0.1622707	-2.5107796	-8.4991565
H	-0.9753538	-2.3229188	-7.7936048
C	2.0976867	-2.0444738	-9.2282049
H	3.0257367	-1.4975281	-9.0660426
C	2.7434717	-2.9638561	-2.8714650
C	1.2286049	-0.8598813	-7.1899417
H	1.8048722	0.0265143	-7.4948427
H	0.2696077	-0.5267284	-6.7738049
C	-0.3356571	-3.4318316	-9.5487520
C	1.9746097	-2.9639636	-10.2700245
C	3.1074519	-3.2366067	-11.2703553
C	0.7476493	-3.6424132	-10.4061078
H	0.6395590	-4.3504253	-11.2261683
C	-1.6942333	-4.1253284	-9.7431968
C	3.4364568	-4.7458650	-11.2761916
H	3.7250982	-5.0879583	-10.2724694
H	4.2697093	-4.9521794	-11.9638984
H	2.5788136	-5.3504580	-11.6006967
C	-2.7731005	-3.0427319	-9.9777441
H	-2.8651846	-2.3670840	-9.1158519
H	-3.7542566	-3.5121762	-10.1434240
H	-2.5282787	-2.4335626	-10.8592985
C	4.3898920	-2.4605914	-10.9240990
H	4.2304411	-1.3727099	-10.9526143
H	5.1745110	-2.6982235	-11.6562507
H	4.7719395	-2.7254850	-9.9281629
C	2.6460600	-2.8067681	-12.6817425
H	1.7542810	-3.3623327	-13.0037051
H	3.4423239	-2.9933465	-13.4176213
H	2.4016938	-1.7349368	-12.7014071
C	-2.0609828	-4.9413811	-8.4846945
H	-1.3516310	-5.7668133	-8.3329467
H	-3.0669787	-5.3743099	-8.5905626
H	-2.0577029	-4.3196782	-7.5781345
C	-1.6987231	-5.0818637	-10.9469868
H	-1.4967629	-4.5531286	-11.8895827
H	-2.6863865	-5.5551890	-11.0387394
H	-0.9564508	-5.8851173	-10.8324349
N	-1.5833665	-2.1286108	-3.4393876
N	-0.9399587	0.4977337	-3.7264363
C	-2.3578018	-1.2304898	-2.7977430
C	-1.9278977	0.1978659	-2.8624568
C	-2.5348356	1.1831189	-2.0624247
H	-3.3111249	0.9273642	-1.3441501
C	-1.9365025	-3.4266122	-3.4551881
O	4.9387056	-5.0558808	-6.4412595
C	-0.5254486	1.7711497	-3.8444740
C	-2.1036046	2.4996944	-2.1751874
H	-2.5438611	3.2782926	-1.5513153
C	-3.5251782	-1.6149786	-2.1187346
H	-4.1649356	-0.8827311	-1.6301153
C	-1.0851271	2.8050906	-3.0751687
H	-0.7319808	3.8295964	-3.1790547

C	-1.0957942	-4.3236803	-4.3267692
H	-0.0735158	-3.9220238	-4.3557222
H	-1.4734299	-4.2048001	-5.3604996
C	3.6412758	-5.3590307	-6.1599300
C	-3.8676411	-2.9631891	-2.0873471
H	-4.7618390	-3.2913660	-1.5559610
C	1.0313615	-6.1627401	-5.4256997
C	-3.0672594	-3.8856725	-2.7570086
H	-3.3253570	-4.9432844	-2.7588544
C	1.2694822	-5.4459004	-6.6037481
H	0.4369109	-5.1895729	-7.2592333
C	2.5518211	-5.0485698	-6.9850238
H	2.6766168	-4.4933729	-7.9124435
C	-0.3568926	-6.6381378	-5.0691398
H	-0.3160481	-7.6894186	-4.7424264
H	-0.9828954	-6.6177005	-5.9759979
C	2.1324325	-6.4301640	-4.5938240
H	1.9796371	-6.9732520	-3.6576485
C	-1.0561561	-5.8097270	-3.9737937
H	-2.0735613	-6.2140741	-3.8474280
H	-0.5350130	-5.9313794	-3.0124060
C	3.4173260	-6.0343325	-4.9472346
H	4.2746556	-6.2546299	-4.3104057
C	0.5557786	2.0072791	-4.8695949
H	0.1039822	1.8726917	-5.8689025
H	1.2901497	1.1914923	-4.7734046
O	6.2620503	-0.6087468	-5.9822959
C	6.6725803	-3.9423438	-7.6463629
H	6.9591105	-3.6137558	-8.6600050
H	7.3017200	-4.8135381	-7.4103643
C	3.8255390	1.9071280	-7.1621754
H	3.4100606	2.2987618	-8.0941337
C	3.3760711	2.4248762	-5.9422575
C	3.9375511	1.8967535	-4.7669282
H	3.5993216	2.2514435	-3.7913234
C	4.8981550	0.8942192	-4.8064835
H	5.2888569	0.4510135	-3.8919300
C	5.3379447	0.3881702	-6.0392821
C	4.7991414	0.9062968	-7.2253503
H	5.1235682	0.5286349	-8.1938938
C	1.2637709	3.3608322	-4.8117099
H	1.7198845	3.4912576	-3.8161229
H	0.5370471	4.1812034	-4.9243437
C	6.3635443	-1.4888259	-7.1154910
H	7.0193916	-1.0417831	-7.8855019
H	5.3575524	-1.6227049	-7.5448903
C	6.9092309	-2.8222230	-6.6231060
H	6.3858614	-3.0725963	-5.6889375
H	7.9800423	-2.7269834	-6.3915930
C	2.3315939	3.5192036	-5.9062551
H	2.8215839	4.4995234	-5.7752618
H	1.8309657	3.5624600	-6.8872778
C	5.2192348	-4.3927783	-7.6842446
H	5.0388907	-5.0936493	-8.5181667
H	4.5400920	-3.5319247	-7.8018520
O	2.9653353	-0.3646794	-2.1285127
O	0.6866430	-4.3139567	-1.6012739
O	-1.9040755	2.9585593	1.7613364
N	1.7853796	-2.3230781	-1.9498525
O	-3.8045449	-1.0859350	2.7894879
N	-2.8571723	0.9377144	2.2520867
N	-2.2088053	3.0279577	6.2080484
C	-0.0956340	-1.0133992	-0.2933681
C	1.9728467	-0.9483557	-1.7097870
N	-1.7988395	1.4597833	4.8228384
C	0.9340868	-0.2631141	-0.9062143
C	-0.2103245	-2.4058416	-0.5135710
N	-1.3446305	2.0429289	5.9149661
C	-0.9132418	1.0396156	0.7573300
C	0.7608996	-3.1064966	-1.3952930
C	0.0897848	1.7608761	0.1185510
H	0.1352147	2.8379408	0.2719236
C	-2.0511858	-1.1118426	1.1784093
C	-2.1576442	-2.4769283	0.9284310
H	-2.9640455	-3.0318013	1.4065794
C	-1.0277521	-0.3562871	0.5570398
C	-2.9496021	2.0700884	4.4156040
C	1.0133953	1.1100369	-0.7090505
H	1.8136898	1.6598377	-1.2033895
C	-1.8940259	1.7414769	1.6028464

C	-0.9359654	3.4391199	8.2953862
C	-3.2192663	3.0890929	5.3048734
H	-4.0072643	3.8321603	5.3480887
C	-1.2417876	-3.1222135	0.0832922
H	-1.3236709	-4.1877325	-0.1281232
C	0.3727255	3.9040563	8.2014061
H	0.6154501	4.6287373	7.4212231
C	-1.2832030	2.5041472	9.2786558
H	-2.3142645	2.1529917	9.3172425
C	-3.7249953	1.6259600	3.2162551
H	-4.5109978	0.9172851	3.5038772
H	-4.1828179	2.4936509	2.7261567
C	-1.9869083	3.9381972	7.3443224
H	-2.9579906	4.0348206	7.8513559
H	-1.7198120	4.9172707	6.9253838
C	-2.9793725	-0.4582306	2.1256524
C	1.3628406	3.4516459	9.0921654
C	-0.3331614	2.0298907	10.1827365
C	-0.6743160	1.0293615	11.2967546
C	0.9810289	2.5231213	10.0661113
H	1.7283151	2.1683112	10.7737714
C	2.7961020	3.9950913	8.9826658
C	0.2439568	-0.2080090	11.1815533
H	0.1166782	-0.6986294	10.2054916
H	0.0002527	-0.9360385	11.9690390
H	1.3041494	0.0575124	11.2894049
C	2.7727517	5.5252461	9.1980303
H	2.1432655	6.0292552	8.4509006
H	3.7890986	5.9384047	9.1155684
H	2.3800458	5.7741225	10.1939586
C	-2.1337884	0.5493847	11.2212557
H	-2.8480579	1.3781171	11.3310741
H	-2.3323072	-0.1636013	12.0330927
H	-2.3390075	0.0355888	10.2700402
C	-0.4540657	1.7104423	12.6668572
H	0.5886639	2.0322428	12.7951240
H	-0.6948531	1.0150125	13.4844175
H	-1.0953706	2.5974258	12.7678514
C	3.3552898	3.6924561	7.5753450
H	3.3456330	2.6126865	7.3692598
H	4.3913969	4.0518674	7.4897593
H	2.7675803	4.1945335	6.7948442
C	3.7438090	3.3714896	10.0210380
H	3.4232639	3.5891007	11.0496933
H	4.7534738	3.7872347	9.8959546
H	3.8180869	2.2803938	9.9032650
N	-0.5801669	-1.0121315	4.1962553
N	1.0696176	0.9992167	3.6507557
C	0.4712160	-1.2807548	3.3725238
C	1.3411958	-0.1448793	3.0040400
C	2.3705297	-0.2619864	2.0603560
H	2.5614370	-1.1895332	1.5242453
C	-1.4280928	-1.9565015	4.6789065
O	-6.3934452	3.4930956	6.6186201
C	1.8023380	2.0938451	3.4010025
C	3.1344816	0.8715561	1.7915636
H	3.9305425	0.8293563	1.0472076
C	0.6697742	-2.5961432	2.9575187
H	1.5000279	-2.8411611	2.3011850
C	2.8525843	2.0587513	2.4635086
H	3.4407305	2.9524361	2.2604535
C	-2.4442314	-1.5158762	5.6885179
H	-2.7620153	-0.4943145	5.4481834
H	-1.9131931	-1.4236685	6.6547528
C	-5.9076581	2.2226194	6.7761023
C	-0.2090013	-3.5878410	3.3849296
H	-0.0713950	-4.6149212	3.0456859
C	-5.0035872	-0.4558602	6.8979634
C	-1.2535083	-3.2735542	4.2519253
H	-1.9353485	-4.0421565	4.6050211
C	-4.6439135	0.4783888	7.8737700
H	-4.0153659	0.1648271	8.7095611
C	-5.0887491	1.8043663	7.8324327
H	-4.8004730	2.4886581	8.6286630
C	-4.5714026	-1.9024863	7.0036262
H	-5.4626475	-2.5479669	7.0532909
H	-4.0370251	-2.0440159	7.9566515
C	-5.8147284	-0.0103816	5.8384726
H	-6.1107677	-0.7107476	5.0552807
C	-3.6773276	-2.4020489	5.8513947

H	-3.3961583	-3.4435123	6.0719062
H	-4.2327680	-2.4066530	4.9024611
C	-6.2612153	1.3047989	5.7733359
H	-6.9021965	1.6444259	4.9591514
C	1.4304732	3.3202498	4.1919323
H	1.7651816	3.1616708	5.2311965
H	0.3341317	3.3370007	4.2603562
O	-4.2102697	6.3064140	4.7585080
C	-6.8156560	5.7616394	7.2563152
H	-6.6588258	6.4853441	8.0726516
H	-7.9004132	5.5939797	7.1894041
C	-0.6161840	6.8279286	5.5020001
H	0.0241757	7.3072202	6.2463635
C	-0.0345820	6.0591964	4.4870403
C	-0.8951323	5.4865769	3.5355073
H	-0.5035492	4.8874219	2.7140219
C	-2.2738964	5.6169393	3.6319107
H	-2.9235897	5.1146509	2.9168954
C	-2.8415740	6.3413694	4.6883186
C	-2.0037089	6.9857968	5.6099190
H	-2.4134067	7.5863112	6.4210853
C	1.9707458	4.6494702	3.6581734
H	1.7061503	4.7540577	2.5923102
H	3.0715615	4.6543718	3.6981329
C	-4.8422895	6.7745621	5.9626476
H	-4.7488855	7.8713951	6.0363008
H	-4.3144241	6.3403911	6.8323221
C	-6.3067667	6.3270322	5.9218095
H	-6.3983950	5.5484063	5.1517404
H	-6.9494085	7.1595676	5.6039523
C	1.4693513	5.8746385	4.4370471
H	1.9312226	6.7758521	3.9993967
H	1.8510063	5.8238046	5.4707670
C	-6.1483329	4.4530713	7.6581896
H	-6.5553316	4.0845187	8.6151951
H	-5.0576255	4.5874511	7.7881855
H	3.7647115	-2.7901527	-2.5085346
H	2.5332816	-4.0405160	-2.8564887
H	-0.8030749	0.0121792	4.4225711

13H$_2$(2+)

N	2.6417189	-2.2561788	-6.6274429
N	1.8907446	-1.0975256	-5.0041224
N	1.6352202	-1.4564301	-6.2446422
C	3.0598501	-1.6607858	-4.5893688
C	1.4819684	-2.4119880	-8.7834275
C	3.5512530	-2.4185219	-5.6334770
H	4.4372517	-3.0319754	-5.7376185
C	1.4527368	-1.1791626	-9.4265458
H	2.3430881	-0.5487722	-9.3857240
C	0.3505572	-3.2362617	-8.7835112
H	0.4034836	-4.1883457	-8.2552198
C	3.6488661	-1.3822093	-3.2455577
C	2.6990195	-2.8055292	-7.9978965
H	2.7972863	-3.8945463	-7.8999518
H	3.6159514	-2.4082041	-8.4566350
C	0.2929365	-0.7281403	-10.0733790
C	-0.8195971	-2.8344103	-9.4329765
C	-2.0831001	-3.7052267	-9.4780053
C	-0.8184994	-1.5773729	-10.0687413
H	-1.7265024	-1.2564547	-10.5761558
C	0.2791038	0.6717902	-10.7040589
C	-3.2833123	-2.9084406	-8.9189344
H	-3.0919005	-2.5913970	-7.8830191
H	-4.1901016	-3.5309193	-8.9286298
H	-3.4936864	-2.0098137	-9.5139804
C	1.4171487	0.7883421	-11.7416219
H	2.4052572	0.6272854	-11.2878968
H	1.4180808	1.7897758	-12.1959357
H	1.2901661	0.0484226	-12.5441622
C	-1.9350469	-4.9859563	-8.6397421
H	-1.1148831	-5.6239789	-9.0004898
H	-2.8605543	-5.5749795	-8.6960117
H	-1.7530506	-4.7520608	-7.5796699
C	-2.3660114	-4.1062900	-10.9431184
H	-2.5174394	-3.2245169	-11.5804465
H	-3.2730388	-4.7253369	-11.0027941
H	-1.5273065	-4.6823423	-11.3591341
C	0.4994994	1.7130756	-9.5801232
H	-0.3036477	1.6474459	-8.8304060

H	0.5037599	2.7319763	-9.9946992
H	1.4577663	1.5524639	-9.0641759
C	-1.0513735	0.9901723	-11.4055251
H	-1.2568518	0.2854514	-12.2236714
H	-1.0086290	1.9984638	-11.8405197
H	-1.9002978	0.9669013	-10.7068362
N	-0.8433252	-1.7499392	-3.8616605
N	-0.0273890	0.7557341	-4.1993026
C	-1.4884630	-0.8396330	-3.1183584
C	-1.1150867	0.5619414	-3.4046947
C	-1.8071251	1.6786269	-2.9392818
H	-2.6920899	1.5523117	-2.3210366
C	-1.1065898	-3.0562834	-3.7011115
O	5.6064125	-5.5378535	-6.4056065
C	0.4184910	1.9746189	-4.5948506
C	-1.3610427	2.9523977	-3.2871631
H	-1.8938256	3.8312349	-2.9225488
C	-2.4421951	-1.1831669	-2.1503366
H	-2.9447396	-0.4313384	-1.5444442
C	-0.2495966	3.1043563	-4.1132008
H	0.0969746	4.0923158	-4.4066117
C	-0.3623486	-3.9878913	-4.6217521
H	0.7082864	-3.7420439	-4.5401832
H	-0.6313481	-3.7124209	-5.6568380
C	4.2759229	-5.8106315	-6.2466791
C	-2.7102601	-2.5370604	-1.9644461
H	-3.4357275	-2.8535370	-1.2145055
C	1.5214268	-6.1715938	-5.7153433
C	-2.0401923	-3.4835102	-2.7383797
H	-2.2458017	-4.5434009	-2.6002993
C	2.0451834	-6.3587323	-6.9989229
H	1.3749308	-6.6428658	-7.8135353
C	3.4066215	-6.1917864	-7.2777288
H	3.7709338	-6.3555601	-8.2904637
C	0.0439169	-6.3779467	-5.4615989
H	-0.1327944	-7.4301848	-5.1806478
H	-0.4970248	-6.2310982	-6.4094294
C	2.4196326	-5.8301186	-4.6895916
H	2.0606401	-5.6760557	-3.6711314
C	-0.5892395	-5.4816252	-4.3863640
H	-1.6684120	-5.7027272	-4.3733684
H	-0.2084273	-5.7565520	-3.3887296
C	3.7735318	-5.6518416	-4.9456592
H	4.4639782	-5.3690023	-4.1504091
C	1.5570627	2.0021135	-5.5640804
H	1.1351300	1.7833728	-6.5634730
H	2.2181042	1.1532010	-5.3484893
O	6.7312078	-1.4778351	-6.2673558
C	7.5517978	-4.8882962	-7.6379668
H	7.9166992	-4.7124078	-8.6626310
H	8.2117843	-5.6491195	-7.1970778
C	4.4175022	1.0475302	-7.6783405
H	3.8610222	1.2562648	-8.5949636
C	4.3346813	1.9448746	-6.6104896
C	5.0810019	1.6525268	-5.4546789
H	5.0402375	2.3269523	-4.5976675
C	5.8746804	0.5156813	-5.3756530
H	6.4702284	0.3010178	-4.4873049
C	5.9437037	-0.3773987	-6.4582377
C	5.2141202	-0.1015614	-7.6215107
H	5.2748406	-0.7531155	-8.4918009
C	2.3894954	3.2820620	-5.6084129
H	2.8495164	3.4375144	-4.6198690
H	1.7674758	4.1652524	-5.8202039
C	6.8128235	-2.4543879	-7.3183187
H	7.2611075	-2.0061348	-8.2224016
H	5.7924880	-2.7889904	-7.5821287
C	7.6469251	-3.6112687	-6.7895141
H	7.3039679	-3.8358961	-5.7698288
H	8.6966587	-3.2952002	-6.7115815
C	3.4779796	3.1890373	-6.6975736
H	4.1125690	4.0871729	-6.6328732
H	2.9951401	3.2224560	-7.6866671
C	6.1394319	-5.4572407	-7.7392784
H	6.1534736	-6.4566536	-8.2059406
H	5.4868012	-4.8091714	-8.3526270
O	2.9518286	1.2133347	-2.7251789
O	2.0617976	-3.2278036	-2.0646871
O	-2.2446001	2.9155311	1.7634778
N	2.6075938	-1.0123447	-2.2726550

O	-2.7917144	-1.5151240	2.7979764
N	-2.5710927	0.6959639	2.2201105
N	-2.5368271	2.3113753	6.4162305
C	0.5326117	-0.3281554	-0.4872763
C	2.3381993	0.3568451	-2.0970606
N	-2.3242630	0.5381680	5.2499822
C	1.2665354	0.6993906	-1.1269020
C	0.8047112	-1.6933968	-0.7449006
N	-1.9994269	1.0819235	6.4014745
C	-0.7985254	1.3817177	0.6572358
C	1.8474121	-2.0687186	-1.7318885
C	-0.0607082	2.3734714	0.0219995
H	-0.3075968	3.4161342	0.2212596
C	-1.1939080	-1.0082065	1.1124062
C	-0.8899292	-2.3403938	0.8641803
H	-1.4375798	-3.1102582	1.4079089
C	-0.4996542	0.0179095	0.4293813
C	-3.0732892	1.4135536	4.5202854
C	0.9782337	2.0329050	-0.8596308
H	1.5635844	2.8027059	-1.3617023
C	-1.9061892	1.7530303	1.5719755
C	-1.2832322	2.7551699	8.4896128
C	-3.2127188	2.5667552	5.2686907
H	-3.7181655	3.5062207	5.0818547
C	0.0957046	-2.6830167	-0.0767705
H	0.3207335	-3.7257140	-0.2997380
C	-0.0008893	3.2338907	8.2327698
H	0.1413636	3.9323443	7.4061454
C	-1.5004920	1.8437548	9.5293498
H	-2.5134304	1.4747475	9.6908423
C	-3.6317526	1.0655963	3.1826935
H	-4.3035795	0.2031748	3.2670045
H	-4.1774056	1.9199171	2.7702203
C	-2.4317895	3.1683891	7.6119484
H	-3.3899616	3.0858903	8.1461699
H	-2.3184901	4.1960718	7.2422759
C	-2.2365000	-0.6638595	2.1095996
C	1.0901177	2.8266002	9.0187908
C	-0.4413739	1.3977283	10.3222189
C	-0.6254295	0.3777036	11.4550730
C	0.8400023	1.9139468	10.0488949
H	1.6695319	1.5859625	10.6728977
C	2.4940756	3.3728312	8.7150155
C	0.2780586	-0.8472368	11.1863503
H	0.0197913	-1.3188440	10.2263107
H	0.1517797	-1.5945819	11.9834287
H	1.3412200	-0.5735571	11.1525842
C	2.4584420	4.9171141	8.7068592
H	1.7776922	5.3063732	7.9369908
H	3.4600156	5.3215993	8.4996896
H	2.1254381	5.3068390	9.6787617
C	-2.0790220	-0.1144433	11.5645110
H	-2.7741854	0.7052825	11.7964853
H	-2.1597110	-0.8517689	12.3754142
H	-2.4133472	-0.6021915	10.6359051
C	-0.2269235	1.0274954	12.7990806
H	0.8208534	1.3569644	12.7965271
H	-0.3506179	0.3086743	13.6222322
H	-0.8562293	1.9033850	13.0109325
C	2.9357979	2.8640031	7.3237590
H	2.9561792	1.7650875	7.2966961
H	3.9414765	3.2370322	7.0797617
H	2.2499584	3.2073308	6.5374137
C	3.5383613	2.9213563	9.7497987
H	3.2767739	3.2559252	10.7636391
H	4.5173053	3.3542048	9.4998716
H	3.6568131	1.8284639	9.7642043
N	-0.8799096	-1.8110468	5.2430333
N	0.4879823	0.2352607	4.1285334
C	0.2384542	-2.0991488	4.5240549
C	0.8642063	-0.9977939	3.7578727
C	1.8201091	-1.2330308	2.7578399
H	2.0863836	-2.2409390	2.4443935
C	-1.4754022	-2.6885893	6.0832573
O	-6.5137079	3.3449996	6.8417670
C	1.0630785	1.3087707	3.5607202
C	2.4051520	-0.1252436	2.1501696
H	3.1483684	-0.2607382	1.3640613
C	0.7801386	-3.3800403	4.6151459
H	1.6896663	-3.6230782	4.0708660

C	2.0347568	1.1564534	2.5547035
H	2.4935553	2.0273786	2.0909417
C	-2.7168312	-2.2751785	6.8048139
H	-2.6834197	-1.2069279	7.0572534
H	-2.7697240	-2.8389046	7.7476351
C	-6.1504996	2.0306127	6.8900410
C	0.1863167	-4.3225998	5.4554808
H	0.6161020	-5.3207234	5.5445033
C	-5.5303754	-0.7251112	6.7668100
C	-0.9341599	-3.9727855	6.2009311
H	-1.4025156	-4.6808537	6.8827394
C	-5.2756381	0.0223330	7.9202375
H	-4.8586753	-0.4715324	8.8007736
C	-5.5823443	1.3874196	7.9980179
H	-5.4122994	1.9220279	8.9315823
C	-5.2703097	-2.2103225	6.6899150
H	-6.1089282	-2.6906142	6.1621943
H	-5.2560926	-2.6437042	7.7023684
C	-6.0820071	-0.0527513	5.6617033
H	-6.3084004	-0.6160851	4.7529155
C	-3.9695564	-2.5824462	5.9556576
H	-3.9796690	-3.6567467	5.7109198
H	-3.9004076	-2.0370771	5.0023178
C	-6.3870128	1.2996555	5.7132149
H	-6.8432128	1.8128251	4.8659609
C	0.6176870	2.6448603	4.1006754
H	0.6670600	2.5760561	5.2003465
H	-0.4576154	2.7661158	3.8843696
O	-4.3862344	6.3080640	5.1536800
C	-7.0019644	5.5232931	7.6967380
H	-6.8339058	6.1775207	8.5670722
H	-8.0913729	5.4377972	7.5751632
C	-0.7537707	6.0497323	5.8081657
H	-0.0356161	6.2259300	6.6123169
C	-0.3105032	5.5174916	4.5935121
C	-1.2596496	5.3534052	3.5715618
H	-0.9610817	4.9466903	2.6050800
C	-2.6012895	5.6544118	3.7708413
H	-3.3362312	5.4833044	2.9846230
C	-3.0376678	6.1368064	5.0151286
C	-2.0979329	6.3663326	6.0306313
H	-2.3987944	6.7855578	6.9894700
C	1.4018152	3.8604627	3.5975265
H	1.1740520	4.0293151	2.5313900
H	2.4831218	3.6549009	3.6501690
C	-4.9126765	6.3857826	6.4927546
H	-4.6807129	7.3677616	6.9397577
H	-4.4151480	5.6136247	7.1065698
C	-6.4147054	6.1376746	6.4161932
H	-6.5961128	5.4482397	5.5809544
H	-6.9410795	7.0724981	6.1784191
C	1.1428421	5.1459827	4.4033979
H	1.6794009	5.9755295	3.9127162
H	1.6090094	5.0395670	5.3954829
C	-6.4383441	4.1443133	8.0335515
H	-7.0221159	3.6736838	8.8426185
H	-5.3872629	4.2064936	8.3707003
H	4.3471569	-0.5372683	-3.3100560
H	4.1743336	-2.2664475	-2.8672264
H	-1.3578969	-0.8446444	5.1298758
H	0.5692114	-0.0997150	-4.4851307

Catalyst-substrate complexes

1H(+)·9 (anion-π)

N	0.0798236	-3.0408006	-0.4061686
N	1.4552201	-1.1565272	-1.5791130
C	1.1056534	-3.4612821	-1.1951259
C	1.7766826	-2.3995612	-1.9669994
C	2.6858696	-2.6695884	-2.9959479
H	2.9240651	-3.6896669	-3.2916106
C	-0.6569836	-3.8347963	0.4012163
O	-5.8502924	1.9376908	1.7214878
C	2.0993120	-0.1097604	-2.1220590
C	3.2811591	-1.5828835	-3.6286532
H	3.9905844	-1.7399634	-4.4405004
C	1.4560972	-4.8037986	-1.1559342
H	2.3022749	-5.1570560	-1.7386414
C	2.9991660	-0.2928647	-3.1835886
H	3.5035955	0.5607918	-3.6301659
C	-1.8069882	-3.2741582	1.1724181
H	-1.8329604	-2.1791370	1.0967690
H	-1.6151298	-3.5090102	2.2328228
C	-5.4277915	0.6359372	1.7849762
C	0.7622776	-5.6594839	-0.2992243
H	1.0638103	-6.7036652	-0.2163048
C	-4.6754304	-2.0878987	1.7118410
C	-0.2952513	-5.1831193	0.4684623
H	-0.8214066	-5.8253622	1.1703156
C	-4.3568679	-1.2789279	2.8060325
H	-3.8102125	-1.7074279	3.6487753
C	-4.7298129	0.0694563	2.8591258
H	-4.4807958	0.6551892	3.7420568
C	-4.3066406	-3.5518225	1.6730714
H	-5.1866974	-4.1385256	1.3624513
H	-4.0433859	-3.8936088	2.6864915
C	-5.3688578	-1.4958236	0.6411489
H	-5.6363792	-2.1036554	-0.2269118
C	-3.1463138	-3.8927600	0.7202533
H	-3.0454933	-4.9885383	0.6715791
H	-3.3873637	-3.5542120	-0.3009018
C	-5.7394101	-0.1570060	0.6682108
H	-6.2852482	0.2983159	-0.1586669
C	1.8562937	1.2150723	-1.4528977
H	2.2272082	1.0848552	-0.4202407
H	0.7708706	1.3685206	-1.3492427
O	-3.0267974	4.8893834	-0.0210032
C	-5.8696577	4.2342355	2.3748461
H	-5.6001081	4.9059924	3.2065318
H	-6.9535554	4.3405169	2.2184575
C	0.4490917	3.6724852	0.3869574
H	1.0703061	3.1436593	1.1130393
C	1.0042842	4.0582716	-0.8363461
C	0.1729751	4.7358546	-1.7445357
H	0.5786721	5.0560681	-2.7071523
C	-1.1586124	5.0063478	-1.4481366
H	-1.8017306	5.5315132	-2.1549396
C	-1.7030726	4.5968910	-0.2202551
C	-0.8874088	3.9319116	0.7061043
H	-1.2741046	3.6116841	1.6714029
C	2.5123522	2.4368860	-2.0942994
H	2.0455494	2.6449588	-3.0718383
H	3.5776633	2.2310385	-2.2835934
C	-3.6358998	4.3817016	1.1719923
H	-3.1926013	4.8712868	2.0609006
H	-3.4427086	3.2973891	1.2460649
C	-5.1298712	4.6487268	1.0923705
H	-5.5369057	4.0962231	0.2339524
H	-5.2948996	5.7192178	0.8990349
C	2.4219971	3.6912717	-1.2010332
H	2.9087158	4.5323169	-1.7197430
H	3.0021564	3.5092434	-0.2815051
C	-5.5619898	2.8097566	2.8220629
H	-6.1717062	2.5241145	3.6979472
H	-4.5000920	2.7165661	3.1107702
H	-0.0201790	-2.0094564	-0.4040928
C	5.0127020	-1.5543031	-0.4571562
C	5.7553700	-1.7954020	-1.6264797
C	6.3205838	-0.7405617	-2.3428433
C	6.1434877	0.5894454	-1.9403921
C	5.4148864	0.8172845	-0.7611868
C	4.8700269	-0.2254848	-0.0155702

H	5.8782644	-2.8194237	-1.9831473
H	6.8923126	-0.9574249	-3.2485939
H	5.2792835	1.8429524	-0.4076693
H	4.3121110	-0.0183422	0.8932218
S	4.3746837	-2.9703681	0.3848575
C	2.8815421	-2.4076897	1.3366965
C	2.3230781	-3.4506644	2.0744149
H	2.7940564	-4.4306640	2.1035869
C	1.0578610	-3.3054912	2.7530686
O	2.4483194	-1.2302121	1.2155836
O	0.4863376	-2.0527133	2.7140830
H	1.1361135	-1.4685826	2.2087177
O	0.4283177	-4.2250959	3.2925222
C	6.7270208	1.7387400	-2.7222808
H	5.9763438	2.5243815	-2.9009268
H	7.5600151	2.2108936	-2.1777107
H	7.1111695	1.4095330	-3.6973628

1H(+)·9 (HB)

N	0.07982360	-3.04080060	-0.40616860
N	1.45522010	-1.15652720	-1.57911300
C	1.10565340	-3.46128210	-1.19512590
C	1.77668260	-2.39956120	-1.96699940
C	2.68586960	-2.66958840	-2.99594790
H	2.92406510	-3.68966690	-3.29161060
C	-0.65698360	-3.83479630	0.40121630
O	-5.85029240	1.93769080	1.72148780
C	2.09931200	-0.10976040	-2.12205900
C	3.28115910	-1.58288350	-3.62865320
H	3.99058440	-1.73996340	-4.44050040
C	1.45609720	-4.80379860	-1.15593420
H	2.30227490	-5.15705600	-1.73864140
C	2.99916600	-0.29286470	-3.18358860
H	3.50359550	0.56079180	-3.63016590
C	-1.80698820	-3.27415820	1.17241810
H	-1.83296040	-2.17913700	1.09676900
H	-1.61512980	-3.50901020	2.23282280
C	-5.42779150	0.63593720	1.78497620
C	0.76227760	-5.65948390	-0.29922430
H	1.06381030	-6.70366520	-0.21630480
C	-4.67543040	-2.08789870	1.71184100
C	-0.29525130	-5.18311930	0.46846230
H	-0.82140660	-5.82536220	1.17031560
C	-4.35686790	-1.27892790	2.80603250
H	-3.81021250	-1.70742790	3.64877530
C	-4.72981290	0.06945630	2.85912580
H	-4.48079580	0.65518920	3.74205680
C	-4.30664060	-3.55182250	1.67307140
H	-5.18669740	-4.13852560	1.36245130
H	-4.04338590	-3.89360880	2.68649150
C	-5.36885780	-1.49582360	0.64114890
H	-5.63637920	-2.10365540	-0.22691180
C	-3.14631380	-3.89276000	0.72025330
H	-3.04549330	-4.98853830	0.67157910
H	-3.38736370	-3.55421200	-0.30090180
C	-5.73941010	-0.15700600	0.66821080
H	-6.28524820	0.29831590	-0.15866690
C	1.85629370	1.21507230	-1.45289770
H	2.22720820	1.08485520	-0.42024070
H	0.77087060	1.36852060	-1.34924270
O	-3.02679740	4.88938340	-0.02100320
C	-5.86965770	4.23423550	2.37484610
H	-5.60010810	4.90599240	3.20653180
H	-6.95355540	4.34051690	2.21845750
C	0.44909170	3.67248520	0.38695740
H	1.07030610	3.14365930	1.11303930
C	1.00428420	4.05827160	-0.83634610
C	0.17297510	4.73585460	-1.74453570
H	0.57867210	5.05606810	-2.70715230
C	-1.15861240	5.00634780	-1.44813660
H	-1.80173060	5.53151320	-2.15493960
C	-1.70307260	4.59689100	-0.22025510
C	-0.88740880	3.93191160	0.70610430
H	-1.27410460	3.61168410	1.67140290
C	2.51235220	2.43688600	-2.09429940
H	2.04554940	2.64495880	-3.07183830
H	3.57766330	2.23103850	-2.28359340
C	-3.63589980	4.38170160	1.17199230
H	-3.19260130	4.87128680	2.06090060
H	-3.44270860	3.29738910	1.24606490

C	-5.12987120	4.64872680	1.09237050
H	-5.53690570	4.09622310	0.23395240
H	-5.29489960	5.71921780	0.89903490
C	2.42199710	3.69127170	-1.20103320
H	2.90871580	4.53231690	-1.71974300
H	3.00215640	3.50924340	-0.28150510
C	-5.56198980	2.80975660	2.82206290
H	-6.17170620	2.52411450	3.69794720
H	-4.50009200	2.71656610	3.11077020
H	-0.02017900	-2.00945640	-0.40409280
C	1.53891357	0.03652548	6.09442824
C	2.80431489	0.04449384	6.70737024
C	2.97237973	0.56554898	7.99017469
C	1.89793202	1.12248770	8.69560383
C	0.63521635	1.09305586	8.08080422
C	0.44242823	0.54821881	6.81345694
H	3.66588804	-0.34979680	6.16592602
H	3.96755161	0.55932718	8.44170744
H	-0.22798753	1.49852845	8.61543347
H	-0.54646862	0.53859373	6.36393330
S	1.44138615	-0.68053140	4.48271724
C	-0.01352579	0.09919117	3.62987478
C	-0.20336245	-0.43219296	2.35492394
H	0.41373067	-1.25110368	1.99208499
C	-1.15752064	0.13466978	1.43260664
O	-0.65092891	1.03923556	4.17653514
O	-1.93706444	1.16160283	1.91771622
H	-1.66859996	1.27929030	2.88357748
O	-1.27088679	-0.18023940	0.24055459
C	2.07376129	1.71296963	10.07152318
H	1.60745128	2.70797613	10.14349508
H	1.60078976	1.08224788	10.84084309
H	3.13556077	1.81696411	10.33324707

4aH(+)·9 (via bipy surface)

O	6.6814745	-1.7722870	-5.2169398
O	3.3009845	-0.8103959	0.9108007
O	3.5294248	0.8067695	-7.3269056
O	0.3616875	2.0404882	-1.1408230
N	5.0707590	-0.5295038	-6.2622609
N	-2.0660917	-0.8249802	1.5712560
N	1.8852796	0.6850289	-0.1053464
N	-1.3406103	-1.8100684	0.9851201
N	-0.1433293	-1.3105102	0.7751191
C	3.3902636	0.6854356	-4.9552105
C	3.9074430	0.0957348	-3.7770949
C	3.2734470	0.3568826	-2.5279848
C	5.0469526	-0.7435262	-3.8181192
C	3.7777784	-0.2498394	-1.3490857
C	5.5767226	-1.2506858	-2.6322702
H	6.4848153	-1.8508256	-2.6872060
C	5.6820086	-1.0607141	-5.1101481
C	2.1381614	1.1989554	-2.4878333
C	3.9786688	0.3612981	-6.2694838
C	4.9417890	-1.0135488	-1.4085805
H	5.3333202	-1.4312540	-0.4817733
C	-0.0840547	-0.0149131	1.2086055
C	3.0316687	-0.1364760	-0.0853152
C	2.3126679	1.5643577	-4.8757380
H	1.9571794	2.0294329	-5.7950300
C	1.6869966	1.8170811	-3.6502577
H	0.8264379	2.4810863	-3.5821149
C	1.3957285	1.3769538	-1.2218764
C	-1.3250300	0.3078519	1.7180279
H	-1.7426397	1.2369563	2.0851354
C	-4.3028714	-1.5553404	0.9617431
H	-3.8898428	-1.8716106	0.0082186
C	-3.4364900	-1.0086386	1.9107260
C	5.7119172	-0.8522547	-7.5376229
H	6.6921916	-0.3605879	-7.5954482
H	5.0662071	-0.4923680	-8.3422016
C	1.1398393	0.8341448	1.1506820
H	0.8747551	1.8905861	1.2624628
H	1.8280084	0.5673593	1.9607447
C	-5.6642679	-1.6557104	1.2463414
C	-3.8857438	-0.6017828	3.1607209
H	-3.1635245	-0.2025960	3.8720346
C	-5.2445780	-0.7250157	3.4914271
C	-6.1076667	-1.2330159	2.5149210
H	-7.1688504	-1.3147123	2.7421558

C	-6.6776452	-2.1720089	0.2150912
C	-5.7167705	-0.3317657	4.8997829
C	-7.6811294	-1.0400162	-0.1045654
H	-8.2301844	-0.7186316	0.7909844
H	-8.4168060	-1.3824670	-0.8471576
H	-7.1617323	-0.1621323	-0.5149628
C	-6.0019869	-2.6041540	-1.0978324
H	-5.4798480	-1.7671698	-1.5846473
H	-6.7637918	-2.9693664	-1.8007265
H	-5.2790910	-3.4168045	-0.9368760
C	-7.4340060	-3.3871124	0.7963440
H	-6.7361271	-4.2008916	1.0389139
H	-8.1642238	-3.7646171	0.0652014
H	-7.9831778	-3.1275680	1.7119112
C	-4.9316979	-1.1626119	5.9406313
H	-5.0849254	-2.2388975	5.7776769
H	-5.2694963	-0.9149386	6.9577052
H	-3.8518083	-0.9646406	5.8904646
C	-5.4448806	1.1688222	5.1373837
H	-4.3809880	1.4098863	5.0108900
H	-5.7363433	1.4537745	6.1588712
H	-6.0217713	1.7855773	4.4331537
C	-7.2183034	-0.5904087	5.1085504
H	-7.8360570	-0.0009726	4.4152381
H	-7.5021366	-0.3006144	6.1299624
H	-7.4699625	-1.6534856	4.9822731
O	0.1430828	0.9530856	5.5065653
N	1.6196469	-2.7599612	-0.9072605
H	0.8527387	-2.1886291	-0.4172143
N	-0.1943992	-1.3079951	-2.2268005
O	-2.4121550	4.5152117	2.9897952
C	0.7869256	-1.8145638	-2.9853469
C	2.1422009	-3.5483938	1.3532227
H	1.4580776	-4.4024064	1.5110706
H	1.5746201	-2.6527905	1.6350616
C	1.9990223	-0.0444570	4.4737798
H	2.4010907	0.9576847	4.3157446
C	1.7668463	-2.6497575	-2.2586052
C	-1.1230785	-0.5118939	-2.7780712
C	2.1932059	-2.4603086	4.1996662
C	0.7540481	-0.2069245	5.1016275
C	2.4502271	-3.4793463	-0.1121078
C	3.3679112	-3.6786920	2.2629009
H	3.9205101	-4.6092795	2.0560480
H	4.0429354	-2.8391144	2.0461648
C	2.7015306	-1.1589146	4.0322055
H	3.6532588	-0.9963200	3.5254470
C	0.2196523	-1.4897837	5.2694117
H	-0.7397682	-1.6445532	5.7604466
C	2.8270690	-3.3164324	-2.8689306
H	2.9762570	-3.2349076	-3.9415316
C	0.8908154	-1.5598403	-4.3603874
H	1.7110052	-1.9478453	-4.9607594
C	-4.4154698	2.0800854	1.0399606
H	-5.2638668	1.4040933	1.1732023
C	0.9440188	-2.5970495	4.8103482
H	0.5201279	-3.5948549	4.9472801
C	-3.9982028	2.8560636	2.1224831
H	-4.5313471	2.7889401	3.0679150
C	-2.1612156	0.0121487	-1.8249792
H	-2.6004123	-0.8585554	-1.3133953
H	-1.6214423	0.5673603	-1.0415971
C	3.5265185	-4.1375261	-0.7125170
H	4.2154730	-4.7018415	-0.0895290
C	-1.0877851	-0.2058834	-4.1488700
H	-1.8371901	0.4508703	-4.5873668
C	-2.6465066	2.9779808	-0.3095180
H	-2.0710080	3.0174514	-1.2336955
C	-2.8874169	3.7023095	1.9889693
C	-3.7628678	2.1335491	-0.1986200
C	-2.2045706	3.7451462	0.7663527
H	-1.3134208	4.3662576	0.6810961
C	-0.0705029	-0.7369201	-4.9400426
H	-0.0081921	-0.4914021	-6.0002575
C	3.7145551	-4.0513539	-2.0884438
H	4.5633690	-4.5479099	-2.5586238
C	2.9762484	-3.6759678	3.7520710
H	2.3877428	-4.5816860	3.9733020
H	3.9016485	-3.7573725	4.3460706
C	-4.2941276	1.3197274	-1.3622141

H	-5.0890991	1.8964276	-1.8660287
H	-4.7877925	0.4220395	-0.9549976
C	-1.8324380	2.7488093	4.5708412
H	-2.3381785	1.9631655	3.9874303
H	-0.8190544	2.8400306	4.1561385
C	-3.2638847	0.8872065	-2.4147838
H	-3.7939976	0.3429346	-3.2140054
H	-2.8212813	1.7773813	-2.8905941
C	-1.1545086	0.8883154	6.0974511
H	-1.0848964	0.5137879	7.1344883
H	-1.7975691	0.1897223	5.5284089
C	-2.5631667	4.0652409	4.3499714
H	-3.6303454	3.9904504	4.6290117
H	-2.1187895	4.8703826	4.9511920
C	-1.7478714	2.2894481	6.0274154
H	-2.7457474	2.2663498	6.4956967
H	-1.1287339	2.9827668	6.6176458
H	5.8679132	-1.9347195	-7.6061654
C	6.2591997	2.4434148	-3.3253316
C	7.1183036	1.6956671	-4.1533618
C	7.1026525	1.8749932	-5.5344209
C	6.2123791	2.7699223	-6.1435628
C	5.3612114	3.5073781	-5.3044933
C	5.3837780	3.3712469	-3.9182066
H	7.7926509	0.9601167	-3.7118979
H	7.7826962	1.2825986	-6.1503908
H	4.6559729	4.2122158	-5.7514172
H	4.7144656	3.9537683	-3.2912544
S	6.4387528	2.1539681	-1.5917669
C	4.8703988	2.6248950	-0.7628595
C	4.8508122	2.1520289	0.5572482
H	5.6505091	1.5187837	0.9344712
C	3.8033164	2.5043565	1.5010992
O	3.9606695	3.2790550	-1.3346932
O	2.8334987	3.3556001	1.0465746
H	3.0438824	3.5361717	0.0799744
O	3.7382117	2.0614703	2.6486259
C	6.1391630	2.9088156	-7.6404397
H	5.2598076	2.3723812	-8.0329536
H	6.0432444	3.9617704	-7.9422906
H	7.0323355	2.4930841	-8.1271265

4aH(+)·9 (via NDI surface)

O	6.6814745	-1.7722870	-5.2169398
O	3.3009845	-0.8103959	0.9108007
O	3.5294248	0.8067695	-7.3269056
O	0.3616875	2.0404882	-1.1408230
N	5.0707590	-0.5295038	-6.2622609
N	-2.0660917	-0.8249802	1.5712560
N	1.8852796	0.6850289	-0.1053464
N	-1.3406103	-1.8100684	0.9851201
N	-0.1433293	-1.3105102	0.7751191
C	3.3902636	0.6854356	-4.9552105
C	3.9074430	0.0957348	-3.7770949
C	3.2734470	0.3568826	-2.5279848
C	5.0469526	-0.7435262	-3.8181192
C	3.7777784	-0.2498394	-1.3490857
C	5.5767226	-1.2506858	-2.6322702
H	6.4848153	-1.8508256	-2.6872060
C	5.6820086	-1.0607141	-5.1101481
C	2.1381614	1.1989554	-2.4878333
C	3.9786688	0.3612981	-6.2694838
C	4.9417890	-1.0135488	-1.4085805
H	5.3333202	-1.4312540	-0.4817733
C	-0.0840547	-0.0149131	1.2086055
C	3.0316687	-0.1364760	-0.0853152
C	2.3126679	1.5643577	-4.8757380
H	1.9571794	2.0294329	-5.7950300
C	1.6869966	1.8170811	-3.6502577
H	0.8264379	2.4810863	-3.5821149
C	1.3957285	1.3769538	-1.2218764
C	-1.3250300	0.3078519	1.7180279
H	-1.7426397	1.2369563	2.0851354
C	-4.3028714	-1.5553404	0.9617431
H	-3.8898428	-1.8716106	0.0082186
C	-3.4364900	-1.0086386	1.9107260
C	5.7119172	-0.8522547	-7.5376229
H	6.6921916	-0.3605879	-7.5954482
H	5.0662071	-0.4923680	-8.3422016
C	1.1398393	0.8341448	1.1506820

H	0.8747551	1.8905861	1.2624628
H	1.8280084	0.5673593	1.9607447
C	-5.6642679	-1.6557104	1.2463414
C	-3.8857438	-0.6017828	3.1607209
H	-3.1635245	-0.2025960	3.8720346
C	-5.2445780	-0.7250157	3.4914271
C	-6.1076667	-1.2330159	2.5149210
H	-7.1688504	-1.3147123	2.7421558
C	-6.6776452	-2.1720089	0.2150912
C	-5.7167705	-0.3317657	4.8997829
C	-7.6811294	-1.0400162	-0.1045654
H	-8.2301844	-0.7186316	0.7909844
H	-8.4168060	-1.3824670	-0.8471576
H	-7.1617323	-0.1621323	-0.5149628
C	-6.0019869	-2.6041540	-1.0978324
H	-5.4798480	-1.7671698	-1.5846473
H	-6.7637918	-2.9693664	-1.8007265
H	-5.2790910	-3.4168045	-0.9368760
C	-7.4340060	-3.3871124	0.7963440
H	-6.7361271	-4.2008916	1.0389139
H	-8.1642238	-3.7646171	0.0652014
H	-7.9831778	-3.1275680	1.7119112
C	-4.9316979	-1.1626119	5.9406313
H	-5.0849254	-2.2388975	5.7776769
H	-5.2694963	-0.9149386	6.9577052
H	-3.8518083	-0.9646406	5.8904646
C	-5.4448806	1.1688222	5.1373837
H	-4.3809880	1.4098863	5.0108900
H	-5.7363433	1.4537745	6.1588712
H	-6.0217713	1.7855773	4.4331537
C	-7.2183034	-0.5904087	5.1085504
H	-7.8360570	-0.0009726	4.4152381
H	-7.5021366	-0.3006144	6.1299624
H	-7.4699625	-1.6534856	4.9822731
O	0.1430828	0.9530856	5.5065653
N	1.6196469	-2.7599612	-0.9072605
H	0.8527387	-2.1886291	-0.4172143
N	-0.1943992	-1.3079951	-2.2268005
O	-2.4121550	4.5152117	2.9897952
C	0.7869256	-1.8145638	-2.9853469
C	2.1422009	-3.5483938	1.3532227
H	1.4580776	-4.4024064	1.5110706
H	1.5746201	-2.6527905	1.6350616
C	1.9990223	-0.0444570	4.4737798
H	2.4010907	0.9576847	4.3157446
C	1.7668463	-2.6497575	-2.2586052
C	-1.1230785	-0.5118939	-2.7780712
C	2.1932059	-2.4603086	4.1996662
C	0.7540481	-0.2069245	5.1016275
C	2.4502271	-3.4793463	-0.1121078
C	3.3679112	-3.6786920	2.2629009
H	3.9205101	-4.6092795	2.0560480
H	4.0429354	-2.8391144	2.0461648
C	2.7015306	-1.1589146	4.0322055
H	3.6532588	-0.9963200	3.5254470
C	0.2196523	-1.4897837	5.2694117
H	-0.7397682	-1.6445532	5.7604466
C	2.8270690	-3.3164324	-2.8689306
H	2.9762570	-3.2349076	-3.9415316
C	0.8908154	-1.5598403	-4.3603874
H	1.7110052	-1.9478453	-4.9607594
C	-4.4154698	2.0800854	1.0399606
H	-5.2638668	1.4040933	1.1732023
C	0.9440188	-2.5970495	4.8103482
H	0.5201279	-3.5948549	4.9472801
C	-3.9982028	2.8560636	2.1224831
H	-4.5313471	2.7889401	3.0679150
C	-2.1612156	0.0121487	-1.8249792
H	-2.6004123	-0.8585554	-1.3133953
H	-1.6214423	0.5673603	-1.0415971
C	3.5265185	-4.1375261	-0.7125170
H	4.2154730	-4.7018415	-0.0895290
C	-1.0877851	-0.2058834	-4.1488700
H	-1.8371901	0.4508703	-4.5873668
C	-2.6465066	2.9779808	-0.3095180
H	-2.0710080	3.0174514	-1.2336955
C	-2.8874169	3.7023095	1.9889693
C	-3.7628678	2.1335491	-0.1986200
C	-2.2045706	3.7451462	0.7663527
H	-1.3134208	4.3662576	0.6810961

C	-0.0705029	-0.7369201	-4.9400426
H	-0.0081921	-0.4914021	-6.0002575
C	3.7145551	-4.0513539	-2.0884438
H	4.5633690	-4.5479099	-2.5586238
C	2.9762484	-3.6759678	3.7520710
H	2.3877428	-4.5816860	3.9733020
H	3.9016485	-3.7573725	4.3460706
C	-4.2941276	1.3197274	-1.3622141
H	-5.0890991	1.8964276	-1.8660287
H	-4.7877925	0.4220395	-0.9549976
C	-1.8324380	2.7488093	4.5708412
H	-2.3381785	1.9631655	3.9874303
H	-0.8190544	2.8400306	4.1561385
C	-3.2638847	0.8872065	-2.4147838
H	-3.7939976	0.3429346	-3.2140054
H	-2.8212813	1.7773813	-2.8905941
C	-1.1545086	0.8883154	6.0974511
H	-1.0848964	0.5137879	7.1344883
H	-1.7975691	0.1897223	5.5284089
C	-2.5631667	4.0652409	4.3499714
H	-3.6303454	3.9904504	4.6290117
H	-2.1187895	4.8703826	4.9511920
C	-1.7478714	2.2894481	6.0274154
H	-2.7457474	2.2663498	6.4956967
H	-1.1287339	2.9827668	6.6176458
H	5.8679132	-1.9347195	-7.6061654
C	6.2591997	2.4434148	-3.3253316
C	7.1183036	1.6956671	-4.1533618
C	7.1026525	1.8749932	-5.5344209
C	6.2123791	2.7699223	-6.1435628
C	5.3612114	3.5073781	-5.3044933
C	5.3837780	3.3712469	-3.9182066
H	7.7926509	0.9601167	-3.7118979
H	7.7826962	1.2825986	-6.1503908
H	4.6559729	4.2122158	-5.7514172
H	4.7144656	3.9537683	-3.2912544
S	6.4387528	2.1539681	-1.5917669
C	4.8703988	2.6248950	-0.7628595
C	4.8508122	2.1520289	0.5572482
H	5.6505091	1.5187837	0.9344712
C	3.8033164	2.5043565	1.5010992
O	3.9606695	3.2790550	-1.3346932
O	2.8334987	3.3556001	1.0465746
H	3.0438824	3.5361717	0.0799744
O	3.7382117	2.0614703	2.6486259
C	6.1391630	2.9088156	-7.6404397
H	5.2598076	2.3723812	-8.0329536
H	6.0432444	3.9617704	-7.9422906
H	7.0323355	2.4930841	-8.1271265

4bH(+)·9 (via bipy surface)

O	7.1143745	-0.2794062	-6.0498690
O	3.9450963	0.8216004	0.1655328
O	3.5570283	1.6740018	-8.1888964
O	0.7099151	3.3198505	-1.9141450
N	5.3528769	0.7184405	-7.1116863
N	-1.6236370	0.4616737	0.6019780
N	2.3059530	2.0481186	-0.8813989
N	-0.7670854	-0.5590062	0.3851007
N	0.4315773	-0.0346804	0.2727661
C	3.5320186	1.7389321	-5.8104636
C	4.1922372	1.3546063	-4.6188102
C	3.5833245	1.6327761	-3.3613344
C	5.4337863	0.6757951	-4.6534328
C	4.2022046	1.1768592	-2.1719773
C	6.0568674	0.3007462	-3.4676052
H	7.0172609	-0.2112465	-3.5247266
C	6.0526709	0.3314882	-5.9554372
C	2.3565924	2.3349672	-3.3230082
C	4.1143959	1.3978836	-7.1313857
C	5.4347474	0.5358490	-2.2316789
H	5.8881166	0.1957337	-1.3012442
C	0.3555519	1.3201912	0.4306757
C	3.5074428	1.3203949	-0.8688769
C	2.3189739	2.4179247	-5.7447450
H	1.8254001	2.6916713	-6.6768252
C	1.7414240	2.7308150	-4.5051901
H	0.7907900	3.2591904	-4.4467397
C	1.7151382	2.6242087	-2.0216031
C	-0.9693853	1.6502681	0.6330021
H	-1.4794847	2.5994532	0.7339460
C	-3.0151465	-1.0321474	2.9572722
H	-2.3888407	-1.7692975	2.4547579
C	-3.4533728	0.0747978	2.2189410
C	5.9762275	0.3597361	-8.3910823
H	6.9827345	0.7920158	-8.4420267
H	5.3450671	0.7476275	-9.1940345
C	-3.0564214	0.2123461	0.7657435
H	-3.2714355	-0.6937974	0.1838851
H	-3.5789279	1.0538409	0.2952628
C	1.5649861	2.1951451	0.3888464
H	1.2689879	3.2450195	0.4970622
H	2.2587332	1.9179829	1.1913396
C	-3.3624646	-1.1760664	4.3051401
C	-4.2574527	1.0430141	2.8239472
H	-4.5824429	1.8930760	2.2244082
C	-4.6470807	0.9233458	4.1679824
C	-4.1746678	-0.1843408	4.8804533
H	-4.4664233	-0.2975085	5.9242961
C	-2.9130070	-2.3697889	5.1618594
C	-5.6431896	1.8960847	4.8185821
C	-4.1596092	-3.1029895	5.7082767
H	-4.7709259	-2.4522872	6.3483939
H	-3.8542662	-3.9718491	6.3101029
H	-4.7941877	-3.4595661	4.8843529
C	-2.0783425	-3.3806167	4.3592738
H	-2.6460195	-3.8022648	3.5173148
H	-1.7792039	-4.2127206	5.0121964
H	-1.1605141	-2.9272056	3.9624067
C	-2.0599430	-1.8568282	6.3448184
H	-1.1513557	-1.3527134	5.9862211
H	-1.7520631	-2.6974902	6.9844771
H	-2.6193524	-1.1466660	6.9695051
C	-5.7066953	3.2397673	4.0714975
H	-4.7154214	3.7124157	4.0222860
H	-6.3843780	3.9287529	4.5946404
H	-6.0872528	3.1250329	3.0471103
C	-7.0412668	1.2352799	4.7674960
H	-7.3353371	1.0266013	3.7290023
H	-7.7978956	1.8972443	5.2158943
H	-7.0448372	0.2837143	5.3171115
C	-5.2792663	2.1832069	6.2903674
H	-5.3346303	1.2829377	6.9159827
H	-5.9822223	2.9154981	6.7130507
H	-4.2638748	2.5943161	6.3806045
O	0.2949792	2.0364335	4.2857090
N	2.1422024	-1.4985526	-1.8138722
H	1.2896802	-1.1029442	-1.3671593
N	0.2693232	-0.0338670	-3.0921044
O	-2.1006851	5.8248847	1.8328197

C	1.1411902	-0.7012268	-3.8678801
C	2.7264046	-2.2659086	0.4186838
H	2.0747391	-3.1541020	0.5018354
H	2.0972917	-1.4144021	0.7161876
C	2.3765196	1.0899707	3.7410224
H	2.8006819	2.0865927	3.8699961
C	2.2465872	-1.3740848	-3.1622280
C	-0.7953003	0.5572902	-3.6657255
C	2.6340946	-1.2805118	3.2410089
C	0.9976319	0.9135139	3.9448770
C	3.0573180	-2.1165159	-1.0269388
C	3.9315226	-2.3865338	1.3495682
H	4.5245830	-3.2867410	1.1179207
H	4.5817713	-1.5115739	1.1935076
C	3.1751742	0.0071672	3.3898570
H	4.2414683	0.1673115	3.2193717
C	0.4361913	-0.3575287	3.7826748
H	-0.6331503	-0.5150137	3.9012403
C	3.3856223	-1.8835984	-3.7801864
H	3.5035675	-1.7874791	-4.8551889
C	1.0135143	-0.8004023	-5.2585319
H	1.7095984	-1.3881620	-5.8525152
C	-4.2446608	3.5528084	-0.1554437
H	-5.1754557	2.9875135	-0.0563851
C	1.2586713	-1.4303284	3.4321237
H	0.8072354	-2.4144324	3.2888507
C	-3.8299988	4.3602303	0.9037143
H	-4.4294271	4.4173661	1.8103807
C	-1.7727476	1.2235068	-2.7382959
H	-2.1939597	0.4237010	-2.1086659
H	-1.2003671	1.8736855	-2.0623206
C	4.2125681	-2.6039858	-1.6309424
H	4.9624493	-3.0990870	-1.0219456
C	-0.9811426	0.5139542	-5.0592717
H	-1.8533767	0.9833956	-5.5075031
C	-2.3307418	4.2054105	-1.4389770
H	-1.7090363	4.1461810	-2.3311421
C	-2.6226360	5.0673687	0.8076523
C	-3.4988599	3.4359095	-1.3389228
C	-1.8881790	5.0017958	-0.3815424
H	-0.9441380	5.5418725	-0.4498347
C	-0.0691807	-0.1678325	-5.8578517
H	-0.2116875	-0.2248191	-6.9372743
C	4.3688633	-2.4958438	-3.0114832
H	5.2649252	-2.8869690	-3.4910655
C	3.4962798	-2.4502746	2.8267331
H	2.9410969	-3.3865056	2.9984437
H	4.3975347	-2.4990141	3.4581738
C	-3.9719803	2.5000318	-2.4331700
H	-4.7687398	3.0020278	-3.0093480
H	-4.4551167	1.6263974	-1.9654646
C	-1.7065125	3.8918971	3.2835014
H	-2.3090536	3.2174740	2.6567139
H	-0.6889965	3.8900832	2.8653952
C	-2.9136382	1.9835979	-3.4152431
H	-3.4309420	1.3006123	-4.1060789
H	-2.5150628	2.8176470	-4.0192027
C	-1.0898503	1.8974491	4.6513952
H	-1.1642792	1.3829396	5.6252637
H	-1.6330339	1.2945805	3.9047101
C	-2.2643214	5.3038879	3.1710417
H	-3.3234199	5.3513913	3.4831977
H	-1.7043512	6.0061632	3.8040061
C	-1.6765965	3.3009547	4.6920553
H	-2.6957155	3.2321176	5.1021206
H	-1.0866529	3.9277086	5.3795444
H	6.0691153	-0.7308657	-8.4665720
C	1.9067993	-4.9391313	-3.0067381
C	3.1252475	-5.2742818	-3.6282857
C	4.1837335	-5.8036868	-2.8894138
C	4.0838832	-5.9860738	-1.5044772
C	2.8584019	-5.6657824	-0.8971790
C	1.7772453	-5.1726224	-1.6241627
H	3.2438713	-5.1088182	-4.7009236
H	5.1158851	-6.0558124	-3.4008145
H	2.7420078	-5.8187244	0.1791833
H	0.8419274	-4.9331228	-1.1254794
S	0.6404514	-4.2877321	-4.0485198
C	-0.5167693	-3.3039069	-2.9609070
C	-1.6122711	-2.8419439	-3.6840676

H	-1.7226477	-3.0747179	-4.7406907
C	-2.6143227	-1.9763132	-3.0954412
O	-0.2278276	-3.0839662	-1.7553688
O	-2.5122180	-1.7639141	-1.7385436
H	-1.6579955	-2.1917942	-1.4281712
O	-3.5154325	-1.4115333	-3.7166847
C	5.2314131	-6.5260469	-0.6900299
H	5.4236383	-5.9006351	0.1954917
H	5.0167429	-7.5418893	-0.3224918
H	6.1576596	-6.5750247	-1.2782458

4bH(+)·9 (via NDI surface)

O	6.4513431	-1.6987718	-5.8803595
O	3.1065605	-0.6410771	0.2545349
O	3.2458945	0.7851234	-8.0239140
O	0.0742342	2.0642298	-1.8519178
N	4.8154824	-0.5030312	-6.9411764
N	-2.2680124	-0.8103189	0.8467554
N	1.6421730	0.7824003	-0.7921780
N	-1.4741117	-1.8109923	0.4105862
N	-0.2802224	-1.2874016	0.2253424
C	3.1209984	0.7087668	-5.6496852
C	3.6569377	0.1556495	-4.4622999
C	3.0242968	0.4300603	-3.2153919
C	4.8105635	-0.6645396	-4.4926940
C	3.5494723	-0.1368589	-2.0259647
C	5.3563008	-1.1363346	-3.2992806
H	6.2735899	-1.7230722	-3.3469927
C	5.4418497	-1.0000537	-5.7819631
C	1.8683199	1.2438126	-3.1874887
C	3.7084370	0.3689070	-6.9602924
C	4.7270636	-0.8802943	-2.0765714
H	5.1335695	-1.2684555	-1.1433359
C	-0.2982563	0.0434303	0.5391286
C	2.8114576	-0.0055403	-0.7587380
C	2.0225932	1.5626712	-5.5833696
H	1.6519583	1.9996520	-6.5104531
C	1.3952519	1.8241283	-4.3607105
H	0.5181045	2.4670864	-4.3028265
C	1.1278813	1.4296058	-1.9218191
C	-1.5835431	0.3578295	0.9359506
H	-2.0501090	1.2940187	1.2140657
C	-3.4960722	-2.1791283	3.3667218
H	-2.8320784	-2.8968639	2.8851519
C	-4.0079250	-1.1263532	2.5974816
C	5.4543789	-0.8421042	-8.2134714
H	6.4301029	-0.3430558	-8.2836332
H	4.8020995	-0.5017143	-9.0212099
C	-3.6834273	-1.0447696	1.1214693
H	-3.9301087	-1.9860347	0.6119735
H	-4.2449464	-0.2295068	0.6459372
C	0.8950753	0.9339024	0.4645450
H	0.5947057	1.9826709	0.5533563
H	1.5942792	0.7069634	1.2769557
C	-3.8269456	-2.3000997	4.7208170
C	-4.8483106	-0.1761444	3.1800407
H	-5.2242737	0.6352802	2.5566939
C	-5.2088054	-0.2657112	4.5347719
C	-4.6795984	-1.3295142	5.2737788
H	-4.9558173	-1.4236735	6.3235588
C	-3.3367618	-3.4603037	5.6016171
C	-6.2336689	0.6883048	5.1677725
C	-4.5631694	-4.2456781	6.1221395
H	-5.2178229	-3.6181689	6.7422596
H	-4.2345212	-5.0965919	6.7371818
H	-5.1611697	-4.6350400	5.2855265
C	-2.4380933	-4.4380692	4.8276018
H	-2.9755037	-4.9095326	3.9919009
H	-2.0984943	-5.2385349	5.4999286
H	-1.5448046	-3.9420676	4.4256136
C	-2.5405607	-2.9045155	6.8042180
H	-1.6408199	-2.3679657	6.4724315
H	-2.2213002	-3.7288015	7.4590985
H	-3.1447859	-2.2130891	7.4071022
C	-6.3345078	2.0154176	4.3949309
H	-5.3554618	2.5098868	4.3278166
H	-7.0248202	2.6982842	4.9093137
H	-6.7203131	1.8724695	3.3759015
C	-7.6115373	-0.0147013	5.1287352
H	-7.9028849	-0.2441085	4.0937168

H	-8.3857456	0.6302911	5.5715604
H	-7.5859806	-0.9590699	5.6904081
C	-5.8805411	1.0111359	6.6346000
H	-5.9202820	0.1219277	7.2773461
H	-6.6006037	1.7358966	7.0406620
H	-4.8747851	1.4455184	6.7206953
O	-0.3282024	1.0622772	4.4987518
N	1.5325274	-2.7301765	-1.4476183
H	0.7629817	-2.1680632	-0.9549994
N	-0.3626617	-1.3743415	-2.8219812
O	-2.8635401	4.6350682	2.0708186
C	0.6477833	-1.8685049	-3.5519448
C	2.1427791	-3.4322540	0.8234937
H	1.5605463	-4.3439874	1.0535178
H	1.4969572	-2.5847039	1.0834665
C	1.7416611	0.2284460	3.7640520
H	2.0836619	1.2591994	3.6629281
C	1.6493332	-2.6592909	-2.8051855
C	-1.3089711	-0.6305685	-3.4182369
C	2.1620727	-2.1621907	3.5398788
C	0.4280308	-0.0339858	4.1850435
C	2.3956416	-3.4113502	-0.6523213
C	3.4137767	-3.3882896	1.6828249
H	4.0417896	-4.2768568	1.5115062
H	3.9941001	-2.5048853	1.3831425
C	2.5876893	-0.8254824	3.4410081
H	3.5905509	-0.5900341	3.0819240
C	-0.0307945	-1.3541322	4.2533236
H	-1.0562721	-1.5741035	4.5408207
C	2.7061027	-3.3318906	-3.4167765
H	2.8279708	-3.2865899	-4.4946018
C	0.7700772	-1.6447220	-4.9316018
H	1.6188355	-2.0123360	-5.5039662
C	-4.8842183	2.2652603	0.0718824
H	-5.8198774	1.7028028	0.1419230
C	0.8433800	-2.3980197	3.9342496
H	0.4842657	-3.4281775	3.9992798
C	-4.5148219	3.0934831	1.1318308
H	-5.1493390	3.1672523	2.0136085
C	-2.3895677	-0.1010731	-2.5127060
H	-2.8485117	-0.9633839	-2.0000853
H	-1.8826546	0.4750681	-1.7230794
C	3.4648651	-4.0772834	-1.2567613
H	4.1723608	-4.6150848	-0.6308445
C	-1.2595088	-0.3666384	-4.7974943
H	-2.0238239	0.2464009	-5.2711692
C	-2.9158878	2.9000471	-1.1436536
H	-2.2489268	2.8251406	-2.0007638
C	-3.3152928	3.8189178	1.0625734
C	-4.0946628	2.1427396	-1.0823194
C	-2.5219920	3.7195905	-0.0860004
H	-1.5761785	4.2596096	-0.1240109
C	-0.2075140	-0.8770844	-5.5544941
H	-0.1315969	-0.6569772	-6.6193518
C	3.6200145	-4.0340028	-2.6378335
H	4.4612614	-4.5382052	-3.1135076
C	3.0798246	-3.3091885	3.1850916
H	2.6143143	-4.2562232	3.5047197
H	4.0258767	-3.2225341	3.7428400
C	-4.5510843	1.2356953	-2.2097261
H	-5.3300857	1.7595406	-2.7904367
H	-5.0530515	0.3549427	-1.7732490
C	-2.4260118	2.7627918	3.5772275
H	-3.0266156	2.0420102	3.0005122
H	-1.4223957	2.7808614	3.1281186
C	-3.4654499	0.7551915	-3.1806884
H	-3.9570487	0.1811700	-3.9832540
H	-2.9975565	1.6257986	-3.6685516
C	-1.6583855	0.8653773	4.9970409
H	-1.6202468	0.4128279	6.0040446
H	-2.2255083	0.1847233	4.3377118
C	-3.0275172	4.1513439	3.4231331
H	-4.0911266	4.1802100	3.7227645
H	-2.4909954	4.8865752	4.0382228
C	-2.3201461	2.2366762	5.0071304
H	-3.3173892	2.1372195	5.4625183
H	-1.7348186	2.9224634	5.6395964
H	5.6197490	-1.9242304	-8.2646373
C	5.9614238	2.5640209	-4.0840351
C	6.8273616	1.8092358	-4.8984908

C	6.8012505	1.9542399	-6.2835199
C	5.8940094	2.8203891	-6.9090231
C	5.0370283	3.5662021	-6.0832417
C	5.0695607	3.4645905	-4.6942472
H	7.5156301	1.0955209	-4.4429304
H	7.4866424	1.3572170	-6.8891035
H	4.3193490	4.2497286	-6.5432062
H	4.3956726	4.0530360	-4.0779594
S	6.1531650	2.3211466	-2.3446990
C	4.5759637	2.7769218	-1.5226094
C	4.5732502	2.3410644	-0.1895766
H	5.3907565	1.7395167	0.2013322
C	3.5222290	2.6941071	0.7509599
O	3.6484016	3.3915025	-2.1087230
O	2.5263608	3.5042854	0.2771071
H	2.7273266	3.6630338	-0.6948453
O	3.4776549	2.2863640	1.9118969
C	5.8087845	2.9202966	-8.4082913
H	4.9343231	2.3616884	-8.7803216
H	5.6956946	3.9637404	-8.7361906
H	6.7041589	2.5046075	-8.8909096

4cH(+)·9 (via bipy surface)

O	7.69616220	-0.25559750	-6.27167460
O	3.60893010	-1.02533790	-0.55915040
O	4.44551110	2.33447750	-8.21059570
O	0.53762230	1.86017260	-2.35023000
N	6.02872730	0.96994860	-7.25336270
N	-1.64764030	-1.90988600	-0.58648920
N	2.12058880	0.49380100	-1.42287320
N	-0.70062330	-2.65250000	-1.18948020
N	0.40610430	-1.94106690	-1.15597290
C	4.08701550	1.69874830	-5.94507760
C	4.56004310	0.93289680	-4.85289400
C	3.77740680	0.85125550	-3.66465440
C	5.79931560	0.25085650	-4.91894340
C	4.23741880	0.06298820	-2.57825320
C	6.27134370	-0.43742670	-3.80153670
H	7.25167640	-0.90996710	-3.86203040
C	6.59811020	0.28990530	-6.15828350
C	2.54188670	1.53627820	-3.59876740
C	4.83878360	1.72394330	-7.21486470
C	5.49231270	-0.53878870	-2.64386740
H	5.84029020	-1.10294040	-1.77963180
C	0.18142910	-0.74615930	-0.53304020
C	3.35814950	-0.18302590	-1.42320430
C	2.89968060	2.41746880	-5.82710280
H	2.57746080	3.02425560	-6.67288090
C	2.12950350	2.33506550	-4.66189080
H	1.18527220	2.87034880	-4.57100200
C	1.65699200	1.35145190	-2.42903130
C	-1.14918840	-0.71650360	-0.16865950
H	-1.75279700	0.04820300	0.30603800
C	6.81630940	0.99874070	-8.48672710
H	7.62379680	1.73975270	-8.40081030
H	6.15435670	1.27868260	-9.31004620
C	-3.00158770	-2.41272620	-0.39148810
H	-3.04564790	-3.39352970	-0.87992780
H	-3.68797920	-1.72710150	-0.90985280
C	1.23519320	0.27424590	-0.27239010
H	0.77956630	1.23382440	-0.00976810
H	1.87029360	-0.04199840	0.56283320
O	-0.32628940	-0.75604190	3.97880800
N	2.47504450	-2.78168550	-2.82401260
H	1.59573090	-2.41157720	-2.31552540
N	0.60701360	-1.30125990	-4.05351970
O	-3.12438600	2.84700310	1.99509270
C	1.71987340	-1.52631560	-4.76553830
C	2.88420610	-3.94422270	-0.70839760
H	2.37385250	-4.92197890	-0.79230350
H	2.12442200	-3.23438960	-0.35989340
C	1.73328800	-1.18558180	2.94228690
H	1.94790280	-0.11765270	3.00818280
C	2.72973410	-2.38058390	-4.10150640
C	-0.35874190	-0.51167430	-4.54963100
C	2.41680180	-3.42952690	2.27472850
C	0.51145030	-1.68509590	3.42437440
C	3.31955400	-3.54331960	-2.08614590
C	4.00637540	-4.02781860	0.33174230
H	4.75324550	-4.78798530	0.05181380

H	4.51956430	-3.05670880	0.35948920
C	2.66539570	-2.04793160	2.37792300
H	3.59328450	-1.61732630	1.99940750
C	0.23186090	-3.05314320	3.31423010
H	-0.70335320	-3.46774370	3.68766690
C	3.92338780	-2.78322120	-4.69801260
H	4.16047500	-2.46925930	-5.71027270
C	1.92854550	-0.97393230	-6.03789960
H	2.84960050	-1.13386510	-6.59403770
C	-4.46394620	0.78176580	-0.77074370
H	-5.26263680	0.06008060	-0.96445410
C	1.18531090	-3.90275060	2.73757780
H	0.96163960	-4.97040660	2.66659250
C	-4.31523470	1.28524900	0.52426210
H	-4.96760690	0.93415760	1.32429670
C	-1.55262080	-0.31715070	-3.65124200
H	-1.95165940	-1.31757250	-3.41130550
H	-1.16761470	0.08196540	-2.70114240
C	4.52827790	-3.93595440	-2.66708520
H	5.22717070	-4.53039560	-2.08396450
C	-0.22248780	0.08319420	-5.81470160
H	-1.00053850	0.73712800	-6.20464820
C	-2.63562320	2.12529450	-1.54489460
H	-1.93118590	2.43838070	-2.31497090
C	-3.32200290	2.24428980	0.78166090
C	-3.63724720	1.18414640	-1.83051800
C	-2.47732850	2.64766100	-0.26287970
H	-1.68080010	3.35913790	-0.04540830
C	0.93207800	-0.15412060	-6.55908600
H	1.07019130	0.31826410	-7.53168700
C	4.82728890	-3.55288540	-3.97100980
H	5.77601920	-3.84213450	-4.42364760
C	3.45901890	-4.38314020	1.72652660
H	3.03271660	-5.39971680	1.69979100
H	4.31342450	-4.42850020	2.42278450
C	-3.87982550	0.63992980	-3.22637970
H	-4.66648080	1.24747930	-3.70692360
H	-4.30465670	-0.37535680	-3.14033620
C	-2.52184930	0.82416210	3.22464210
H	-2.91174690	0.10968110	2.48507570
H	-1.50662690	1.09986950	2.90566940
C	-2.66840800	0.58695960	-4.16992540
H	-3.02217080	0.23189050	-5.15170530
H	-2.27574910	1.60372340	-4.33391580
C	-1.62267930	-1.15166320	4.44995800
H	-1.52504490	-1.68332190	5.41199360
H	-2.10931230	-1.83234370	3.73064310
C	-3.38130650	2.07600330	3.19005270
H	-4.45473820	1.83623620	3.28907950
H	-3.11707230	2.76212420	4.00596730
C	-2.45311540	0.11831550	4.57891500
H	-3.45780220	-0.15796090	4.93215210
H	-2.00427650	0.78091070	5.33629630
H	7.26719190	0.01442040	-8.64724040
C	2.35966125	-6.58248767	-3.84266842
C	2.76338000	-7.26613554	-2.67990785
C	3.86753716	-8.11445318	-2.70648184
C	4.58598926	-8.34074798	-3.88843734
C	4.17108048	-7.65160205	-5.03950086
C	3.08966855	-6.77321018	-5.02972958
H	2.19917250	-7.14286480	-1.75422696
H	4.15817788	-8.62985099	-1.78829218
H	4.71374571	-7.80584069	-5.97524370
H	2.78851048	-6.25211264	-5.93407610
S	0.98479805	-5.49132922	-3.64190494
C	0.63055245	-4.71188497	-5.26615362
C	-0.62187667	-4.08022966	-5.24521076
H	-1.27659690	-4.16913970	-4.38163747
C	-1.10445640	-3.25746850	-6.34190625
O	1.42486802	-4.76309892	-6.24007446
O	-0.25434763	-3.09245468	-7.40075005
H	0.55983010	-3.64729254	-7.19977491
O	-2.22388121	-2.74345188	-6.37046579
C	5.73361901	-9.31363512	-3.93433738
H	5.40411788	-10.27583589	-4.35902106
H	6.55395585	-8.93910865	-4.56335207
H	6.13508701	-9.50996550	-2.93017155
C	-3.31956130	-2.46293350	1.10411570
H	-2.86852450	-1.57605570	1.56763010
H	-2.83870870	-3.33332540	1.57227930

C	-4.79958230	-2.39613620	1.45036710
H	-5.31948680	-1.68471950	0.77645790
H	-5.30561730	-3.37710550	1.35347550
O	-4.85569970	-1.92233800	2.79211530
C	-6.18624980	-1.71123640	3.28336390
H	-6.75358310	-1.07077080	2.57994120
H	-6.71632050	-2.68113410	3.35991130
C	-6.05608690	-1.04506520	4.62464900
C	-6.33683210	0.31881980	4.77460770
C	-5.55938100	-1.77214740	5.70575920
C	-6.12451590	0.96696270	5.99519250
H	-6.72141810	0.86746130	3.91437700
C	-5.32316870	-1.16056380	6.94676040
H	-5.32911810	-2.82912030	5.55897990
C	-5.61450380	0.20293010	7.06035340
H	-5.44148240	0.70012190	8.01350910
C	-4.74043460	-1.98490880	8.10401240
C	-6.42676100	2.45684690	6.21366100
C	-6.93554990	3.14609130	4.93634330
H	-7.14370610	4.20460520	5.14698990
H	-6.19039560	3.11350450	4.12815770
H	-7.86746720	2.69218580	4.56968200
C	-7.51221520	2.59472070	7.30551060
H	-7.18441200	2.16074900	8.26014550
H	-7.74516600	3.65586440	7.47985180
H	-8.43737460	2.08377860	7.00208430
C	-5.14151810	3.18032160	6.67424910
H	-5.33963480	4.25215050	6.82352370
H	-4.76041750	2.77461320	7.62078830
H	-4.34720990	3.08052470	5.92156020
C	-4.62474030	-1.17505270	9.40618400
H	-3.95166670	-0.31260720	9.29466530
H	-5.60408730	-0.80937380	9.74738960
H	-4.21194360	-1.81319630	10.20048860
C	-5.64442110	-3.20784630	8.37453240
H	-6.66143600	-2.88952800	8.64514360
H	-5.71917660	-3.86489050	7.49718210
H	-5.23828620	-3.80514000	9.20464400
C	-3.32710580	-2.46569050	7.70523710
H	-2.88781030	-3.07709210	8.50779320
H	-3.34915240	-3.07248400	6.78967530
H	-2.66419090	-1.60748240	7.52477750

4cH(+)·9 (via bipy surface)

O	7.6961622	-0.2555975	-6.2716746
O	3.6089301	-1.0253379	-0.5591504
O	4.4455111	2.3344775	-8.2105957
O	0.5376223	1.8601726	-2.3502300
N	6.0287273	0.9699486	-7.2533627
N	-1.6476403	-1.9098860	-0.5864892
N	2.1205888	0.4938010	-1.4228732
N	-0.7006233	-2.6525000	-1.1894802
N	0.4061043	-1.9410669	-1.1559729
C	4.0870155	1.6987483	-5.9450776
C	4.5600431	0.9328968	-4.8528940
C	3.7774068	0.8512555	-3.6646544
C	5.7993156	0.2508565	-4.9189434
C	4.2374188	0.0629882	-2.5782532
C	6.2713437	-0.4374267	-3.8015367
H	7.2516764	-0.9099671	-3.8620304
C	6.5981102	0.2899053	-6.1582835
C	2.5418867	1.5362782	-3.5987674
C	4.8387836	1.7239433	-7.2148647
C	5.4923127	-0.5387887	-2.6438674
H	5.8402902	-1.1029404	-1.7796318
C	0.1814291	-0.7461593	-0.5330402
C	3.3581495	-0.1830259	-1.4232043
C	2.8996806	2.4174688	-5.8271028
H	2.5774608	3.0242556	-6.6728809
C	2.1295035	2.3350655	-4.6618908
H	1.1852722	2.8703488	-4.5710020
C	1.6569920	1.3514519	-2.4290313
C	-1.1491884	-0.7165036	-0.1686595
H	-1.7527970	0.0482030	0.3060380
C	6.8163094	0.9987407	-8.4867271
H	7.6237968	1.7397527	-8.4008103
H	6.1543567	1.2786826	-9.3100462
C	-3.0015877	-2.4127262	-0.3914881
H	-3.0456479	-3.3935297	-0.8799278
H	-3.6879792	-1.7271015	-0.9098528
C	1.2351932	0.2742459	-0.2723901

H	0.7795663	1.2338244	-0.0097681
H	1.8702936	-0.0419984	0.5628332
O	-0.3262894	-0.7560419	3.9788080
N	2.4750445	-2.7816855	-2.8240126
H	1.5957309	-2.4115772	-2.3155254
N	0.6070136	-1.3012599	-4.0535197
O	-3.1243860	2.8470031	1.9950927
C	1.7198734	-1.5263156	-4.7655383
C	2.8842061	-3.9442227	-0.7083976
H	2.3738525	-4.9219789	-0.7923035
H	2.1244220	-3.2343896	-0.3598934
C	1.7332880	-1.1855818	2.9422869
H	1.9479028	-0.1176527	3.0081828
C	2.7297341	-2.3805839	-4.1015064
C	-0.3587419	-0.5116743	-4.5496310
C	2.4168018	-3.4295269	2.2747285
C	0.5114503	-1.6850959	3.4243744
C	3.3195540	-3.5433196	-2.0861459
C	4.0063754	-4.0278186	0.3317423
H	4.7532455	-4.7879853	0.0518138
H	4.5195643	-3.0567088	0.3594892
C	2.6653957	-2.0479316	2.3779230
H	3.5932845	-1.6173263	1.9994075
C	0.2318609	-3.0531432	3.3142301
H	-0.7033532	-3.4677437	3.6876669
C	3.9233878	-2.7832212	-4.6980126
H	4.1604750	-2.4692593	-5.7102727
C	1.9285455	-0.9739323	-6.0378996
H	2.8496005	-1.1338651	-6.5940377
C	-4.4639462	0.7817658	-0.7707437
H	-5.2626368	0.0600806	-0.9644541
C	1.1853109	-3.9027506	2.7375778
H	0.9616396	-4.9704066	2.6665925
C	-4.3152347	1.2852490	0.5242621
H	-4.9676069	0.9341576	1.3242967
C	-1.5526208	-0.3171507	-3.6512420
H	-1.9516594	-1.3175725	-3.4113055
H	-1.1676147	0.0819654	-2.7011424
C	4.5282779	-3.9359544	-2.6670852
H	5.2271707	-4.5303956	-2.0839645
C	-0.2224878	0.0831942	-5.8147016
H	-1.0005385	0.7371280	-6.2046482
C	-2.6356232	2.1252945	-1.5448946
H	-1.9311859	2.4383807	-2.3149709
C	-3.3220029	2.2442898	0.7816609
C	-3.6372472	1.1841464	-1.8305180
C	-2.4773285	2.6476610	-0.2628797
H	-1.6808001	3.3591379	-0.0454083
C	0.9320780	-0.1541206	-6.5590860
H	1.0701913	0.3182641	-7.5316870
C	4.8272889	-3.5528854	-3.9710098
H	5.7760192	-3.8421345	-4.4236476
C	3.4590189	-4.3831402	1.7265266
H	3.0327166	-5.3997168	1.6997910
H	4.3134245	-4.4285002	2.4227845
C	-3.8798255	0.6399298	-3.2263797
H	-4.6664808	1.2474793	-3.7069236
H	-4.3046567	-0.3753568	-3.1403362
C	-2.5218493	0.8241621	3.2246421
H	-2.9117469	0.1096811	2.4850757
H	-1.5066269	1.0998695	2.9056694
C	-2.6684080	0.5869596	-4.1699254
H	-3.0221708	0.2318905	-5.1517053
H	-2.2757491	1.6037234	-4.3339158
C	-1.6226793	-1.1516632	4.4499580
H	-1.5250449	-1.6833219	5.4119936
H	-2.1093123	-1.8323437	3.7306431
C	-3.3813065	2.0760033	3.1900527
H	-4.4547382	1.8362362	3.2890795
H	-3.1170723	2.7621242	4.0059673
C	-2.4531154	0.1183155	4.5789150
H	-3.4578022	-0.1579609	4.9321521
H	-2.0042765	0.7809107	5.3362963
H	7.2671919	0.0144204	-8.6472404
C	6.4914665	3.3782886	-3.6193762
C	7.5001224	2.9218065	-4.4892111
C	7.5775780	3.4009794	-5.7945486
C	6.6423191	4.3187079	-6.2920089
C	5.6425748	4.7644342	-5.4123169
C	5.5642123	4.3240339	-4.0928373

H	8.2180253	2.1761994	-4.1441077
H	8.3722156	3.0298931	-6.4457208
H	4.9009961	5.4821994	-5.7711538
H	4.7807097	4.6841604	-3.4321452
S	6.5583183	2.7332651	-1.9760086
C	4.8719672	2.8256657	-1.2556690
C	4.7953793	2.0575816	-0.0842652
H	5.6329508	1.4415944	0.2340996
C	3.6263324	2.0576936	0.7793837
O	3.9398689	3.4929509	-1.7733611
O	2.5977763	2.8819844	0.4137586
H	2.8710010	3.3079355	-0.4551691
O	3.5140905	1.3461260	1.7789906
C	6.6821563	4.7804832	-7.7240867
H	5.9431903	4.2272504	-8.3262663
H	6.4424470	5.8501648	-7.8089561
H	7.6718616	4.6153300	-8.1727280
C	-3.3195613	-2.4629335	1.1041157
H	-2.8685245	-1.5760557	1.5676301
H	-2.8387087	-3.3333254	1.5722793
C	-4.7995823	-2.3961362	1.4503671
H	-5.3194868	-1.6847195	0.7764579
H	-5.3056173	-3.3771055	1.3534755
O	-4.8556997	-1.9223380	2.7921153
C	-6.1862498	-1.7112364	3.2833639
H	-6.7535831	-1.0707708	2.5799412
H	-6.7163205	-2.6811341	3.3599113
C	-6.0560869	-1.0450652	4.6246490
C	-6.3368321	0.3188198	4.7746077
C	-5.5593810	-1.7721474	5.7057592
C	-6.1245159	0.9669627	5.9951925
H	-6.7214181	0.8674613	3.9143770
C	-5.3231687	-1.1605638	6.9467604
H	-5.3291181	-2.8291203	5.5589799
C	-5.6145038	0.2029301	7.0603534
H	-5.4414824	0.7001219	8.0135091
C	-4.7404346	-1.9849088	8.1040124
C	-6.4267610	2.4568469	6.2136610
C	-6.9355499	3.1460913	4.9363433
H	-7.1437061	4.2046052	5.1469899
H	-6.1903956	3.1135045	4.1281577
H	-7.8674672	2.6921858	4.5696820
C	-7.5122152	2.5947207	7.3055106
H	-7.1844120	2.1607490	8.2601455
H	-7.7451660	3.6558644	7.4798518
H	-8.4373746	2.0837786	7.0020843
C	-5.1415181	3.1803216	6.6742491
H	-5.3396348	4.2521505	6.8235237
H	-4.7604175	2.7746132	7.6207883
H	-4.3472099	3.0805247	5.9215602
C	-4.6247403	-1.1750527	9.4061840
H	-3.9516667	-0.3126072	9.2946653
H	-5.6040873	-0.8093738	9.7473896
H	-4.2119436	-1.8131963	10.2004886
C	-5.6444211	-3.2078463	8.3745324
H	-6.6614360	-2.8895280	8.6451436
H	-5.7191766	-3.8648905	7.4971821
H	-5.2382862	-3.8051400	9.2046440
C	-3.3271058	-2.4656905	7.7052371
H	-2.8878103	-3.0770921	8.5077932
H	-3.3491524	-3.0724840	6.7896753
H	-2.6641909	-1.6074824	7.5247775

5H(+)·9

O	2.4195114	-1.7384798	2.1575550
O	0.9687238	2.6007866	2.2432064
N	-0.4992050	0.5057345	5.8035570
N	1.8042874	0.4730385	2.1413722
N	0.4616151	-0.1943905	6.4883475
N	1.5289190	-0.2191112	5.7340966
O	-0.6392540	2.1889865	-4.6375857
N	-0.0546256	-0.0368950	-4.6269192
C	-1.8032737	0.6778763	6.3351409
O	0.2286466	-2.3082712	-4.5652025
C	1.2868763	0.4606910	4.5674822
C	-0.0094055	0.9312294	4.6044334
H	-0.5613098	1.5560003	3.9151090
C	0.8946063	0.2218400	-0.5051318
C	-3.2535203	0.9197449	8.2553233
C	1.4081393	-0.9182674	0.1593668

C	1.9221973	-0.8011576	1.5384057
C	-1.9750835	0.7486928	7.7240053
H	-1.0913851	0.6660666	8.3512857
C	-2.8906140	0.7708357	5.4699854
H	-2.7238175	0.6756645	4.3974868
C	-4.3394596	1.0282066	7.3650081
H	-5.3357091	1.1699937	7.7798624
C	0.7740018	1.4684951	0.1525407
C	0.5159361	0.1192736	-1.8735505
C	1.1759149	1.5930456	1.5677566
C	2.3297365	0.6343170	3.5059504
H	2.7649856	1.6413504	3.5602473
H	3.1116165	-0.1183749	3.6516478
C	-3.5105965	1.0010941	9.7684731
C	-4.1853858	0.9574987	5.9778981
C	1.4641216	-2.1449534	-0.4998890
H	1.8754020	-3.0028629	0.0301045
C	0.0755540	1.2794992	-2.5573103
C	-5.3670069	1.0465738	5.0001947
C	0.6086973	-1.1309630	-2.5278310
C	1.0504396	-2.2577632	-1.8301509
H	1.1233775	-3.2063472	-2.3606491
C	-0.0457469	2.4913046	-1.8814341
H	-0.3664682	3.3703918	-2.4383265
C	0.2925278	2.5847142	-0.5263965
H	0.2463605	3.5358175	-0.0000424
C	-2.2160826	0.8781300	10.5899418
H	-1.7096935	-0.0818908	10.4138908
H	-2.4538966	0.9386897	11.6619373
H	-1.5089929	1.6872816	10.3584386
C	-4.1693266	2.3583087	10.1034316
H	-3.5157301	3.1907845	9.8061942
H	-4.3566785	2.4357089	11.1855319
H	-5.1307076	2.4831815	9.5862072
C	-5.1236615	2.2013851	4.0027188
H	-5.0285036	3.1603371	4.5320202
H	-5.9643312	2.2785727	3.2966040
H	-4.2088416	2.0498498	3.4147475
C	-4.4582117	-0.1479808	10.1824876
H	-5.4252817	-0.0844133	9.6651065
H	-4.6514525	-0.1123589	11.2656652
H	-4.0114899	-1.1236135	9.9433249
C	-0.2394401	1.2135829	-3.9985858
C	-0.6490839	-0.7148245	-11.0038154
H	-1.0391517	-0.7535120	-12.0222478
C	-0.6538212	-1.8608863	-10.2041949
H	-1.0495204	-2.8001780	-10.5950698
C	-5.4822862	-0.2870099	4.2269927
H	-4.5727973	-0.5037035	3.6502303
H	-6.3257570	-0.2469888	3.5207293
H	-5.6495974	-1.1245939	4.9192669
C	0.2592054	-1.2382678	-3.9530402
C	-0.1350905	0.4803600	-10.4913091
H	-0.1242548	1.3800851	-11.1091775
C	0.3709103	-0.6152850	-8.3797401
C	-0.3437561	-0.1384380	-6.0634537
H	-1.1387151	-0.8841003	-6.2045361
H	-0.7245031	0.8395615	-6.3758506
C	0.3710646	0.5283190	-9.1908302
H	0.7861975	1.4581149	-8.7981350
C	-0.1490281	-1.8079879	-8.9022991
H	-0.1554965	-2.7027092	-8.2753382
C	-6.7021425	1.3051250	5.7185973
H	-6.9553728	0.4924033	6.4151987
H	-7.5118966	1.3705988	4.9774677
H	-6.6858615	2.2514053	6.2789352
C	4.5496596	1.6650820	-6.4967690
H	5.4891683	2.2006544	-6.3554343
C	3.3370966	2.2771272	-6.1774765
H	3.3237644	3.2966171	-5.7895879
C	2.1319806	1.5825206	-6.3261269
H	1.1991818	2.0807847	-6.0621109
C	2.1247035	0.2561563	-6.7751383
C	3.3506440	-0.3449498	-7.1005651
H	3.3595562	-1.3810731	-7.4468306
C	4.5518895	0.3497624	-6.9689592
H	5.4945630	-0.1469732	-7.2032102
C	0.8599780	-0.5675982	-6.9379308
H	1.1055545	-1.5976234	-6.6419713
C	3.8438709	-2.0936355	-3.2322284

C	3.3210174	-2.9566947	-4.2158355
C	3.3334240	-4.3354911	-4.0290303
C	3.8528119	-4.9080831	-2.8575904
C	4.3701682	-4.0400299	-1.8862094
C	4.3826607	-2.6553337	-2.0606285
H	2.8644532	-2.5340218	-5.1108675
H	2.8995179	-4.9787831	-4.7985906
H	4.7710998	-4.4537362	-0.9571733
H	4.7631011	-1.9996917	-1.2817704
S	3.7375201	-0.3722237	-3.5982616
C	3.7853241	0.5668077	-2.0047450
C	3.4073081	1.8918712	-2.2268132
H	3.1111803	2.2235922	-3.2195951
C	3.3030128	2.8581249	-1.1528057
O	4.0972280	0.0322418	-0.9080588
O	3.6963427	2.4328518	0.0930203
H	3.9651086	1.4696683	-0.0199128
O	2.8560958	3.9996122	-1.2912982
C	3.8717206	-6.4041434	-2.6661706
H	4.7506245	-6.8587495	-3.1531150
H	3.9122006	-6.6721316	-1.6009631
H	2.9792188	-6.8764777	-3.1027119

6·9

O	-3.3992266	-2.4191542	2.0704763
O	-3.4897511	2.0907122	2.7911553
N	0.7658654	-0.4505767	3.2033615
N	-3.3971303	-0.1636231	2.4288260
N	0.6809276	-0.3220107	4.5554656
N	-0.5917898	-0.2641589	4.8646212
C	2.0378896	-0.5453211	2.5671823
C	-1.3330360	-0.3609324	3.7212300
C	-0.4757860	-0.4799714	2.6420166
H	-0.6159989	-0.5759983	1.5656114
C	4.3488138	-1.2156658	2.7081800
C	-3.5480373	-1.2909967	1.6115280
C	3.0807593	-1.1389229	3.2821963
H	2.8769258	-1.5180182	4.2795474
C	2.2144692	-0.0305907	1.2827670
H	1.3626480	0.4289867	0.7762214
C	4.5212127	-0.6974512	1.4144531
H	5.5061803	-0.7708422	0.9559621
C	-3.6660611	1.1566337	2.0220764
C	-2.8301515	-0.3624304	3.7680598
H	-3.2043183	-1.3212117	4.1493521
H	-3.1834008	0.4504461	4.4124894
C	5.5258681	-1.8875884	3.4296250
C	3.4846607	-0.1018140	0.6838675
C	3.7184414	0.3906979	-0.7555362
C	5.2139928	-2.1528074	4.9111853
H	4.9665803	-1.2187771	5.4382285
H	6.0927150	-2.5957802	5.4032292
H	4.3735853	-2.8468121	5.0446463
C	5.8125009	-3.2378151	2.7352066
H	4.9225022	-3.8823397	2.7755489
H	6.6453075	-3.7597905	3.2323655
H	6.0778410	-3.0943962	1.6778311
C	3.3032194	-0.7492551	-1.7092643
H	3.8969964	-1.6573193	-1.5238380
H	3.4343214	-0.4464029	-2.7593289
H	2.2470946	-0.9983904	-1.5661625
C	6.7813618	-0.9915543	3.3586881
H	7.1105070	-0.8166328	2.3255484
H	7.6151160	-1.4670571	3.8977345
H	6.5879607	-0.0121712	3.8203802
C	2.8786861	1.6374870	-1.0840405
H	1.8041445	1.4718197	-0.9471631
H	3.0147916	1.9174962	-2.1373997
H	3.1754329	2.4914619	-0.4601197
C	5.1974827	0.7414636	-1.0108726
H	5.5647747	1.4812095	-0.2840636
H	5.2992789	1.1733729	-2.0168329
H	5.8535327	-0.1403206	-0.9679116
O	2.2069372	-3.5462824	6.0723383
O	2.9432203	0.7026081	6.8656579
N	-0.3428550	-1.2039915	-0.6923111
N	-0.5682180	1.4896416	0.0250836
C	-0.5249401	-0.2570162	-1.6392960
C	-0.9806791	1.0863328	-1.1972236
C	0.0615508	-4.8120256	2.6956494
C	1.4524682	-3.9244307	4.9905873
C	0.1367164	-2.4045778	-1.0646593
C	-0.8427822	2.7426489	0.4249559
C	-0.5322987	-3.9260195	3.6042298
H	-1.5411111	-3.5535274	3.4097755
C	0.4062212	-2.7156579	-2.4071693
H	0.7934092	-3.7027450	-2.6652579
C	-0.2312938	3.1965275	1.7225678
H	-0.2068424	2.3614507	2.4384935
H	-0.8491165	3.9934614	2.1651364
C	1.3737896	-5.2328320	2.9574885
H	1.8687990	-5.9088326	2.2557273
C	2.0673018	-4.8000524	4.0856539
H	3.0903457	-5.1232902	4.2822449
C	2.1320250	3.4650960	3.8087024
C	-0.2367028	-0.4838716	-2.9914949
H	-0.3182429	0.3296628	-3.7065435
C	0.1423232	-3.4881432	4.7451519
H	-0.3493387	-2.7883456	5.4164397
C	0.3735756	-3.4120898	0.0264845
H	0.8160565	-2.9023229	0.8958561
H	1.1080750	-4.1587738	-0.3170328
C	1.2641383	3.3571626	4.8996938

H	0.3674465	3.9799703	4.9312221
C	-0.6776591	-5.2570273	1.4560319
H	-1.6523243	-5.6888752	1.7376958
H	-0.1048028	-6.0606596	0.9638072
C	1.4996965	2.4598035	5.9447784
H	0.7760328	2.3822451	6.7531862
C	0.2231646	-1.7373762	-3.3811322
H	0.4605268	-1.9253917	-4.4292820
C	2.6321794	1.6390240	5.9075893
C	-1.7443938	1.9031289	-2.0390107
H	-2.0940414	1.5439221	-3.0062696
C	-1.5657494	3.6344706	-0.3834758
H	-1.7250367	4.6628780	-0.0614895
C	-0.9224566	-4.1186236	0.4491933
H	-1.4259135	-4.5279573	-0.4428747
H	-1.6097125	-3.3796476	0.8855039
C	-2.0344746	3.1990851	-1.6182417
H	-2.5959128	3.8726579	-2.2611137
C	1.1921762	3.7209229	1.4533587
H	1.1637910	4.4162144	0.5990666
H	1.8162472	2.8711784	1.1413670
C	1.8476575	4.4064141	2.6637508
H	2.7896199	4.8706100	2.3288387
H	1.1996615	5.2283573	3.0106219
C	3.5195012	1.7370186	4.8273934
H	4.3834952	1.0737966	4.7936013
C	3.2618526	2.6295924	3.7925170
H	3.9434301	2.6642987	2.9394138
C	2.8076249	-2.0192308	7.8134764
H	3.1141576	-2.8460052	8.4730136
H	3.6759578	-1.7581160	7.1949989
C	2.4102700	-0.7983014	8.6558311
H	3.2731835	-0.4925669	9.2680575
H	1.5995495	-1.0583546	9.3570805
C	1.6921133	-2.4796107	6.8922776
H	1.3673219	-1.6545194	6.2409049
H	0.8181509	-2.8379789	7.4697155
C	1.9327885	0.3957274	7.8338961
H	1.7620347	1.2649619	8.4963246
H	0.9838502	0.1742219	7.3141415
C	-3.8081365	-1.0471528	0.1472616
H	-2.8092818	-0.9149146	-0.3074377
H	-4.2214124	-1.9816615	-0.2478660
C	-4.1489453	1.3798179	0.6115234
H	-3.2829162	1.7944555	0.0767025
H	-4.8764169	2.1983796	0.6753507
C	-4.6884279	0.1692352	-0.1623694
C	-4.7775794	0.4639721	-1.6618218
C	-5.0957296	1.7581509	-2.1104488
C	-4.5142323	-0.5197051	-2.6153298
C	-5.1056720	2.0831058	-3.4672474
H	-5.2893791	2.5378337	-1.3779119
C	-4.5512662	-0.2455534	-3.9941562
H	-4.2535627	-1.5261856	-2.2912082
C	-4.8309618	1.0611428	-4.3925227
C	-5.3767584	3.5039538	-3.9844750
C	-4.2715427	-1.3733416	-4.9990087
H	-4.8253305	1.3087584	-5.4519560
C	-5.6092183	4.5104146	-2.8453459
C	-4.1688738	3.9821084	-4.8221679
C	-6.6399518	3.4840049	-4.8750137
C	-2.8441214	-1.9086250	-4.7727271
C	-5.2934442	-2.5121441	-4.7862367
C	-4.3803088	-0.8957849	-6.4569495
H	-6.4856355	4.2419999	-2.2364252
H	-4.7375114	4.5878897	-2.1809209
H	-5.7890504	5.5093373	-3.2675921
H	-3.9914848	3.3368508	-5.6926848
H	-4.3410164	5.0055635	-5.1881050
H	-3.2419508	3.9867009	-4.2327418
H	-6.5179835	2.8111017	-5.7352042
H	-7.5159908	3.1449206	-4.3021629
H	-6.8486576	4.4928340	-5.2631138
H	-2.1052634	-1.1121178	-4.9232681
H	-2.7033235	-2.2968294	-3.7551318
H	-2.6164038	-2.7206687	-5.4802538
H	-5.1093964	-3.3272111	-5.5032767
H	-5.2252842	-2.9347056	-3.7742951
H	-6.3212794	-2.1479745	-4.9315121
H	-5.3919881	-0.5348732	-6.6950079

H	-3.6678491	-0.0869162	-6.6717222
H	-4.1525691	-1.7303823	-7.1362284
H	-5.7072979	-0.0514810	0.2065513
C	0.2620618	0.9180594	-6.1665311
C	-0.3728596	-0.1516365	-6.8233990
C	0.3353322	-1.3008497	-7.1748306
C	1.6957940	-1.4397893	-6.8682233
C	2.3217403	-0.3688483	-6.2116063
C	1.6347043	0.7958310	-5.8743657
H	-1.4374321	-0.0856560	-7.0519175
H	-0.1911030	-2.1164137	-7.6771218
H	3.3809954	-0.4470539	-5.9531230
H	2.1465892	1.5988677	-5.3512302
S	-0.7201769	2.3373507	-5.7787685
C	0.1168982	3.1269575	-4.2909805
C	-0.3356025	4.4197116	-4.0672337
H	-1.0064239	4.9043452	-4.7729951
C	0.0080666	5.1672400	-2.8677528
O	0.9019768	2.4366168	-3.5913725
O	0.9189545	4.5785736	-2.0226031
H	1.1270929	3.6791502	-2.4118904
O	-0.4760972	6.2579030	-2.5652674
C	2.4661116	-2.6820864	-7.2393612
H	3.1372038	-2.9959385	-6.4261851
H	1.7922072	-3.5202342	-7.4661359
H	3.0941364	-2.5133116	-8.1296741

13H(+)·9

N	2.1027827	-1.0365333	-6.5123668
N	3.6080499	-2.0858835	-5.4073118
N	3.3293251	-1.6088043	-6.6007877
C	2.5676375	-1.8298513	-4.5621175
C	0.5538555	-1.5250304	-8.3663516
C	1.5883902	-1.1552203	-5.2635597
H	0.6092931	-0.7581082	-4.9977337
C	-0.8326587	-1.4114485	-8.3216926
H	-1.2737998	-0.5728223	-7.7794228
C	1.1482264	-2.6114328	-9.0250919
H	2.2357897	-2.6783178	-9.0259219
C	2.5928588	-2.2294879	-3.1200310
C	1.4350369	-0.4949045	-7.6920096
H	2.2296742	-0.1503372	-8.3674567
H	0.8470682	0.3737133	-7.3662333
C	-1.6577071	-2.3691204	-8.9416558
C	0.3664531	-3.5884315	-9.6440542
C	0.9746185	-4.7639200	-10.4267344
C	-1.0340804	-3.4419771	-9.5839516
H	-1.6510091	-4.1933901	-10.0740861
C	-3.1835149	-2.1892938	-8.9129261
C	0.4217857	-6.1077024	-9.9013350
H	0.7224207	-6.2811792	-8.8593301
H	0.8143573	-6.9366821	-10.5088949
H	-0.6748981	-6.1492500	-9.9513182
C	-3.5420776	-0.8646713	-9.6248987
H	-3.0866499	0.0006911	-9.1239199
H	-4.6326187	-0.7168917	-9.6276428
H	-3.1919376	-0.8748898	-10.6669792
C	2.5092767	-4.8046376	-10.3232190
H	2.9727490	-3.8996755	-10.7414497
H	2.8921163	-5.6650254	-10.8905997
H	2.8472556	-4.9183500	-9.2831538
C	0.5916408	-4.6054363	-11.9169815
H	-0.4978587	-4.6322648	-12.0598955
H	1.0311723	-5.4203436	-12.5117530
H	0.9610572	-3.6491125	-12.3141062
C	-3.6748515	-2.1306194	-7.4509717
H	-3.4625145	-3.0740069	-6.9292445
H	-4.7616847	-1.9622229	-7.4204331
H	-3.1921989	-1.3235114	-6.8846618
C	-3.9236631	-3.3344753	-9.6238174
H	-3.6499693	-3.4021966	-10.6867518
H	-5.0078592	-3.1601729	-9.5715954
H	-3.7205028	-4.3064134	-9.1501329
N	-1.5700321	-1.0968600	-4.6069147
N	-0.4624057	1.4083151	-4.3539244
C	-2.2802130	-0.1456164	-3.9773741
C	-1.7707082	1.2534989	-4.0650724
C	-2.6101768	2.3467172	-3.8024704
H	-3.6700436	2.2046269	-3.5978568
C	-1.9589977	-2.3845125	-4.5282649

O	4.6308728	-5.4294998	-4.0523820
C	0.0731381	2.6411276	-4.3521317
C	-2.0643115	3.6272754	-3.8160277
H	-2.6915077	4.4960138	-3.6120420
C	-3.4265169	-0.4525804	-3.2233226
H	-3.9600936	0.3203702	-2.6730538
C	-0.7075301	3.7797796	-4.0836968
H	-0.2544456	4.7706915	-4.0855071
C	-1.0913443	-3.3704568	-5.2620907
H	-0.0857910	-3.3412229	-4.8118784
H	-0.9511526	-2.9946912	-6.2890423
C	3.4063100	-5.5302406	-4.6597927
C	-3.8368991	-1.7790558	-3.1450245
H	-4.7110928	-2.0493707	-2.5515026
C	0.7670918	-5.7052676	-5.6558829
C	-3.1017364	-2.7601311	-3.8065604
H	-3.4005966	-3.8050870	-3.7482550
C	1.7932669	-5.1333770	-6.4120192
H	1.5563725	-4.6944106	-7.3817908
C	3.1077640	-5.0620428	-5.9451955
H	3.8653308	-4.5853155	-6.5612775
C	-0.6617135	-5.7074497	-6.1475172
H	-1.0630476	-6.7343933	-6.1528776
H	-0.6915260	-5.3485712	-7.1885141
C	1.0971009	-6.2076202	-4.3868312
H	0.3193141	-6.6474068	-3.7594537
C	-1.5799505	-4.8151026	-5.2925461
H	-2.6058418	-4.8665399	-5.6959202
H	-1.6201825	-5.2133598	-4.2649308
C	2.3907136	-6.1172137	-3.8882955
H	2.6357543	-6.4746543	-2.8879431
C	1.5577357	2.7475756	-4.6233172
H	1.7090875	3.0622739	-5.6720414
H	1.9914376	1.7432248	-4.5240347
O	6.1425714	-1.1951936	-2.5110670
C	6.8791937	-4.6544205	-3.8456880
H	7.7745454	-4.4022167	-4.4381800
H	7.1062882	-5.5887116	-3.3100105
C	4.8172619	1.5655382	-4.5905856
H	4.5825355	1.7846589	-5.6350390
C	4.5261794	2.5145956	-3.6073013
C	4.8591532	2.2004767	-2.2801443
H	4.6514532	2.9191064	-1.4828768
C	5.4288425	0.9763656	-1.9500640
H	5.6895064	0.7338923	-0.9192795
C	5.6658593	0.0153836	-2.9446504
C	5.3896880	0.3303224	-4.2809868
H	5.5742775	-0.3830260	-5.0788405
C	2.2927248	3.7305514	-3.6973010
H	2.1239975	3.4323983	-2.6499255
H	1.8766632	4.7446369	-3.8088504
C	6.4105027	-2.1920350	-3.5159523
H	7.3210663	-1.9078624	-4.0770680
H	5.5692878	-2.2362733	-4.2246500
C	6.5883706	-3.5365586	-2.8312602
H	5.6699374	-3.7777834	-2.2749835
H	7.4056807	-3.4738390	-2.0967812
C	3.8129981	3.7994926	-3.9461971
H	4.2251400	4.6246979	-3.3431432
H	3.9933726	4.0612898	-5.0018899
C	5.7337738	-4.9146605	-4.8178015
H	6.0242292	-5.6570403	-5.5827757
H	5.4309092	-3.9876224	-5.3304954
O	2.2928547	0.3820197	-2.5227764
O	0.5124819	-3.8404184	-2.2642525
O	-3.4975199	2.9511595	0.6349574
N	1.4084179	-1.7255807	-2.4011883
O	-4.5162162	-1.3129884	1.9959111
N	-4.0227781	0.8217540	1.3048769
N	-2.8479627	2.3389535	5.3512389
C	-0.8289118	-0.6987752	-1.0192551
C	1.3639241	-0.3330836	-2.1698412
N	-2.5570190	1.2438016	3.5368934
C	0.1682375	0.1905257	-1.4793696
C	-0.7303554	-2.0887911	-1.2475977
N	-1.9483685	1.6392003	4.6346087
C	-2.0671255	1.2125558	-0.1245610
C	0.4156259	-2.6421438	-2.0100113
C	-1.0964357	2.0704214	-0.6257028
H	-1.2249670	3.1436109	-0.4926846

C	-2.8449630	-1.0849052	0.3159634
C	-2.7244248	-2.4521365	0.0862441
H	-3.4335463	-3.1260889	0.5656828
C	-1.9277503	-0.1866462	-0.2745279
C	-3.8470899	1.6970349	3.5425916
C	0.0422942	1.5556906	-1.2580958
H	0.8364739	2.2122118	-1.6045249
C	-3.2283456	1.7548134	0.5978904
C	-1.1888089	2.4178874	7.1849437
C	-4.0403610	2.4121645	4.7050923
H	-4.8936977	2.9489562	5.0971269
C	-1.6893308	-2.9474397	-0.7193162
H	-1.5913986	-4.0145003	-0.9156920
C	-0.0435437	3.1948981	7.0091631
H	-0.1400660	4.1512815	6.4945759
C	-1.1034895	1.1697978	7.8037161
H	-2.0104389	0.5717973	7.8928456
C	-4.7955072	1.3799019	2.4216964
H	-5.5313108	0.6266452	2.7275889
H	-5.3146147	2.2822101	2.0781712
C	-2.5088822	2.9144727	6.6635955
H	-3.3313068	2.6372548	7.3399022
H	-2.4958496	4.0070892	6.5453285
C	-3.8601798	-0.5712704	1.2640983
C	1.2036964	2.7419138	7.4545041
C	0.1272076	0.6722399	8.2466012
C	0.2285124	-0.6914289	8.9473604
C	1.2579639	1.4824637	8.0672939
H	2.2230265	1.1077848	8.3983944
C	2.4538616	3.6218852	7.3196431
C	1.6299121	-1.3127736	8.7855049
H	1.9123439	-1.3932399	7.7273121
H	1.6354894	-2.3246610	9.2150349
H	2.4028235	-0.7335571	9.3098443
C	2.5344606	4.5345096	8.5648529
H	1.6341088	5.1609510	8.6517956
H	3.4127362	5.1961006	8.5030561
H	2.6188956	3.9343920	9.4823410
C	-0.7973622	-1.6936920	8.3811316
H	-1.8301329	-1.3546547	8.5423159
H	-0.6913143	-2.6605621	8.8947650
H	-0.6354569	-1.8586872	7.3076900
C	-0.0566473	-0.4705532	10.4509706
H	0.6671123	0.2333410	10.8876456
H	0.0104579	-1.4221571	11.0001801
H	-1.0646275	-0.0554722	10.6013802
C	2.3623311	4.5055332	6.0592210
H	2.1369556	3.8935803	5.1756387
H	3.3143094	5.0295642	5.8893093
H	1.5811432	5.2738590	6.1503120
C	3.7381108	2.7745070	7.2322826
H	3.9497168	2.2526728	8.1761779
H	4.6007237	3.4226817	7.0216176
H	3.6623255	2.0235305	6.4350269
N	-0.8499495	-1.4403981	2.9633269
N	0.5080148	0.6222555	2.1628368
C	0.2055825	-1.7156683	2.1539644
C	0.9990691	-0.5487123	1.7327405
C	2.1575150	-0.6496495	0.9491922
H	2.5480158	-1.6116583	0.6216289
C	-1.6468370	-2.3707021	3.5371960
O	-6.8933359	2.4789423	6.7064645
C	1.1536623	1.7608305	1.8702916
C	2.8030723	0.5315454	0.6022736
H	3.6858960	0.5117722	-0.0320941
C	0.4412502	-3.0425770	1.8043670
H	1.2720682	-3.2939861	1.1506865
C	2.3012953	1.7477724	1.0600496
H	2.8234286	2.6713440	0.8167335
C	-2.5813552	-1.8957598	4.6022318
H	-3.1280288	-1.0159498	4.2339512
H	-1.9231392	-1.5178657	5.4056966
C	-6.2361889	1.2727581	6.7396608
C	-0.4129864	-4.0275777	2.2876352
H	-0.2516197	-5.0677865	2.0038897
C	-4.8742555	-1.1980590	6.5397333
C	-1.4575797	-3.6968690	3.1485030
H	-2.0984962	-4.4705125	3.5604962
C	-4.7025554	-0.3576645	7.6440979
H	-4.0337743	-0.6671946	8.4499626

C	-5.3704593	0.8667001	7.7607215
H	-5.2089243	1.4814182	8.6447171
C	-4.1729811	-2.5376211	6.4879535
H	-4.8867070	-3.3235620	6.7893731
H	-3.3758420	-2.5436770	7.2469107
C	-5.7576759	-0.7733237	5.5311234
H	-5.9019529	-1.3874878	4.6404066
C	-3.5552858	-2.9416102	5.1396458
H	-3.0348355	-3.8960994	5.3111703
H	-4.3354082	-3.1355092	4.3870875
C	-6.4355560	0.4366722	5.6301056
H	-7.1075298	0.7738034	4.8398175
C	0.6012454	2.9896092	2.5326887
H	1.0009785	2.9678884	3.5619760
H	-0.4820140	2.8469062	2.6494067
O	-4.9608922	5.5926405	4.4336963
C	-7.2251299	4.7724977	7.3026095
H	-6.9816756	5.5218650	8.0740183
H	-8.3184398	4.6503238	7.3172564
C	-1.2703725	5.9869464	4.5203947
H	-0.4775951	6.3088138	5.1996497
C	-0.9331896	5.5398289	3.2388090
C	-1.9850120	5.1361277	2.3990101
H	-1.7787548	4.7524129	1.3997905
C	-3.3079533	5.1718256	2.8196960
H	-4.1029547	4.8146510	2.1676194
C	-3.6249821	5.6229111	4.1071389
C	-2.5992981	6.0411019	4.9630324
H	-2.8183637	6.4121517	5.9640711
C	0.9141772	4.3356208	1.8832021
H	0.4197769	4.4027678	0.8999316
H	1.9940379	4.4232639	1.6878534
C	-5.3254688	5.6540550	5.8170766
H	-5.1580848	6.6686205	6.2230480
H	-4.6844762	4.9610206	6.3922322
C	-6.7955324	5.2551529	5.9095224
H	-6.9719349	4.4460651	5.1865619
H	-7.4242534	6.1005808	5.5944058
C	0.5092893	5.5227901	2.7745304
H	0.7228332	6.4602638	2.2315081
H	1.1625953	5.5311029	3.6609038
C	-6.5822726	3.4517313	7.7147412
H	-6.9656170	3.1195201	8.6952355
H	-5.4856783	3.5549381	7.8020466
H	3.4900769	-1.8168024	-2.6359108
H	2.5946164	-3.3229419	-3.0221002
H	-0.9623344	-0.4310658	3.1920963
C	4.8730129	1.6731923	3.0435489
C	5.6476169	1.6631747	1.8714702
C	5.8359134	2.8329036	1.1348830
C	5.2441425	4.0435479	1.5241840
C	4.4966865	4.0474370	2.7125555
C	4.3248342	2.8922409	3.4733420
H	6.0864378	0.7253091	1.5258842
H	6.4398269	2.7978130	0.2258167
H	4.0413138	4.9786574	3.0609851
H	3.7446246	2.9200559	4.3873549
S	4.6718730	0.1406757	3.9071744
C	2.9007489	0.1085254	4.4572738
C	2.4624095	-1.1997597	4.6940510
H	3.1152450	-2.0479392	4.5021798
C	1.1309607	-1.4953008	5.1574683
O	2.2416323	1.1688246	4.5994653
O	0.2905897	-0.4320472	5.3567733
H	0.7975967	0.4084439	5.1390737
O	0.6771775	-2.6334656	5.3425569
C	5.4169590	5.2989128	0.7072525
H	4.4503324	5.7919652	0.5223229
H	6.0566158	6.0300997	1.2261485
H	5.8797854	5.0838402	-0.2656247

$13H_2(2+)\cdot 9$

N	1.6048821	-1.9701280	-6.7510422
N	0.5699635	-0.9073488	-5.2139193
N	0.7571540	-0.9500574	-6.5171000
C	1.2971466	-1.8908607	-4.6077402
C	3.4322959	-1.3808361	-8.2832265
C	1.9654457	-2.5833000	-5.5976736
H	2.6154671	-3.4492816	-5.5768589
C	4.6752592	-2.0051682	-8.3497679
H	4.7247709	-3.0942090	-8.2746486
C	3.3406853	0.0155428	-8.3799972
H	2.3536685	0.4731891	-8.3149779
C	1.3772214	-2.0989969	-3.1303614
C	2.1666347	-2.1881925	-8.0870229
H	1.3880241	-1.8970778	-8.8039155
H	2.3525184	-3.2656194	-8.1854989
C	5.8520291	-1.2536205	-8.5359392
C	4.4854317	0.7984891	-8.5374573
C	4.4348390	2.3277920	-8.6699902
C	5.7255088	0.1353225	-8.6141636
H	6.6219428	0.7360881	-8.7555251
C	7.2051632	-1.9677763	-8.6777557
C	5.3754580	2.9806864	-7.6318151
H	5.0957287	2.7028554	-6.6063205
H	5.3288104	4.0760486	-7.7193768
H	6.4214391	2.6827974	-7.7840277
C	7.1421075	-2.9282172	-9.8870930
H	6.3675886	-3.6969979	-9.7581077
H	8.1060537	-3.4421024	-10.0155034
H	6.9205583	-2.3771130	-10.8118379
C	3.0158956	2.8842192	-8.4596070
H	2.3112310	2.5133653	-9.2179825
H	3.0347530	3.9805739	-8.5353591
H	2.6176831	2.6267394	-7.4664268
C	4.8999221	2.7152844	-10.0930927
H	5.9284345	2.3806204	-10.2863822
H	4.8719253	3.8077135	-10.2186227
H	4.2484766	2.2629189	-10.8542959
C	7.4954563	-2.7780596	-7.3969545
H	7.5607574	-2.1150741	-6.5224379
H	8.4502018	-3.3162580	-7.4902818
H	6.7099344	-3.5198013	-7.2035257
C	8.3686382	-0.9867751	-8.8997004
H	8.2393502	-0.4033803	-9.8222012
H	9.3090308	-1.5475474	-8.9933097
H	8.4829823	-0.2881867	-8.0582570
N	-2.3267016	-1.5684369	-4.8645839
N	-1.4597220	0.9448050	-4.5357799
C	-3.1659268	-0.7509370	-4.2147676
C	-2.7472698	0.6659445	-4.1827713
C	-3.5781052	1.7195122	-3.8068016
H	-4.6115593	1.5233538	-3.5334538
C	-2.5890785	-2.8835897	-4.9307216
O	4.5338742	-5.7864133	-4.4683877
C	-0.9245209	2.1887312	-4.5107883
C	-3.0750685	3.0181664	-3.7880815
H	-3.7204827	3.8459661	-3.4935838
C	-4.3318749	-1.2052796	-3.5806859
H	-4.9800932	-0.5357879	-3.0179925
C	-1.7481206	3.2538000	-4.1344653
H	-1.3344191	4.2595176	-4.1122520
C	-1.5617090	-3.6984431	-5.6693321
H	-0.6078369	-3.5620086	-5.1355051
H	-1.4096171	-3.2250509	-6.6543637
C	3.3037855	-5.8149446	-5.0603885
C	-4.6115373	-2.5683455	-3.6471407
H	-5.4982210	-2.9680783	-3.1548520
C	0.6588333	-5.8267783	-6.0780888
C	-3.7394365	-3.4186344	-4.3267400
H	-3.9427999	-4.4868196	-4.3766646
C	1.7684938	-5.8318200	-6.9317512
H	1.6092613	-5.8562623	-8.0127431
C	3.0821619	-5.8299464	-6.4461036
H	3.9160554	-5.8583009	-7.1458625
C	-0.7459137	-5.8991175	-6.6454231
H	-1.0262507	-6.9604949	-6.7555877
H	-0.7366204	-5.4861449	-7.6675682
C	0.9066886	-5.8127653	-4.6941616
H	0.0793121	-5.7768005	-3.9857352
C	-1.8388236	-5.1900024	-5.8306309

H	-2.8039564	-5.3475817	-6.3386064
H	-1.9312077	-5.6599046	-4.8379638
C	2.2024073	-5.8066037	-4.1898639
H	2.3870295	-5.7861527	-3.1154725
C	0.5194148	2.3540052	-4.8764864
H	0.5723602	2.7354127	-5.9117291
H	0.9998962	1.3690823	-4.8764720
O	5.1995261	-1.7490264	-2.9652654
C	6.7553249	-4.9132967	-4.3214341
H	7.5763239	-4.5175720	-4.9406878
H	7.1638186	-5.7664405	-3.7608332
C	3.6932907	1.0035789	-4.9293080
H	3.4284382	1.2353447	-5.9609108
C	3.4446792	1.9356817	-3.9194074
C	3.7788631	1.5760999	-2.6036810
H	3.5766059	2.2682362	-1.7826941
C	4.3549593	0.3453618	-2.3177783
H	4.6248973	0.0718214	-1.2971158
C	4.6216414	-0.5690317	-3.3514896
C	4.2832574	-0.2373460	-4.6703209
H	4.4715456	-0.9146060	-5.5023817
C	1.2884446	3.2961436	-3.9360364
H	1.1119817	2.9757810	-2.8983095
H	0.9248332	4.3310675	-4.0356079
C	5.5895147	-2.6662804	-3.9960963
H	6.2700712	-2.1644366	-4.7071068
H	4.6948338	-2.9870615	-4.5587131
C	6.2665064	-3.8500138	-3.3256004
H	5.5552072	-4.3048764	-2.6219431
H	7.1170181	-3.4829640	-2.7333023
C	2.8039791	3.2703185	-4.2171311
H	3.2804535	4.0525378	-3.6059665
H	2.9839260	3.5448696	-5.2690724
C	5.6658829	-5.4043671	-5.2664379
H	6.0136190	-6.2599243	-5.8705025
H	5.3643391	-4.6012265	-5.9618219
O	0.8870865	0.4647467	-2.5170008
O	-0.6048199	-3.8678253	-2.4493463
O	-4.9888196	2.5177249	0.8180139
N	0.1537058	-1.7007630	-2.4263821
O	-5.7112348	-1.8603618	2.0094999
N	-5.3584248	0.3360969	1.4282382
N	-3.9131001	1.8426402	5.3805719
C	-2.1301425	-0.8874990	-1.0224251
C	0.0229433	-0.3285652	-2.1464259
N	-3.7466966	0.7444394	3.5531372
C	-1.1849663	0.0849566	-1.4264350
C	-1.9593799	-2.2579195	-1.3276562
N	-3.0593841	1.1616626	4.5954492
C	-3.4686924	0.9041636	-0.0272625
C	-0.7875111	-2.6981597	-2.1061334
C	-2.5428051	1.8443216	-0.4667877
H	-2.7285971	2.8965911	-0.2564862
C	-4.1283706	-1.4502465	0.2816178
C	-3.9424798	-2.7961246	-0.0299922
H	-4.6125953	-3.5314152	0.4144648
C	-3.2584920	-0.4750714	-0.2609807
C	-5.0438243	1.1647886	3.6643767
C	-1.3811624	1.4313901	-1.1260042
H	-0.6178791	2.1479948	-1.4230091
C	-4.6507970	1.3419257	0.7337756
C	-2.1491733	1.8974044	7.1181871
C	-5.1562272	1.8858381	4.8345029
H	-5.9836287	2.4118608	5.2919966
C	-2.8825488	-3.1925060	-0.8565807
H	-2.7313811	-4.2416159	-1.1083652
C	-0.9907482	2.6458082	6.9035665
H	-1.0807254	3.6084210	6.3990076
C	-2.0747023	0.6472525	7.7347019
H	-2.9938905	0.0752131	7.8615057
C	-6.0713106	0.8028501	2.6282761
H	-6.7141236	-0.0156707	2.9733774
H	-6.6909786	1.6671424	2.3646298
C	-3.4773065	2.4335173	6.6581633
H	-4.2708720	2.2009982	7.3837335
H	-3.4317414	3.5218835	6.5151127
C	-5.1321206	-1.0425885	1.2967086
C	0.2585981	2.1625591	7.3117360
C	-0.8432212	0.1191477	8.1383155
C	-0.7542821	-1.2412813	8.8459898

C	0.3008058	0.9011809	7.9215971
H	1.2652355	0.5058403	8.2296698
C	1.5246792	3.0162657	7.1565251
C	0.6295344	-1.8933796	8.6572848
H	0.8853217	-1.9889368	7.5931581
H	0.6251212	-2.9010614	9.0958276
H	1.4272877	-1.3269572	9.1573669
C	1.6436158	3.9244600	8.4022261
H	0.7623826	4.5756242	8.4995455
H	2.5393147	4.5608047	8.3321775
H	1.7205979	3.3208407	9.3177308
C	-1.8145931	-2.2250693	8.3117457
H	-2.8349793	-1.8621617	8.4945290
H	-1.7184785	-3.1900544	8.8302226
H	-1.6847774	-2.4014466	7.2355654
C	-1.0002522	-1.0020511	10.3536695
H	-0.2535251	-0.3087904	10.7673424
H	-0.9391513	-1.9494638	10.9099051
H	-1.9960540	-0.5659367	10.5232615
C	1.4337951	3.9057980	5.9004850
H	1.1964032	3.3022092	5.0137937
H	2.3900661	4.4213834	5.7282460
H	0.6618990	4.6819084	6.0017669
C	2.7907916	2.1436093	7.0508661
H	3.0095846	1.6219805	7.9929137
H	3.6623940	2.7738053	6.8235381
H	2.6879408	1.3894037	6.2595805
N	-2.1221890	-1.8972089	2.9322801
N	-0.7319373	0.1541841	2.1160510
C	-1.1022413	-2.1786475	2.0800194
C	-0.2850451	-1.0246960	1.6611107
C	0.8666144	-1.1493619	0.8698375
H	1.2271080	-2.1194493	0.5319776
C	-2.9116660	-2.8239683	3.5241881
O	-7.9076055	2.0406012	6.9921259
C	-0.0472242	1.2761437	1.8428719
C	1.5533320	0.0155248	0.5438672
H	2.4443107	-0.0223777	-0.0809947
C	-0.9066650	-3.5028982	1.6998231
H	-0.1033438	-3.7593822	1.0144496
C	1.0958346	1.2397836	1.0262446
H	1.6515173	2.1486386	0.8038004
C	-3.7878871	-2.3594750	4.6421380
H	-4.3470225	-1.4707248	4.3163758
H	-3.0876800	-1.9973111	5.4172137
C	-7.2737512	0.8239523	6.9859208
C	-1.7631536	-4.4812306	2.1960023
H	-1.6322705	-5.5197457	1.8911539
C	-5.9703548	-1.6700630	6.6975202
C	-2.7645752	-4.1466794	3.1034127
H	-3.4046201	-4.9146593	3.5272711
C	-5.6925824	-0.8219545	7.7736373
H	-4.9670564	-1.1361384	8.5268026
C	-6.3303239	0.4129945	7.9340057
H	-6.0894658	1.0301071	8.7979048
C	-5.2928383	-3.0183026	6.5954020
H	-6.0024004	-3.8008315	6.9134595
H	-4.4636856	-3.0482216	7.3180181
C	-6.9364776	-1.2444225	5.7685826
H	-7.1810502	-1.8775859	4.9130440
C	-4.7414111	-3.4072307	5.2133287
H	-4.2204684	-4.3673719	5.3464887
H	-5.5579136	-3.5852548	4.4961397
C	-7.5841621	-0.0222558	5.9091243
H	-8.3259022	0.3120198	5.1824261
C	-0.5454671	2.5105747	2.5361480
H	-0.1138097	2.4715109	3.5521812
H	-1.6267516	2.3932732	2.6895296
O	-5.9886447	5.1191368	4.6669015
C	-8.1618807	4.3380979	7.6128788
H	-7.8751962	5.0768590	8.3793720
H	-9.2556414	4.2364534	7.6687838
C	-2.3017060	5.5342063	4.5968347
H	-1.4833365	5.8688568	5.2385444
C	-2.0159149	5.0714966	3.3082266
C	-3.1019191	4.6602377	2.5171605
H	-2.9343744	4.2775433	1.5100151
C	-4.4061907	4.6940757	2.9914437
H	-5.2294316	4.3344163	2.3774347
C	-4.6705565	5.1547788	4.2879286

C	-3.6110418	5.5888754	5.0941386
H	-3.7893239	5.9714113	6.0988077
C	-0.2217527	3.8557011	1.8886693
H	-0.7346920	3.9331364	0.9150232
H	0.8558369	3.9275329	1.6719167
C	-6.3038969	5.1964391	6.0637382
H	-6.1065051	6.2106859	6.4549148
H	-5.6532863	4.4961002	6.6189277
C	-7.7762254	4.8225382	6.2077532
H	-7.9938967	4.0219410	5.4864338
H	-8.4006029	5.6809699	5.9216525
C	-0.5919789	5.0480579	2.7897669
H	-0.3930516	5.9820960	2.2358322
H	0.0929312	5.0572082	3.6514486
C	-7.5285924	3.0032516	7.9894105
H	-7.8713964	2.6713452	8.9842264
H	-6.4271054	3.0838650	8.0239282
H	2.2006394	-1.4935277	-2.7241018
H	1.5582499	-3.1587754	-2.9212175
H	-2.2200662	-0.8907641	3.1790053
C	3.8113187	1.0091587	2.8154352
C	4.5321927	0.9892183	1.6088366
C	4.7290125	2.1635261	0.8836841
C	4.2006611	3.3883495	1.3209956
C	3.5099725	3.3994182	2.5426387
C	3.3319235	2.2383400	3.2934290
H	4.9240366	0.0424639	1.2322380
H	5.2988220	2.1263056	-0.0471757
H	3.1080445	4.3402703	2.9272196
H	2.8016225	2.2749132	4.2369846
S	3.5737510	-0.5339533	3.6487780
C	1.8318008	-0.4928751	4.2640551
C	1.3526942	-1.7872433	4.5263917
H	1.9752626	-2.6593900	4.3405396
C	0.0125042	-2.0373115	5.0069840
O	1.2082608	0.5834004	4.4148072
O	-0.7841684	-0.9522502	5.2331941
H	-0.2581085	-0.1250149	5.0196902
O	-0.4681768	-3.1635890	5.1792832
C	4.4051679	4.6535926	0.5283806
H	3.5138004	5.2964205	0.5608803
H	5.2437358	5.2425012	0.9325262
H	4.6367987	4.4370447	-0.5239711
H	-0.8011862	0.1315827	-4.7900198

S11. REFERENCES

[1] A. Pigorsch, M. Kockerling, *Cryst.Growth Des.* **2016**, *16*, 4240–4246.

[2] J. E. M. Lewis, R. J. Bordoli, M. Denis, C. J. Fletcher, M. Galli, E. A. Neal, E. M. Rochette, S. M. Goldup, *Chem. Sci.* **2016**, *7*, 3154-3161.

[3] H. Lahlali, K. Jobe, M. Watkinson, S. M. Goldup, *Angew. Chem. Int. Ed.* **2011**, *50*, 4151-4155.

[4] P. Ruminski, D. Griggs, S. Seiwert, *Integrin Antagonists*, WO2018132268, **2018**.

[5] N. Sakai, N. Sordé, S. Matile, *Molecules* **2001**, *6*, 845–851.

[6] Y. Yu, Y. Li, S. Chen, T. Liu, Z. Qin, H. Liu, Y. Li, *Eur. J. Org. Chem.* **2012**, *23*, 4287–4292.

[7] MestReNova 11.0.4 © 2017 MestreLab Research S.L.

[8] Y. Cotelle, S. Benz, A.-J. Avestro, T. R. Ward, N. Sakai, S. Matile, *Angew. Chem. Int. Ed.* **2016**, 55, 4275–4279.

[9] A.-B. Bornhof, A. Bauzá, A. Aster, M. Pupier, A. Frontera, E. Vauthey, N. Sakai, S. Matile, *J. Am. Chem. Soc.* **2018**, *140*, 4884–4892.

[10] J. López-Andarias, A. Bauzá, N. Sakai, A. Frontera, S. Matile, *Angew. Chem. Int. Ed.* **2018**, *57*, 10883–10887.

[11] A.-T. Pham, S. Matile, *Chem. Asian J.* **2020**, *15*, 1562.

[12] R. Ahlrichs, M. Bär, M. Hacer, H. Horn, C. Kömel, *C. Chem. Phys. Lett.* **1989**, *162*, 165–169.

[13] S. Grimme, J. Antony, S. Ehrlich, H. Krieg, *J. Chem. Phys.* **2010**, *132*, 154104-154119.

[14] A. Klamt, G. Schüürmann, *J. Chem. Soc., Perkin Trans. 2* **1993**, 799-805.

[15] A. Klampt, A. *WIREs Comput. Mol. Sci.* **2011**, *1*, 699-709.

[16] Gaussian 16, Revision C.01, M. J. Frisch, G. W. Trucks, H. B. Schlegel, G. E. Scuseria, M. A. Robb, J. R. Cheeseman, G. Scalmani, V. Barone, G. A. Petersson, H. Nakatsuji, X. Li, M. Caricato, A. V. Marenich, J. Bloino, B. G. Janesko, R. Gomperts, B. Mennucci, H. P. Hratchian, J. V. Ortiz, A. F. Izmaylov, J. L. Sonnenberg, D. Williams-Young, F. Ding, F. Lipparini, F. Egidi, J. Goings, B. Peng, A. Petrone, T. Henderson, D. Ranasinghe, V. G. Zakrzewski, J. Gao, N. Rega, G. Zheng, W. Liang, M. Hada, M. Ehara, K. Toyota, R. Fukuda, J. Hasegawa, M. Ishida, T. Nakajima, Y. Honda, O. Kitao, H. Nakai, T. Vreven, K. Throssell, J. A. Montgomery, Jr., J. E. Peralta, F. Ogliaro, M. J. Bearpark, J. J. Heyd, E. N. Brothers, K. N. Kudin, V. N. Staroverov, T. A. Keith, R. Kobayashi, J. Normand, K. Raghavachari, A. P. Rendell, J. C. Burant, S. S. Iyengar, J. Tomasi, M. Cossi, J. M. Millam, M. Klene, C. Adamo, R. Cammi, J. W. Ochterski, R. L. Martin, K. Morokuma, O. Farkas, J. B. Foresman, and D. J. Fox, Gaussian, Inc., Wallingford CT, 2016.

[17] J. Ho, M. L. Coote, *Wiley Interdiscip. Rev.: Comput. Mol. Sci.*, **2011**, *1*, 649–660

[18] A. Bolag, J. López-Andarias, S. Lascano, S. Soleimanpour, C. Atienza, N. Sakai, N. Martín, S. Matile, *Angew. Chem. Int. Ed.* **2014**, *53*, 4890–4895.

[19] L. Liu, Y. Cotelle, J. Klehr, N. Sakai, T. R. Ward, S. Matile, *Chem. Sci.* **2017**, *8*, 3770–3774.

[20] X. Zhang, L. Liu, J. López-Andarias, C. Wang, N. Sakai, S. Matile, *Helv. Chim. Acta* **2018**, *101*, e1700288.

[21] Y. Zhao, C. Beuchat, Y. Domoto, J. Gajewy, A. Wilson, J. Mareda, N. Sakai, S. Matile, *J. Am. Chem. Soc.* **2014**, *136*, 2101–2111.
